# Proteomic Candidate Biomarkers of Drug-Induced Nephrotoxicity in the Rat

**DOI:** 10.1371/journal.pone.0034606

**Published:** 2012-04-11

**Authors:** Rodney Rouse, Justyna Siwy, William Mullen, Harald Mischak, Jochen Metzger, Joseph Hanig

**Affiliations:** 1 Division of Drug Safety Research, Center for Drug Evaluation and Research, US Food and Drug Administration, Silver Spring, Maryland, United States of America; 2 Office of Pharmaceutical Science, Center for Drug Evaluation and Research, US Food and Drug Administration, Silver Spring, Maryland, United States of America; 3 Mosaiques Diagnostics GmbH, Hannover, Germany; 4 BHF Glasgow Cardiovascular Research Centre, University of Glasgow, Glasgow, United Kingdom; IIT Research Institute, United States of America

## Abstract

Improved biomarkers of acute nephrotoxicity are coveted by the drug development industry, regulatory agencies, and clinicians. In an effort to identify such biomarkers, urinary peptide profiles of rats treated with two different nephrotoxins were investigated. 493 marker candidates were defined that showed a significant response to cis-platin comparing a cis-platin treated cohort to controls. Next, urine samples from rats that received three consecutive daily doses of 150 or 300 mg/kg gentamicin were examined. 557 potential biomarkers were initially identified; 108 of these gentamicin-response markers showed a clear temporal response to treatment. 39 of the cisplatin-response markers also displayed a clear response to gentamicin. Of the combined 147 peptides, 101 were similarly regulated by gentamicin or cis-platin and 54 could be identified by tandem mass spectrometry. Most were collagen type I and type III fragments up-regulated in response to gentamicin treatment. Based on these peptides, classification models were generated and validated in a longitudinal study. In agreement with histopathology, the observed changes in classification scores were transient, initiated after the first dose, and generally persistent over a period of 10–20 days before returning to control levels. The data support the hypothesis that gentamicin-induced renal toxicity up-regulates protease activity, resulting in an increase in several specific urinary collagen fragments. Urinary proteomic biomarkers identified here, especially those common to both nephrotoxins, may serve as a valuable tool to investigate potential new drug candidates for the risk of nephrotoxicity.

## Introduction

The drug development industry, government regulatory agencies, and healthcare professionals are all major stakeholders in the development of biomarkers of drug-induced injury. The advancement of organ-specific biomarkers of drug-induced injury promises each stakeholder improved efficiency and effectiveness in the drug development process that ultimately results in safe and efficacious products coming to the market. The United States Food and Drug Administration (U. S. FDA) has gone as far as describing an official biomarker qualification process [1] to hasten adoption of candidate biomarkers. Kidney injury biomarkers were the logical test case for the biomarker qualification process. Classical, functional biomarkers of kidney injury (blood urea nitrogen, serum creatinine), while accessible (serum, plasma), are not sensitive or specific to etiology or location of injury, leaving considerable room for biomarker improvement. Individual biomarkers have been evaluated and qualified by the U. S. FDA based on validated immune-based assays and data packages put together by industrial consortia [2]. Ongoing evaluation of these biomarkers suggests that specific insults may illicit different biomarker responses and that building biomarker profiles might be the ultimate tool for identifying injury [3]–[5]. As a consequence, there is a specific need for additional biomarkers that enable the generation of such specific biomarker profiles. Ideally, the biomarkers should be easily accessible in a non-invasive way, and should be applicable in animal models, as well as in man.

“Omic” technologies (genomics, metabolomics, proteomics, etc.) hold the promise to fulfil this need and enable identification of multiple biomarkers that reflect specific types of injury in the kidney. Several proteomics approaches have been described in this context [6]–[9]. CE-MS methodology was validated as an analytical tool for the measurement of peptides in rat urine and subsequently used to profile the urinary low-molecular proteome of the rat [10]. In an earlier publication [11], CE-MS was used as a biomarker discovery tool for nephrotoxicity in rats treated with cis-platin. In the study reported here we aimed to identify common and disparate biomarkers of cis-platin- and gentamicin-induced nephrotoxicity by applying CE-MS proteomics in rat urine. The aim of the study was to detect multiple biomarkers that can be efficiently analysed in a non-invasive approach. Such biomarkers could form the basis for specific multi-marker models for displaying drug-induced kidney injury in pre-clinical and clinical application and may have substantial translational value.

## Results

### Identification of the rat urinary peptides indicative of cis-platin and gentamicin induced nephrotoxicity

**Figure 1:**
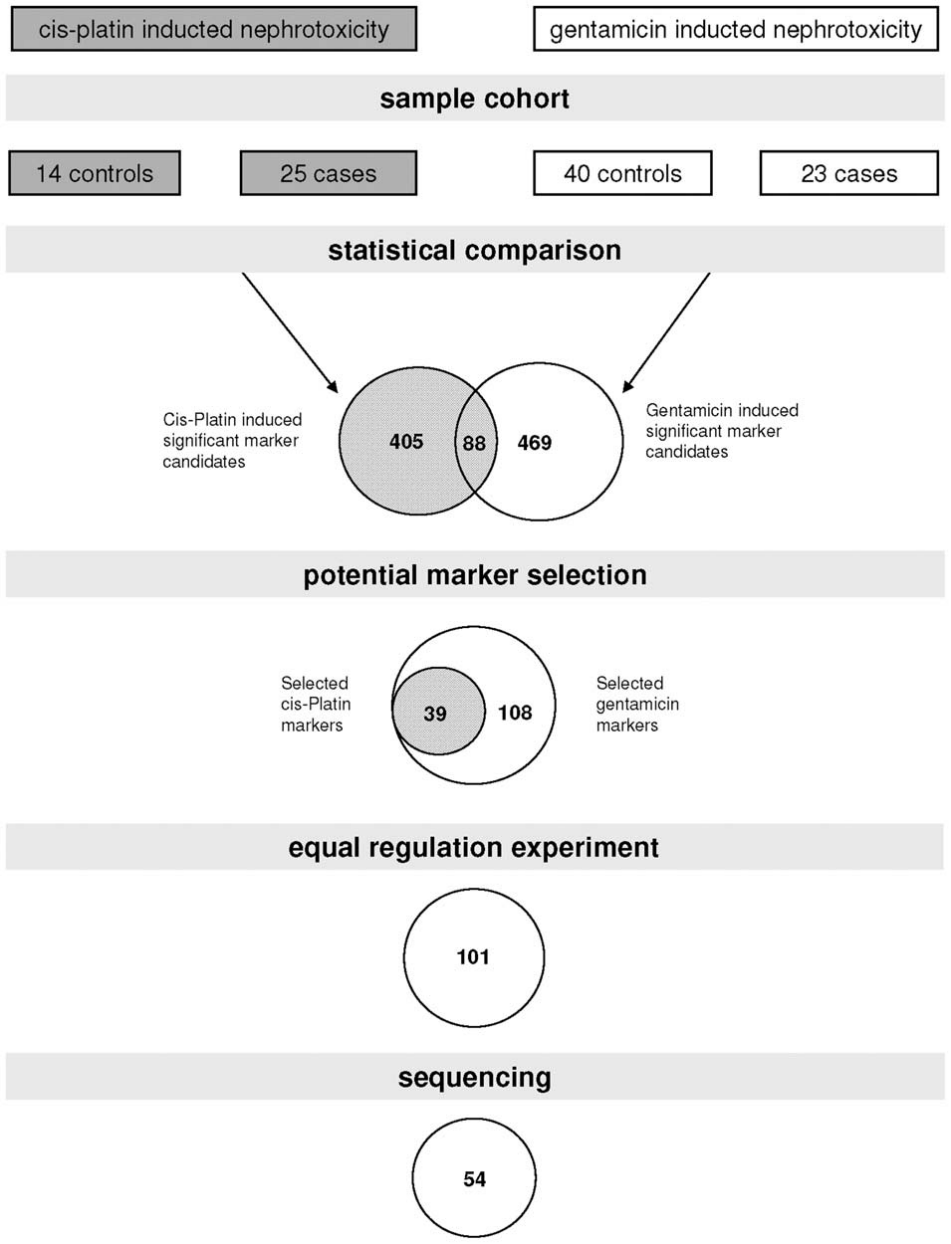
Study design and rationale for biomarker selection. Depicted is the size of the sample cohorts used in the biomarker definition step and the number of selected peptides after statistical comparison of the respective treated and control groups. Numbers of peptides identified in both cis-platin and genatmicin treated rats as markers are denominated in the intersection of the diagrams. The significant marker candidates were visually inspected and only those candidates were selected that showed a clear response to gentamicin. In the next step the direction of regulation was controlled for the markers identified in both studies and selected were only those demonstrating similar (up or down) regulation with both cis-platin and gentamicin. In the last step the number of markers for which the amino acid sequence could be resolved is listed.

Two parallel approaches were employed to identify potential biomarkers for drug-induced nephrotoxicity. The study design is graphically depicted in **figure 1**. In the first approach, CE-MS data generated in a previous study [11] were used as an initial reference to identify biomarkers for cis-platin-induced nephrotoxicity (**table S1 sheet 1**). The comparison of the urinary peptides and proteins from 14 controls and 25 treated animals resulted in identification of 493 peptides that showed statistically significant changes in distribution (*P*<0.05 after correction for multiple testing).

In the second approach, rat urine samples from a gentamicin study [12] were used as a second source for nephrotoxicity biomarker discovery. Samples were analyzed using CE-MS and the data were matched against a previously established rat urinary proteome database [10], [11]. The compiled proteomic profiles of all samples in this study are shown according to dose and treatment duration in **figure S1**. Urine samples collected at days 3, 7 or 10 (the first samples taken after treatment) from rats treated once daily for three consecutive days with 150 mg/kg or 300 mg/kg gentamicin (n = 23) or saline (from all time points n = 40) were analyzed as cases and controls, respectively, to form the discovery set (**table S1 sheet 2**). A total of 557 potential biomarker candidates could be defined (*P*<0.05). Of this peptide pool, 88 peptides intersected with the 493 peptides profiled in the cis-platin study (**figure 1**).

**Figure 2:**
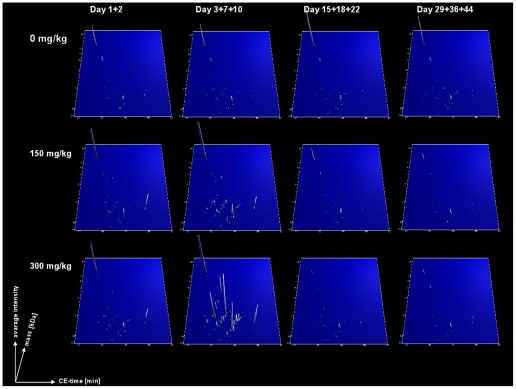
Group specific polypeptide profiles for the 101 drug-induced nephrotoxicity markers in the gentamicin-treated rat cohort. The compiled data sets of urine samples from gentamicin-treated rats at different doses and sampling days are shown. The data sets were divided into four groups according to the days of sampling after initiation of treatment (samples collected at day 1+2; day 3+7+10; day 15+18+22 and day 29+36+44 from left to right) and for the gentamicin dose (0, 150 and 300 mg/kg gentamicin from the top to bottom). Molecular mass of the analyzed polypeptides (kDa) in logarithmic scale is plotted against CE migration time (min). The mean signal intensity is represented on the z-axis of the 3D plot.

Next, proteomic data from untreated animals and animals treated with 150 or 300 mg/kg gentamicin were evaluated and a mean amplitude was calculated for each peptide at each time point (1,2,3,7,10,15,18,22,29,36 and 44 days) for both doses. The distributions of all peptides (493+557) were visually inspected on a graph with amplitude on the y-axis and treatment-time on the x-axis for controls (0 mg), 150 and 300 mg/kg gentamicin to determine regulation over time. This analysis revealed that 39 of the 493 biomarker candidates responsive to cis-platin also displayed a clear response to gentamicin (**figures S2**). Of the 557 potential candidate peptides from the gentamicin study, an additional 108 peptides showed a clear gentamicin response over the whole observation period (see **figures S2**). Combining the markers of both analyses provided a list of 147 marker candidates. Some of these peptides showed opposite regulation in the two studies. Only peptides with a change in the same direction were selected as drug induced nephrotoxicity markers (**table S2**). The mean signal intensities of the resultant 101 potential biomarkers are shown in **figure 2**.

To obtain sequence information for the nephrotoxicity marker candidates, tandem mass spectrometry was applied. We were able to identify 54 of the 101 marker candidates. **Table 1** gives sequence information for the 54 biomarker candidates. The majority of the identified biomarkers were fragments of collagen alpha-1 (I), (III) and alpha-2 (I). Fragments of apolipoprotein A-IV, fibrinogen beta, fetuin-A, actin, hemoglobin subunit beta, inter alpha-trypsin inhibitor, osteopontin, pro-epidermal growth factor, prothrombin, tropomyosin-1 alpha and contrapsin-like protease inhibitors 3 and 6 were also identified.

**Table 1:** Sequenced urinary biomarkers for drug induced kidney injury.

Mass [Da]	CE-T [Min]	Regulation factor in gentamicin study	p-value (BH)	AUC	Sequence	UniProt name (start AA- stop AA)
1197.58	36.97	12.5	2.9E−04	0.793	DSYVGDEAQSK	ACTS_RAT (53–63)
1685.83	41.55	4.7	2.1E−02	0.688	AFSPVASVESASGEVLH	FETUA_RAT (307–323)
1215.56	38.53	1234.6	1.3E−03	0.688	TIDQNLEDLR	APOA4_RAT (211–220)
2070.03	30.75	9.8	2.5E−04	0.823	AEGSpGRDGApGAKGDRGETGP	CO1A1_RAT (1009–1030)
1998.98	30.79	86.1	0.0E+00	0.998	EGSpGRDGApGAKGDRGETGP	CO1A1_RAT (1010–1030)
1366.66	39.46	16.1	1.7E−02	0.667	DRGETGPAGPpGApG	CO1A1_RAT (1024–1038)
2047.06	36.4	9.3	6.9E−04	0.798	GApGApGPVGPAGKNGDRGETGP	CO1A1_RAT (1038–1060)
1990.04	35.94	5.0	2.4E−05	0.872	ApGApGPVGPAGKNGDRGETGP	CO1A1_RAT (1039–1060)
1805.94	34.27	13.9	0.0E+00	0.959	GApGPVGPAGKNGDRGETGP	CO1A1_RAT (1041–1060)
2048.01	30.91	87.4	1.8E−03	0.710	NGDDGEAGKPGRPGERGppGP	CO1A1_RAT (218–238)
1876.95	31.11	109.9	6.4E−04	0.793	DDGEAGKPGRPGERGPpGp	CO1A1_RAT (220–238)
1646.89	27.78	10.2	1.5E−02	0.724	GEAGKPGRpGERGPpGP	CO1A1_RAT (222–238)
1662.89	27.84	7.3	3.8E−03	0.764	GEAGKpGRpGERGPpGP	CO1A1_RAT (222–238)
2040.99	36.17	100.9	0.0E+00	0.973	NSGEpGApGNKGDTGAKGEpGP	CO1A1_RAT (421–442)
1737.87	41.58	5.8	3.3E−02	0.700	TGSpGSpGPDGKTGPpGPAG	CO1A1_RAT (530–549)
1235.62	38.24	9.2	2.0E−03	0.776	pGPDGKTGPpGPAG	CO1A1_RAT (536–549)
1691.86	35.21	5.1	1.0E−05	0.898	DGKTGPpGPAGQDGRPGp	CO1A1_RAT (539–556)
1737.9	33.68	75.2	6.9E−04	0.720	GTAGEpGKAGERGVpGPpG	CO1A1_RAT (576–594)
1508.78	32.47	146.3	5.9E−05	0.785	GEpGKAGERGVpGPpG	CO1A1_RAT (579–594)
1451.77	32.23	6.0	8.2E−03	0.831	EpGKAGERGVpGPpG	CO1A1_RAT (580–594)
1550.78	40.72	76.6	4.8E−04	0.726	VGPAGKDGEAGAQGApGP	CO1A1_RAT (596–613)
1405.72	39.05	3.4	1.8E−04	0.827	GLpGPAGPpGEAGKpG	CO1A1_RAT (633–648)
1860.95	42.67	4.9	3.7E−04	0.777	TGPIGPpGPAGApGDKGETGP	CO1A1_RAT (755–775)
1584.8	33.97	19.6	6.0E−06	0.904	DGQPGAKGEpGDTGVKG	CO1A1_RAT (809–825)
1469.76	32.4	6.0	3.4E−03	0.766	GQPGAKGEpGDTGVKG	CO1A1_RAT (810–825)
1182.6	39.65	4.0	4.0E−05	0.873	DTGVKGDAGPpGP	CO1A1_RAT (820–832)
1067.56	36.83	9.4	1.5E−02	0.724	TGVKGDAGPpGP	CO1A1_RAT (821–832)
1844.97	34.95	18.4	2.0E−06	0.927	SGNAGPpGPpGPVGKEGGKGP	CO1A1_RAT (878–898)
2058.09	30.81	108.6	2.4E−04	0.758	SGNAGPpGPpGPVGKEGGKGPRG	CO1A1_RAT (878–900)
1590.87	27.67	5.8	3.0E−06	0.920	GPpGPVGKEGGKGPRGE	CO1A1_RAT (885–901)
1900.95	42.53	7.0	4.5E−02	0.655	ETGPAGRpGEVGPpGPpGPAG	CO1A1_RAT (901–921)
1714.81	42.32	119.1	2.4E−02	0.641	TGPAGRpGEVGPpGPpGPA	CO1A1_RAT (902–920)
1771.91	41.88	13.0	2.9E−03	0.756	TGPAGRpGEVGPpGPpGPAG	CO1A1_RAT (902–921)
1388.72	39.15	2.2	1.5E−05	0.866	RpGEVGPpGPpGPAG	CO1A1_RAT (907–921)
1944.02	35.43	13.6	3.0E−06	0.908	RpGEVGPpGPpGPAGEKGSPG	CO1A1_RAT (907–927)
1308.64	39.09	4.7	1.0E−02	0.707	GLpGPSGEPGKQGp	CO1A1_RAT (963–976)
1314.6	48.73	36.7	4.1E−02	0.624	GNpGPpGPpGPpGPG	CO2A1_RAT (1135–1149)
1441.74	32.22	31.9	0.0E+00	0.946	SpGIpGPKGEDGKDG	CO3A1_RAT (453–467)
1695.81	40.95	32.4	4.8E−03	0.720	GMpGSpGGPGNDGKpGPpG	CO3A1_RAT (536–554)
1351.66	39.57	6.1	6.0E−06	0.904	ApGDKGDAGPpGPQG	CO3A1_RAT (624–638)
1558.73	48.82	10.0	7.0E−06	0.892	GLpGPpGNNGNpGPpGP	CO3A1_RAT (878–894)
2071.06	36.16	4.9	4.0E−02	0.678	VGEpGPAGSKGETGNKGEpGSAG	CO1A2_RAT (351–373)
1620.84	40.7	55.9	2.5E−04	0.757	GLpGSpGNVGPAGKEGPV	CO1A2_RAT (457–474)
1677.84	41.02	46.6	3.3E−02	0.749	GLpGSpGNVGPAGKEGPVG	CO1A2_RAT (457–475)
1142.56	36.71	10.4	3.1E−03	0.751	NIGFpGPKGPSG	CO1A2_RAT (497–508)
1414.68	39.01	4.9	5.9E−03	0.753	LYQAEAFIADFK	SPA3L_RAT (156–167)
989.49	35.54	26.6	9.3E−03	0.690	IDELYLPK	SPA3N_RAT (306–313)
1690.86	41.54	13.0	2.2E−02	0.691	EPPSLRPAPPPISGGGY	FIBB_RAT (43–59)
1711.8	40.76	7.5	2.0E−02	0.759	DSFGDLSSASAImGNPK	HBB1_RAT (44–60)
1163.56	37.95	6.7	2.8E−02	0.720	LGDGLVGSRQY	O35802_RAT (651–661)
1416.68	39.34	9.0	1.1E−02	0.707	ISHELESSSSEVN	OSTP_RAT (305–317)
1583.81	33.26	119.5	2.8E−02	0.740	DGTDYKTLLSRQMG	EGF_RAT (501–514)
2082.11	31.05	44.6	4.3E−02	0.633	SLTDKTEKELLDSYIDGR	THRB_RAT (342–359)
1486.72	39.89	13.2	2.6E−02	0.684	EELDHALNDMTSI	TPM1_RABIT (272–284)

Given are molecular mass (in Da), normalized migration time (in min) regulation factor (mean signal intensity of urine samples from 150 and 300 mg/kg gentamicin treated rats collected at days 3, 7 and 10 divided by mean signal intensity of control samples), adjusted p-value (Benjamini and Hochberg), amino acid sequence (modified amino acids: p = hydroxyproline; k = hydroxylysine; m = oxidized methionine) and short protein name of the UniProt database with the position of the firs and last amino acid in parenthesis.

### Evaluation of the distribution of protein fragments of previously defined biomarkers

The U.S. FDA and European Medicines Agency (EMEA) have qualified beta-2-microglobulin, cystatin C, clusterin, kim-1, trefoil factor-3, albumin, total protein, and rpa-1 as biomarkers of acute drug-induced kidney injury in the rat [13]. Recently, researchers there described the association of clusterin, kim-1, albumin, rpa-1, osteopontin, lipocalin-2 (NGAL), alpha GST and mu GST to gentamicin treatment [12], [14]. Our rat urinary proteome database which contains naturally occurring peptides (defined by exact mass, migration time, and, if available, exact sequence), but generally not whole proteins (as these are generally not observed in urine), was searched for peptides derived from these biomarkers. We could identify 5 fragments of clusterin, 16 fragments of osteopontin and 11 fragments of albumin in the rat urine. To examine if any of these fragments are altered after treatment with gentamicin the distribution of these 32 peptides in samples of untreated rats (n = 40) were compared to samples of rats treated with 150 and 300 mg/kg gentamicin collected at days 3, 7 and 10 using correction for multiple testing. One clusterin, two albumin, and five osteopontin fragments were significantly altered (*P*<0.05, see **table 2**) and showed a direction of regulation identical to that of the total protein.

**Table 2:** Significantly changed fragments of serum albumin, osteopontin and clusterin by gentamicin induced kidney injury in rats.

Masse [Da]	CE-time [Min]	Sequence	Protein name (start AA- stop AA)	*P*-value (BH)
1180.63	31.49	DEDLTSRMKS	Osteopontin (171–180)	0.0196
1314.68	39.17	SQESDEAIKVIP	Osteopontin (180–191)	0.0002
1326.63	39.17	TVDETYVPKEF	Serum abumin (516–526)	0.0154
1416.68	39.34	ISHELESSSSEVN	Osteopontin (305–317)	0.0196
1465.73	31.50	EGALDDTRDSEMK	Clusterin (81–93)	0.0196
1753.77	43.58	DEQYPDATDEDLTSR	Osteopontin (163–177)	0.0195
2012.96	36.86	DEQYPDATDEDLTSRMK	Osteopontin (163–179)	0.0154
2406.29	22.03	EAHKSEIAHRFKDLGEQHFK	Serum abumin (25–44)	0.0283

Given are molecular mass (in Da), normalized migration time (in min), amino acid sequence (modified amino acids: p = hydroxyproline; k = hydroxylysine; m = oxidized methionine), protein name with the position of the first and last amino acid in parenthesis and p-values (adjusted according to the method of Benjamini and Hochberg).

### Generation of high-dimensional biomarker models for drug induced nephrotoxicity in the rat

As drug-induced nephrotoxicity represents a highly complex injury, we aimed to capture this complex pathophysiology in a multidimensional biomarker model. To this end, we selected two different sets of biomarker candidates for modeling purposes. First, we used all selected 101 candidate biomarkers for nephrotoxicity as listed in **table S2**. Second, we used a biomarker set composed of those 54 candidate biomarkers that were sequenced (**table 1**).

**Figure 3:**
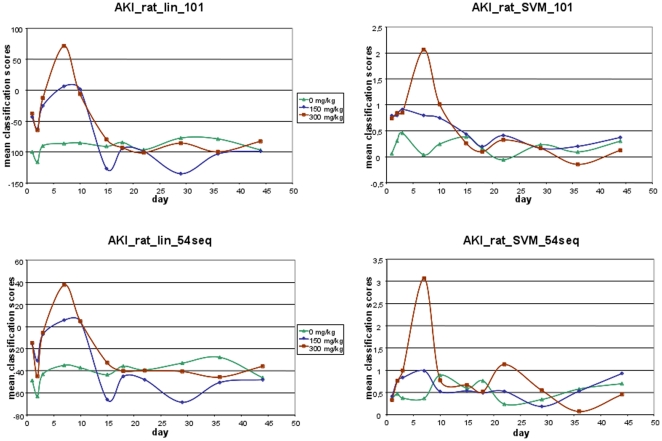
Time course of nephrotoxicity classification by the urinary models composed of gentamicin-sensitive polypeptide markers. Mean classification factors obtained with the linear (left row) and SVM (right row) model variants of the marker panels AKI_rat_lin_101 (upper panel) and AKI_rat_lin_54seq (lower panel) for urine samples from untreated animals (green curve) and animals treated with 150 (blue curve) and 300 mg/kg (red curve) gentamicin once daily for three consecutive days over time.

Linear- and support-vector-machine (SVM)-based models for the 101 and 54 marker combinations were generated using the 39 samples from the cis-platin study as a training set. The classification of the cis-platin (training) data using complete take-one-out cross-validation resulted in an AUC of 1.00 with all SVM models. Classification with the linear model based on 54 and 101 markers yielded AUC's of 0.87 and 0.95, respectively. Subsequently, samples from the gentamicin study were classified with all generated models. Classification factors for all samples from the gentamicin study are listed in **table S3** and are shown in **figure 3 A** for the linear models and **figure 3 B** for the SVM models. The classification factors represent a composite index of signal intensities of all biomarkers included in the model, calculated using linear- or SVM-based algorithms. All models showed a similar response to gentamicin treatment over time. With the exception of the SVM model with 54 peptides, the models showed higher scores immediately after the first drug dose for treated animals in comparison to untreated controls. The highest scores were observed between days 3 and 10, shortly thereafter the scores returned to the level of controls. Classification with the biomarker models correlated with the administered drug dose; such that higher doses resulted in higher classification scores.

In an effort to estimate the value and validity of the urinary peptide biomarkers, as well as the biomarker models developed here, we examined their correlation with the pathophysiological changes observed and with other kidney injury biomarkers. As evident from the data presented in **table 3**, significant correlation could be established for most variables.

**Table 3:** Correlation of urinary peptide biomarkers, as well as the biomarker models with histopathology changes and other investigated kidney injury biomarkers.

	histopathology score		necrosis score		apoptosis score		regeneration score		BUN		serum creatinine		albumin	
UniProt name(start AA- stop AA)	rho	p-value	rho	p-value	rho	p-value	rho	p-value	rho	p-value	rho	p-value	rho	p-value
CO1A1_RAT (1009–1030)	0.358	4.1E−05	0.416	1.4E−06	0.384	9.9E−06	0.199	2.6E−02	0.147	1.0E−01	0.340	1.0E−04	0.210	1.9E−02
FETUA_RAT (307–323)	0.195	2.9E−02	0.239	7.2E−03	0.250	4.9E−03	0.229	1.0E−02	0.070	4.4E−01	0.135	1.3E−01	0.250	4.9E−03
CO1A1_RAT (1039–1060)	0.398	4.4E−06	0.430	5.5E−07	0.377	1.5E−05	0.220	1.4E−02	−0.009	9.2E−01	0.346	7.8E−05	0.395	5.3E−06
CO3A1_RAT (624–638)	0.418	1.2E−06	0.468	3.9E−08	0.445	1.9E−07	0.296	7.9E−04	0.092	3.1E−01	0.310	4.3E−04	0.347	7.5E−05
CO1A1_RAT (220–238)	0.306	5.2E−04	0.309	4.6E−04	0.284	1.4E−03	0.192	3.2E−02	0.081	3.7E−01	0.362	3.3E−05	0.374	1.8E−05
CO1A1_RAT (539–556)	0.396	4.8E−06	0.432	4.8E−07	0.391	6.7E−06	0.182	4.2E−02	0.164	6.7E−02	0.306	5.1E−04	0.289	1.1E−03
CO1A1_RAT (809–825)	0.424	8.4E−07	0.461	6.3E−08	0.431	5.2E−07	0.301	6.4E−04	0.114	2.1E−01	0.307	4.9E−04	0.269	2.4E−03
EGF_RAT (501–514)	0.379	1.3E−05	0.425	7.6E−07	0.406	2.6E−06	0.272	2.1E−03	0.146	1.0E−01	0.328	1.9E−04	0.223	1.2E−02
CO1A1_RAT (1024–1038)	0.249	5.1E−03	0.254	4.2E−03	0.240	6.9E−03	0.247	5.5E−03	0.081	3.7E−01	0.284	1.3E−03	0.203	2.3E−02
HBB1_RAT (44–60)	0.188	3.6E−02	0.208	2.0E−02	0.161	7.3E−02	0.078	3.9E−01	0.126	1.6E−01	0.171	5.6E−02	0.174	5.2E−02
ACTS_RAT (53–63)	0.391	6.3E−06	0.415	1.5E−06	0.350	6.3E−05	0.278	1.7E−03	−0.040	6.6E−01	0.153	8.9E−02	0.207	2.1E−02
CO1A1_RAT (820–832)	0.362	3.3E−05	0.391	6.6E−06	0.341	1.0E−04	0.089	3.3E−01	0.118	1.9E−01	0.395	5.3E−06	0.383	1.1E−05
TPM1_RABIT (272–284)	0.293	9.0E−04	0.239	7.3E−03	0.290	1.0E−03	0.205	2.2E−02	0.193	3.1E−02	0.063	4.9E−01	0.176	5.0E−02
CO1A1_RAT (1010–1030)	0.563	8.6E−12	0.618	1.6E−14	0.633	2.4E−15	0.509	1.4E−09	0.149	9.7E−02	0.378	1.4E−05	0.255	4.0E−03
CO1A1_RAT (580–594)	0.416	1.4E−06	0.446	1.8E−07	0.463	5.3E−08	0.271	2.2E−03	0.143	1.1E−01	0.198	2.7E−02	0.208	2.0E−02
FIBB_RAT (43–59)	0.282	1.5E−03	0.335	1.3E−04	0.263	3.1E−03	0.149	9.6E−02	0.331	1.7E−04	0.241	6.8E−03	0.183	4.2E−02
CO1A1_RAT (901–921)	0.244	6.1E−03	0.260	3.4E−03	0.230	9.9E−03	0.144	1.1E−01	−0.002	9.9E−01	0.288	1.1E−03	0.288	1.1E−03
CO1A1_RAT (1038–1060)	0.316	3.4E−04	0.319	2.8E−04	0.313	3.8E−04	0.151	9.2E−02	0.052	5.6E−01	0.283	1.4E−03	0.176	5.0E−02
CO1A1_RAT (1041–1060)	0.532	1.8E−10	0.533	1.6E−10	0.529	2.3E−10	0.297	7.8E−04	0.126	1.6E−01	0.376	1.5E−05	0.381	1.2E−05
CO1A1_RAT (222–238)	0.250	4.9E−03	0.270	2.3E−03	0.285	1.3E−03	0.109	2.3E−01	0.102	2.6E−01	0.341	1.0E−04	0.280	1.6E−03
CO1A1_RAT (222–238)	0.272	2.2E−03	0.315	3.5E−04	0.298	7.5E−04	0.094	3.0E−01	0.178	4.7E−02	0.407	2.5E−06	0.309	4.6E−04
CO1A1_RAT (579–594)	0.399	4.1E−06	0.419	1.2E−06	0.408	2.3E−06	0.232	9.3E−03	0.382	1.1E−05	0.214	1.6E−02	0.246	5.7E−03
CO1A1_RAT (633–648)	0.278	1.7E−03	0.384	1.0E−05	0.346	7.6E−05	0.063	4.9E−01	0.008	9.3E−01	0.449	1.6E−07	0.462	5.9E−08
CO3A1_RAT (878–894)	0.357	4.4E−05	0.488	8.0E−09	0.479	1.6E−08	0.355	4.9E−05	0.261	3.2E−03	0.246	5.7E−03	0.148	9.9E−02
CO1A1_RAT (963–976)	0.235	8.3E−03	0.237	7.8E−03	0.205	2.2E−02	0.305	5.5E−04	0.032	7.2E−01	0.266	2.7E−03	0.207	2.1E−02
CO1A2_RAT (457–474)	0.317	3.2E−04	0.318	3.0E−04	0.330	1.7E−04	0.365	2.9E−05	0.079	3.8E−01	0.202	2.4E−02	0.236	7.9E−03
CO1A2_RAT (457–475)	0.310	4.3E−04	0.374	1.7E−05	0.362	3.3E−05	0.280	1.5E−03	0.055	5.4E−01	0.316	3.2E−04	0.238	7.5E−03
CO3A1_RAT (536–554)	0.265	2.9E−03	0.224	1.2E−02	0.313	3.9E−04	0.218	1.5E−02	−0.060	5.0E−01	0.163	6.9E−02	0.204	2.3E−02
CO2A1_RAT (1135–1149)	0.179	4.6E−02	0.195	2.9E−02	0.152	9.0E−02	0.180	4.5E−02	0.178	4.7E−02	0.229	1.0E−02	0.185	3.9E−02
CO1A1_RAT (885–901)	0.390	6.9E−06	0.455	9.5E−08	0.423	9.2E−07	0.146	1.0E−01	0.202	2.4E−02	0.439	3.0E−07	0.374	1.7E−05
CO1A1_RAT (810–825)	0.325	2.2E−04	0.381	1.2E−05	0.437	3.4E−07	0.135	1.3E−01	0.292	9.7E−04	0.254	4.3E−03	0.199	2.6E−02
CO1A1_RAT (576–594)	0.370	2.1E−05	0.389	7.3E−06	0.336	1.3E−04	0.198	2.7E−02	0.235	8.4E−03	0.316	3.2E−04	0.305	5.4E−04
SPA3N_RAT (306–313)	0.310	4.4E−04	0.352	5.7E−05	0.291	9.8E−04	0.182	4.2E−02	0.135	1.3E−01	0.333	1.5E−04	0.092	3.1E−01
OSTP_RAT (305–317)	0.273	2.1E−03	0.300	6.9E−04	0.260	3.4E−03	0.017	8.5E−01	0.275	1.9E−03	0.381	1.2E−05	0.154	8.7E−02
O35802_RAT (651–661)	0.141	1.2E−01	0.135	1.3E−01	0.105	2.4E−01	0.105	2.4E−01	−0.016	8.6E−01	0.197	2.8E−02	0.087	3.3E−01
SPA3L_RAT (156–167)	0.311	4.2E−04	0.260	3.4E−03	0.274	2.0E−03	0.179	4.5E−02	0.205	2.2E−02	0.114	2.0E−01	0.281	1.5E−03
CO1A1_RAT (218–238)	0.335	1.4E−04	0.373	1.8E−05	0.339	1.1E−04	0.363	3.1E−05	0.169	5.9E−02	0.269	2.4E−03	0.295	8.6E−04
CO1A2_RAT (497–508)	0.306	5.1E−04	0.281	1.5E−03	0.334	1.4E−04	0.244	6.1E−03	0.178	4.7E−02	0.264	3.0E−03	0.229	1.0E−02
CO1A1_RAT (421–442)	0.565	6.8E−12	0.573	3.0E−12	0.501	2.6E−09	0.326	2.1E−04	0.184	4.0E−02	0.450	1.4E−07	0.302	6.2E−04
CO1A1_RAT (536–549)	0.271	2.2E−03	0.292	9.6E−04	0.307	5.0E−04	0.253	4.4E−03	0.131	1.5E−01	0.184	4.0E−02	0.119	1.9E−01
CO1A1_RAT (907–921)	0.423	8.9E−07	0.415	1.5E−06	0.353	5.4E−05	0.249	5.1E−03	0.179	4.6E−02	0.223	1.2E−02	0.283	1.4E−03
CO1A1_RAT (907–927)	0.462	5.9E−08	0.438	3.3E−07	0.377	1.4E−05	0.246	5.6E−03	0.045	6.2E−01	0.347	7.3E−05	0.312	3.9E−04
CO1A1_RAT (878–898)	0.408	2.3E−06	0.432	4.9E−07	0.404	2.9E−06	0.253	4.4E−03	0.182	4.2E−02	0.372	1.9E−05	0.259	3.5E−03
CO1A1_RAT (878–900)	0.434	4.2E−07	0.463	5.4E−08	0.418	1.2E−06	0.246	5.7E−03	0.204	2.2E−02	0.480	1.5E−08	0.395	5.2E−06
THRB_RAT (342–359)	0.205	2.2E−02	0.258	3.6E−03	0.220	1.4E−02	0.097	2.8E−01	0.212	1.8E−02	0.301	6.4E−04	0.178	4.7E−02
CO3A1_RAT (453–467)	0.479	1.6E−08	0.496	4.0E−09	0.471	3.0E−08	0.256	3.9E−03	0.142	1.1E−01	0.413	1.7E−06	0.450	1.4E−07
CO1A1_RAT (902–920)	0.242	6.5E−03	0.291	1.0E−03	0.231	9.4E−03	0.147	1.0E−01	0.147	1.0E−01	0.146	1.0E−01	0.284	1.3E−03
CO1A1_RAT (902–921)	0.294	8.9E−04	0.288	1.1E−03	0.283	1.4E−03	0.193	3.1E−02	0.125	1.7E−01	0.304	5.6E−04	0.392	6.0E−06
CO1A1_RAT (755–775)	0.310	4.4E−04	0.388	7.8E−06	0.357	4.4E−05	0.247	5.4E−03	0.123	1.7E−01	0.447	1.7E−07	0.302	6.2E−04
CO1A1_RAT (530–549)	0.140	1.2E−01	0.201	2.5E−02	0.169	5.9E−02	−0.019	8.3E−01	−0.050	5.8E−01	0.327	2.0E−04	0.299	6.9E−04
CO1A1_RAT (821–832)	0.287	1.2E−03	0.342	9.4E−05	0.293	9.3E−04	0.091	3.1E−01	0.078	3.9E−01	0.246	5.6E−03	0.288	1.1E−03
APOA4_RAT (211–220)	0.333	1.5E−04	0.381	1.1E−05	0.370	2.1E−05	0.317	3.2E−04	0.141	1.2E−01	0.206	2.1E−02	0.220	1.4E−02
CO1A2_RAT (351–373)	0.281	1.5E−03	0.341	9.8E−05	0.327	2.0E−04	0.127	1.6E−01	0.350	6.3E−05	0.159	7.6E−02	0.135	1.3E−01
CO1A1_RAT (596–613)	0.354	5.0E−05	0.423	9.0E−07	0.377	1.5E−05	0.342	9.6E−05	0.050	5.8E−01	0.411	2.0E−06	0.296	8.2E−04
**Model**														
AKI_rat_lin_101	0.531	1.8E−10	0.577	1.8E−12	0.567	5.6E−12	0.229	1.0E−02	0.320	2.7E−04	0.489	7.2E−09	0.409	2.1E−06
AKI_rat_lin_54seq	0.488	7.8E−09	0.528	2.6E−10	0.532	1.8E−10	0.250	4.9E−03	0.241	6.8E−03	0.398	4.2E−06	0.379	1.3E−05
AKI_rat_SVM_101	0.568	5.0E−12	0.570	3.8E−12	0.492	5.6E−09	0.261	3.2E−03	0.406	2.7E−06	0.360	3.7E−05	0.385	9.0E−06
AKI_rat_SVM_54seq	0.314	3.6E−04	0.373	1.9E−05	0.366	2.7E−05	0.241	6.8E−03	0.254	4.2E−03	0.135	1.3E−01	0.111	2.2E−01

Given are short protein name of the UniProt database with the position of the firs and last amino acid in parenthesis, for the sequenced biomarkers and names of the biomarker models, rank correlation coefficients (and p-values) between histopathology, necrosis, apoptosis, regeneration scores, classical clinical chemistry endpoints (BUN, serum creatinine, albumin) and the detection level of the sequenced candidate biomarkers and the biomarker models\ classification scores.

## Discussion

Previously, urine obtained from Sprague Dawley rats before and after administration of cis-platin was analyzed to identify biomarkers of drug-induced nephrotoxicity. In a blinded sample set, a set of 34 urinary peptides was validated that demonstrated significant differences between treated and untreated animals [11]. In the present study, all data obtained previously (discovery and validation set) were used as a discovery set to define additional cis-platin-induced nephrotoxicity biomarker candidates. In addition, a set of marker candidates was defined in the urine of gentamicin treated rats. While an overlap of injury markers existed with the two nephrotoxins, it was quite modest. Mitochondrial injury leading to apoptotic and necrotic cell death is common to both nephrotoxins, however, initiating events are thought to be very different [15]–[17]. Furthermore, gentamicin is recognized to have broader nephrotoxic effects impacting the collecting ducts and glomeruli [15] while cis-platin injury is more localized to the proximal and distal tubules especially that segment (S3) of the proximal tubule located in the outer medulla and the corticomedullary junction [18]. Data from the present study further support that substantial differences exist on a molecular level between cis-platin- and gentamicin-induced injury and suggest that the toxic mechanisms of these two nephrotoxins vary considerably and/or target different structures within the nephron.

Individually, the candidate biomarkers showed a clear response to the gentamicin treatment. Biomarker models representing a composite index of all marker intensities based on both cis-platin and gentamicin specific biomarkers were generated. These models performed well in both the cis-platin and gentamicin treated cohorts. Longitudinal data from the gentamicin cohort revealed a dose-dependent response immediately after the first administration of gentamicin. The highest intensity response was observed between days 3 and 10. These results demonstrated that the gentamicin-induced changes in urinary peptides are rapid but return to control levels concurrent with injury resolution. No significant differences between the control group and the gentamicin-treated animals were observed 4 weeks after injury. Consistent with these peptide data, all the urinary protein biomarkers elevated in the original study and the associated histopathology changes [12] demonstrated the same temporal response. The correlation with the different variables was not uniform (e.g. highest correlation with the different histopathological datasets was not consistently observed with one specific biomarker), but on average the highest correlation was detected with the biomarker model based on the 101 biomarkers, again supporting the concept that a multi-marker model is better suited to display complex pathophysiological changes. Furthermore, when examining the data in a hypothesis-driven approach and limiting the dataspace to only previously described biomarkers [12], [19]–[24], we could detect fragments of peptides from three of the eight specific acute kidney injury (AKI) -associated biomarkers (albumin, osteopontin, and clusterin) from the original study. Using the untargeted MS it was not possible to detect fragments of all eight AKI-associated biomarkers that were previously described. This may be because the protein biomarkers are not represented by specific peptides (e.g. may not fragment in the same areas consistently or may degrade to very small fragments) and hence cannot be detected by an approach targeted at naturally occurring peptides. In addition, we were not able to identify the sequence of all potential peptide biomarkers in this study. This is due to the specific challenges associated with sequencing of naturally occurring peptides. These challenges are described in detail elsewhere [25]. The most prominent hurdles are poor fragmentation and/or post-translational modifications altering the mass, and thereby interfering with sequence assignment. It should also be noted that the sensitivity of untargeted MS is generally below that of targeted immunological methods. Therefore, low abundant prototypic peptides that may represent the biomarkers may be present but below the limit of detection with the MS technology applied in this study.

Altered regulation of human urinary peptides in AKI and tubular injury was previously described [26], [27]. All 54 identified sequences in this study were compared with sequenced human urinary biomarkers for AKI [26] and for Fanconi syndrome [27]. Regulation of collagen fragments in human AKI was opposite to that seen in the rat models described here. Generally, urinary collagen fragments were substantially reduced in human AKI while a significant up-regulation was observed in the rat. In addition to inherent species differences, disease etiology and severity could account for some response differences. In the gentamicin rodent study, experimental kidney injury due to a toxic insult was very apparent upon histopathology examination and was detectable with urinary protein biomarkers. However, the classical serum biomarkers, blood urea nitrogen and serum creatinine, were only minimally elevated and animals never demonstrated morbidity or clinical signs of kidney injury. In the human study, kidney injury was generally observed in conjunction with morbidity and/or more severe alteration of classical biomarkers.

Perhaps more significantly, the human study encompassed AKI of largely pre-renal or glomerular etiology while the animal models represented drug-induced, primarily kidney tubular injury. This may explain the difference in regulation of urinary collagen fragments. Reduction of glomerular function has been associated with a reduction of specific collagen fragments in the urine [28], [29]. Tubular damage resulting in reduced tubular re-absorption and consequently in an increases of the urinary protein and peptides secretion has been investigated in proteomic experiments [27]. A comparison of rat nephrotoxicity markers and human markers for tubular injury (Fanconi syndrome) [27] reveals similarities including the elevation of urinary collagen fragments. Interestingly, two collagen fragments were observed to have identical cleavage sites in the human and in the rat, indicating upregulation of similar proteases. These data give rise to the hypothesis that the observed alterations in specific urinary peptides may be indicative of disease-associated changes in extracellular remodeling, displayed in the urine by increase in the specific urinary peptides reported here.

The majority of identified biomarkers present in this study were fragments of the collagen chains alpha-1 (I), alpha-1 (III) and alpha-2 (I). Several of the collagen fragments had a PGP-motif at the C-terminus, suggesting these fragments may be generated by matrix metalloproteinase (MMP) activity. Up-regulated MMPs, especially MMP-2 and MMP-9, have been detected following acute kidney injury, mostly in animal models of ischemia [30], [31], and up-regulation of MMP-9 was reported to protect from apoptosis in AKI [32]. Deregulation of MMPs, up-regulation of MMP-2 and down-regulation of MMP-9 have also been reported in CKD [33]. In addition, MMP-9 deletion was reported to mitigate vascular lesions (hence insult) after ischemia [34], [35]. It appears reasonable that increases in urinary collagen fragments may evolve from increased MMP (and possibly other protease) activity as a result of a toxic insult. Significant up-regulation of peptides derived from the N-terminus of Fibrinogen-beta was also recognized. Recently, a peptide of similar origin was ascribed nephroprotective properties [36]. The nephroprotective capability of the Fibrinogen-beta fragment described here needs to be evaluated. For a fibrinogen alpha chain derived peptide identified in this study as a rat nephrotoxicity marker, a peptide homologue in humans exists which was previously described as prognostic for AKI development [26]. This study also identified as a potential biomarker candidate a fragment of apolipoprotein A-IV that has been described as predictive for progression of chronic kidney diseases [37].

In conclusion, we have identified a panel of urinary peptide biomarkers that are significantly associated with drug-induced nephrotoxicity in two different rat models. The biomarkers correlate with pathophysiology and likely reflect collagen degradation and changes in extracellular matrix turnover associated with increased MMP activity. These biomarkers, and especially the high-dimensional biomarker models, appear to be valuable for the monitoring of early nephrotoxicity in drug safety trials. To further establish their value and validity, we will aim at analyzing their performance in additional studies, and also investigate their distribution in rat models for kidney disease like the ZDF model.

## Methods

All animal procedures were performed in accordance with the U. S. Public Health Service Guide for the Care and Use of Laboratory Animals through an animal use protocol (WO-2006-52) approved by the White Oak Animal Program Institutional Animal Care and Use Committee in an AAALAC-accredited facility on the White Oak Campus of the U. S. FDA, Silver Spring, MD.

### Specimen characteristics

Urine samples from rats in a previously described cis-platin study [11] were analyzed. Rats were given a single i.p. injection of cis-platin (3 or 6 mg/kg). Urine samples were collected at 0, 24, 48 and 72 hrs after dosing. Details on this sample cohort are given in **table S1 sheet 1**. In addition, urine samples were obtained from rats following gentamicin administration [12]. Saline treated controls were included with animals receiving intramuscular injection of 150 or 300 mg/kg gentamicin once a day for up to 3 consecutive days. Urine samples were collected 24 hours following a single dose (day 1), two consecutive daily doses (day 2), or three consecutive daily doses (day 3). Subsequently, urine samples were collected following a recovery time (no additional treatments) at 7,10,15,18,22,29,36 or 44 days following the first of the three consecutive daily gentamicin doses. For each dose group, 3–4 samples were analyzed at each time point. Three control animals from which no samples were obtained were tested as sentinels. One control and one treated animal were removed from the study due to non-treatment related complications. **Table S1 sheet 2** includes all samples, doses and collection days for the gentamicin study.

### CE-MS analysis

150 µl urine were mixed with 150 µl of 2 M urea, 100 mM NaCl, 10 mM NH_4_OH containing 0.02% SDS. Samples were ultrafiltered using a Centristat 20 kDa cut-off centrifugal filter (Satorius, Göttingen, Germany) to eliminate high molecular weight compounds. The filtrate was desalted using a NAP-5 gel filtration column (GE Healthcare Bio Sciences, Uppsala, Sweden) to remove urea and electrolytes. The sample was lyophilized in a Christ Speed-Vac RVC 2-18/Alpha 1-2 (Christ, Germany) and stored at 4°C until use. Shortly before CE-MS analysis, the samples were re-suspended in 10 µL HPLC grade H_2_O.

CE-MS analysis was performed using a P/ACE MDQ capillary electrophoresis system (Beckman Coulter, Fullerton, USA) on-line coupled to a MicrOTOF MS (Bruker Daltonic, Bremen, Germany). The ESI-sprayer (Agilent Technologies, Palo Alto, CA, USA) was grounded, and the ion spray interface potential was set −4.5 kV. Data acquisition and MS acquisition methods were automatically controlled by the CE via contact-close-relays. Spectra were accumulated every 3 s, over a range of m/z 350 to 3000. The analytical characteristics of the CE-MS system were extensively investigated by Theodorescu et al. [38] and Kolch et al. [39].

### Data processing

Mass spectral ion peaks representing identical molecules at different charge states were deconvoluted into single masses using MosaiquesVisu software [40]. We defined “rat urinary housekeeping polypeptides” and calibrated the CE-MS data utilizing 545 migration time data points, 108 mass data points by applying local and global linear regression, respectively. References of 37 highly abundant peptides were used as “internal standard peptides” for ion signal intensity (amplitude) calibration using global linear regression. The procedure to use “internal standard” for amplitude normalization, was shown to be a reliable method to address both analytical and dilution variances [41]. The resulting peak list characterizes each polypeptide by its molecular mass [Da], normalized migration time [min] and signal intensity. All detected peptides were deposited, matched, and annotated in a Microsoft SQL database, allowing further analysis and comparison of multiple samples. Polypeptides within different samples were considered identical if the mass deviation was lower than ±50 ppm for masses <4.000 Da, for masses between 4.000 and 6.000 Da gradually increasing to ±150 ppm, and 150 ppm for features >6 kDa. Acceptable migration time deviation was <±1 minutes for 19 min, gradually increasing to <±2.5 min at 50 min.

### Statistical analysis

The reported *P*-values were calculated using the natural logarithm transformed intensities and the Wilcoxon test. Only peptides that were found at frequencies >30% in either case or control group were examined. The false discovery rate (FDR) adjustments of Banjamini-Hochberg [42] were employed to correct for multiple testing. Receiver operator characteristic (ROC) curves have been constructed and the area under the ROC curve (AUC) has been calculated using MedCalc version 8.1.1.0 (MedCalc Software, Belgium, www.medcalc.be).

### Sequencing

Urine samples were analysed on a Dionex Ultimate 3000 RSLS nano flow system (Dionex, Camberly UK). After loading (5 µl) onto a Dionex 0.1×20 mm 5 µm C18 nano trap column at a flowrate of 5 µl/min in 0.1% formic acid and 2% acetonitrile, sample was eluted onto an Acclaim PepMap C18 nano column 75 µm×15 cm, 2 µm 100 Å at a flow rate of 0.3 µl/min. The trap and nano flow column were maintained at 35°C. The samples were eluted with a gradient of solvent A: 0.1% formic acid verses solvent B: 80% acetonitrile starting at 5% B rising to 50% B over 100 min.

The eluant was ionized using a Proxeon nano spray ESI source (Thermo Fisher Hemel UK) operating in positive ion mode into an Orbitrap Velos FTMS. Ionization voltage was 2.5 kV and the capillary temperature was 200°C. The mass spectrometer was operated in MS/MS mode scanning from 380 to 2000 amu. The top 10 multiply charged ions were selected from each scan for MS/MS analysis using HCD at 35% collision energy. The resolution of ions in MS1 was 60,000 and 7,500 for HCD MS2. Data files were searched against the IPI rat non-redundant database using the Open Mass Spectrometry Search Algorithm (OMSSA, http://pubchem.ncbi.nlm.nih.gov/omssa) and SEQUEST (by using Thermo Proteome Discoverer), without any enzyme specificity. No fixed modification and oxidation of methionine and proline as variable modifications were selected. Mass error window of 10 ppm and 0.05 Da were allowed for MS and MS/MS, respectively. Peptide data were extracted using high peptide confidence and top one peptide rank filters. The OMSSA results were further optimized using COMPASS [43], 1% FDR was used as a cut-off value. The correlation between peptide charge at the working pH of 2 and CE-migration time was utilized to minimize false-positive identification rates [44]: Calculated CE-migration time based on the number of basic amino acids was compared to the experimental migration time. Accepted were only those peptides which were found with both search algorithms (OMSSA and SEQUEST) and having a mass deviation below ±50 ppm and a CE-migration time deviation below ±2 min.

### Establishment of biomarker-based classifiers

For generation of disease-specific polypeptide patterns two different algorithms were used: Support vector machine (SVM)-based MosaCluster software [45] and a linear combination of log-transformed, normalized data, as described [46], [47]. MosaCluster (version 1.7.0) was developed for discrimination between different patient groups in the high-dimensional parameter space by using SVM learning. It generates high dimensional models, which rely on features (biomarkers) displaying statistically significant differences between data from patients with a specific disease to controls or other diseases. Each feature allegorizes one dimension in the n-dimensional parameter space [48]–[51].

For linear combination, normalized signal intensity values below 1 were substituted with a value of 1 to avoid negative values by log-transformation. The average signal intensity for a specific biomarker over all cases was compared to the average intensity for the biomarker over all controls. To avoid artificial weighting of specific biomarkers in the set due to the difference in observed signal intensities for case and control, the relative distance between the two averages (case and control) was set to a value of 2. This relative distance of signal intensities between the disease and control samples was provided using the formula:
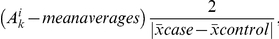


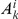
 is the log-transformed signal intensity of the i^th^ biomarker in the k^th^ sample in either set, mean_averages_ is the average of the mean intensity of all possible markers for test set samples, 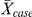 represents the mean observed signal intensity of the possible biomarker from all 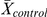 cases samples and represents the mean signal intensity of the possible biomarker from the combined control samples.

## Supporting Information

Figure S1 **Capillary electrophoresis coupled to mass spectrometry profiling of rat urine.** The compiled data sets of urine samples from gentamicin-treated rats at different doses and sampling days are shown. Molecular mass of the analyzed polypeptides (kDa) in logarithmic scale is plotted against CE migration time (min). The mean signal intensity is represented in arbitrary units on the z-axis of the 3D plot.
(PDF)

Figure S2 **Time course of mean signal intensities of all defined (n = 147) nephrotoxicity peptide maker candidates.** Mean signal intensities of the respective peptide in urine samples from untreated animals and animals treated once daily for three consecutive days with 150 and 300 mg/kg gentamicin over time are shown. The first 39 diagrams depict the selected cis-platin markers and the subsequent figures the additional 108 gentamicin markers.
(PDF)

Table S1 **Characteristics of sample cohorts.** **(A) cis-Platin cohort.** The animal ID, evaluation ID, the cis-platin dosage, time of treatment (* urine sample collected before treatment) and the group (usage as), are given. **(B) gentamicin cohort.** The animal ID, evaluation ID, the gentamicin dosage, time of treatment and group usage, are given.
(XLS)

Table S2 **Characteristics of the 101 cis-platin- and/or gentamicin-specific polypeptides.** Shown are the peptide identification number in the dataset (Peptid ID), molecular mass (in Da) and normalized migration time (in min). Given are the p-values (adjusted according to Benjamini-Hochberg), AUC-values and the regulation factor for the case group compared to the control group for gentamicin and for cis-platin. In addition, amino acid sequence (modified amino acids: p = hydroxyproline; k = hydroxylysine; m = oxidized methionine), parent protein name with the position of the first (start) and last (stop) amino acid, and Swiss-Prot entry numbers are given.
(XLS)

Table S3 **Classification scores of gentamicin-treated rats as determined by different biomarker models.**
(XLS)
