# Supplementary material for: Proteomic Candidate Biomarkers of Drug-Induced Nephrotoxicity in the Rat
Source: PLoS One. 2012 Apr 11;7(4):e34606. doi: 10.1371/journal.pone.0034606 (PMC3324487; doi:10.1371/journal.pone.0034606)
Supplement: Figure S1 — Capillary electrophoresis coupled to mass spectrometry profiling of rat urine. The compiled data sets of urine samples from gentamicin-treated rats at different doses and sampling days are shown. Molecular mass of the analyzed polypeptides (kDa) in logarithmic scale is plotted against CE migration time (min). The mean signal intensity is represented in arbitrary units on the z-axis of the 3D plot. (PDF) [file pone.0034606.s001.pdf]

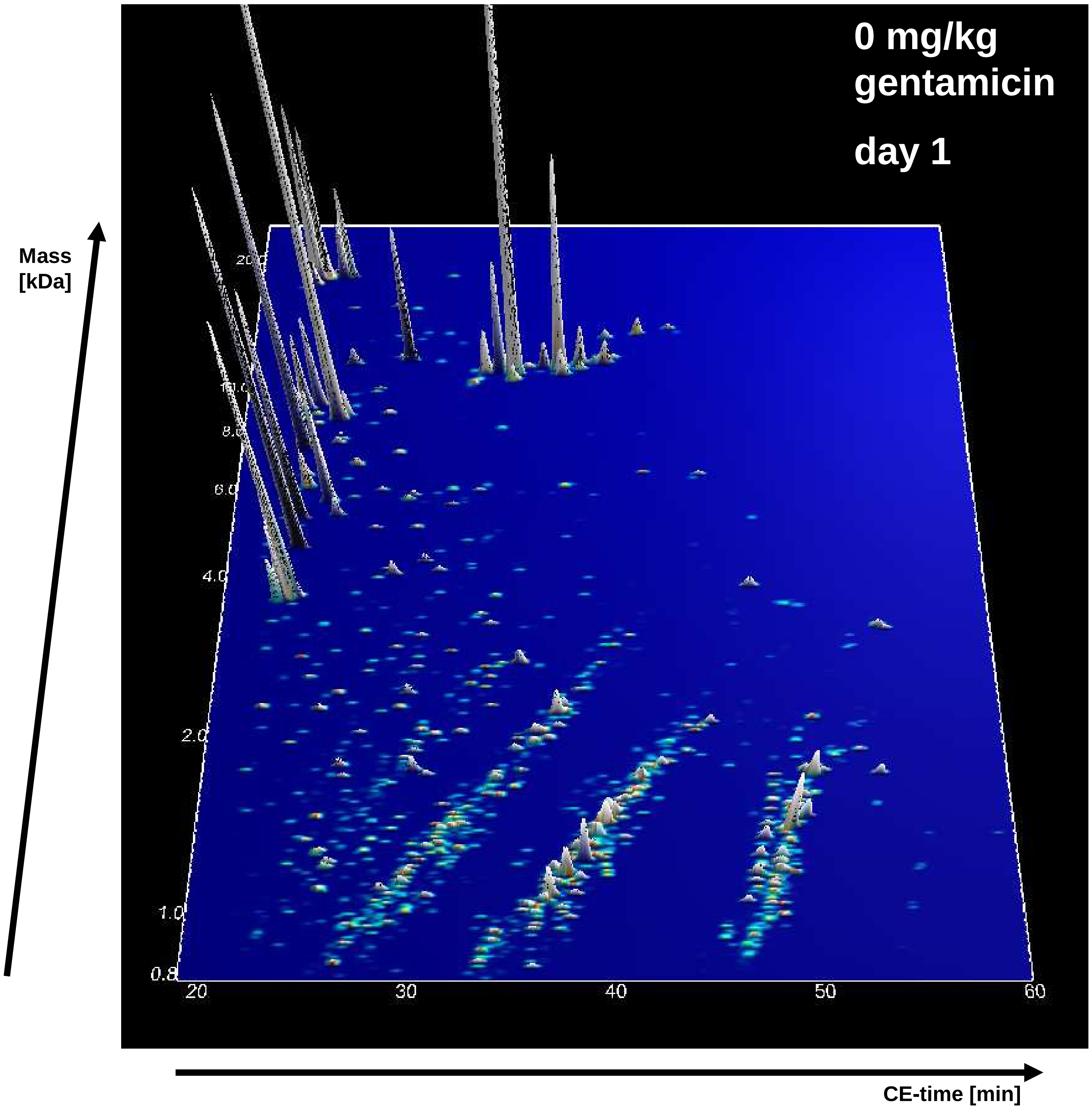
0 mg/kg
gentamicin
day 1
Mass
[kDa]
CE-time [min]
0.8
1.0
2.0
4.0
6.0
8.0
20
30
40
50
60

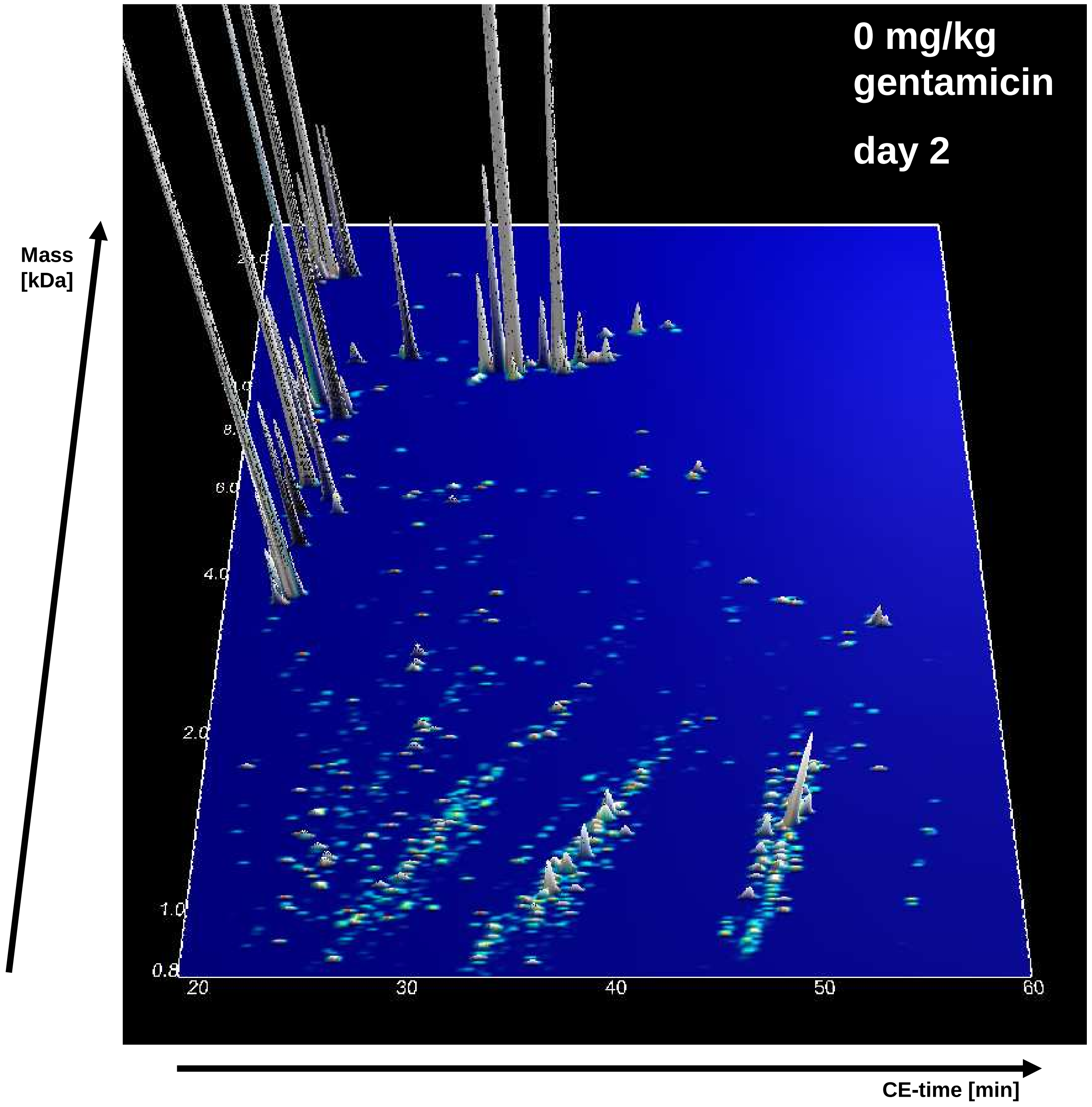

0 mg/kg gentamicin
day 2
Mass [kDa]
CE-time [min]
20
30
40
50
60
0.8
1.0
2.0
4.0
6.0

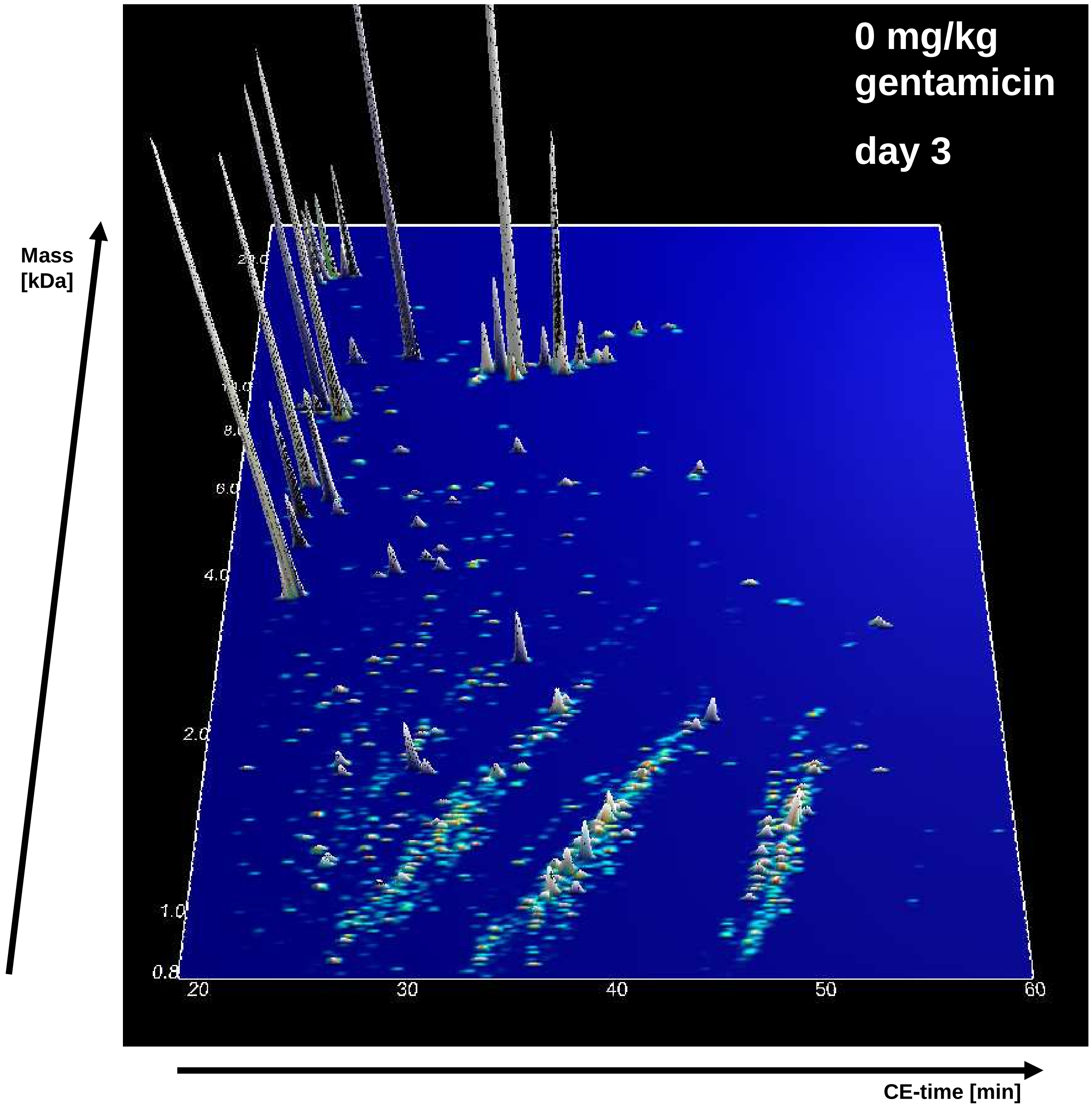
0 mg/kg
gentamicin
day 3
Mass
[kDa]
CE-time [min]
20
30
40
50
60
0.8
1.0
2.0
4.0
6.0
8.0

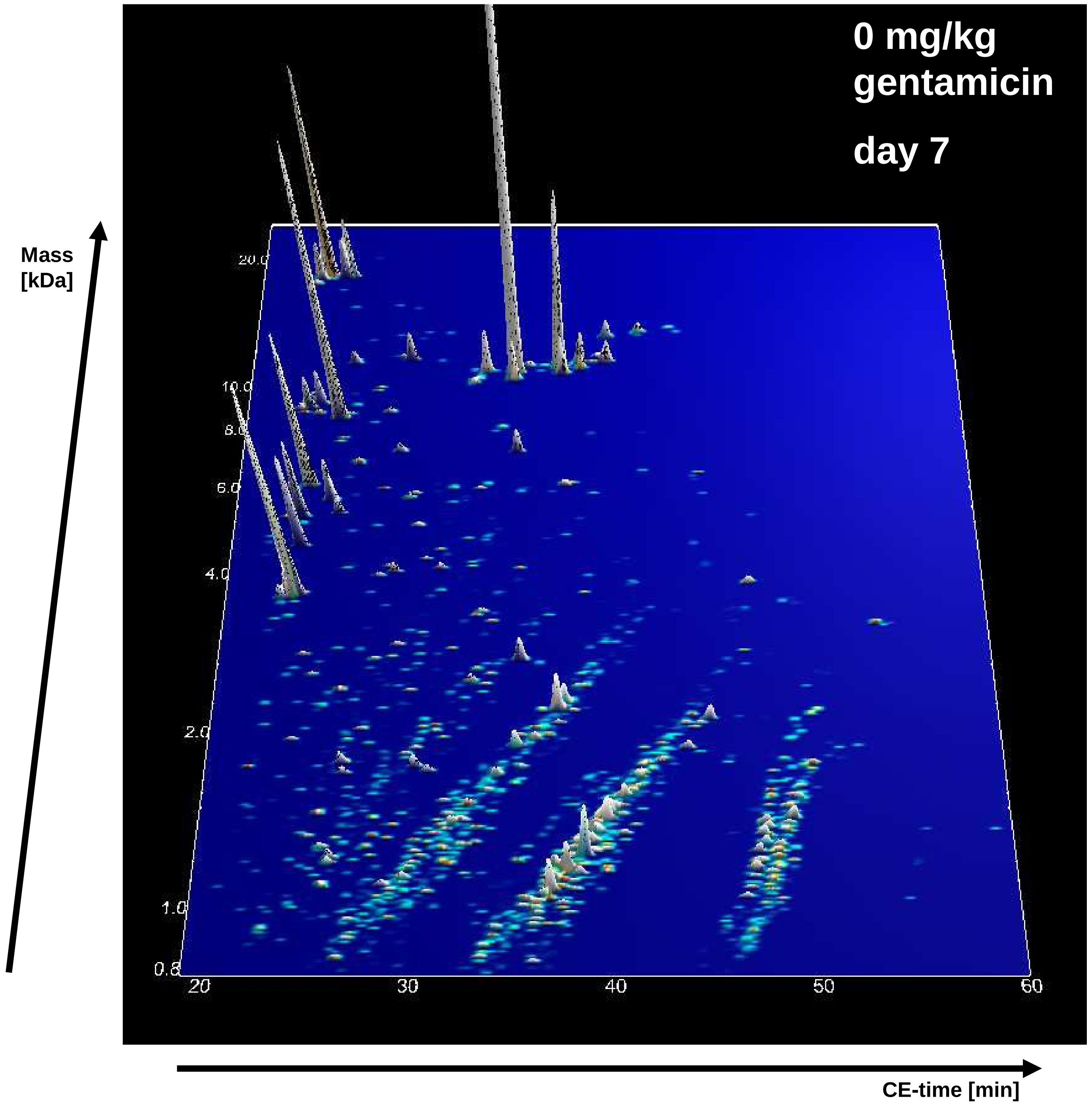
0 mg/kg
gentamicin
day 7
Mass
[kDa]
20.0
10.0
8.0
6.0
4.0
2.0
1.0
0.8
20
30
40
50
60
CE-time [min]

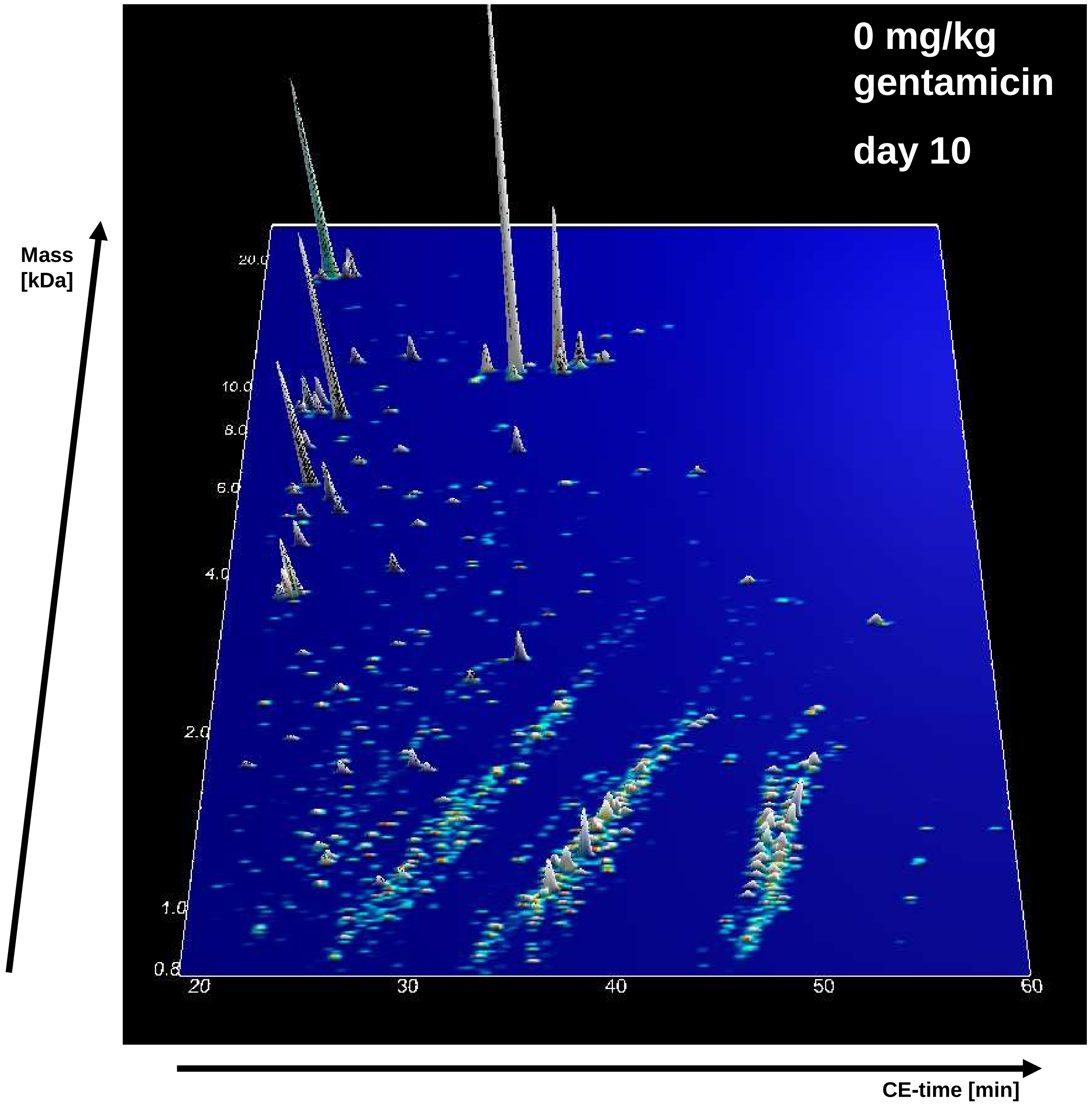

0 mg/kg
gentamicin
day 10
Mass
[kDa]
20.0
10.0
8.0
6.0
4.0
2.0
1.0
0.8
20
30
40
50
60
CE-time [min]

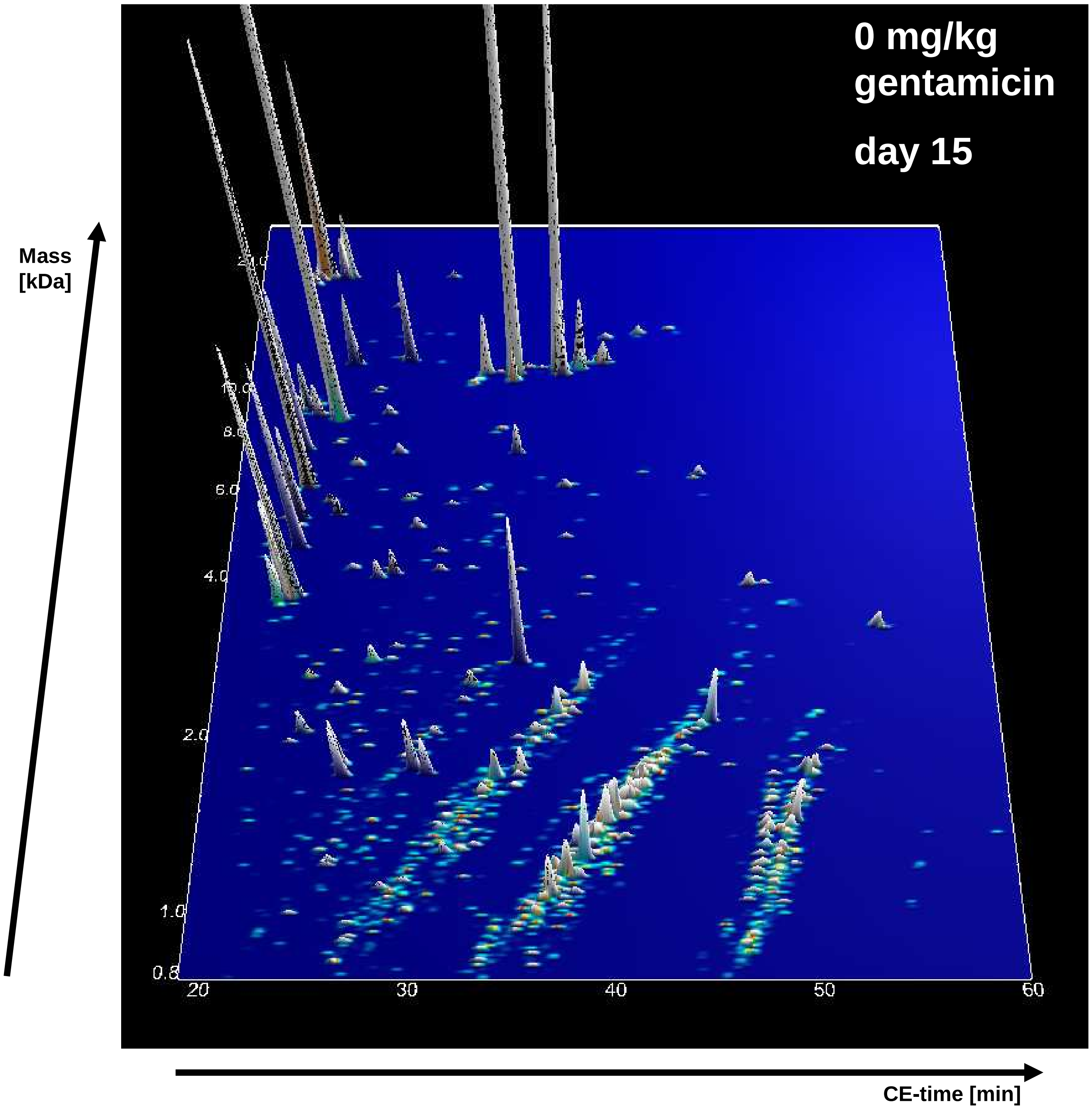

0 mg/kg
gentamicin
day 15
Mass
[kDa]
20.0
10.0
8.0
6.0
4.0
2.0
1.0
0.8
20
30
40
50
60
CE-time [min]

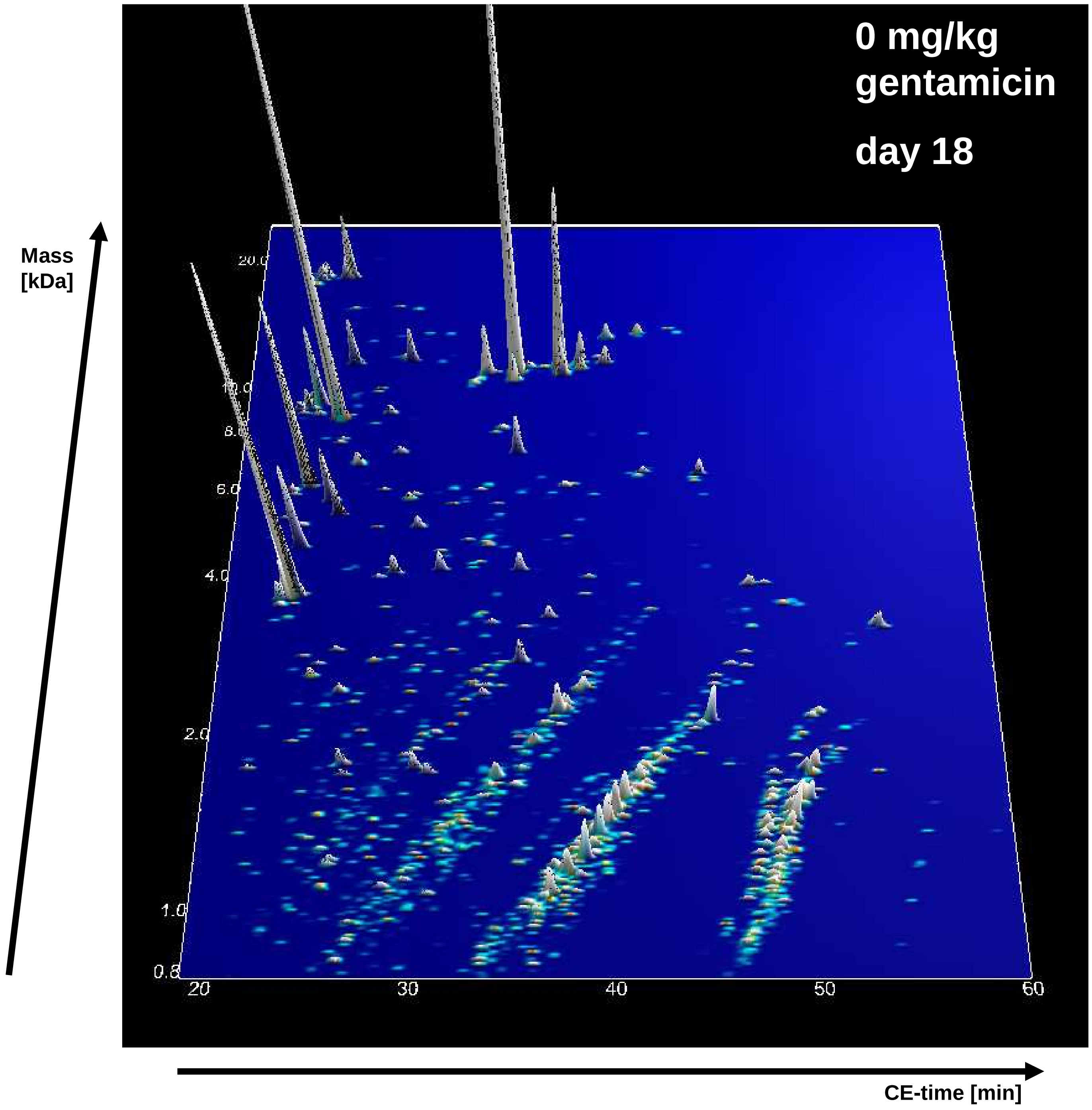

0 mg/kg
gentamicin
day 18
Mass
[kDa]
20.0
10.0
8.0
6.0
4.0
2.0
1.0
0.8
20
30
40
50
60
CE-time [min]

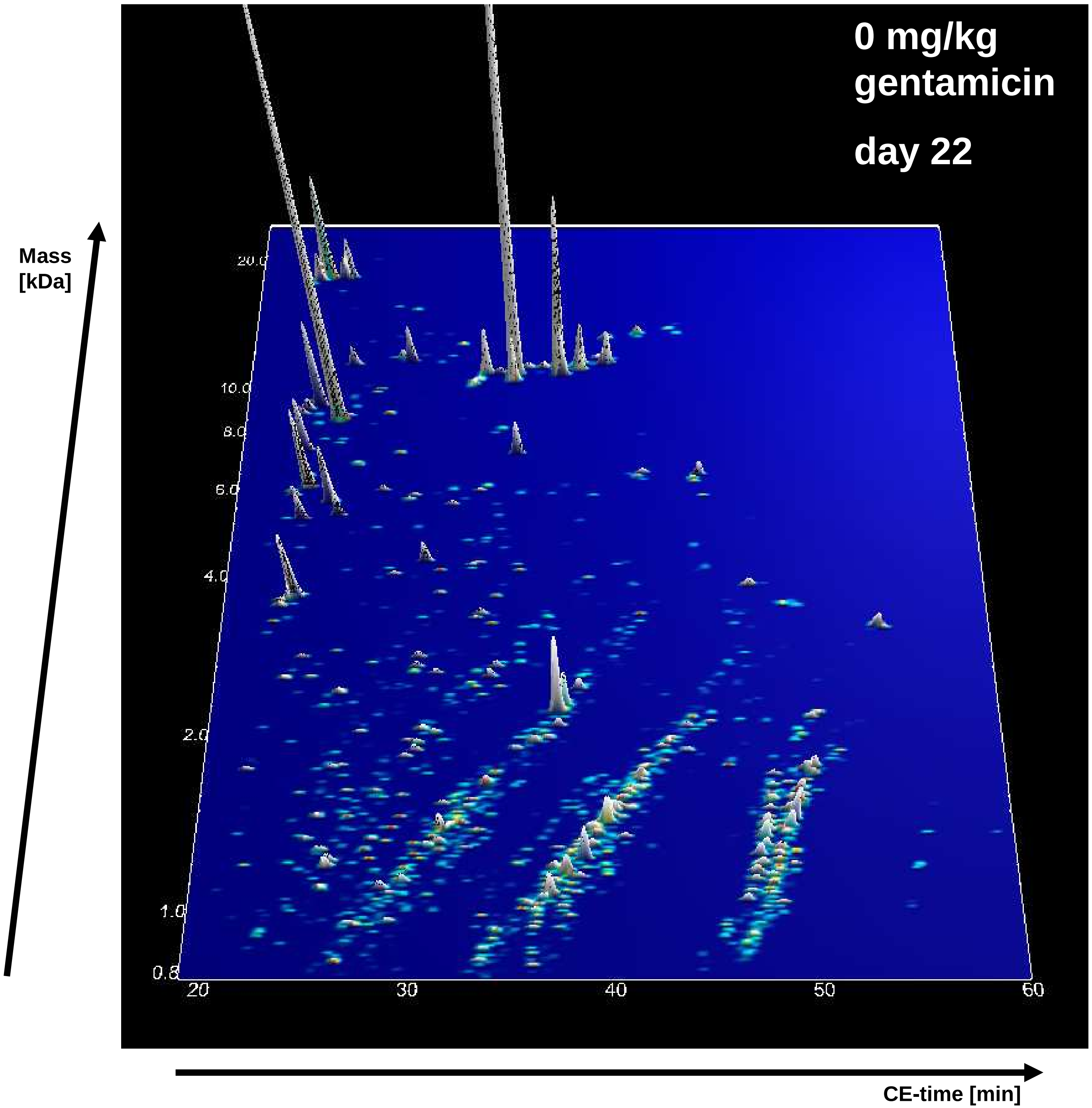

0 mg/kg
gentamicin
day 22
Mass
[kDa]
20.0
10.0
8.0
6.0
4.0
2.0
1.0
0.8
20
30
40
50
60
CE-time [min]

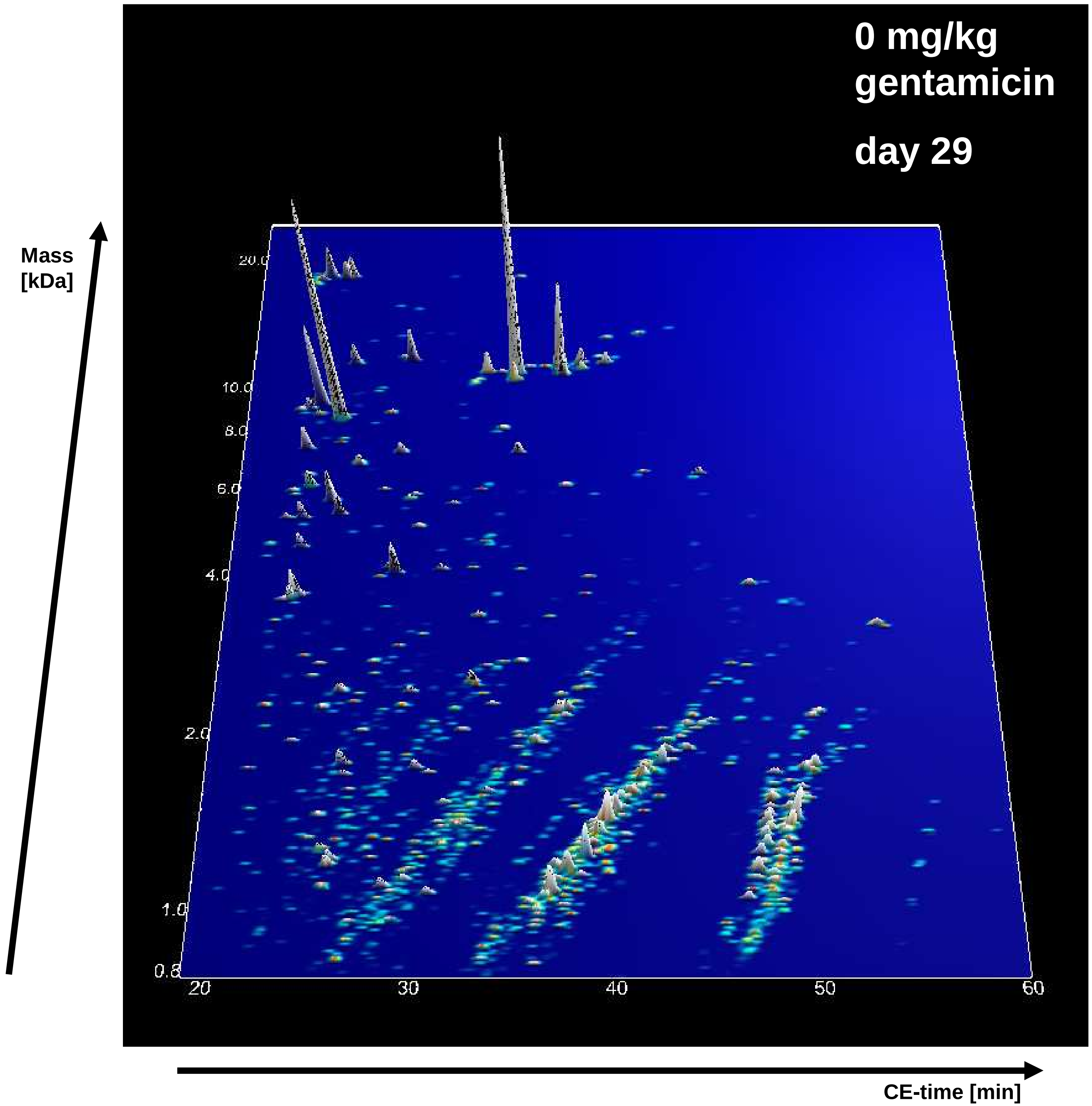
0 mg/kg
gentamicin
day 29
Mass
[kDa]
20.0
10.0
8.0
6.0
4.0
2.0
1.0
0.8
20
30
40
50
60
CE-time [min]

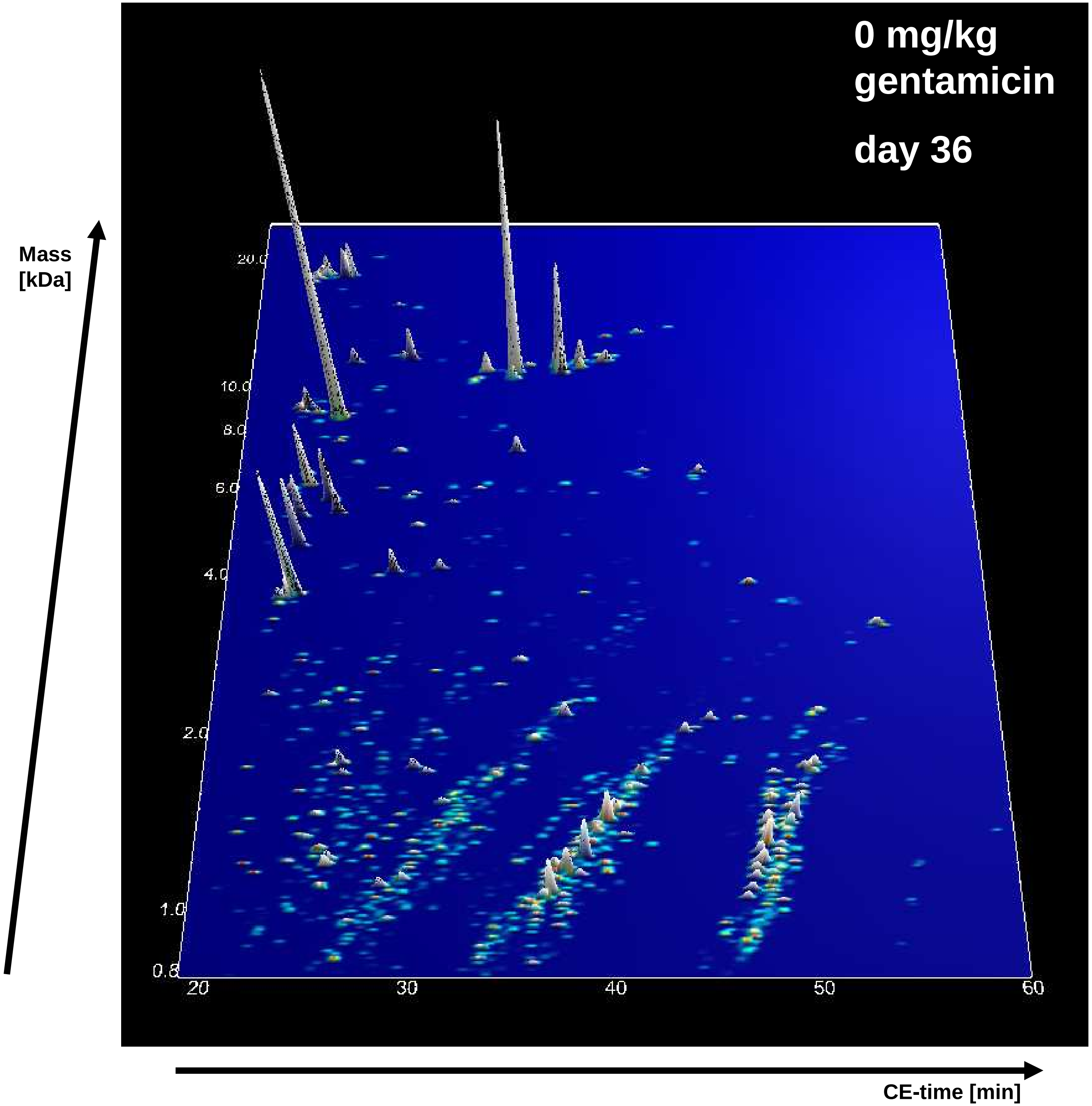

0 mg/kg gentamicin
day 36
Mass [kDa]
20.0
10.0
8.0
6.0
4.0
2.0
1.0
0.8
20
30
40
50
60
CE-time [min]

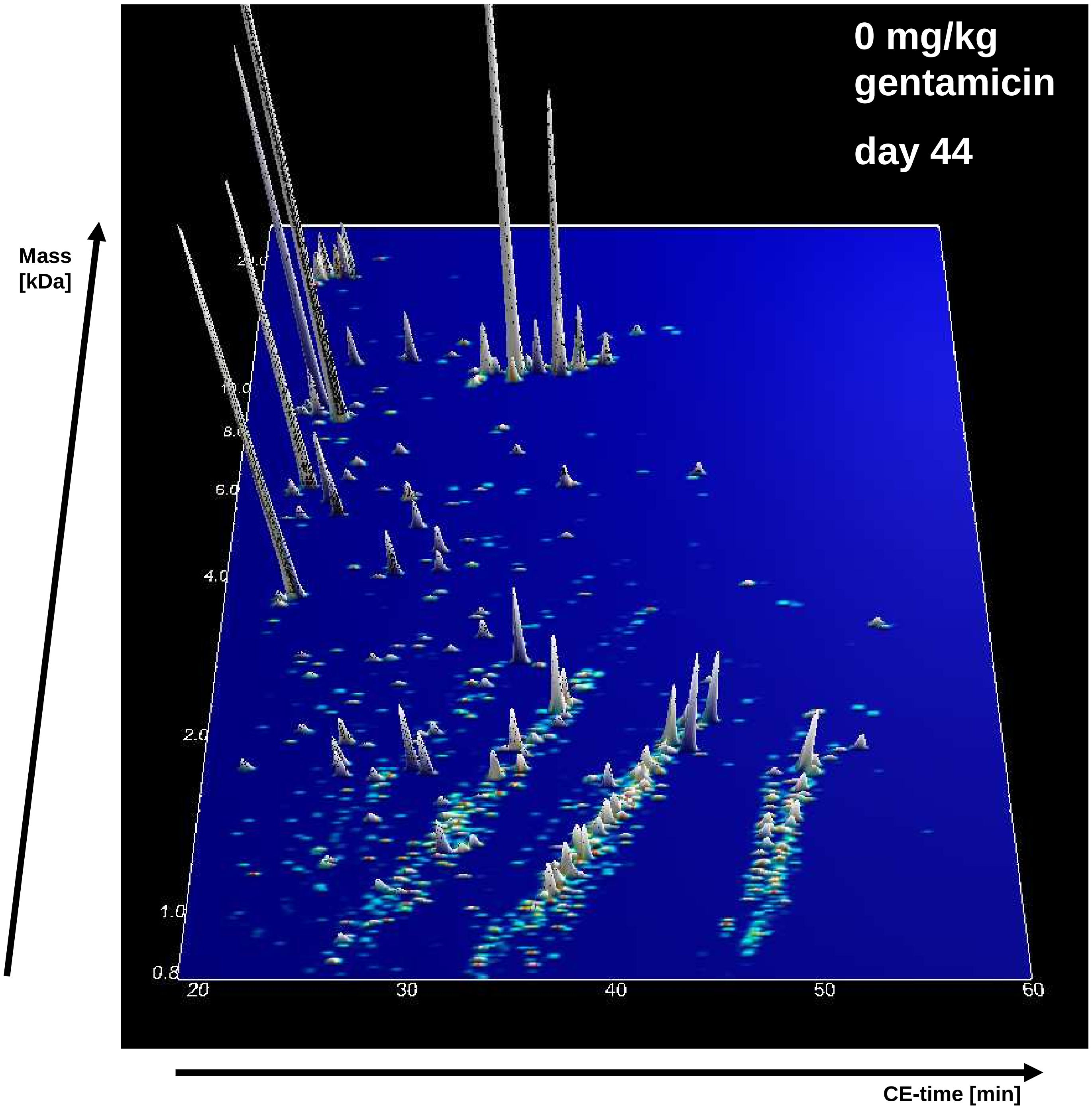
0 mg/kg
gentamicin
day 44
Mass
[kDa]
20.0
10.0
8.0
6.0
4.0
2.0
1.0
0.8
20
30
40
50
60
CE-time [min]

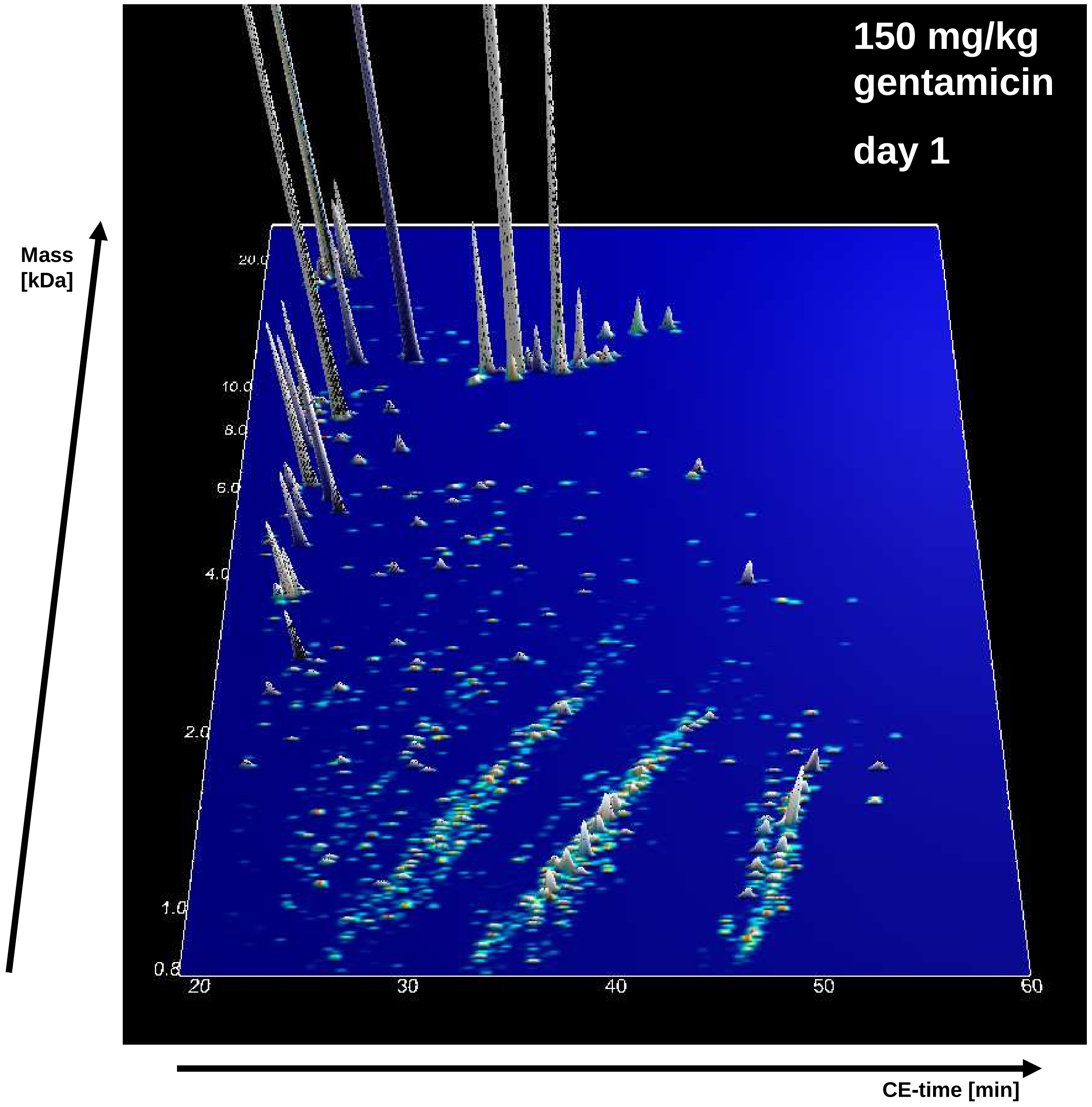
150 mg/kg
gentamicin
day 1
Mass
[kDa]
20.0
10.0
8.0
6.0
4.0
2.0
1.0
0.8
20
30
40
50
60
CE-time [min]

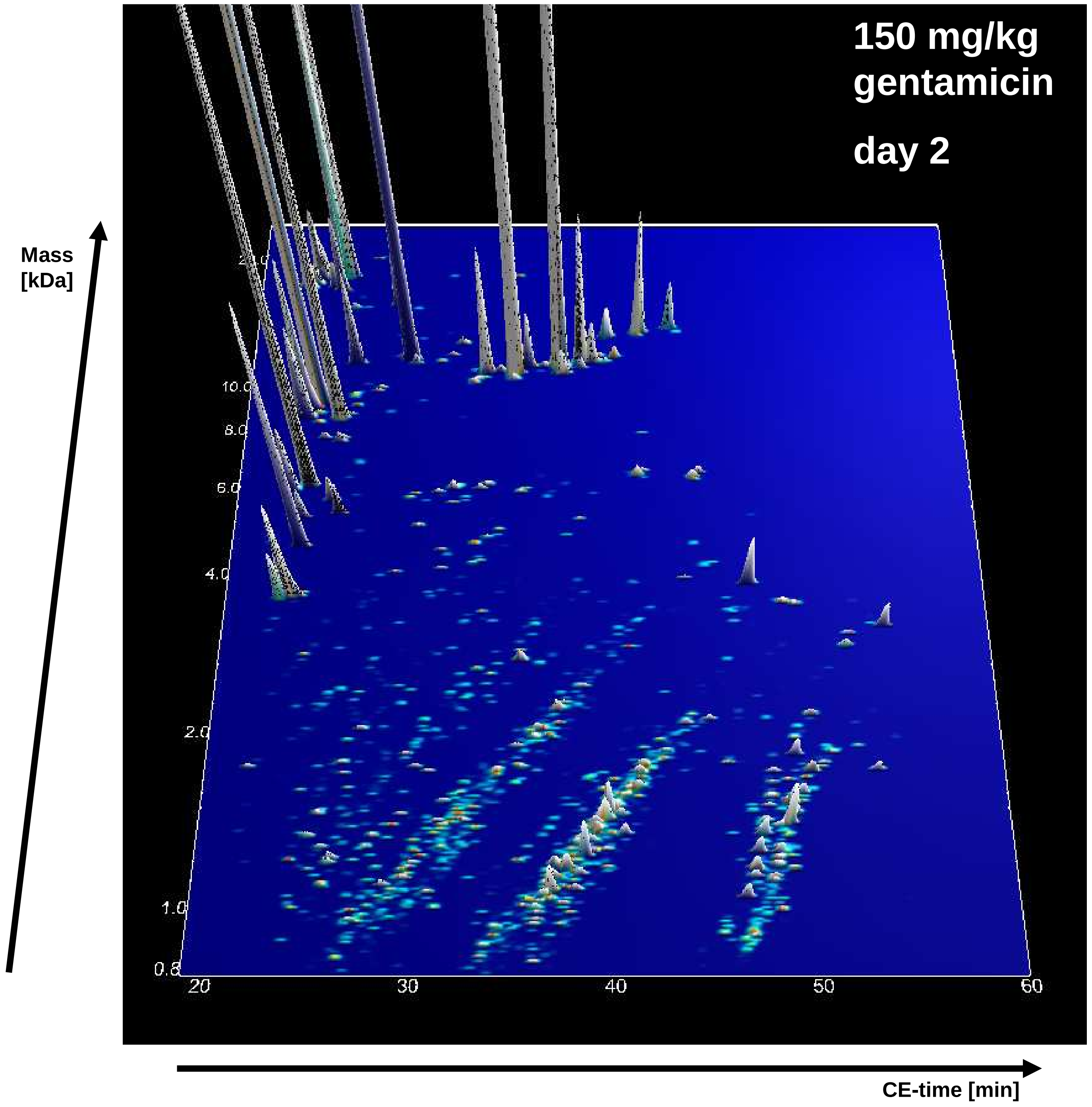
150 mg/kg
gentamicin
day 2
Mass
[kDa]
20.0
10.0
8.0
6.0
4.0
2.0
1.0
0.8
20
30
40
50
60
CE-time [min]

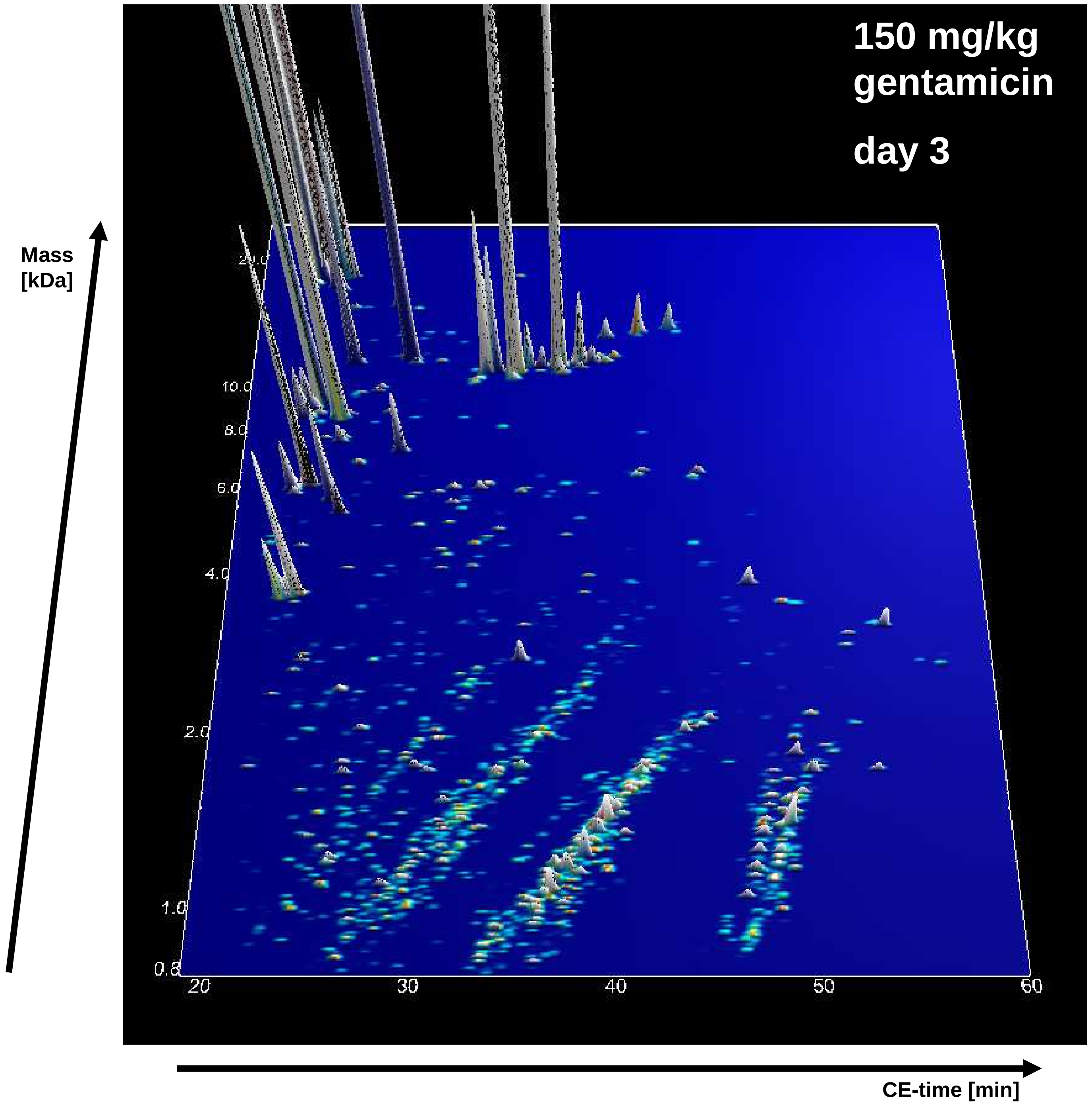
150 mg/kg
gentamicin
day 3
Mass
[kDa]
20.0
10.0
8.0
6.0
4.0
2.0
1.0
0.8
20
30
40
50
60
CE-time [min]

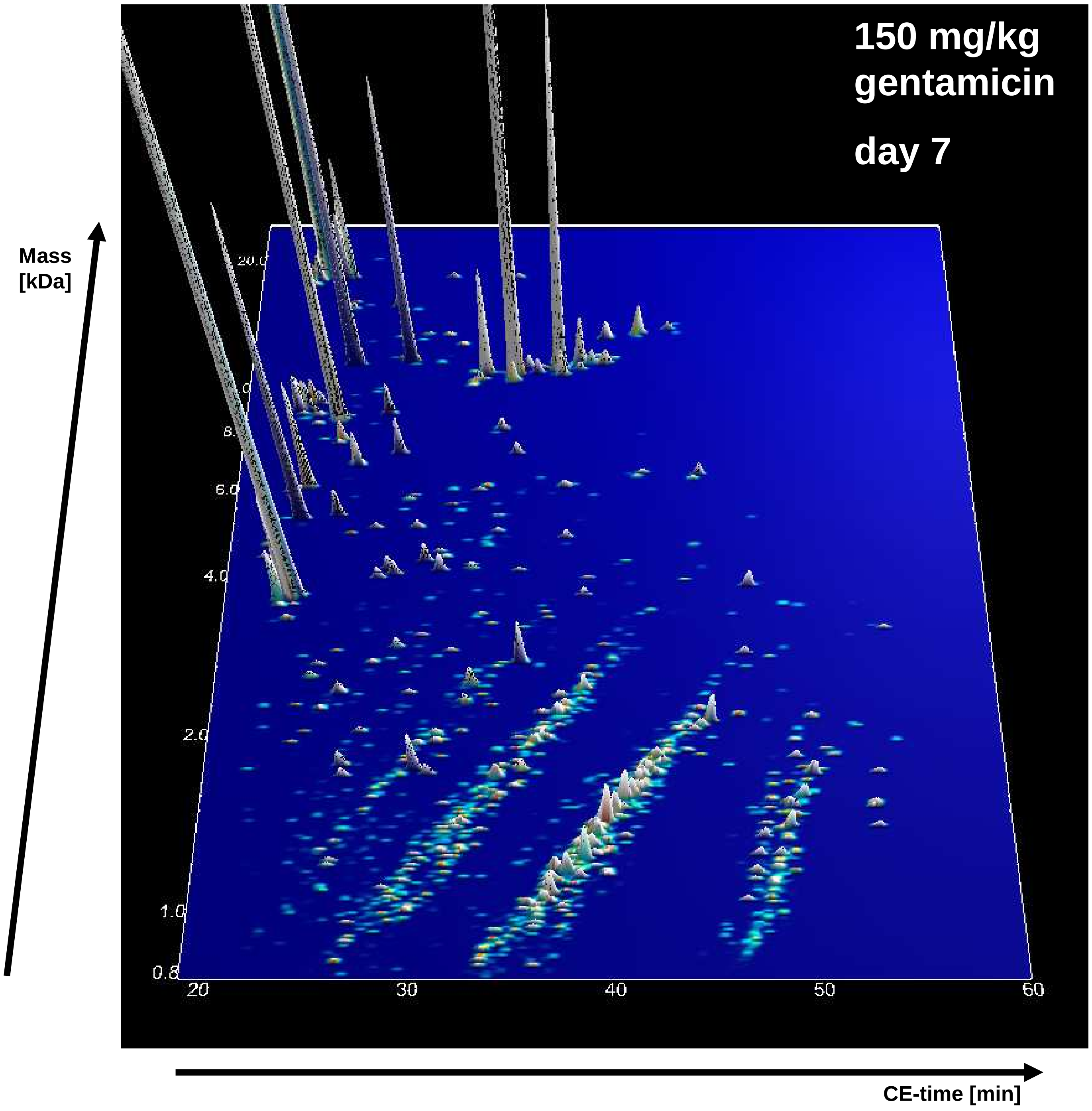
150 mg/kg
gentamicin
day 7
Mass
[kDa]
20.0
6.0
4.0
2.0
1.0
0.8
20
30
40
50
60
CE-time [min]

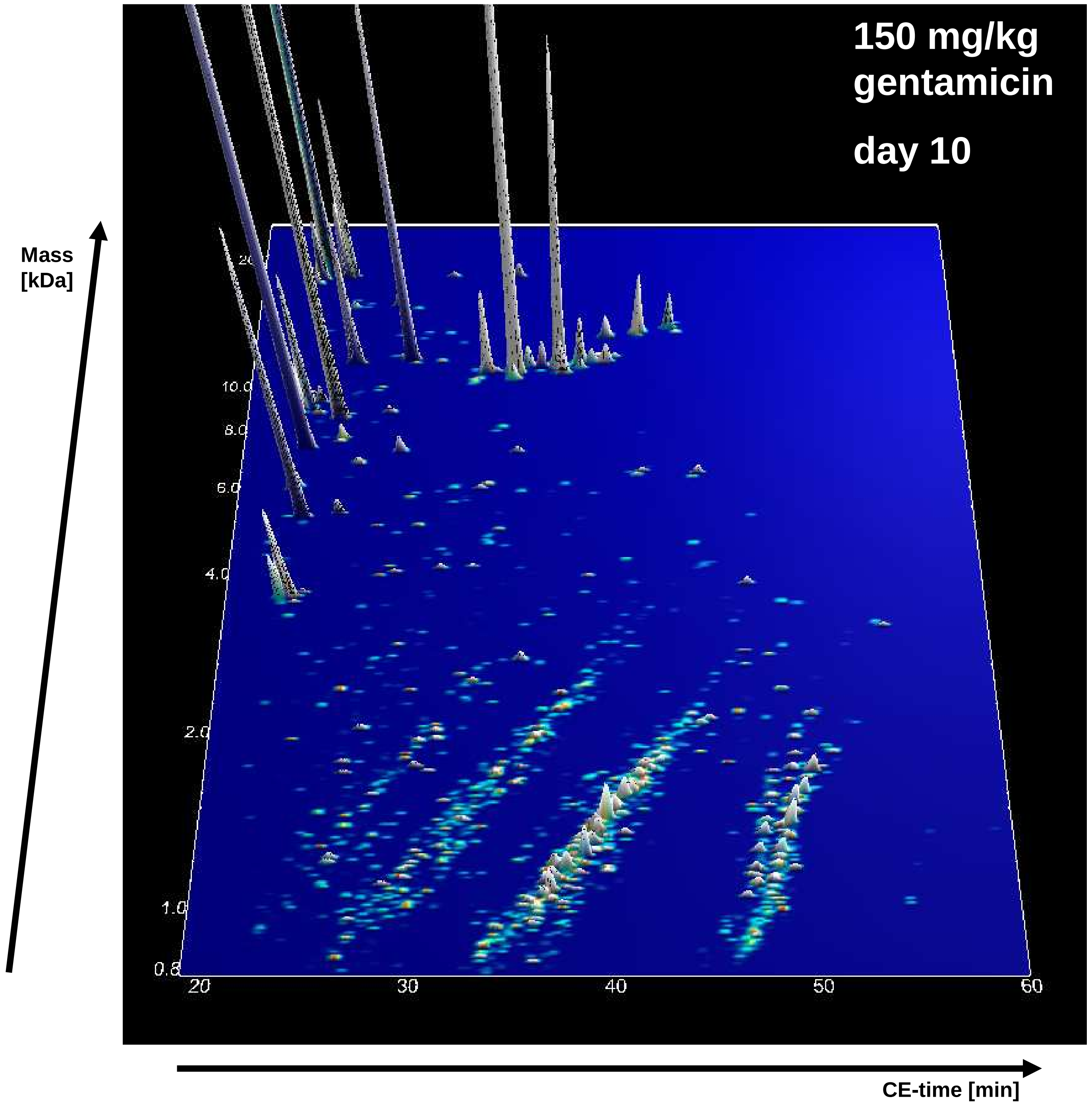
150 mg/kg
gentamicin
day 10
Mass
[kDa]
10.0
8.0
6.0
4.0
2.0
1.0
0.8
20
30
40
50
60
CE-time [min]

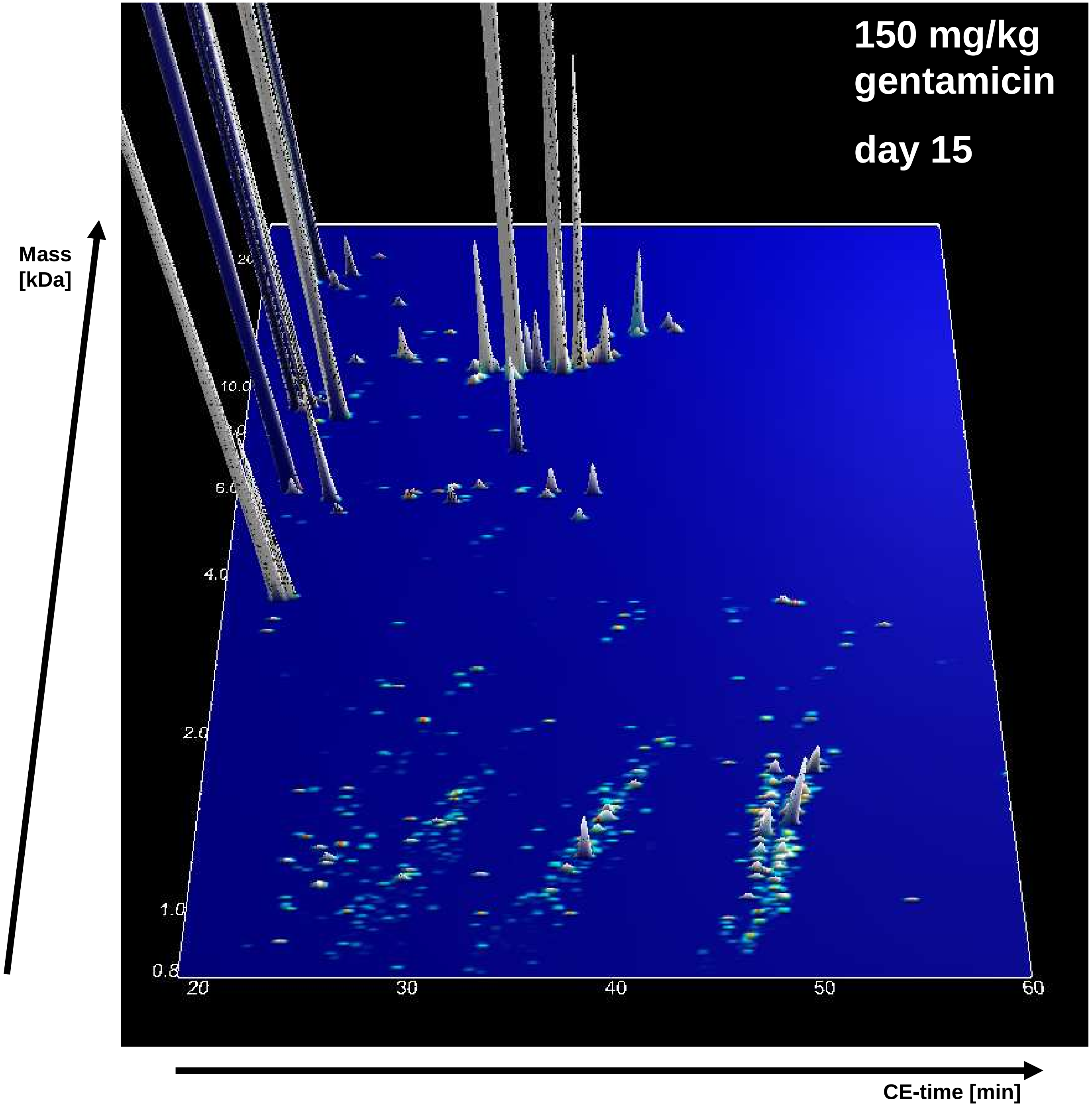
150 mg/kg
gentamicin
day 15
Mass
[kDa]
10.0
6.0
4.0
2.0
1.0
0.8
20
30
40
50
60
CE-time [min]

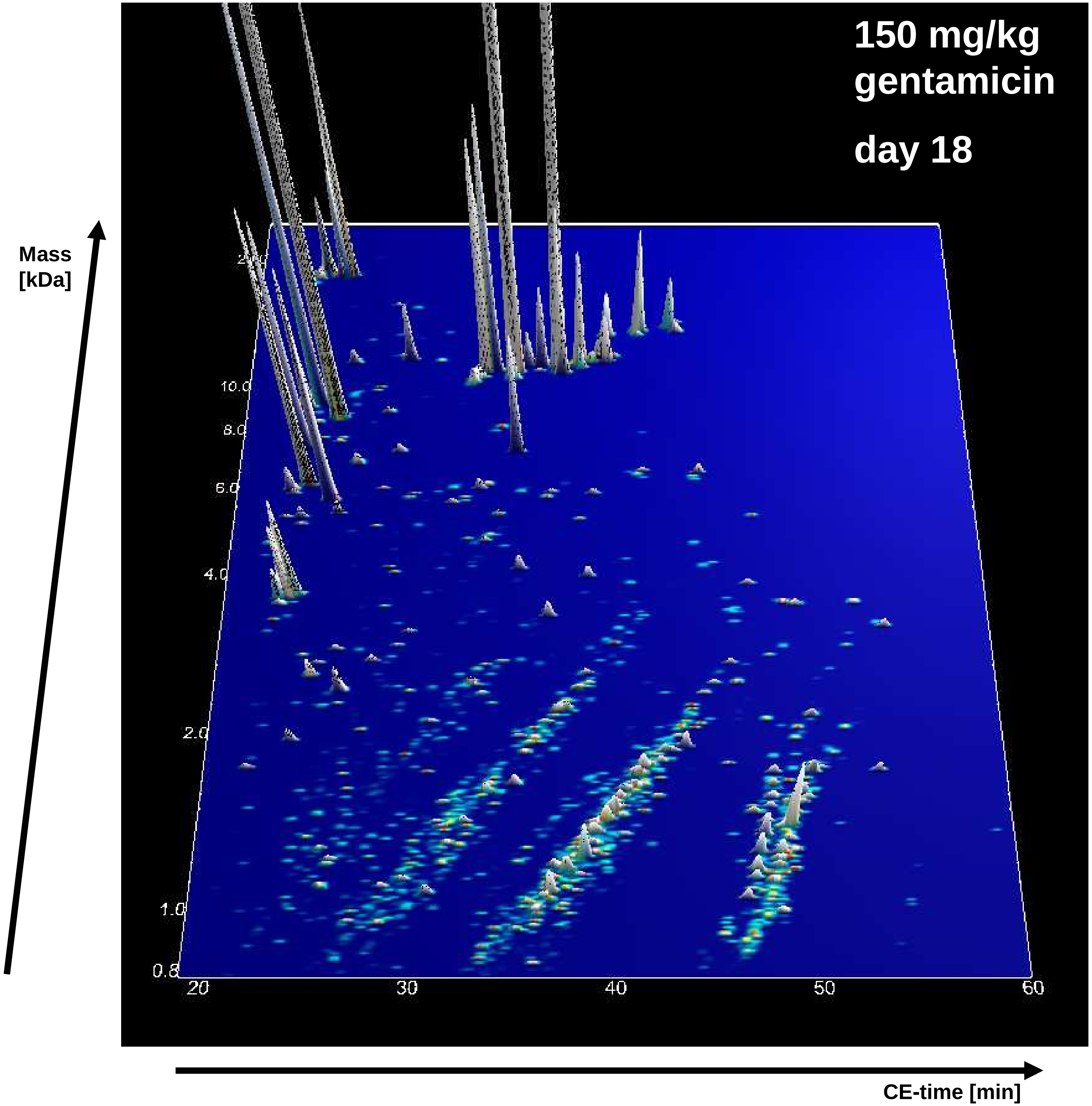

150 mg/kg
gentamicin
day 18
Mass
[kDa]
20.0
10.0
8.0
6.0
4.0
2.0
1.0
0.8
20
30
40
50
60
CE-time [min]

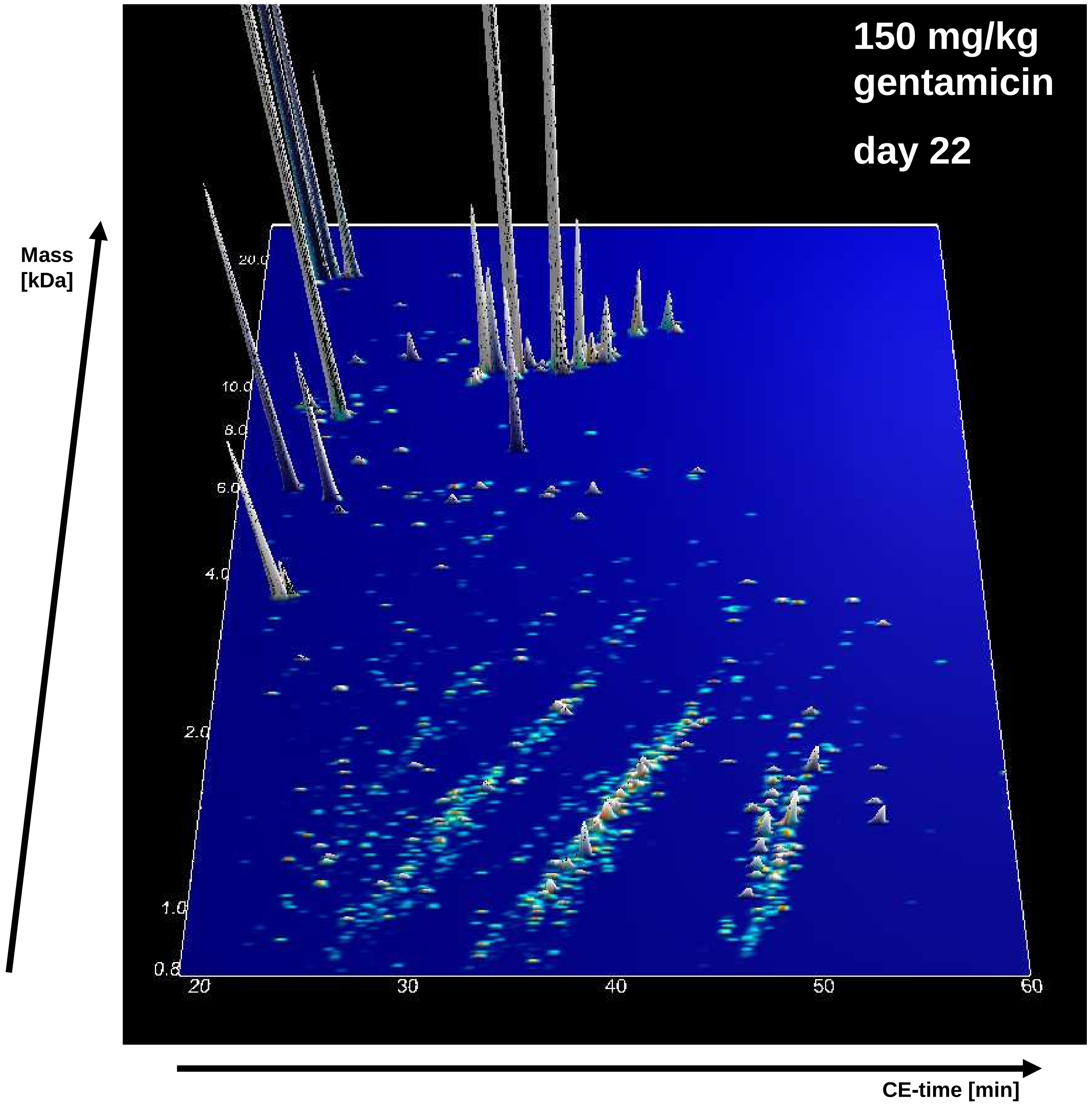

150 mg/kg
gentamicin
day 22
Mass
[kDa]
20.0
10.0
8.0
6.0
4.0
2.0
1.0
0.8
20
30
40
50
60
CE-time [min]

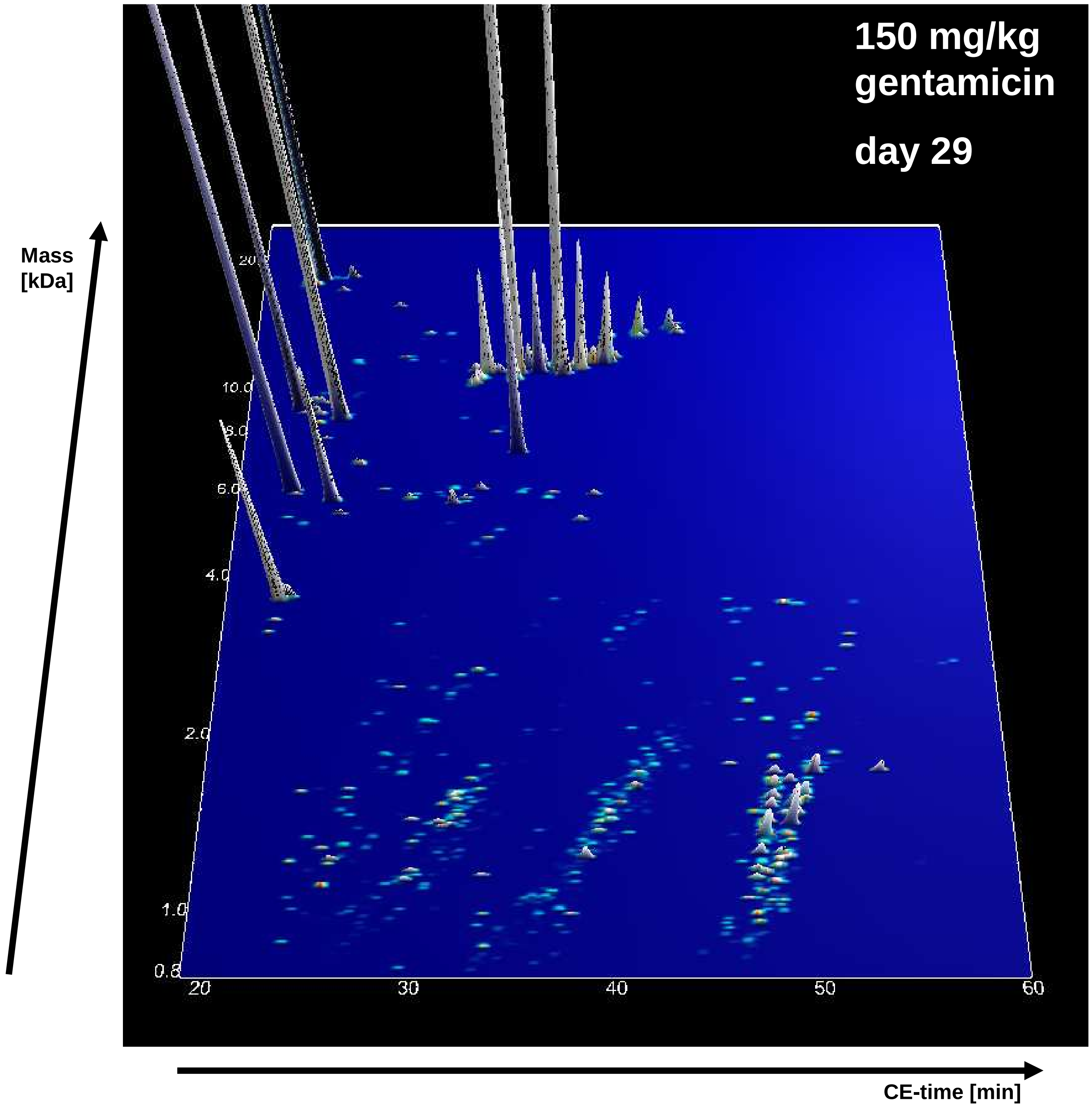
150 mg/kg
gentamicin
day 29
Mass
[kDa]
20.0
10.0
8.0
6.0
4.0
2.0
1.0
0.8
20
30
40
50
60
CE-time [min]

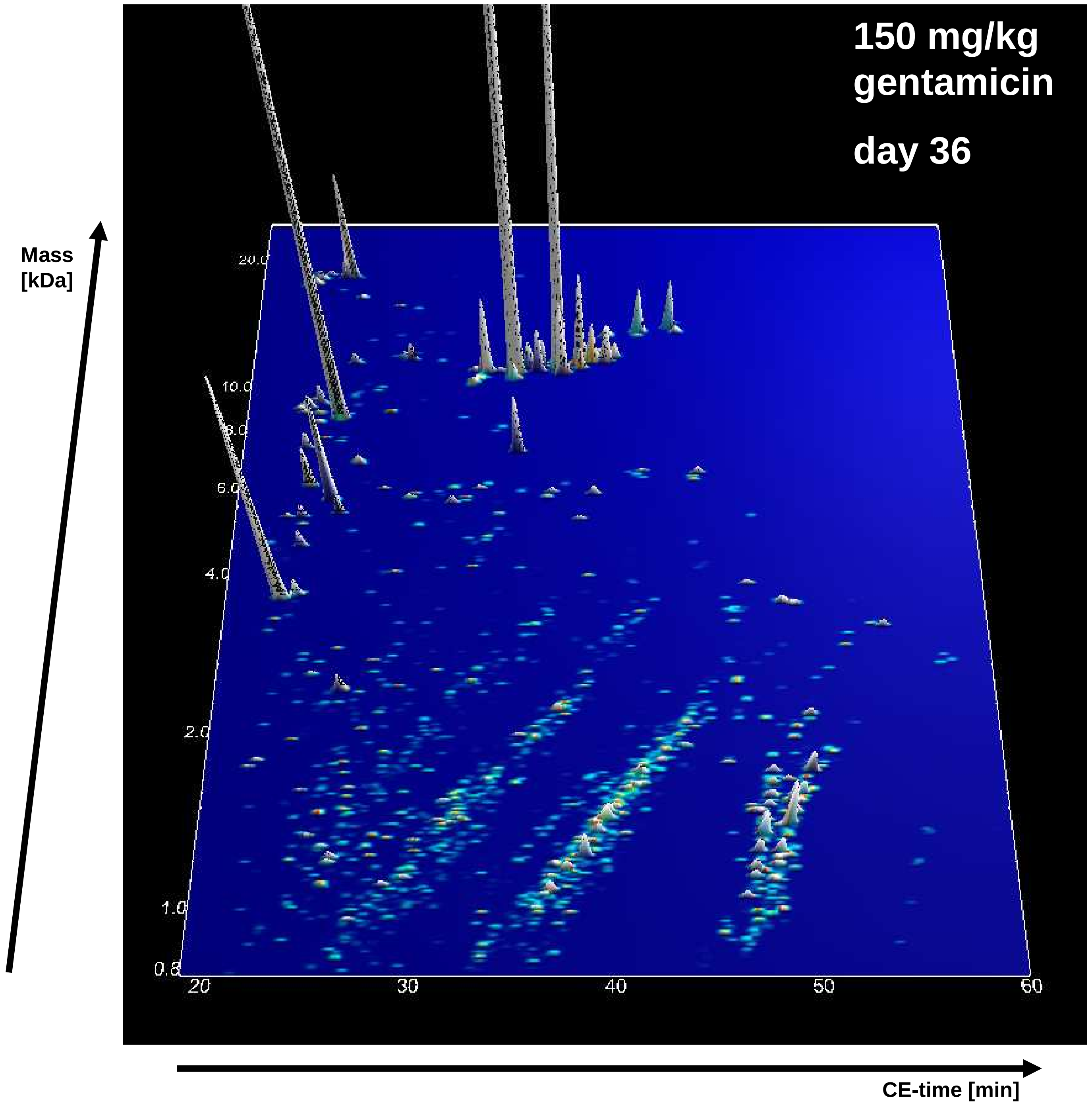
150 mg/kg
gentamicin
day 36
Mass
[kDa]
20.0
10.0
8.0
6.0
4.0
2.0
1.0
0.8
20
30
40
50
60
CE-time [min]

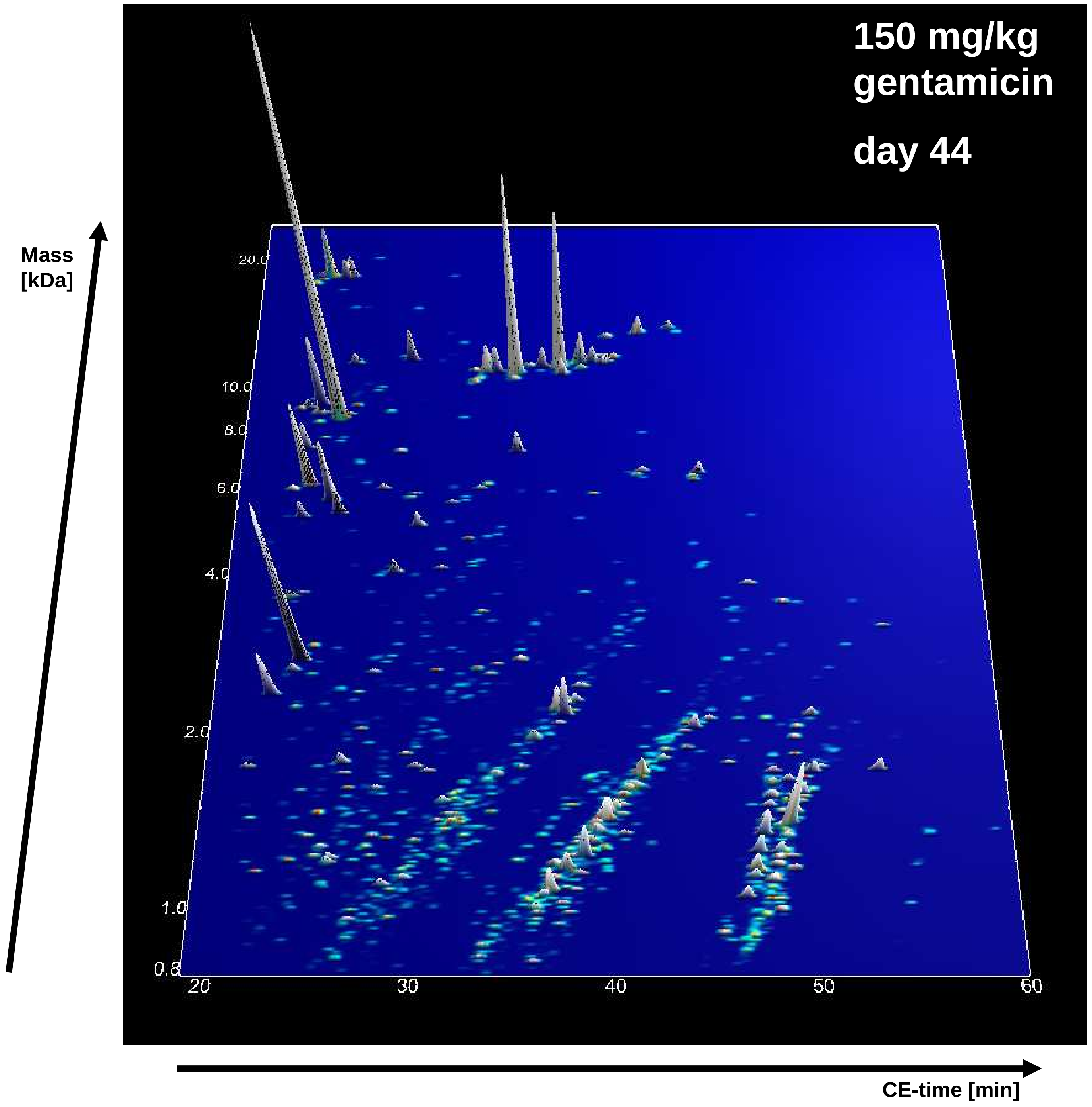
150 mg/kg
gentamicin
day 44
Mass
[kDa]
20.0
10.0
8.0
6.0
4.0
2.0
1.0
0.8
20
30
40
50
60
CE-time [min]

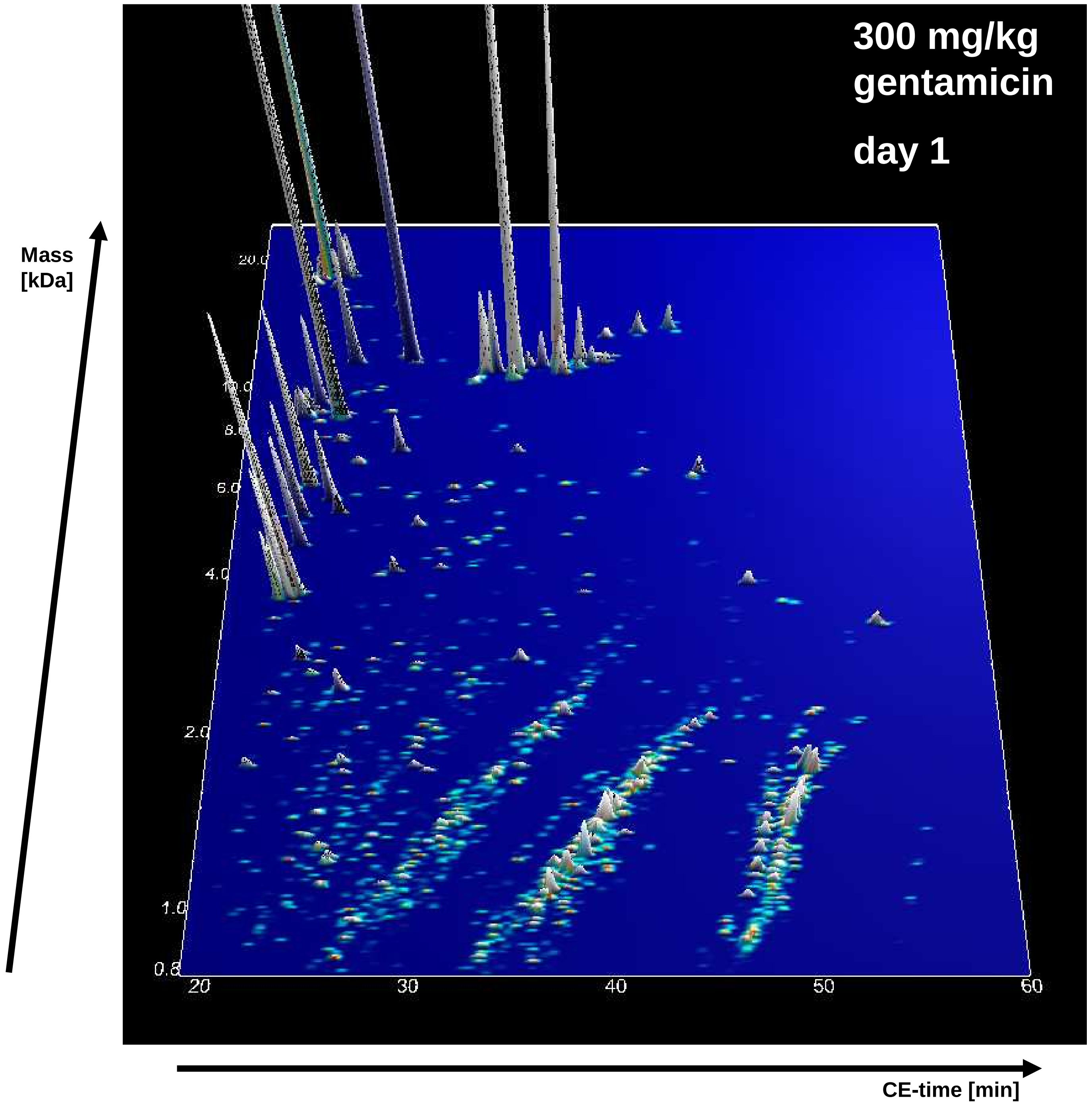

300 mg/kg
gentamicin
day 1
Mass
[kDa]
20.0
10.0
8.0
6.0
4.0
2.0
1.0
0.8
20
30
40
50
60
CE-time [min]

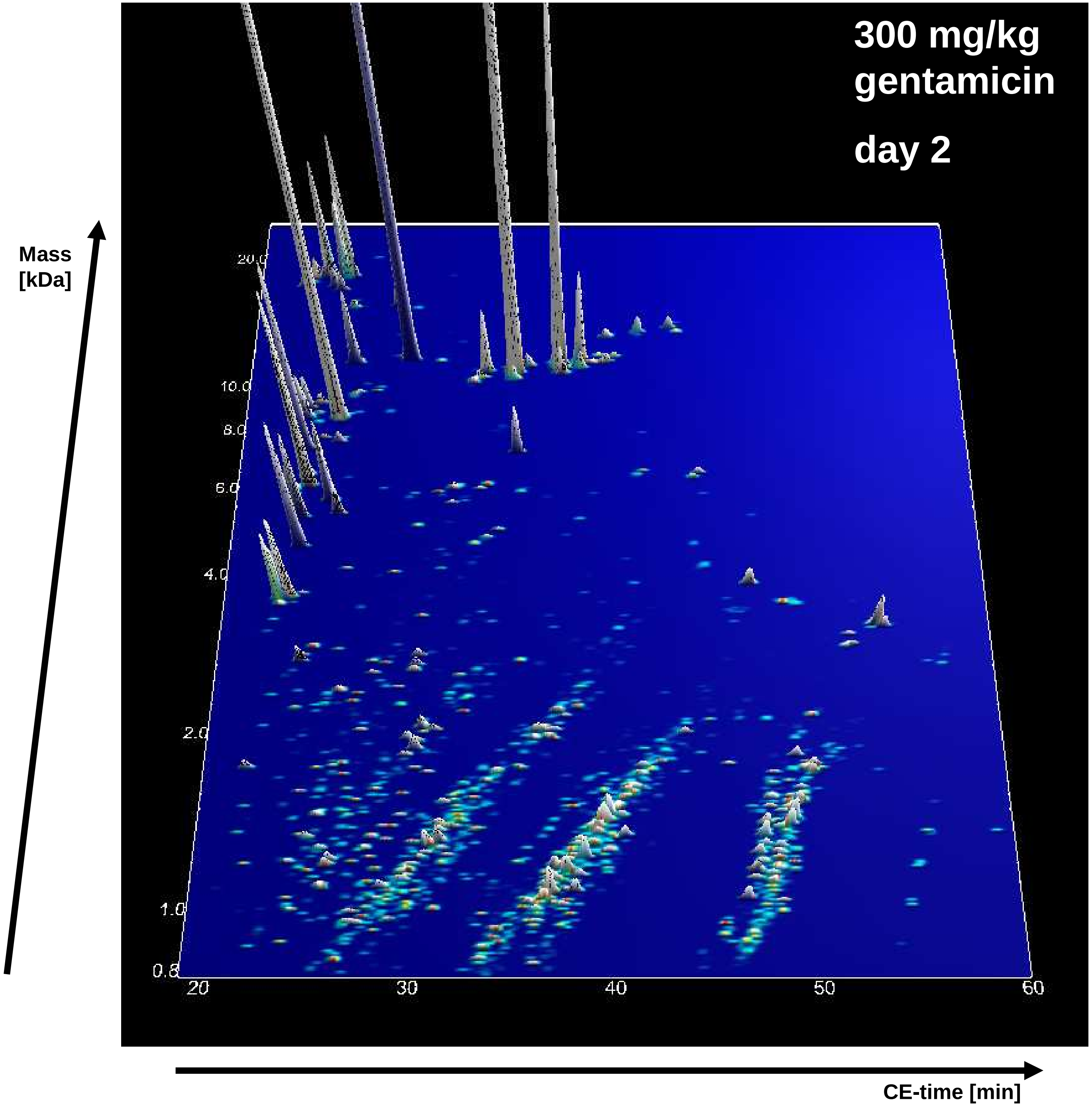

300 mg/kg
gentamicin
day 2
Mass
[kDa]
20.0
10.0
8.0
6.0
4.0
2.0
1.0
0.8
20
30
40
50
60
CE-time [min]

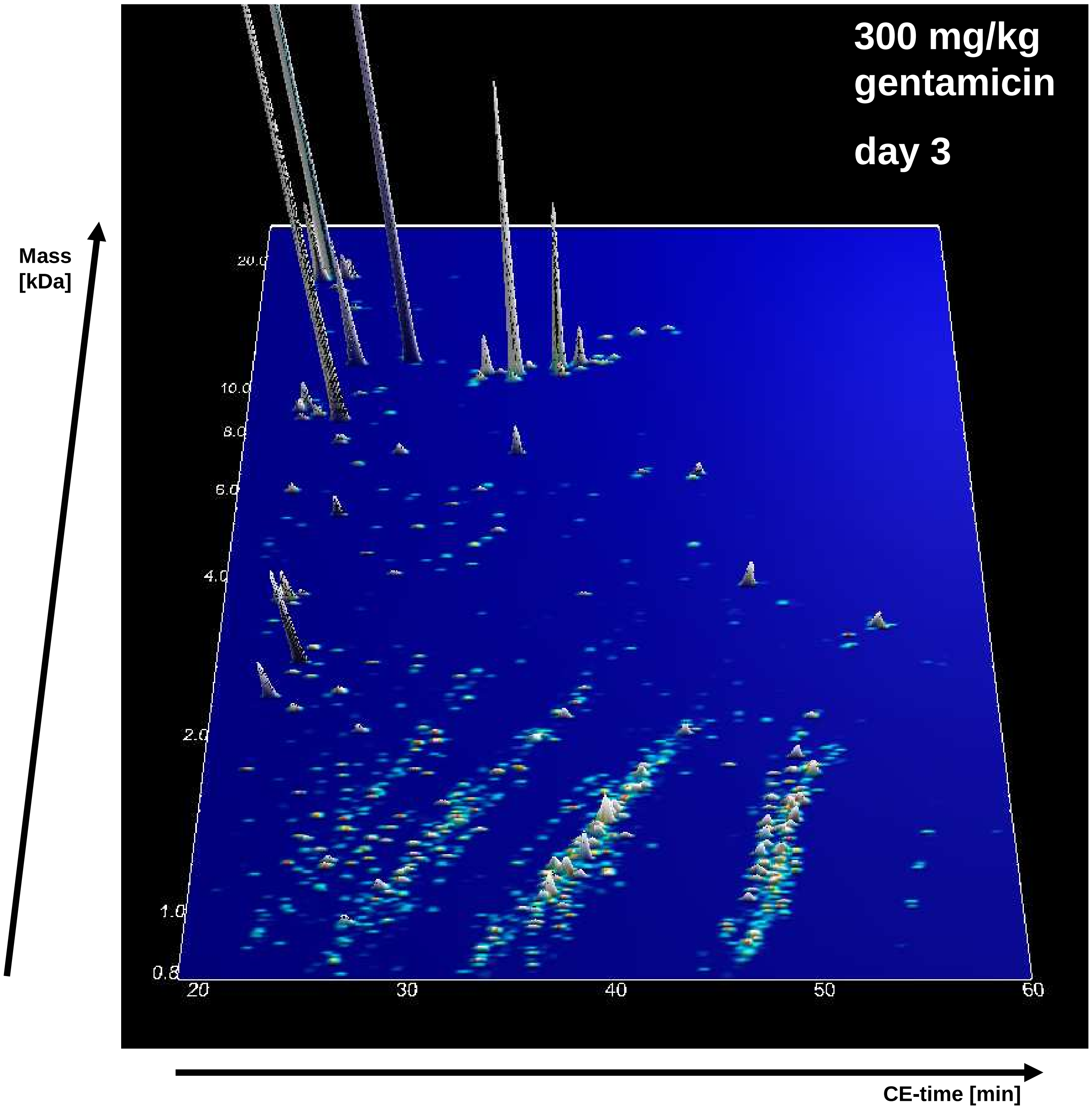
300 mg/kg
gentamicin
day 3
Mass
[kDa]
20.0
10.0
8.0
6.0
4.0
2.0
1.0
0.8
20
30
40
50
60
CE-time [min]

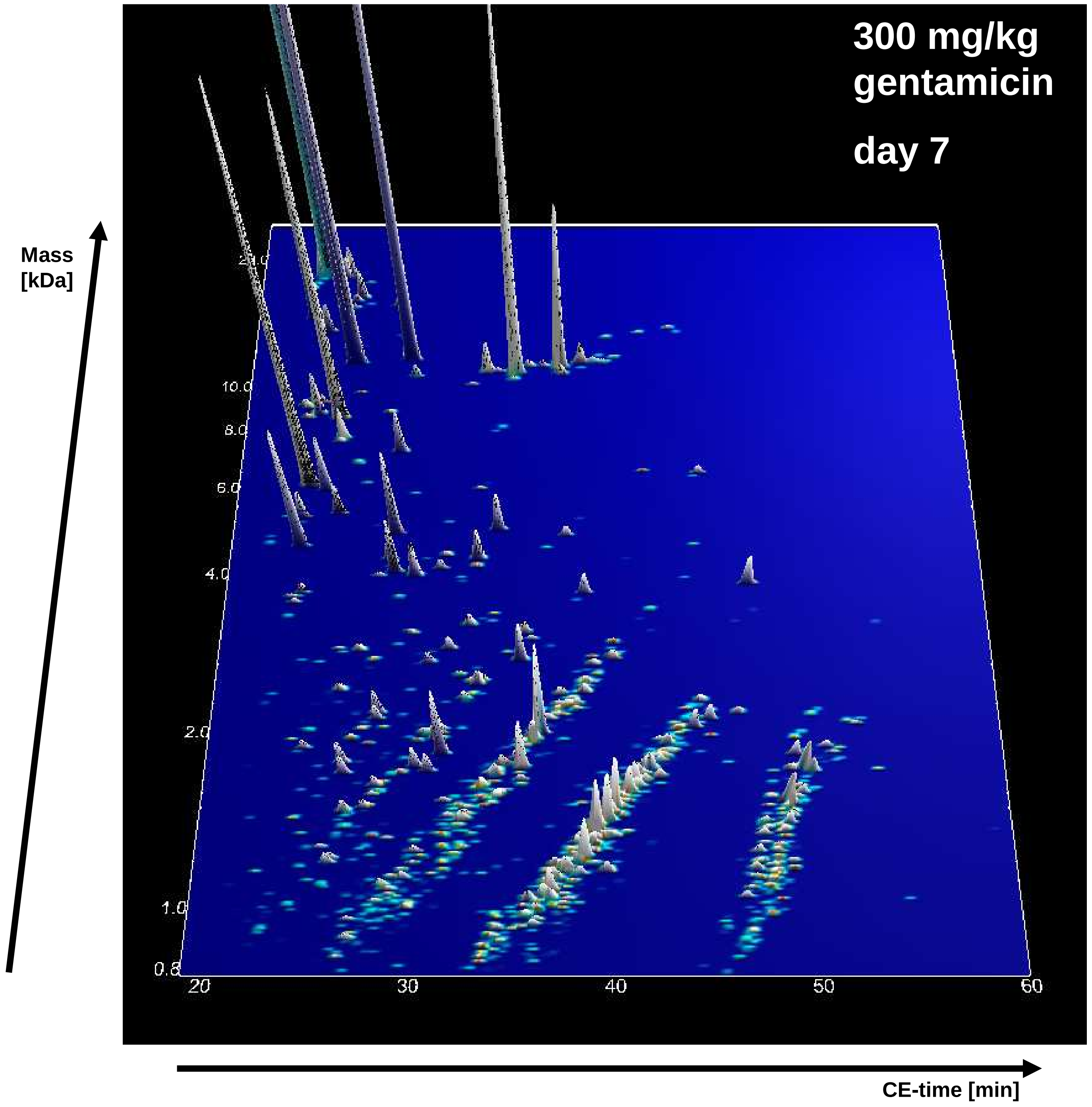

300 mg/kg
gentamicin
day 7
Mass
[kDa]
20.0
10.0
8.0
6.0
4.0
2.0
1.0
0.8
20
30
40
50
60
CE-time [min]

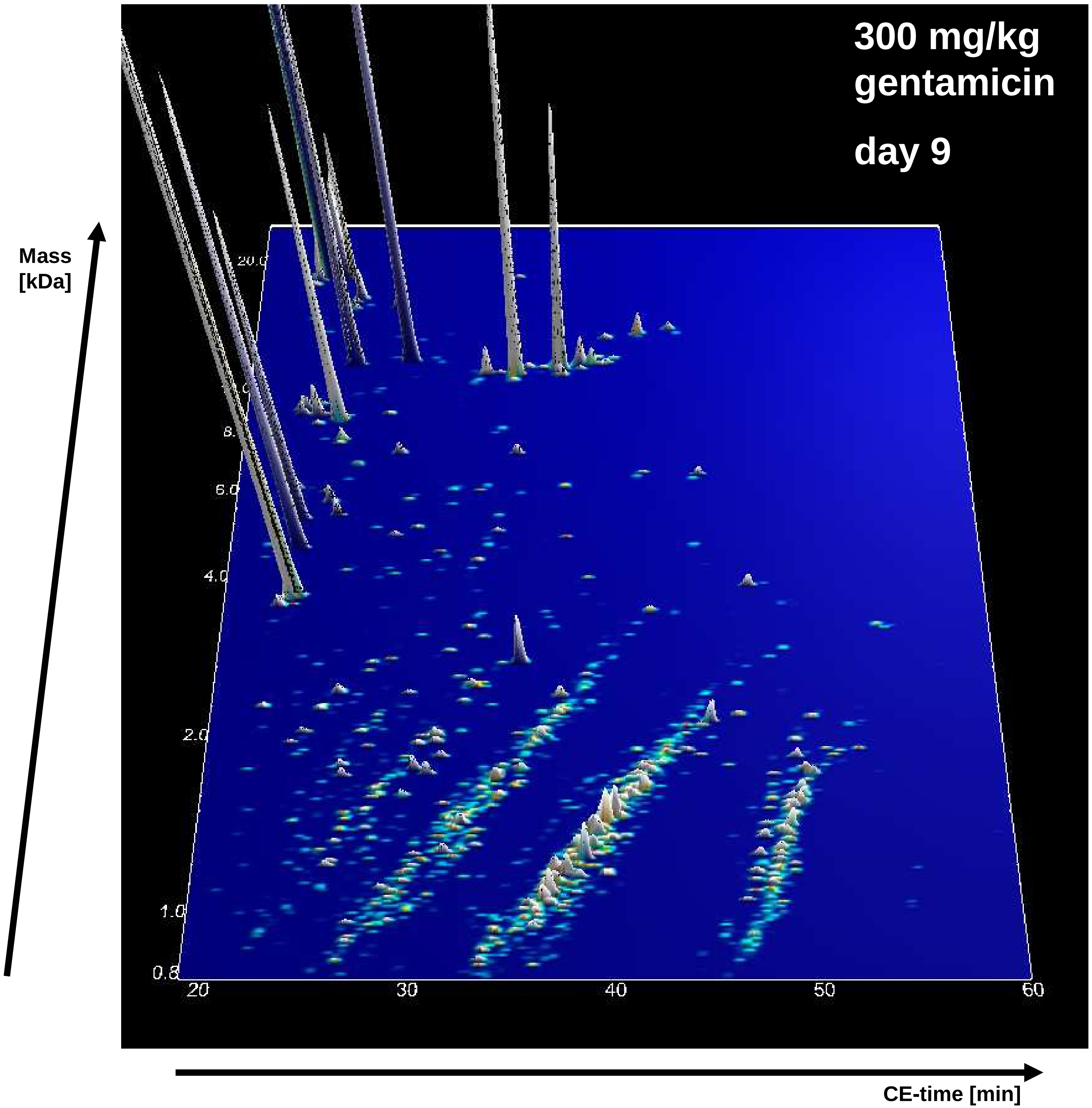

300 mg/kg
gentamicin
day 9
Mass
[kDa]
20.0
8.
6.0
4.0
2.0
1.0
0.8
20
30
40
50
60
CE-time [min]

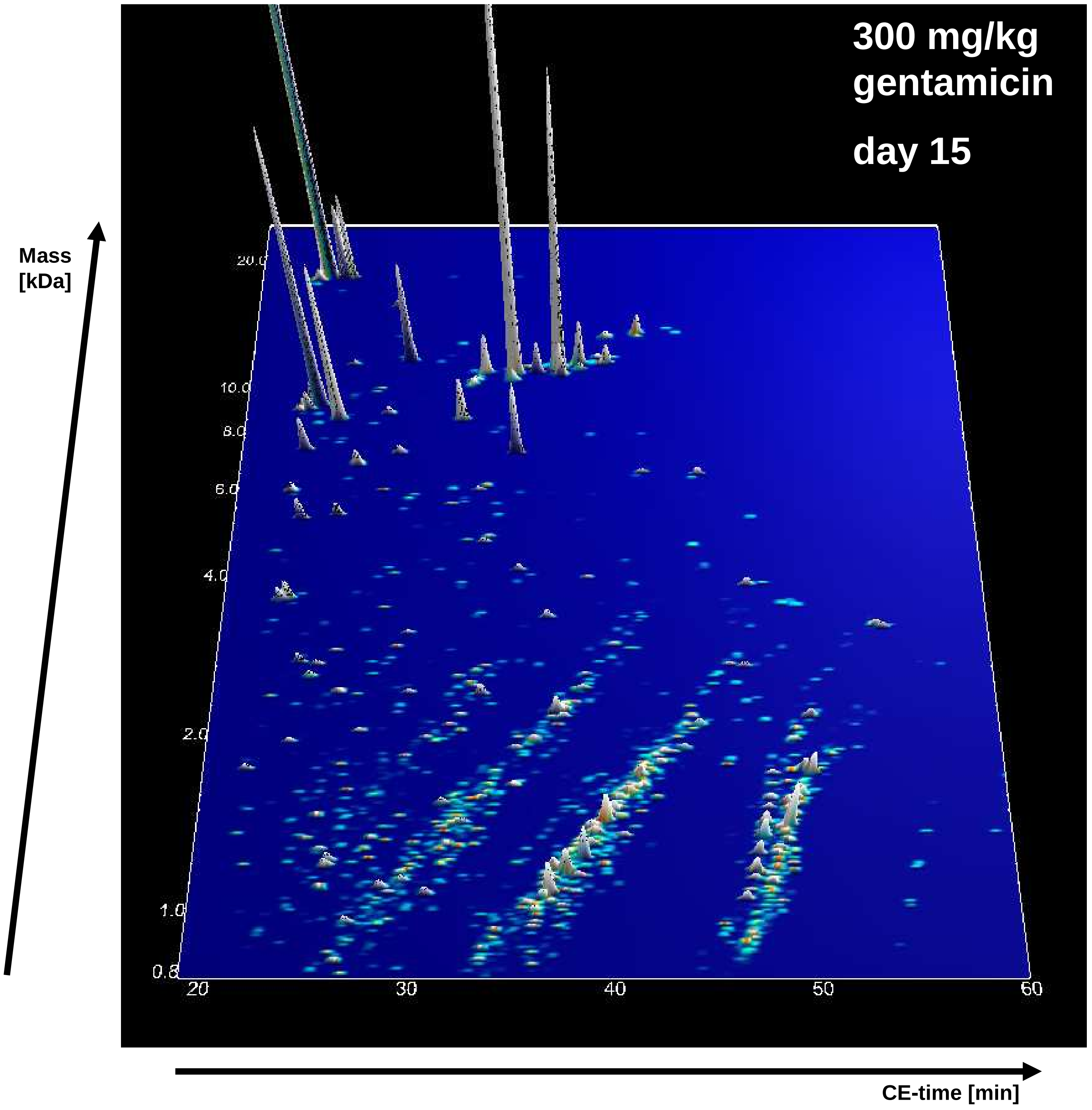

300 mg/kg
gentamicin
day 15
Mass
[kDa]
20.0
10.0
8.0
6.0
4.0
2.0
1.0
0.8
20
30
40
50
60
CE-time [min]

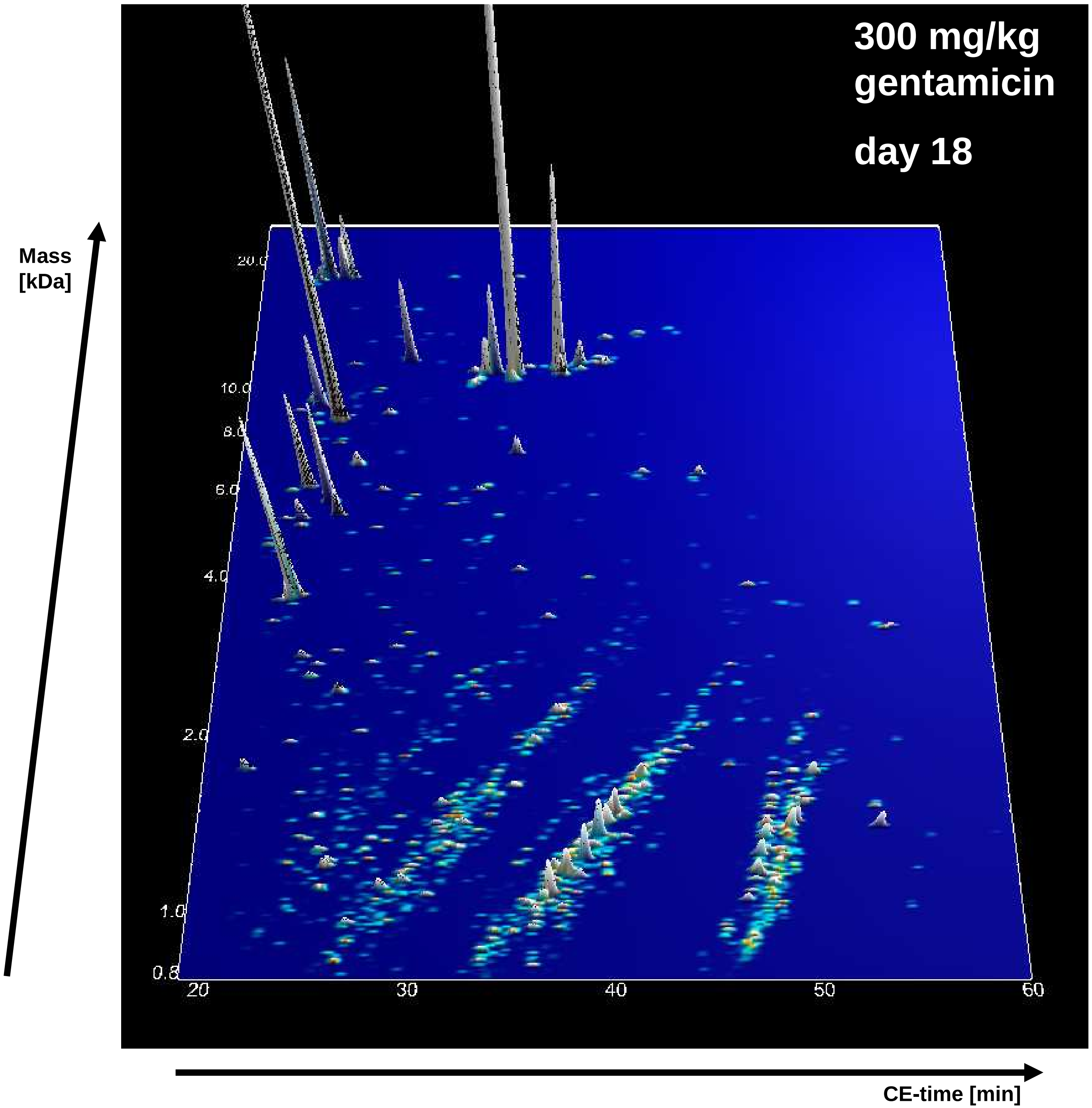

300 mg/kg
gentamicin
day 18
Mass
[kDa]
20.0
10.0
8.0
6.0
4.0
2.0
1.0
0.8
20
30
40
50
60
CE-time [min]

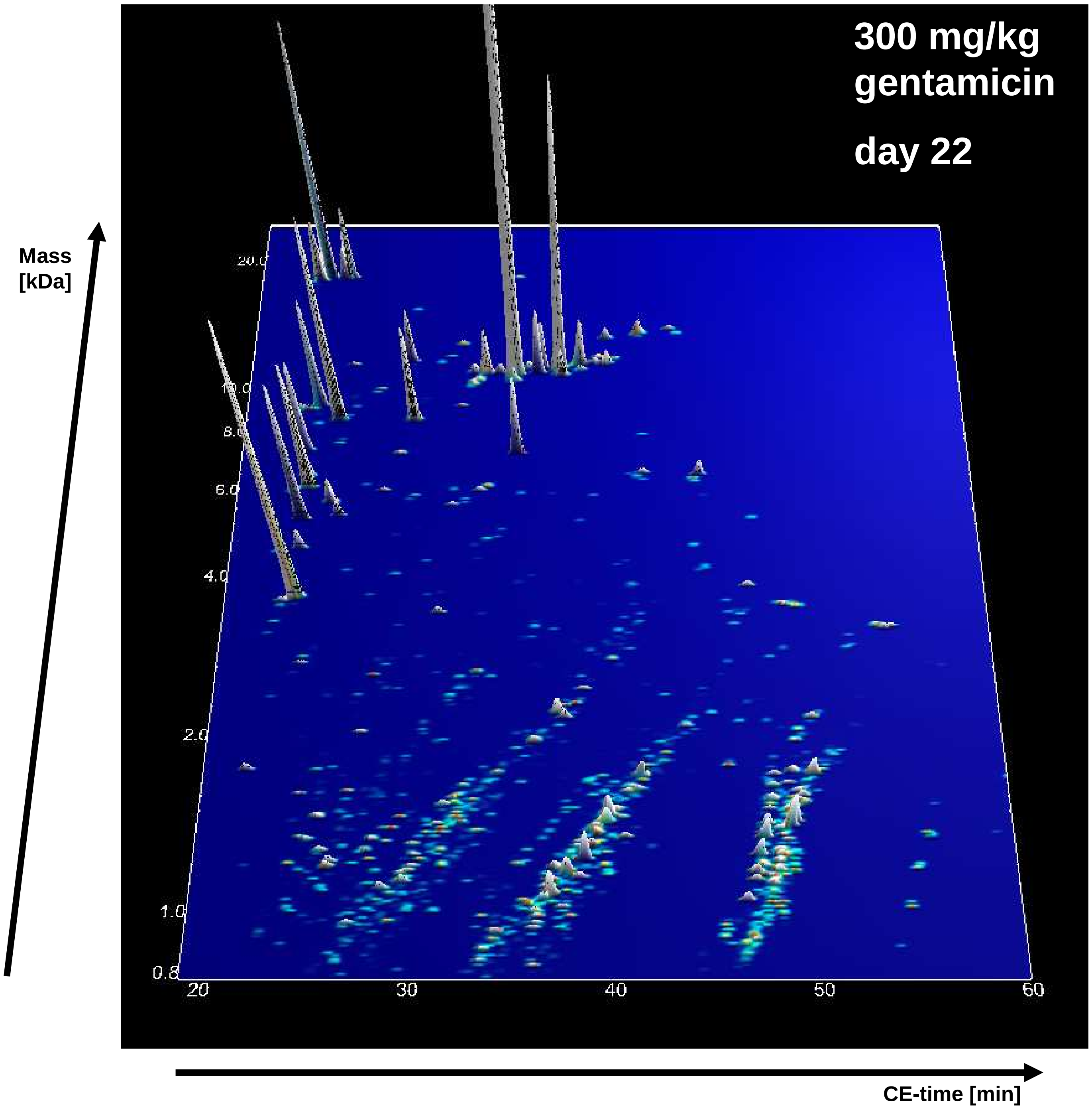

300 mg/kg
gentamicin
day 22
Mass
[kDa]
20.0
10.0
8.0
6.0
4.0
2.0
1.0
0.8
20
30
40
50
60
CE-time [min]

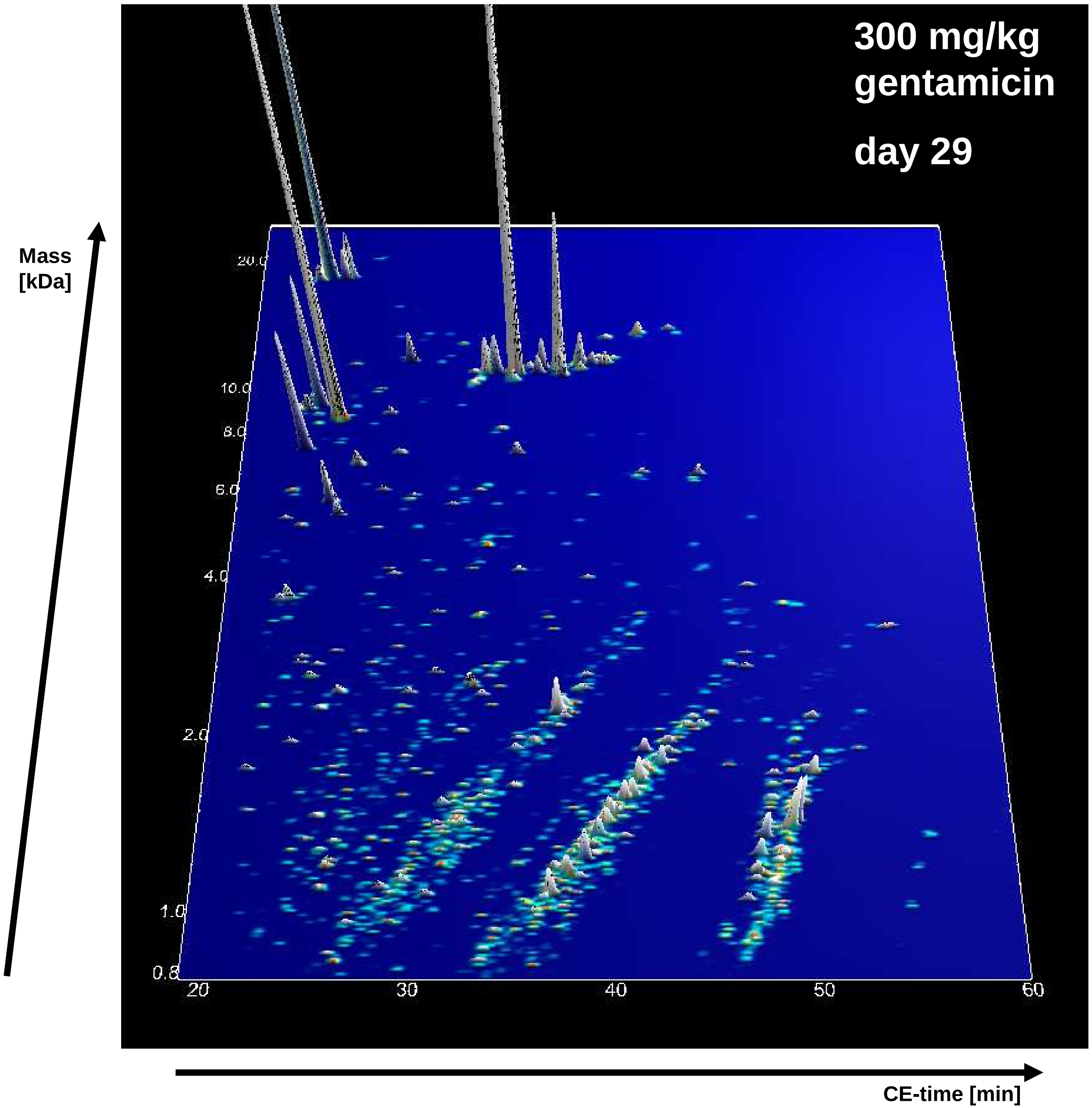
300 mg/kg
gentamicin
day 29
Mass
[kDa]
20.0
10.0
8.0
6.0
4.0
2.0
1.0
0.8
20
30
40
50
60
CE-time [min]

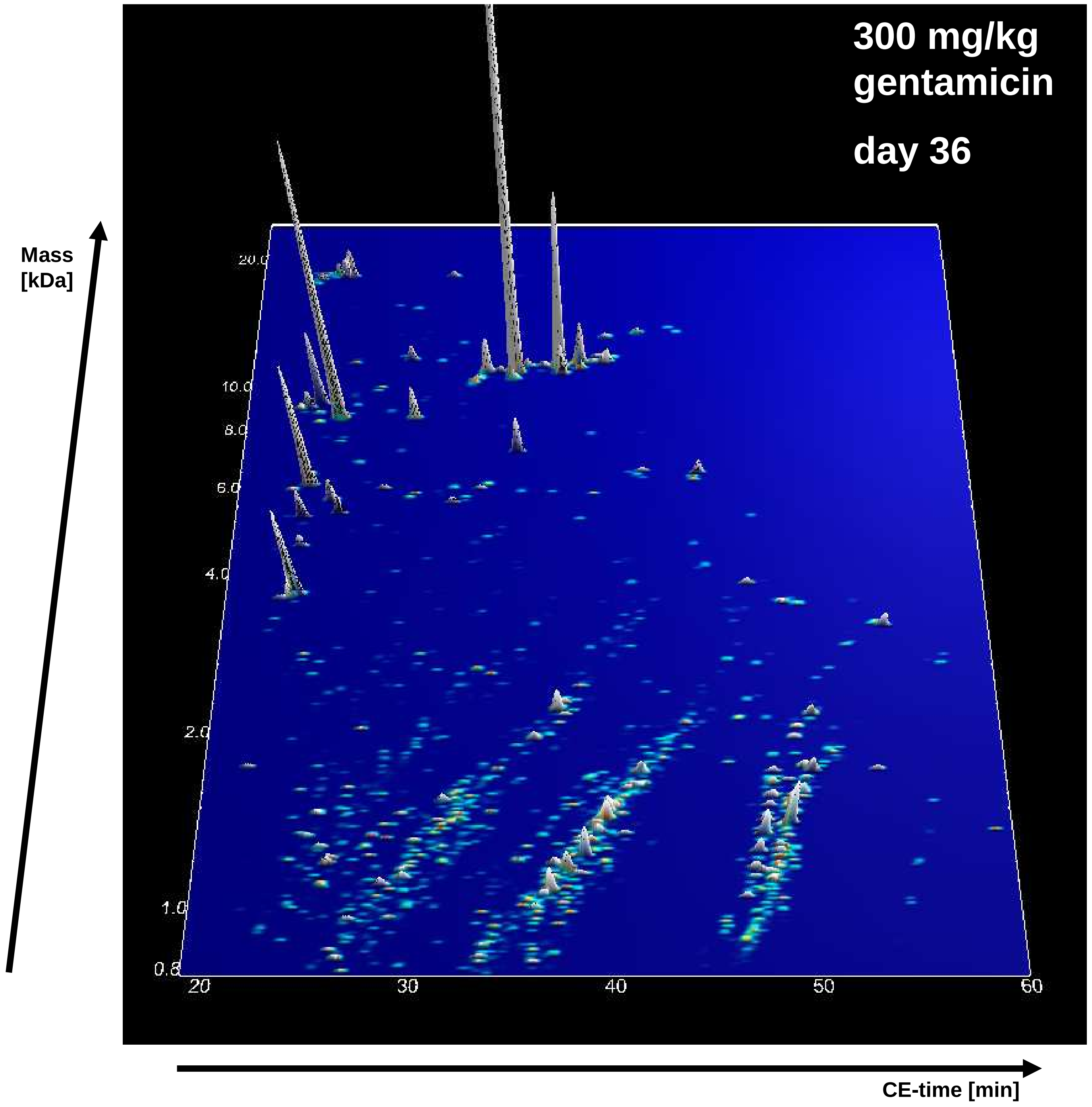
300 mg/kg
gentamicin
day 36
Mass
[kDa]
20.0
10.0
8.0
6.0
4.0
2.0
1.0
0.8
20
30
40
50
60
CE-time [min]

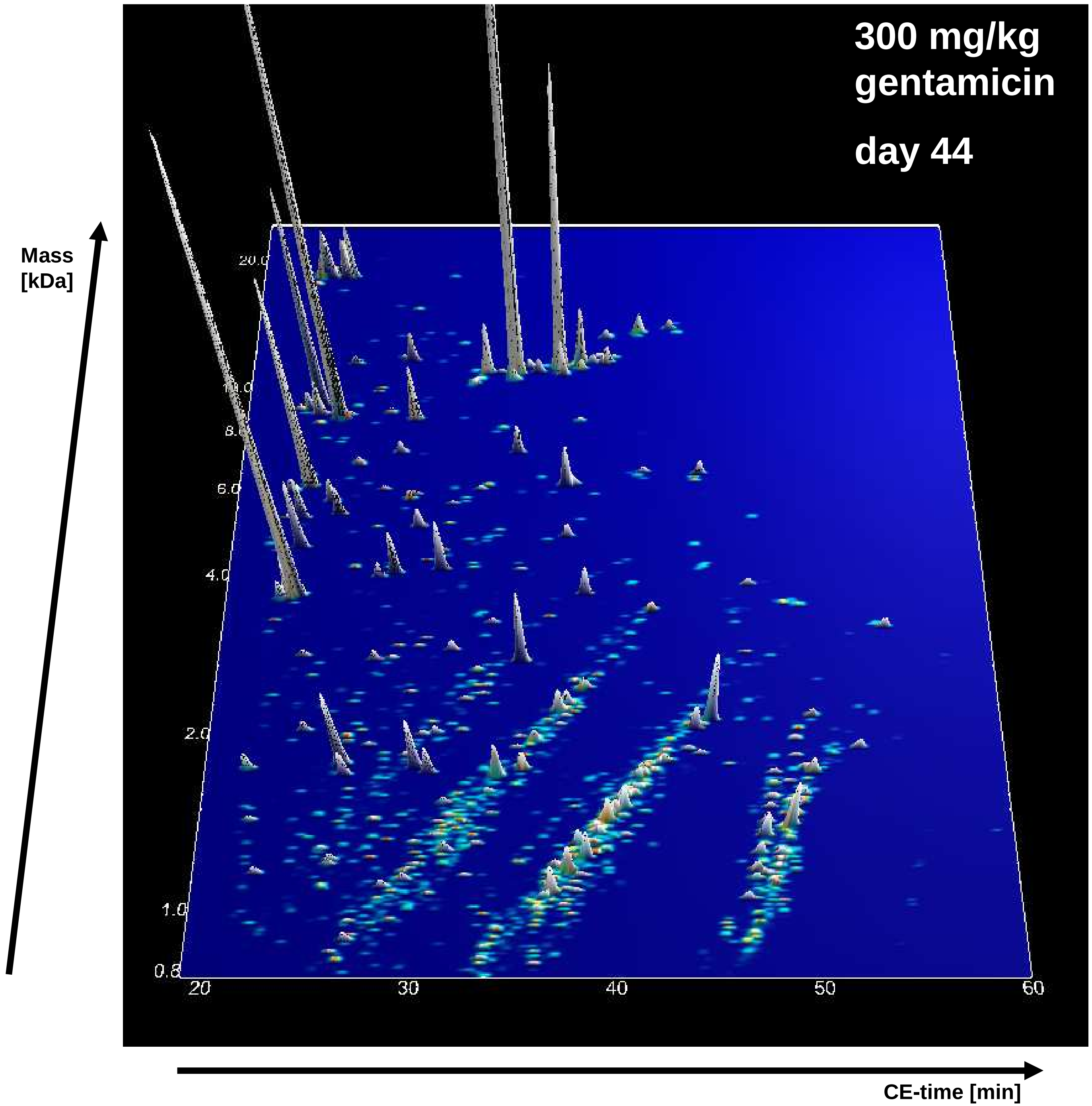

300 mg/kg
gentamicin
day 44
Mass
[kDa]
20.0
10.0
8.0
6.0
4.0
2.0
1.0
0.8
20
30
40
50
60
CE-time [min]
