# Supplementary material for: Proteomic Candidate Biomarkers of Drug-Induced Nephrotoxicity in the Rat
Source: PLoS One. 2012 Apr 11;7(4):e34606. doi: 10.1371/journal.pone.0034606 (PMC3324487; doi:10.1371/journal.pone.0034606)
Supplement: Figure S2 — Time course of mean signal intensities of all defined (n = 147) nephrotoxicity peptide maker candidates. Mean signal intensities of the respective peptide in urine samples from untreated animals and animals treated once daily for three consecutive days with 150 and 300 mg/kg gentamicin over time are shown. The first 39 diagrams depict the selected cis-platin markers and the subsequent figures the additional 108 gentamicin markers. (PDF) [file pone.0034606.s002.pdf]

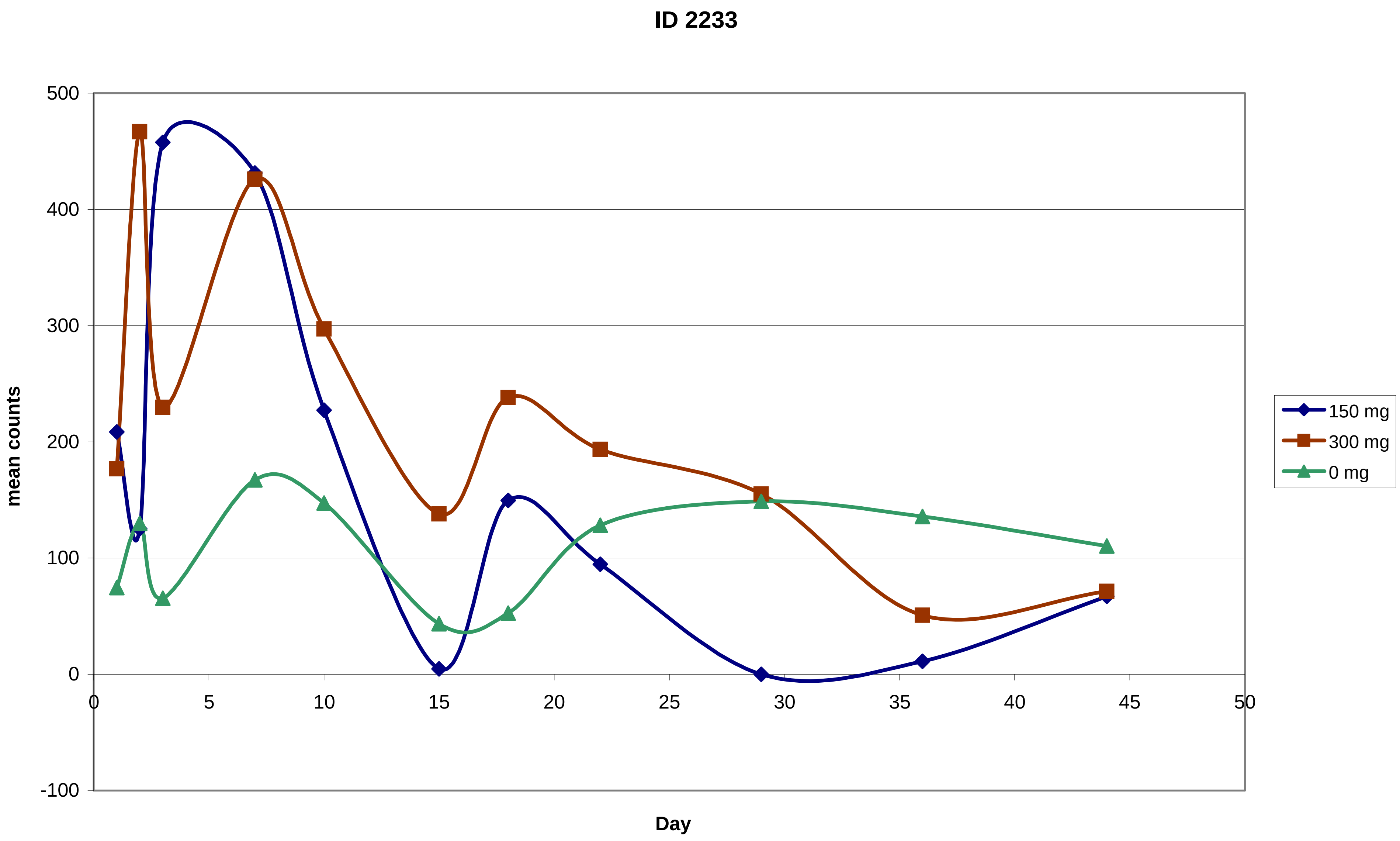
ID 2233
mean counts
Day
500
400
300
200
100
0
-100
0
5
10
15
20
25
30
35
40
45
50
150 mg
300 mg
0 mg

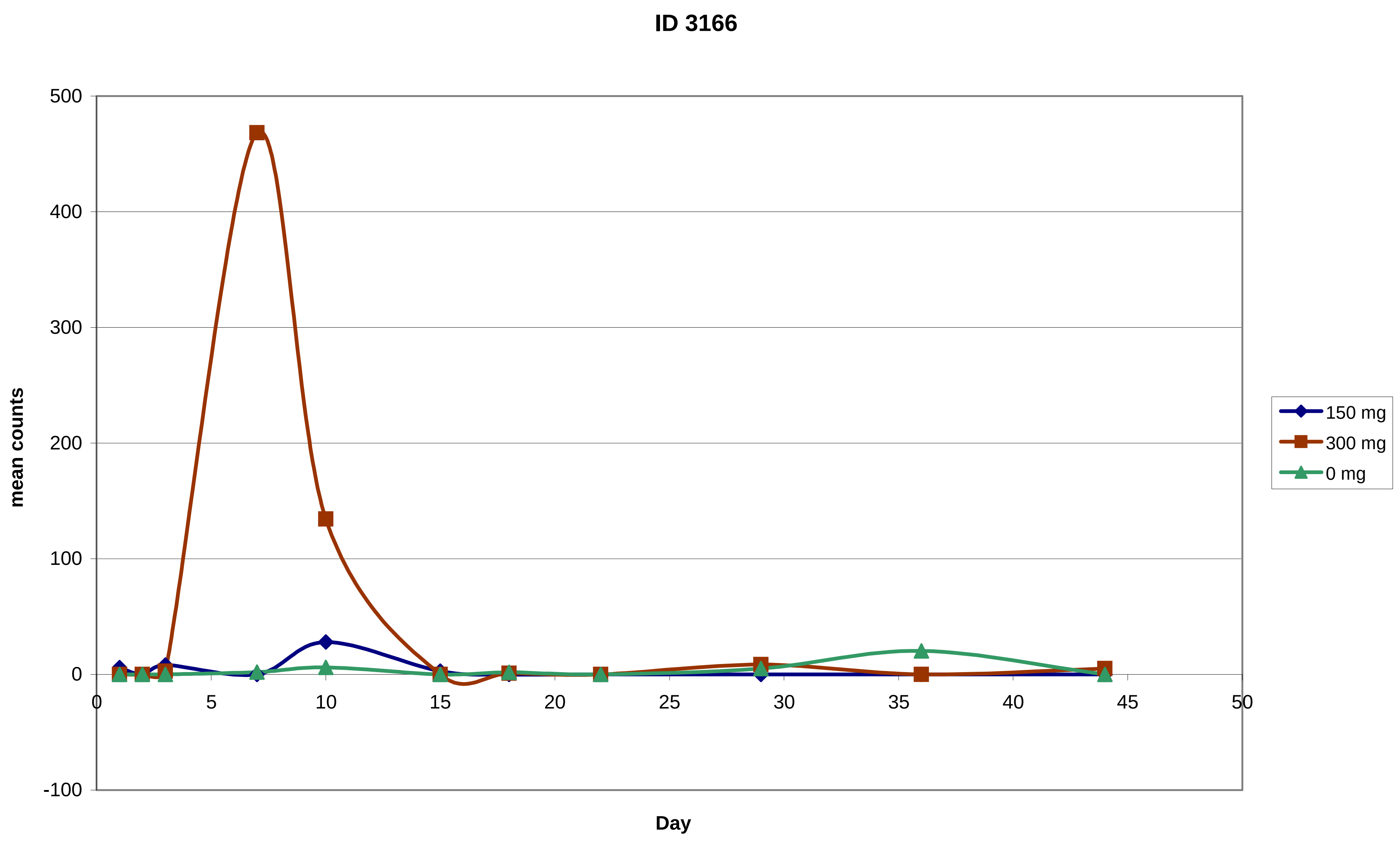

ID 3166
mean counts
500
400
300
200
100
0
-100
0
5
10
15
20
25
30
35
40
45
50
Day
150 mg
300 mg
0 mg

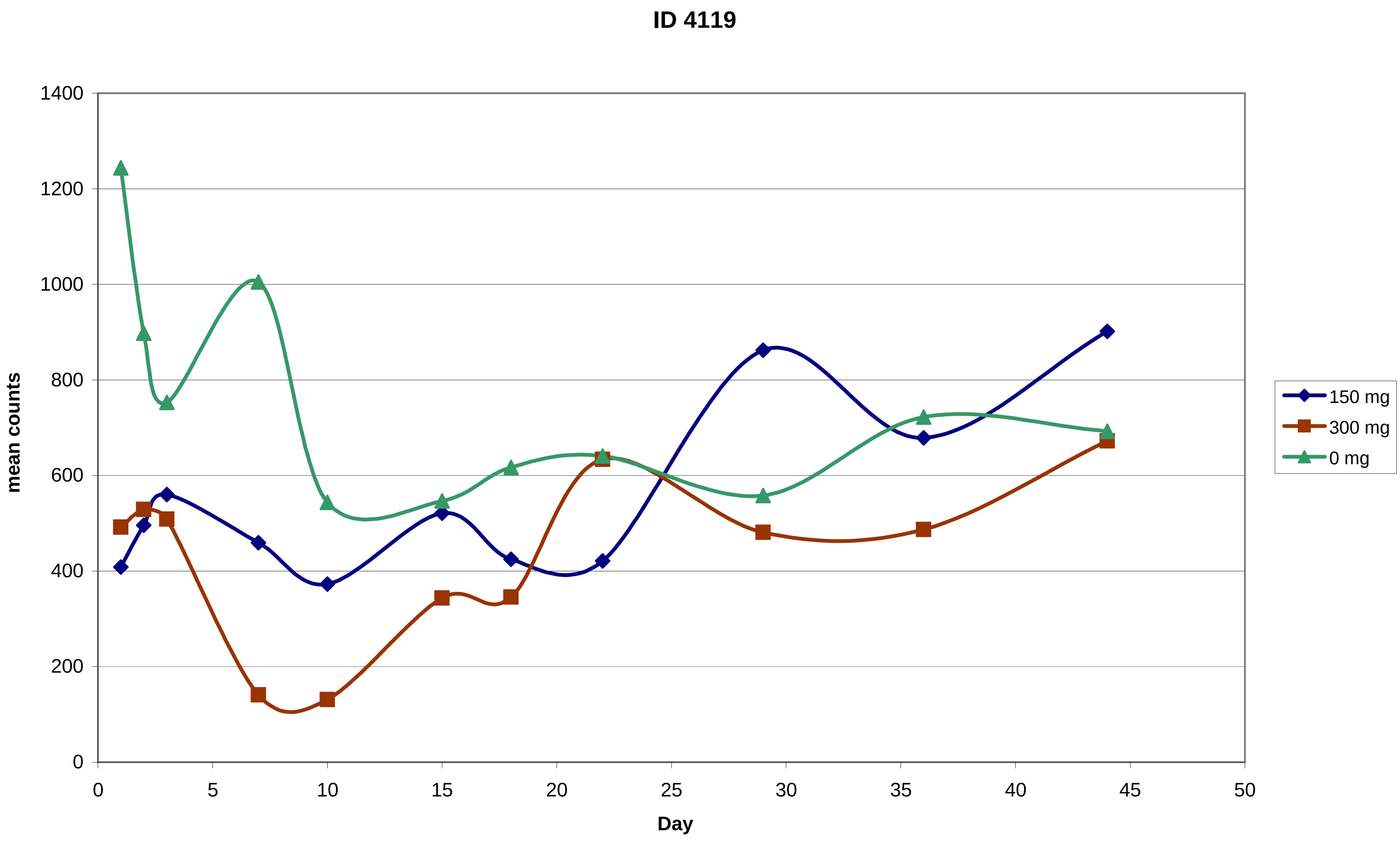
ID 4119
mean counts
Day
0
200
400
600
800
1000
1200
1400
0
5
10
15
20
25
30
35
40
45
50
150 mg
300 mg
0 mg

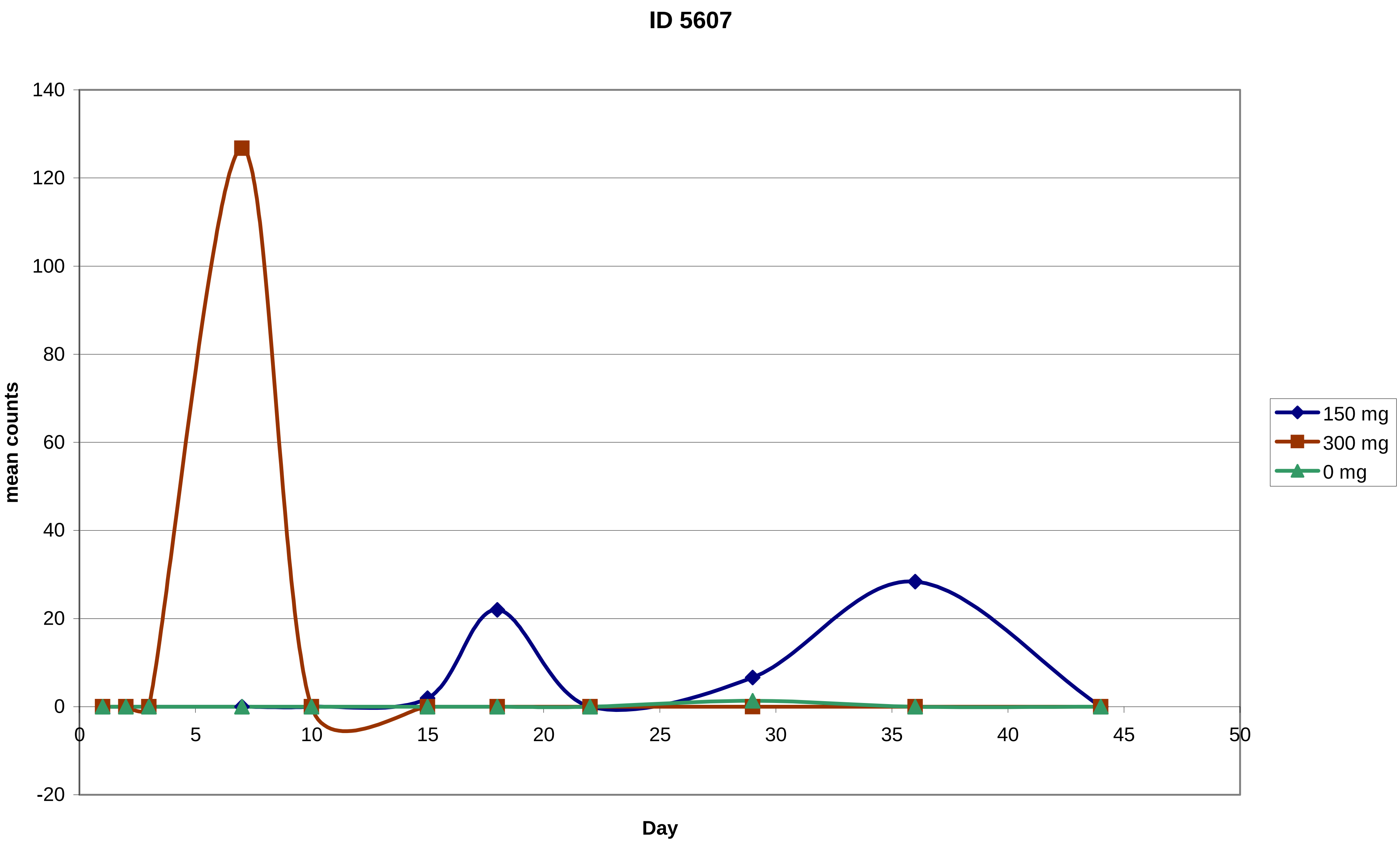

ID 5607
mean counts
140
120
100
80
60
40
20
0
-20
0
5
10
15
20
25
30
35
40
45
50
Day
150 mg
300 mg
0 mg

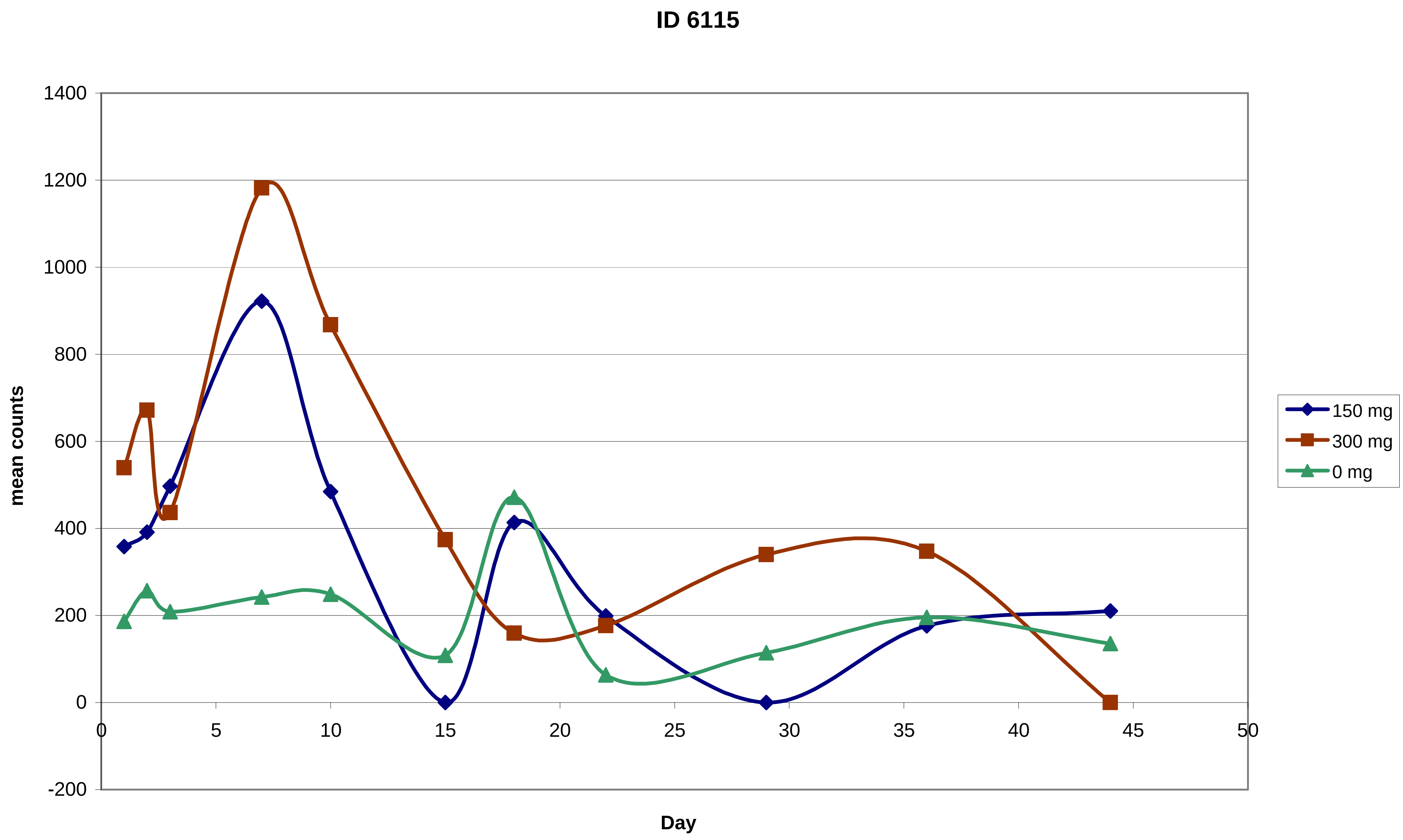
ID 6115
mean counts
1400
1200
1000
800
600
400
200
0
-200
0
5
10
15
20
25
30
35
40
45
50
Day
150 mg
300 mg
0 mg

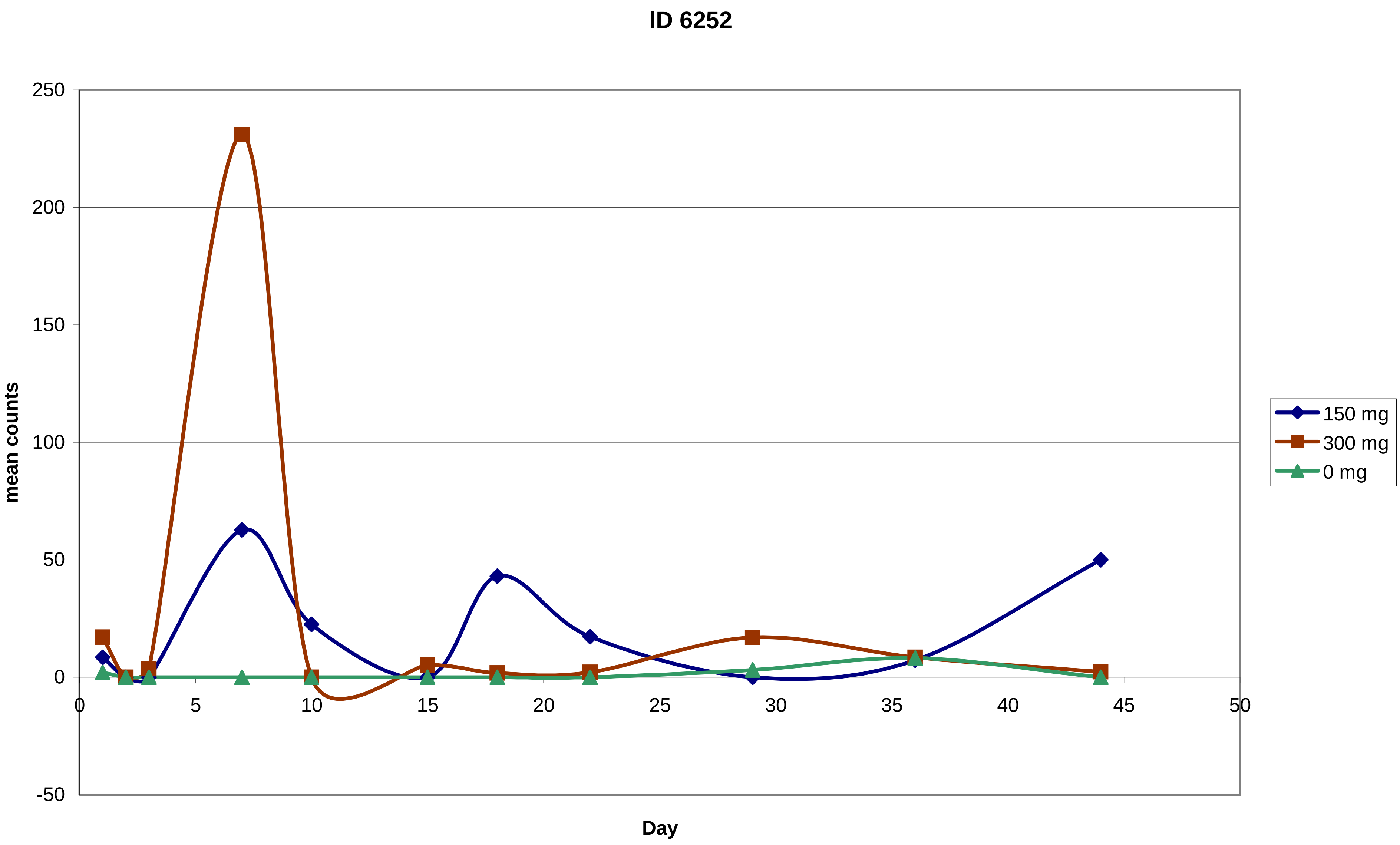

ID 6252
mean counts
250
200
150
100
50
0
-50
0
5
10
15
20
25
30
35
40
45
50
Day
150 mg
300 mg
0 mg

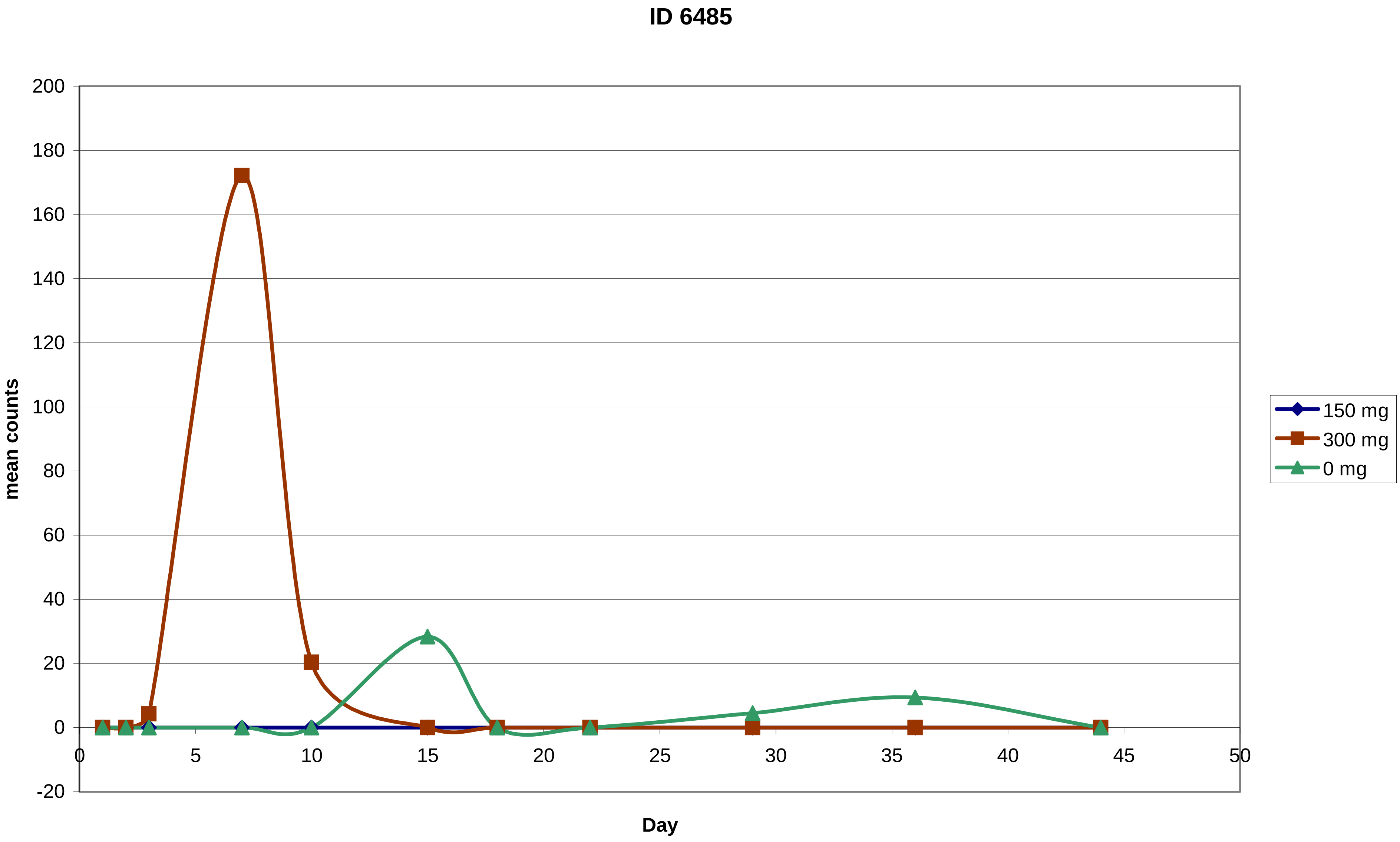
ID 6485
mean counts
200
180
160
140
120
100
80
60
40
20
0
-20
0
5
10
15
20
25
30
35
40
45
50
Day
150 mg
300 mg
0 mg

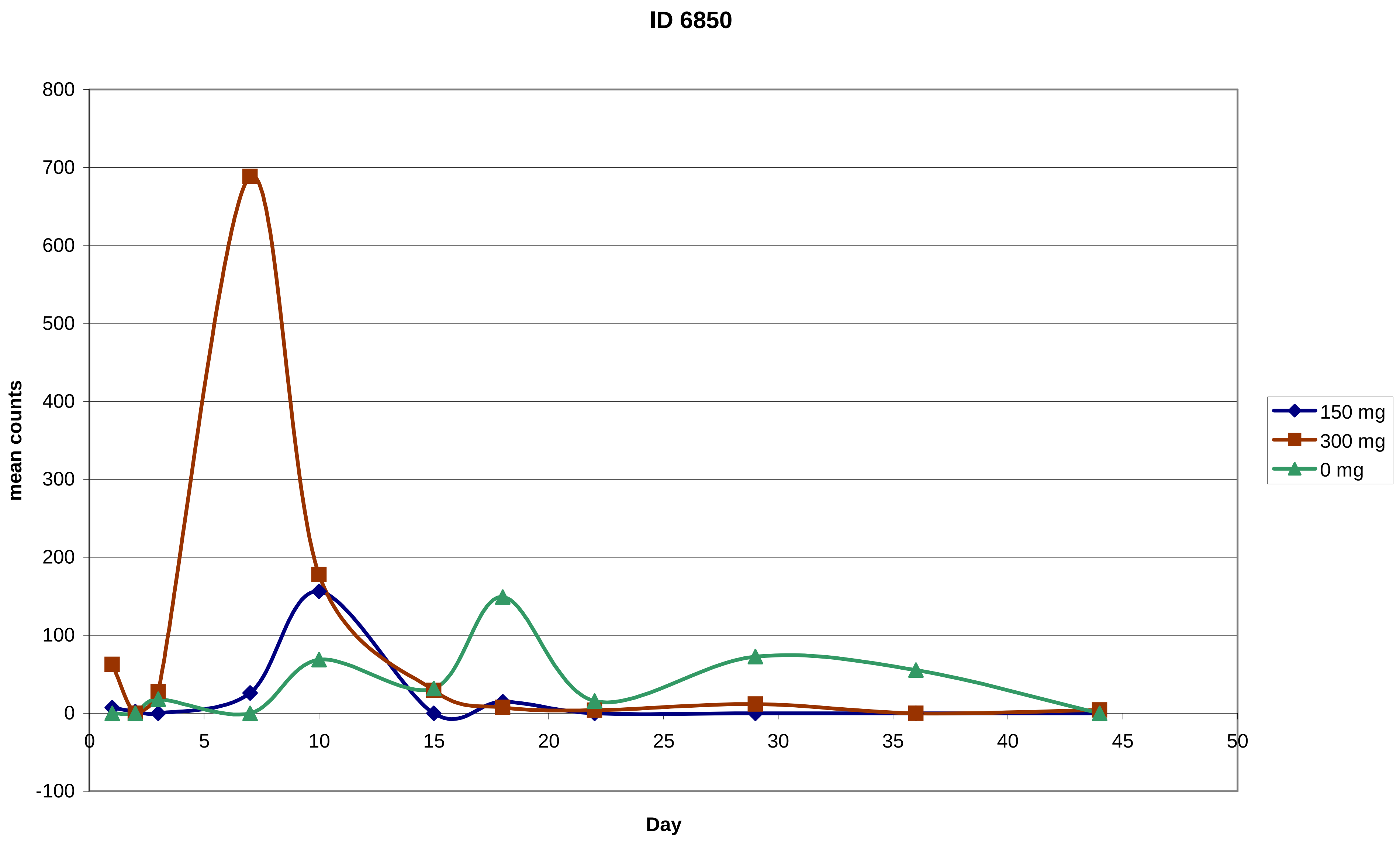
ID 6850
mean counts
800
700
600
500
400
300
200
100
0
-100
0
5
10
15
20
25
30
35
40
45
50
Day
150 mg
300 mg
0 mg

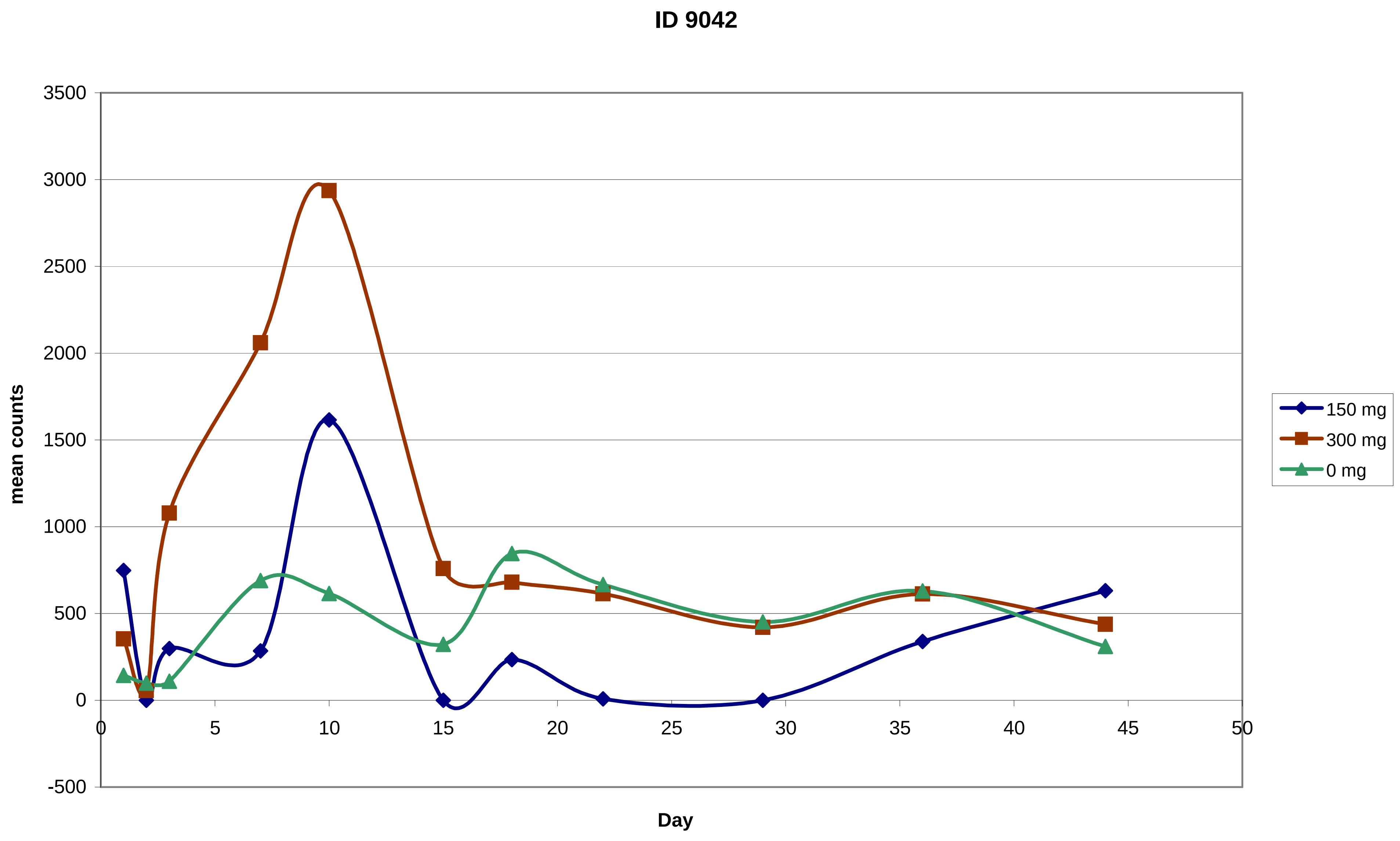
ID 9042
mean counts
3500
3000
2500
2000
1500
1000
500
0
-500
0
5
10
15
20
25
30
35
40
45
50
Day
150 mg
300 mg
0 mg

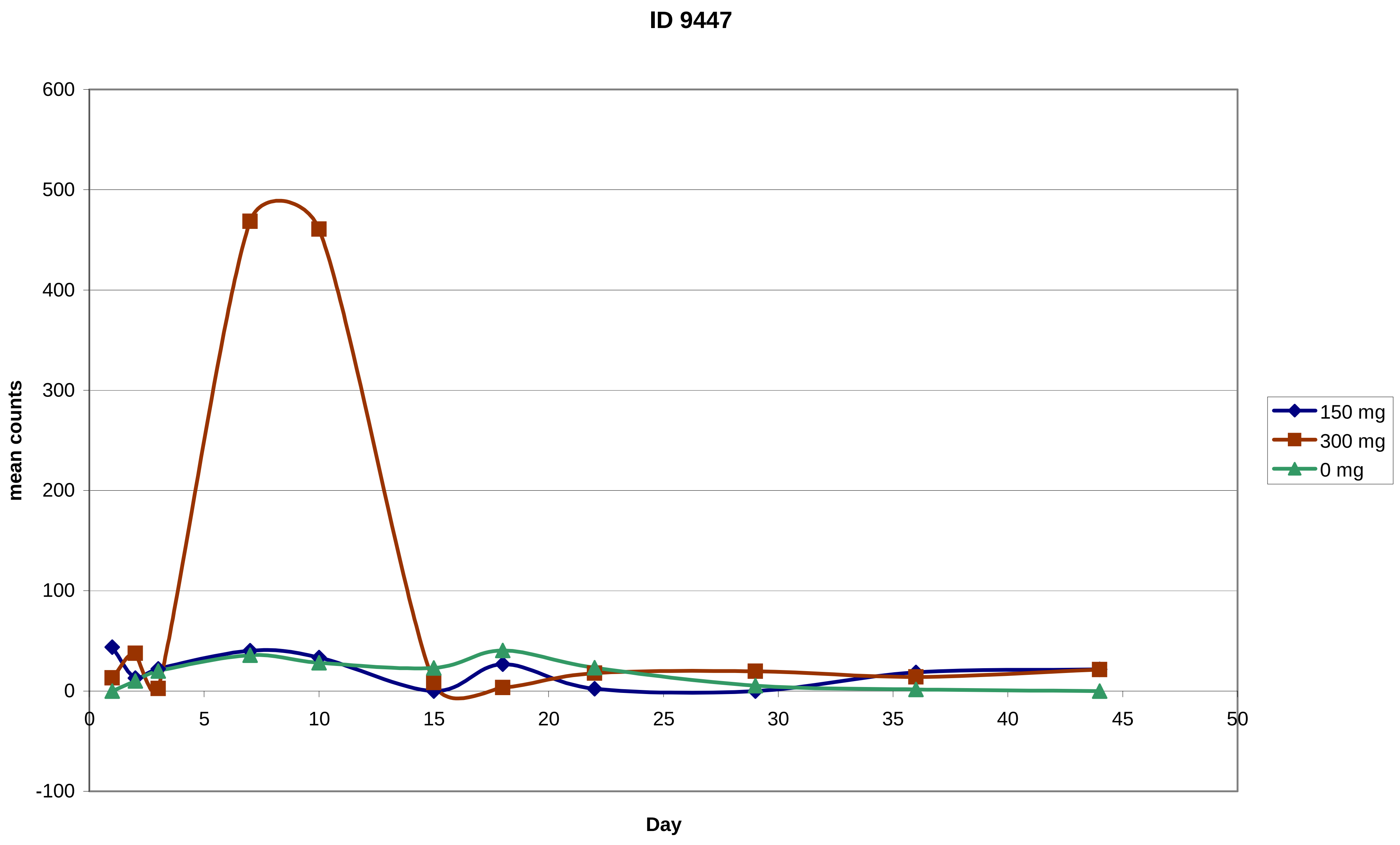
ID 9447
600
500
400
300
200
100
0
-100
mean counts
0
5
10
15
20
25
30
35
40
45
50
Day
150 mg
300 mg
0 mg

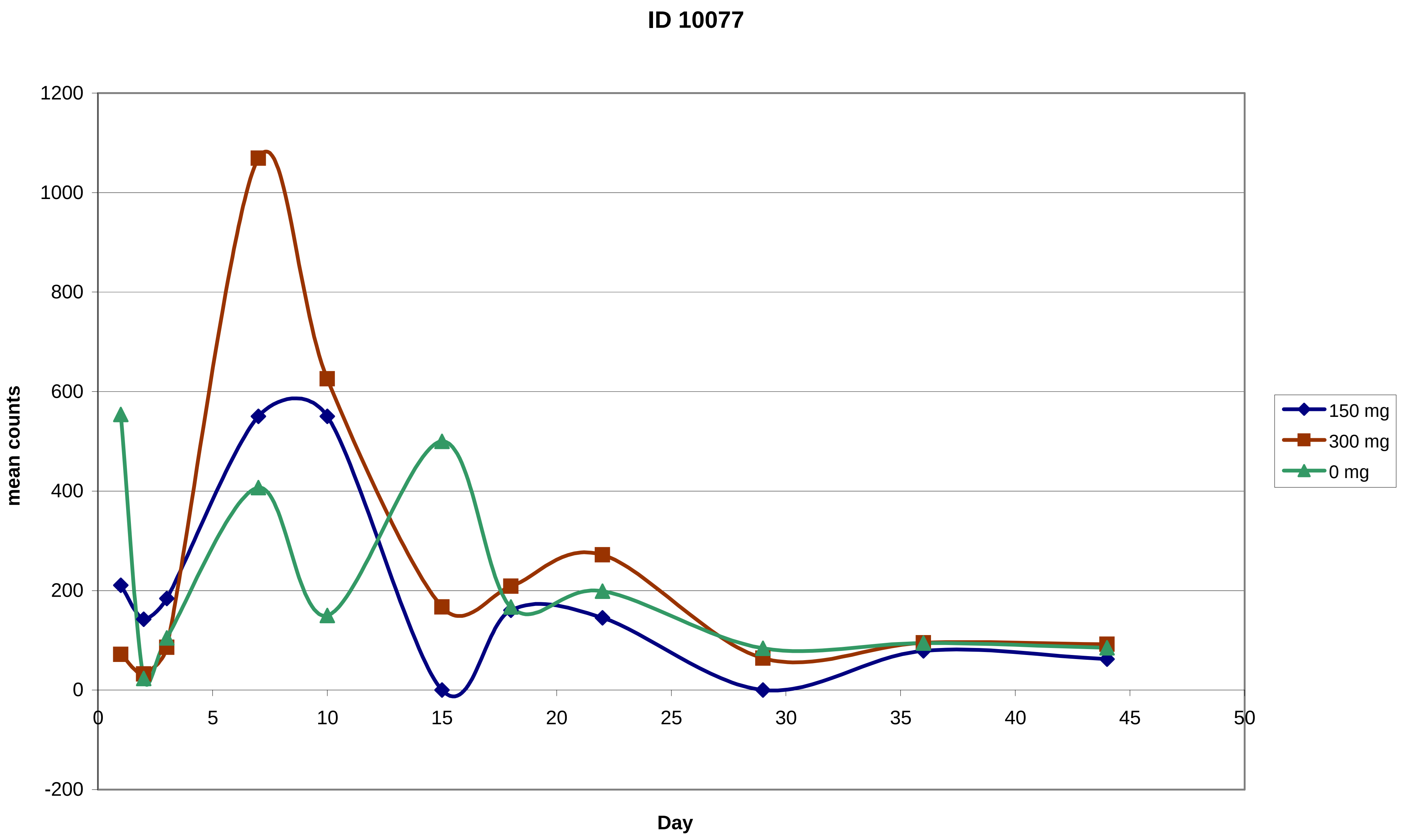

ID 10077
mean counts
1200
1000
800
600
400
200
0
-200
0
5
10
15
20
25
30
35
40
45
50
Day
150 mg
300 mg
0 mg

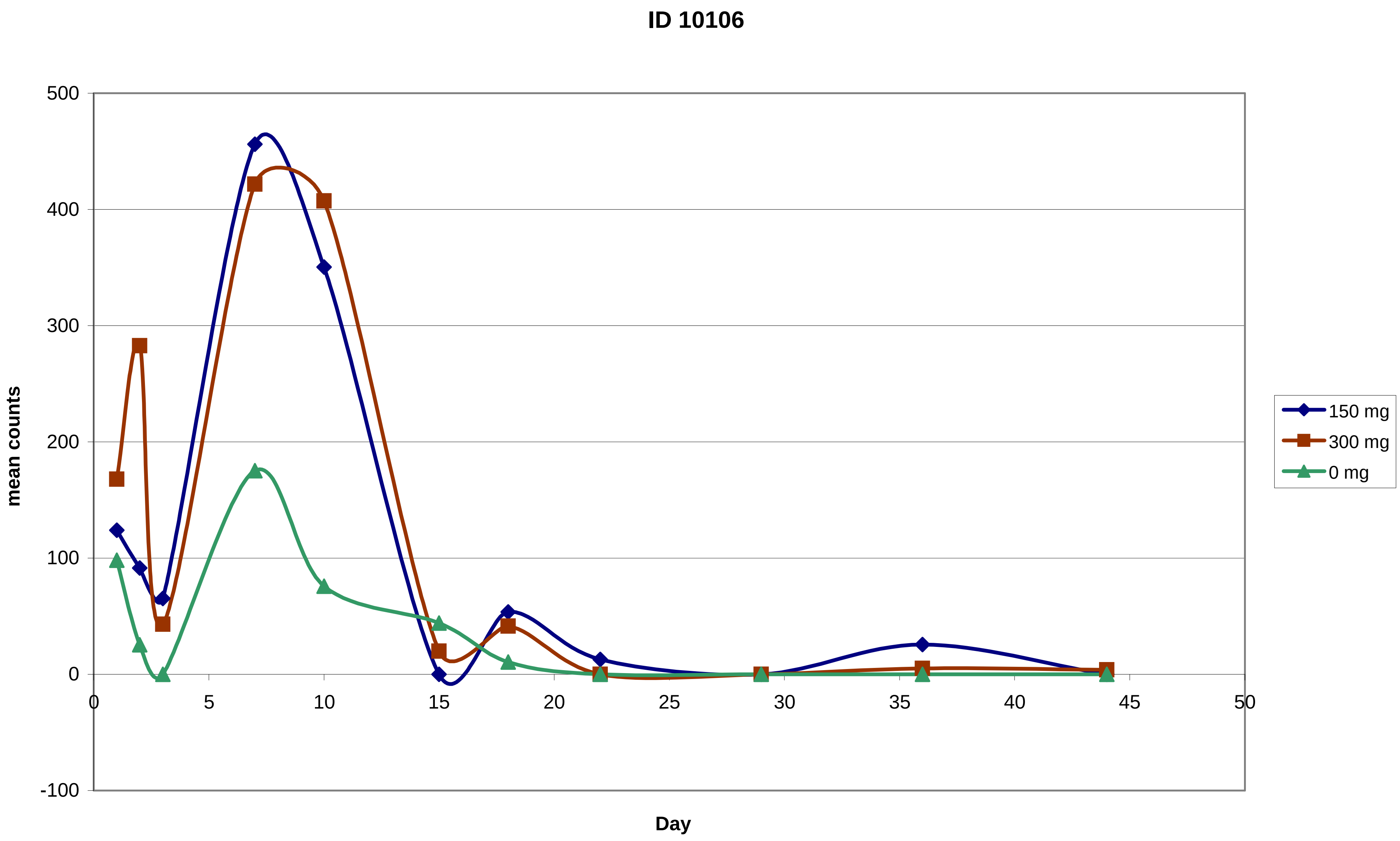
ID 10106
mean counts
500
400
300
200
100
0
-100
0
5
10
15
20
25
30
35
40
45
50
Day
150 mg
300 mg
0 mg

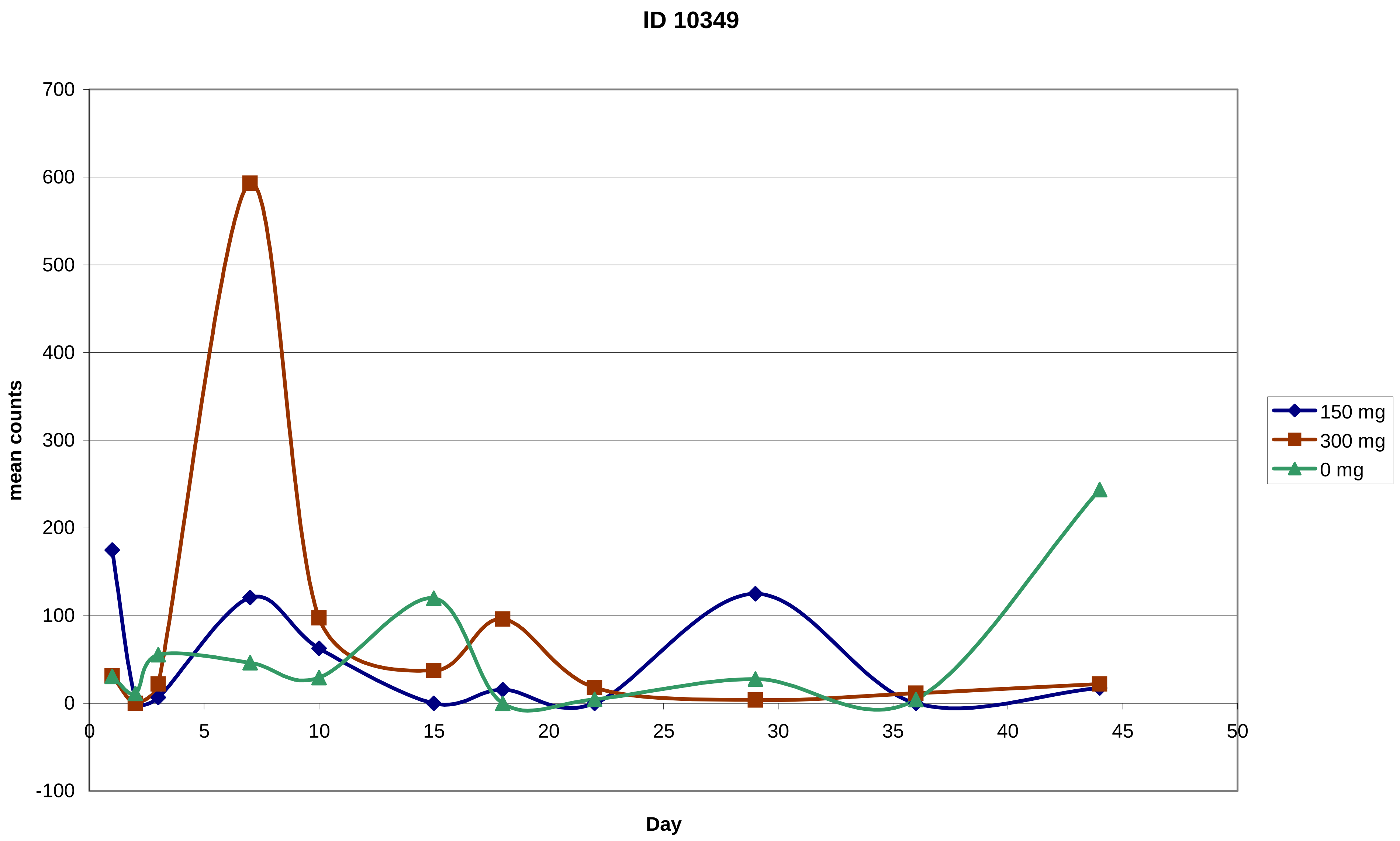
ID 10349
700
600
500
400
300
200
100
0
-100
mean counts
0
5
10
15
20
25
30
35
40
45
50
Day
150 mg
300 mg
0 mg

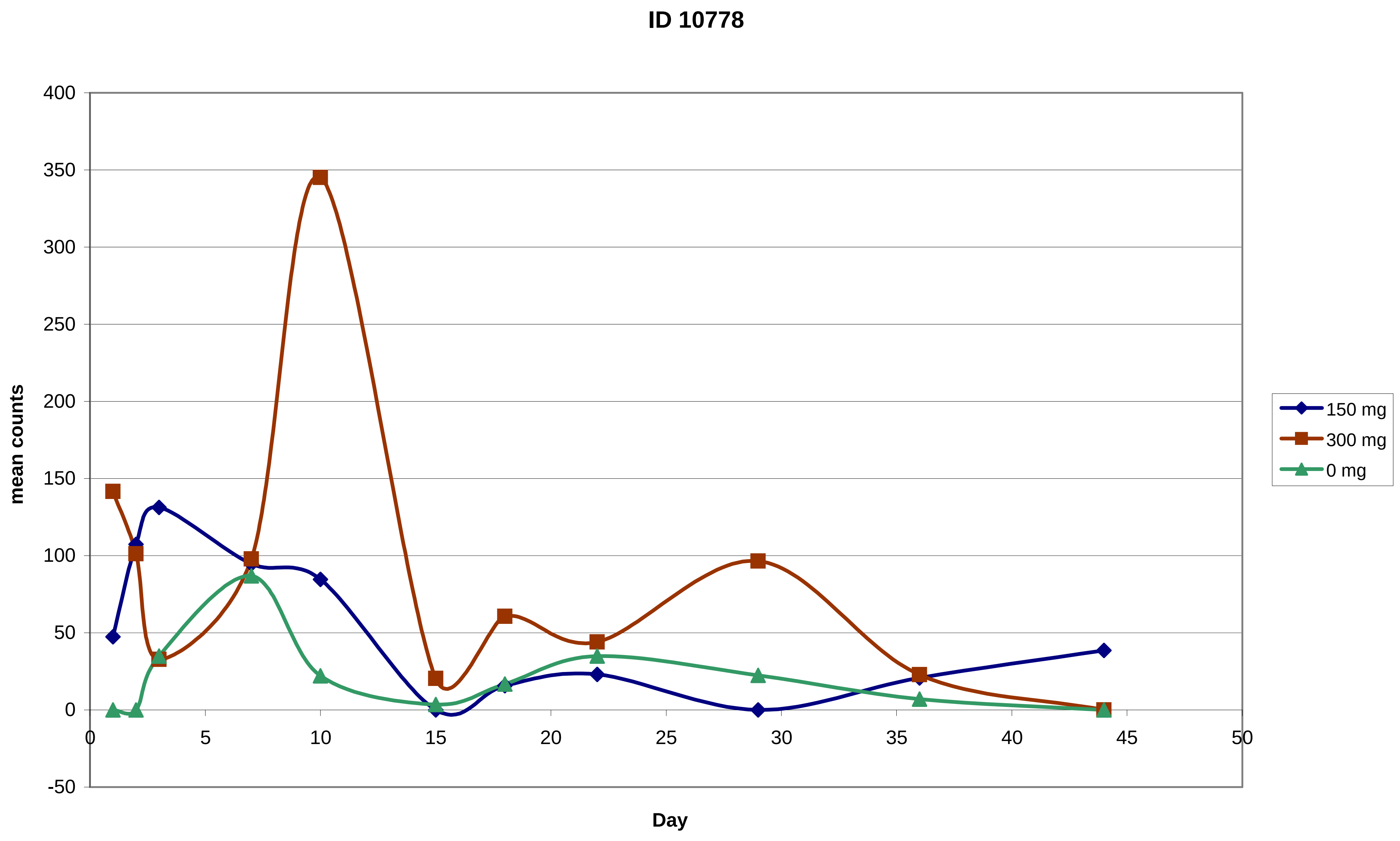
ID 10778
mean counts
Day
400
350
300
250
200
150
100
50
0
-50
0
5
10
15
20
25
30
35
40
45
50
150 mg
300 mg
0 mg

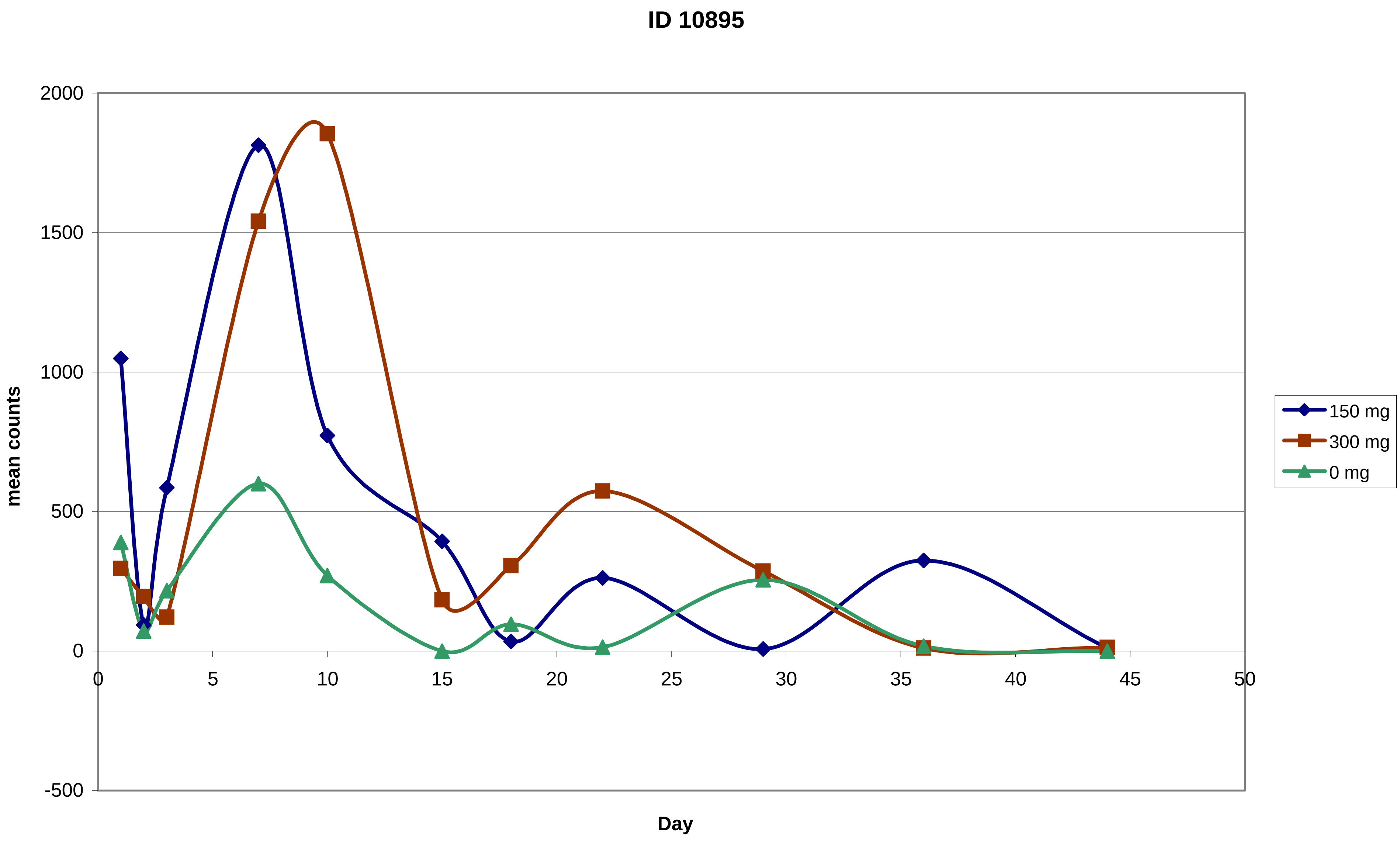

ID 10895
2000
1500
1000
500
0
-500
mean counts
0
5
10
15
20
25
30
35
40
45
50
Day
150 mg
300 mg
0 mg

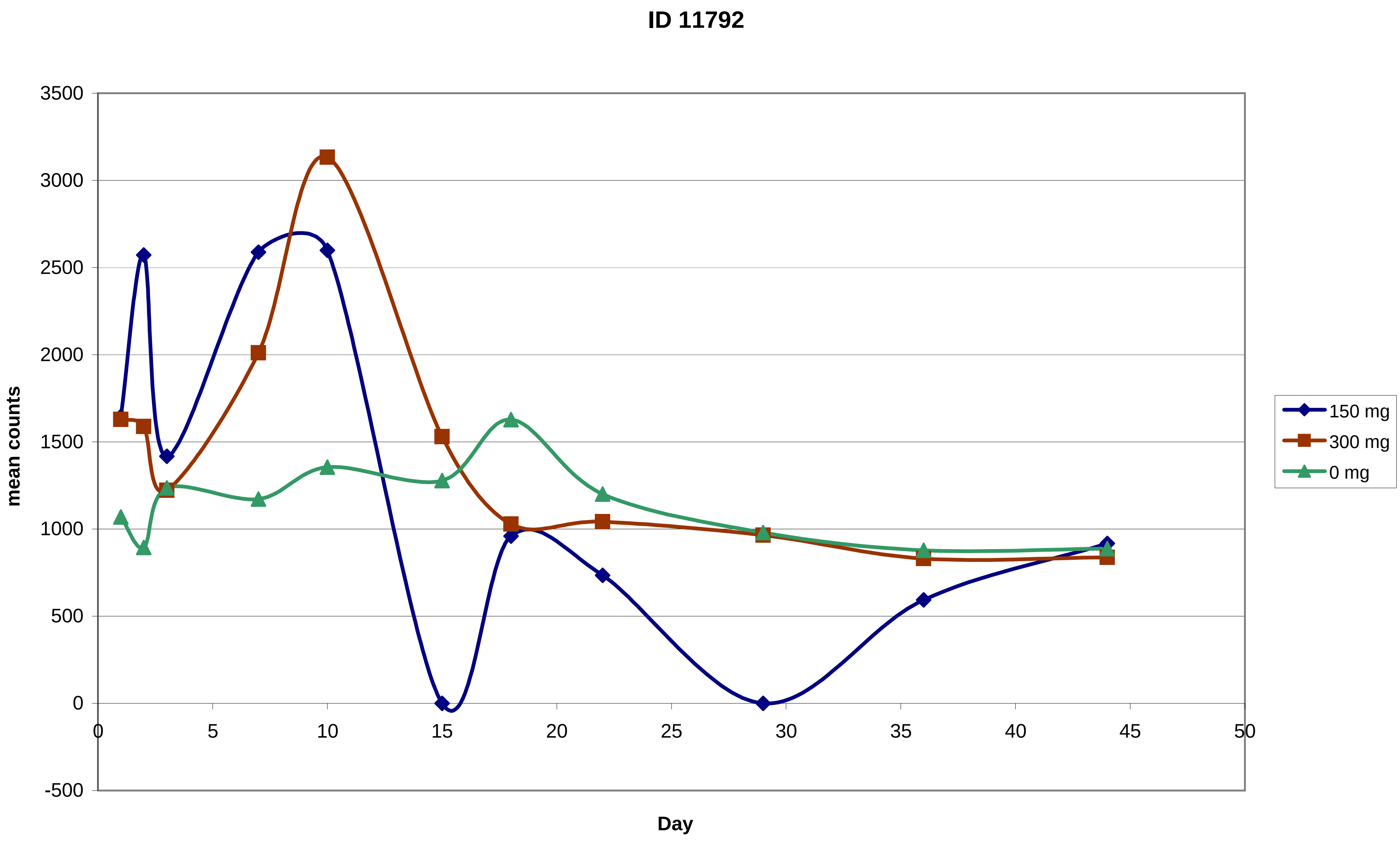
ID 11792
mean counts
3500
3000
2500
2000
1500
1000
500
0
-500
0
5
10
15
20
25
30
35
40
45
50
Day
150 mg
300 mg
0 mg

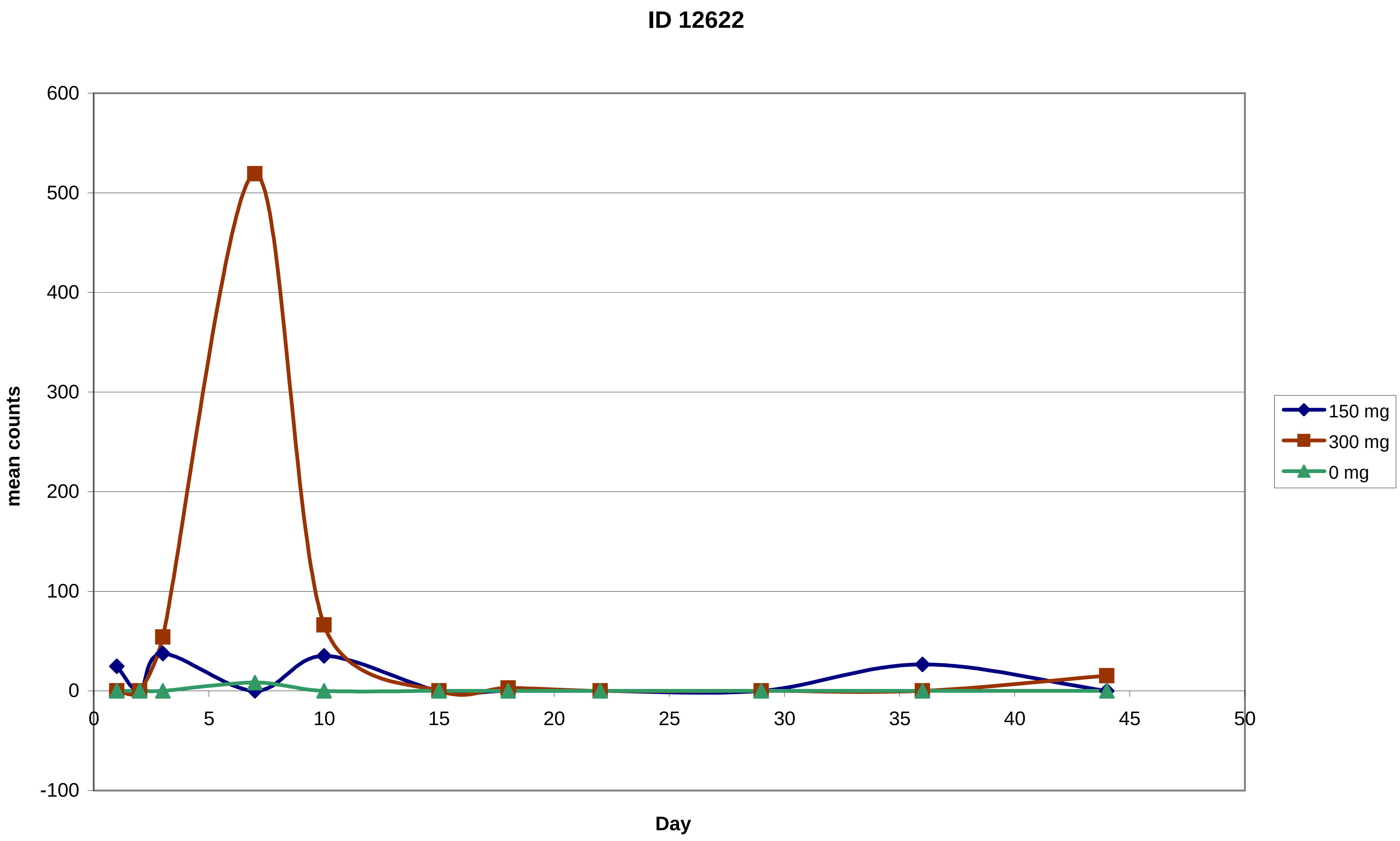

ID 12622
mean counts
600
500
400
300
200
100
0
-100
0
5
10
15
20
25
30
35
40
45
50
Day
150 mg
300 mg
0 mg

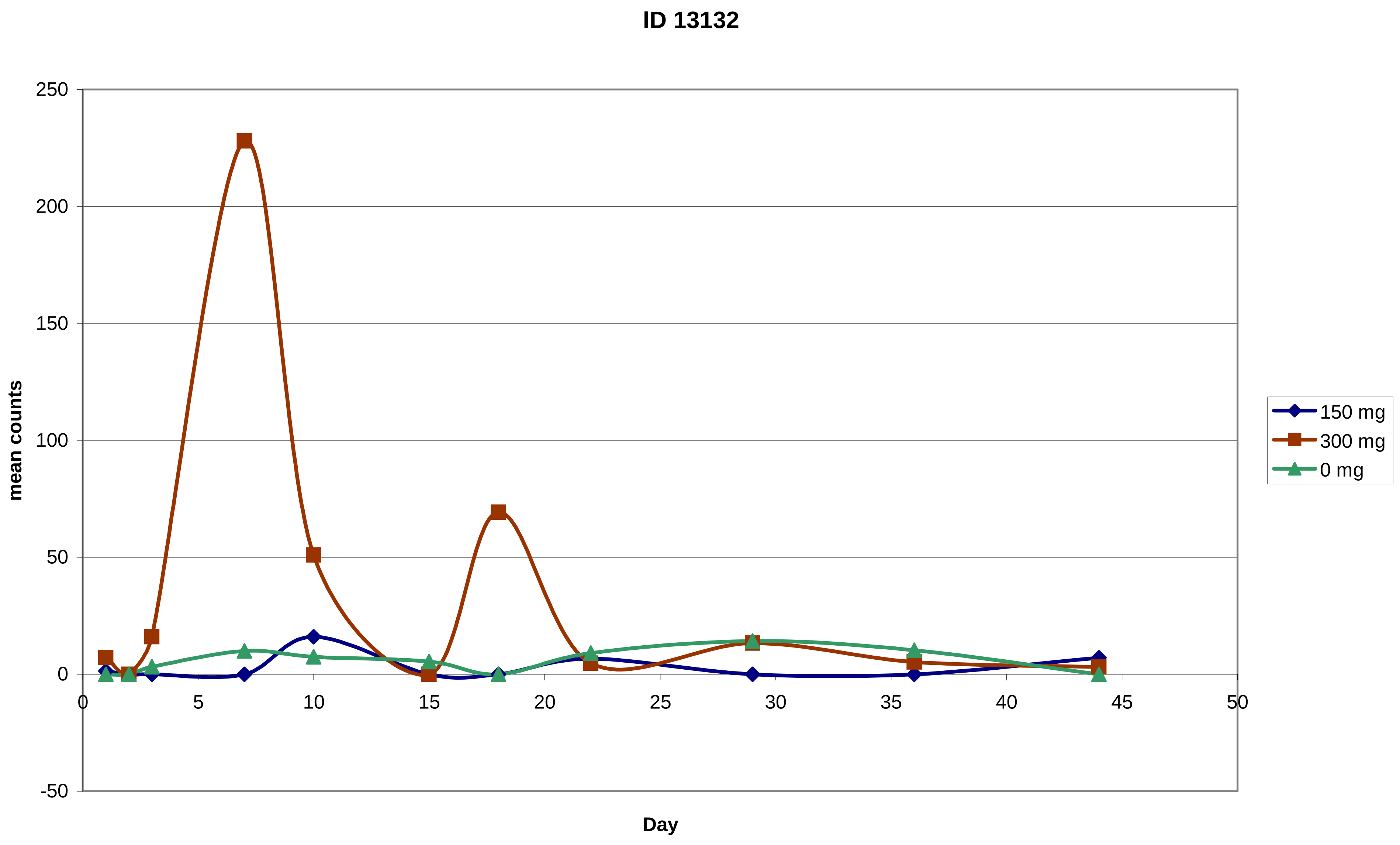
ID 13132
mean counts
250
200
150
100
50
0
-50
0
5
10
15
20
25
30
35
40
45
50
Day
150 mg
300 mg
0 mg

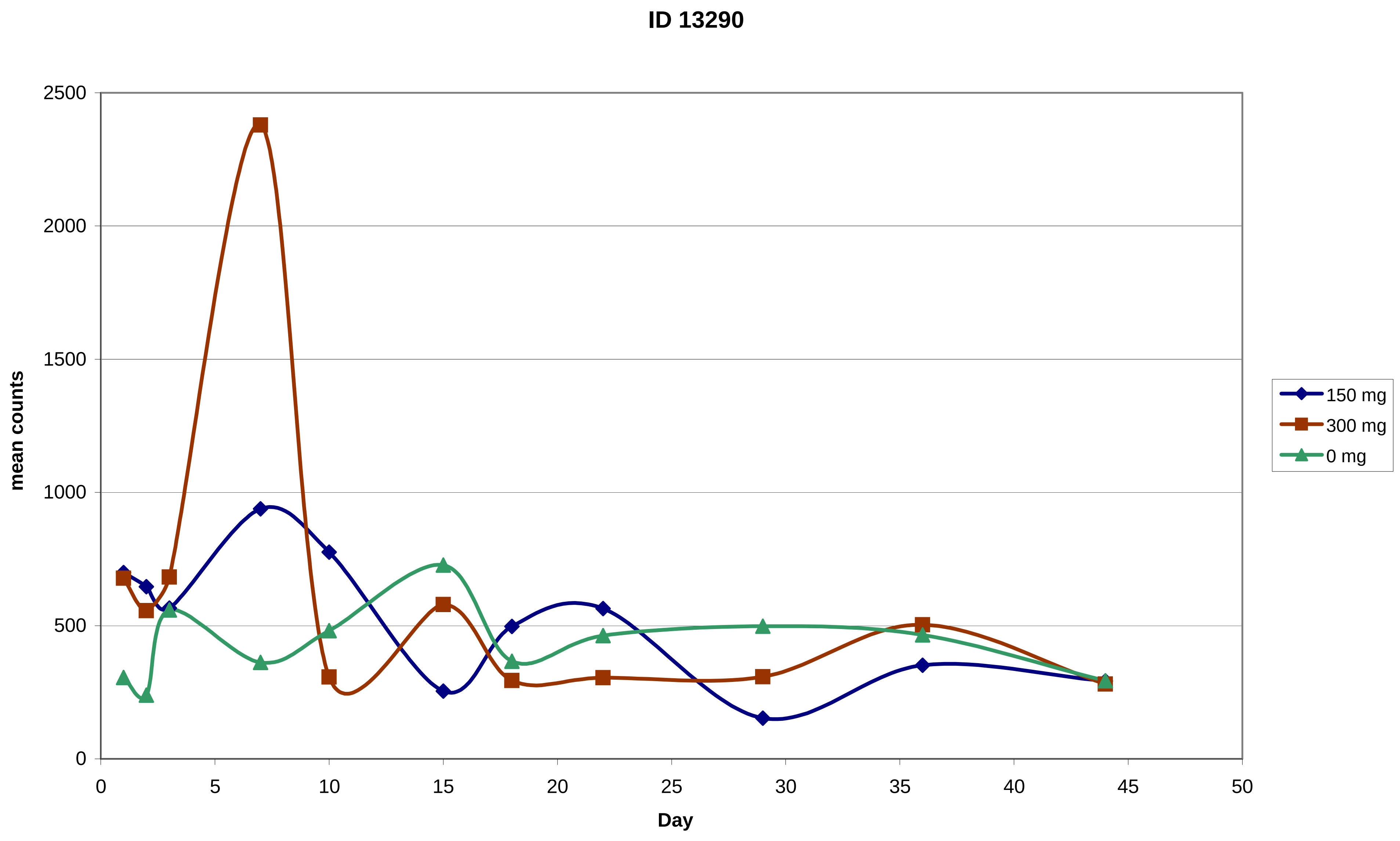

ID 13290
mean counts
2500
2000
1500
1000
500
0
0
5
10
15
20
25
30
35
40
45
50
Day
150 mg
300 mg
0 mg

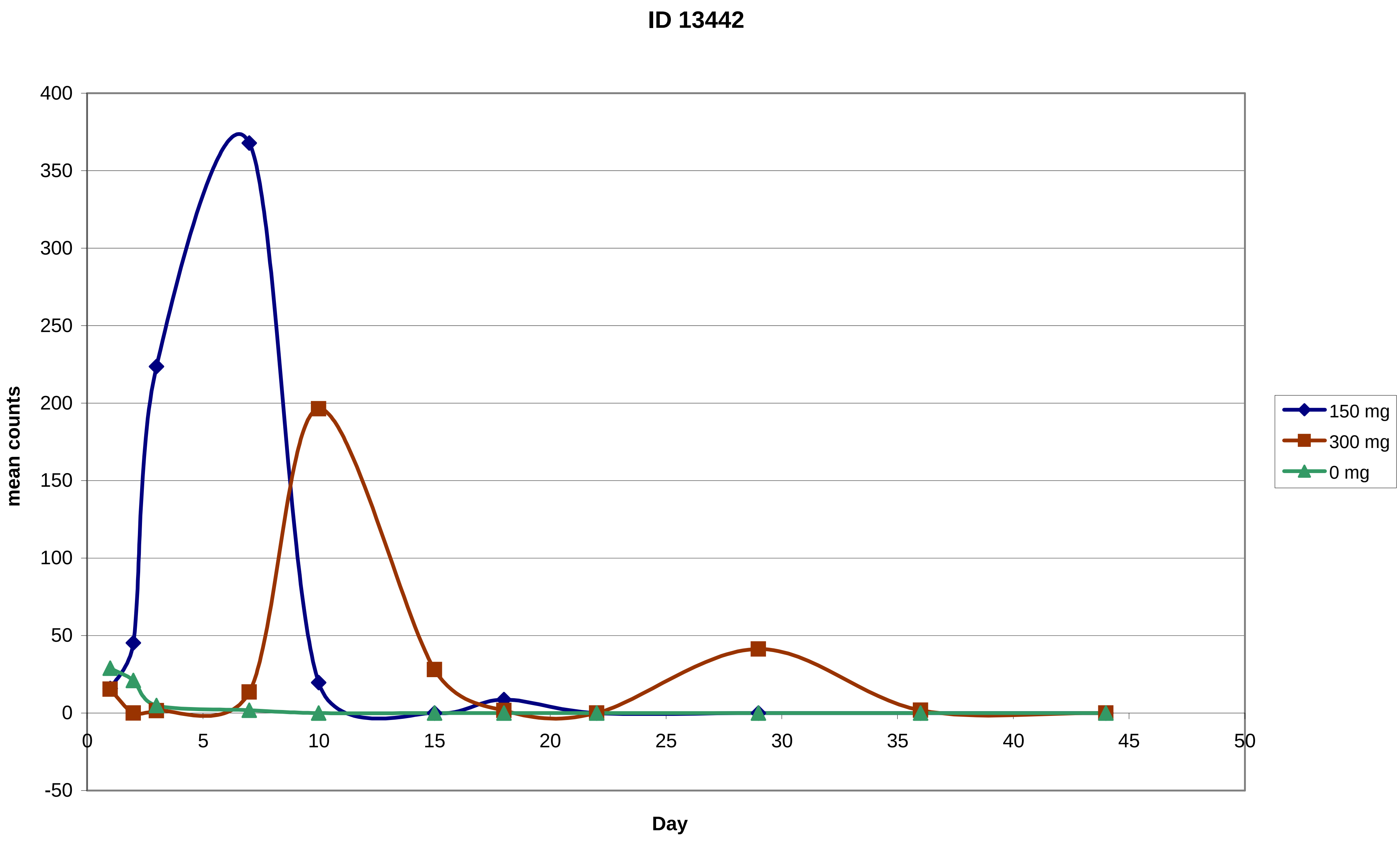

ID 13442
mean counts
400
350
300
250
200
150
100
50
0
-50
0
5
10
15
20
25
30
35
40
45
50
Day
150 mg
300 mg
0 mg

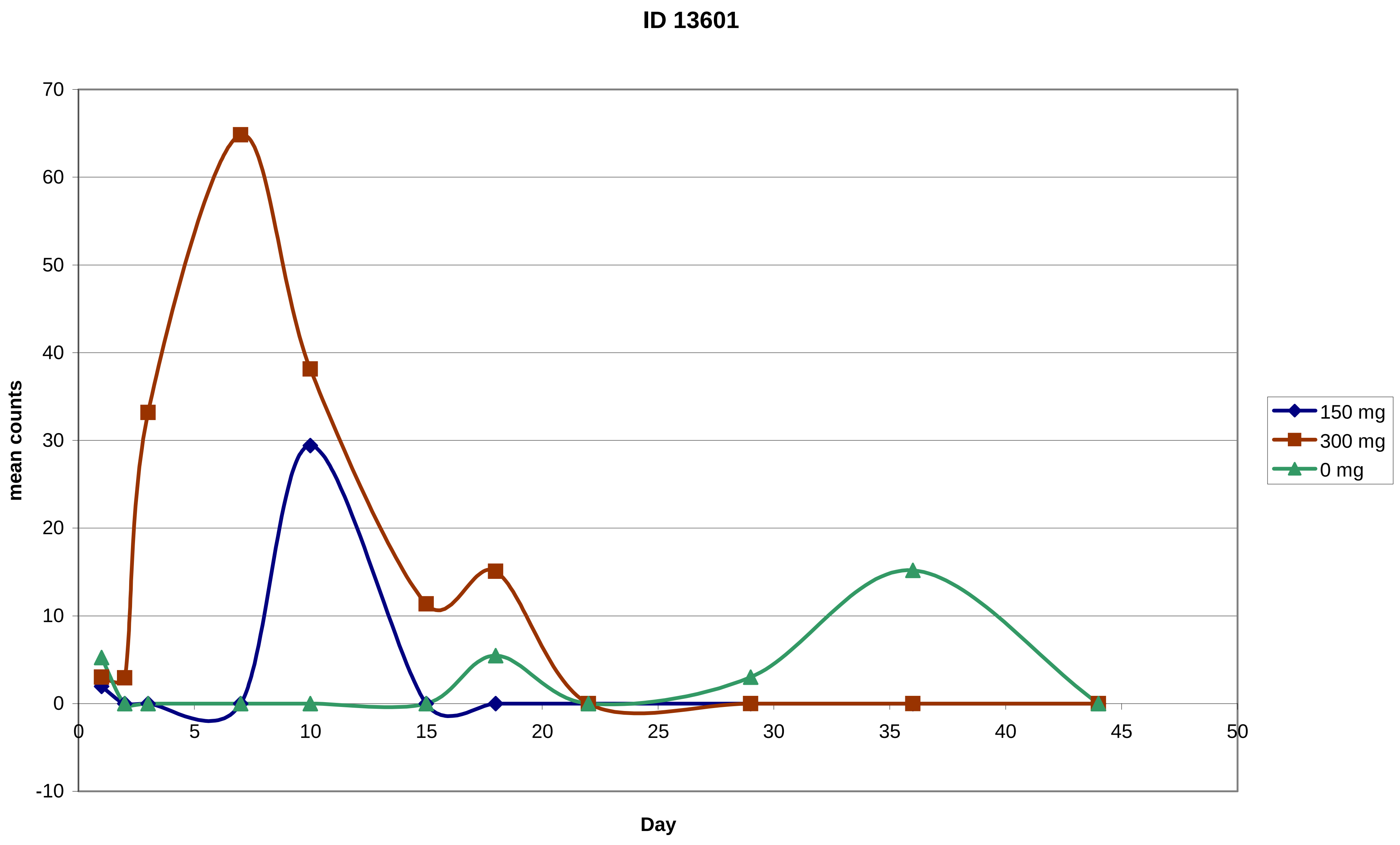

ID 13601
mean counts
70
60
50
40
30
20
10
0
-10
0
5
10
15
20
25
30
35
40
45
50
Day
150 mg
300 mg
0 mg

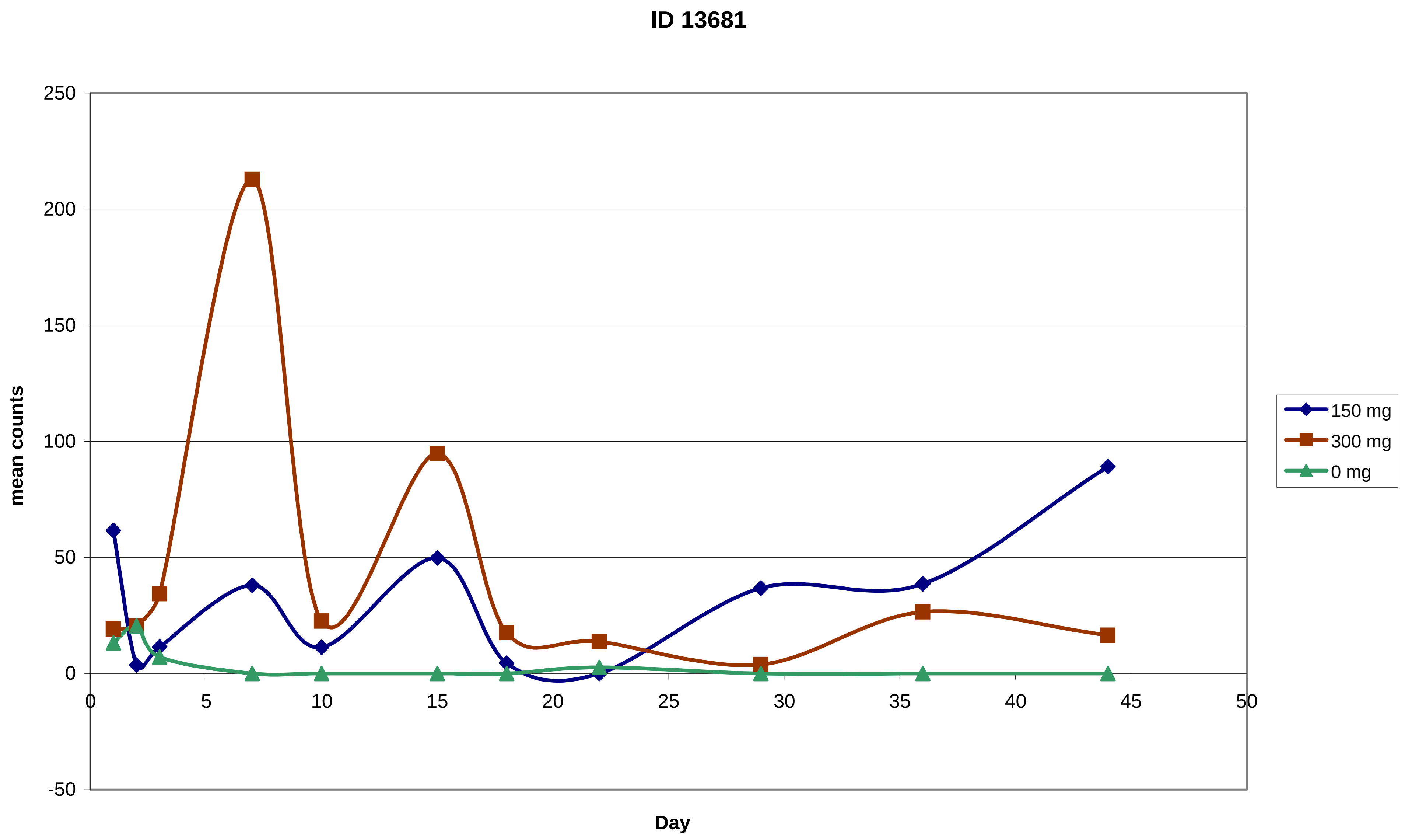
ID 13681
mean counts
250
200
150
100
50
0
-50
0
5
10
15
20
25
30
35
40
45
50
Day
150 mg
300 mg
0 mg

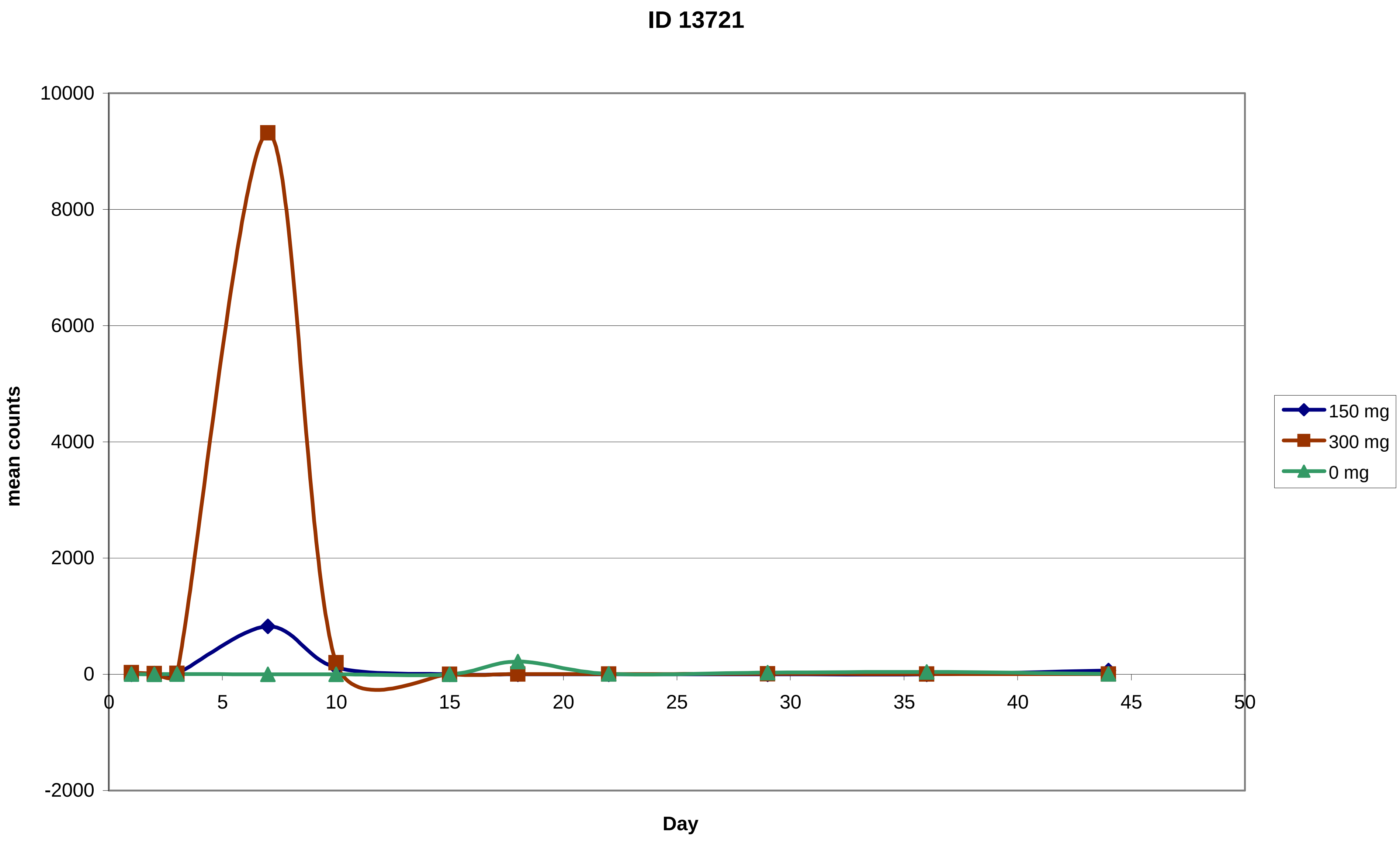
ID 13721
mean counts
Day
10000
8000
6000
4000
2000
0
-2000
0
5
10
15
20
25
30
35
40
45
50
150 mg
300 mg
0 mg

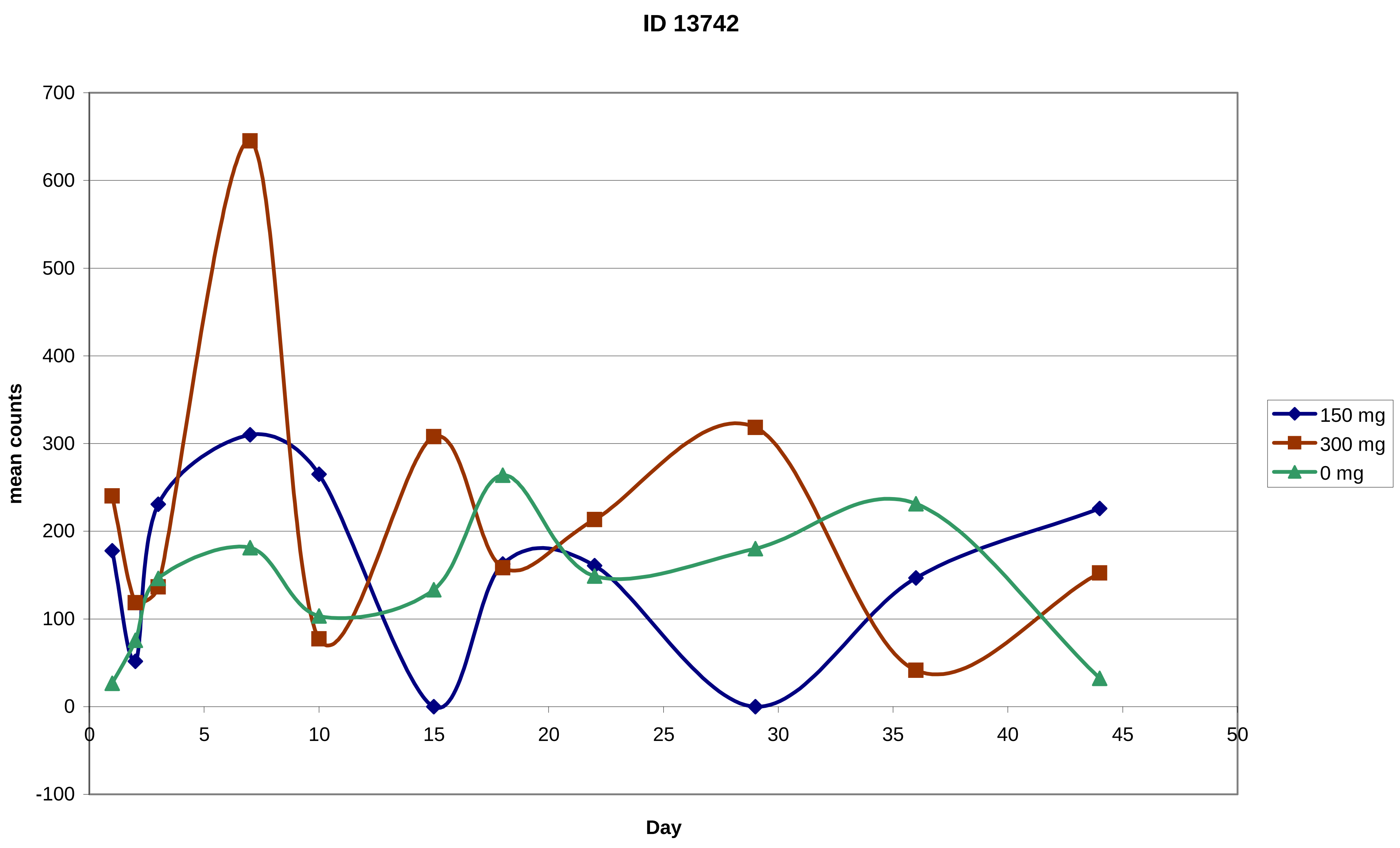
ID 13742
mean counts
700
600
500
400
300
200
100
0
-100
0
5
10
15
20
25
30
35
40
45
50
Day
150 mg
300 mg
0 mg

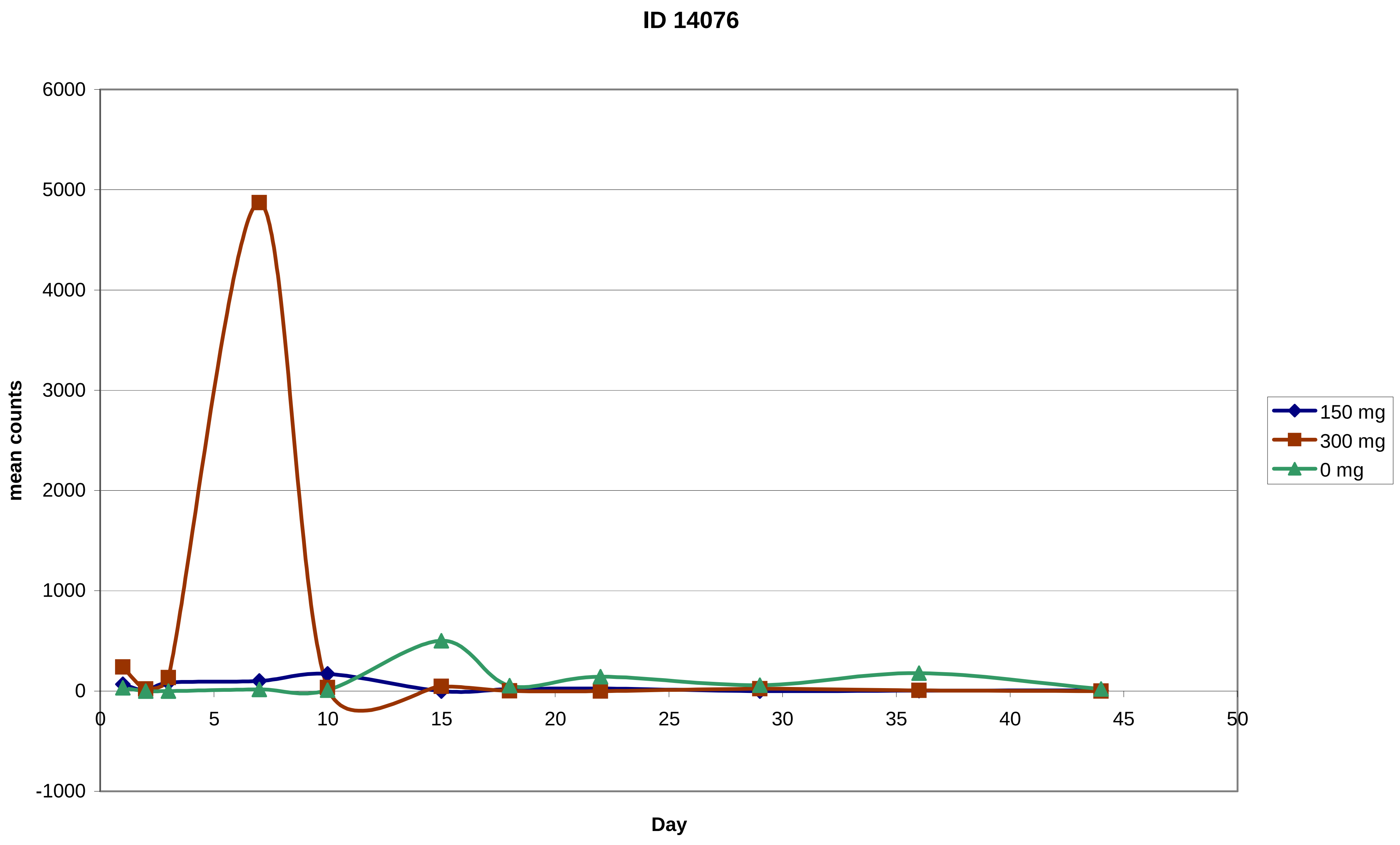
ID 14076
mean counts
Day
6000
5000
4000
3000
2000
1000
0
-1000
0
5
10
15
20
25
30
35
40
45
50
150 mg
300 mg
0 mg

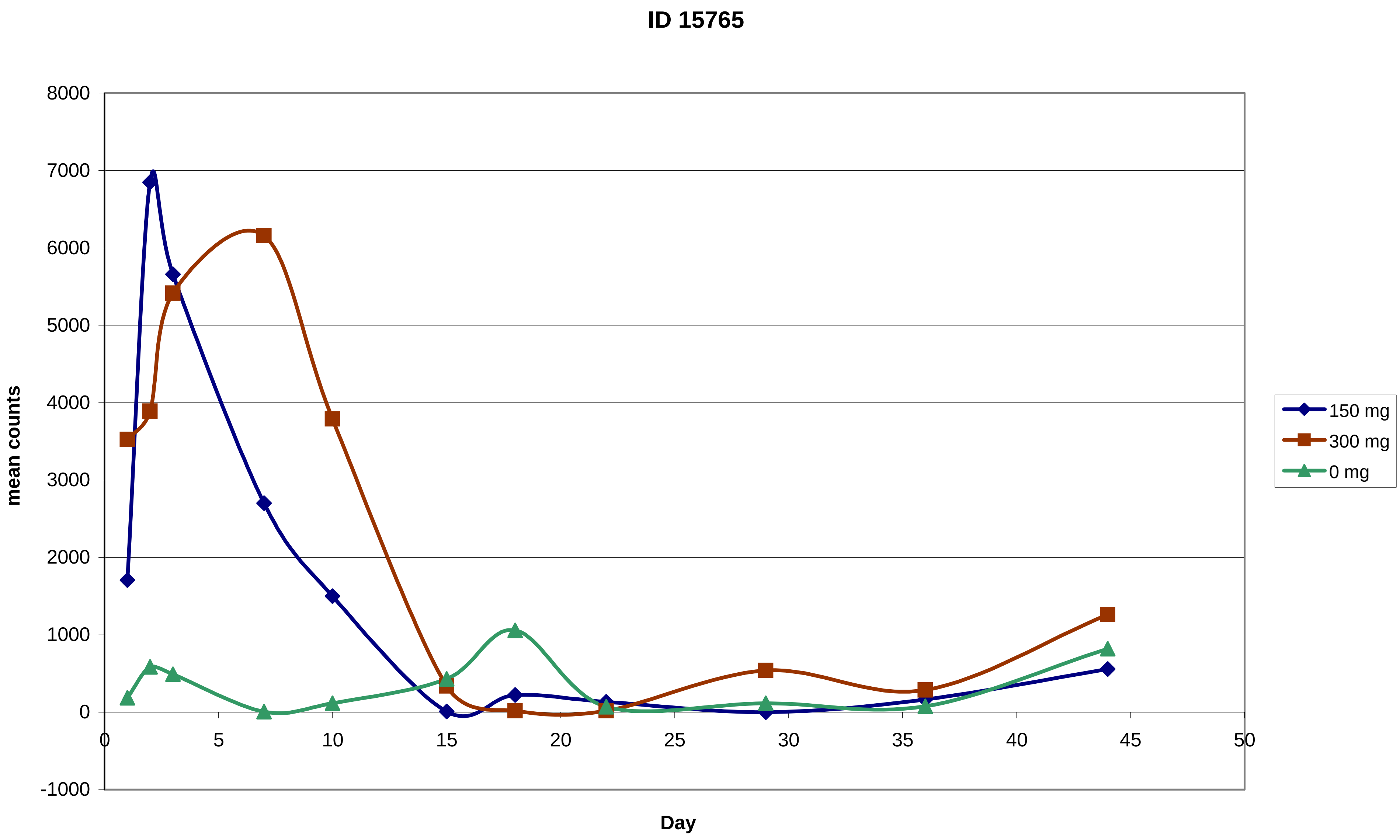
ID 15765
mean counts
8000
7000
6000
5000
4000
3000
2000
1000
0
-1000
0
5
10
15
20
25
30
35
40
45
50
Day
150 mg
300 mg
0 mg

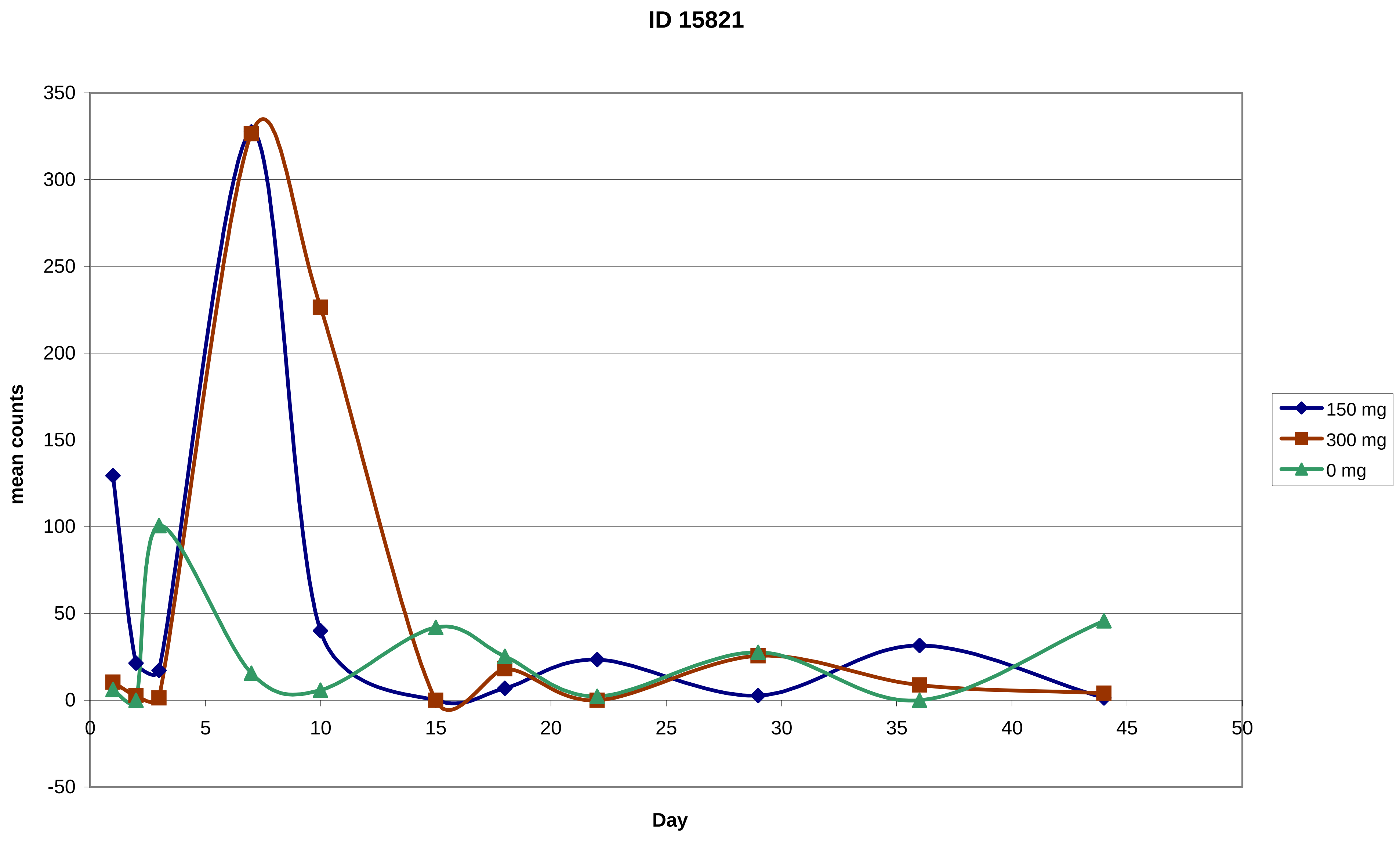

ID 15821
mean counts
350
300
250
200
150
100
50
0
-50
0
5
10
15
20
25
30
35
40
45
50
Day
150 mg
300 mg
0 mg

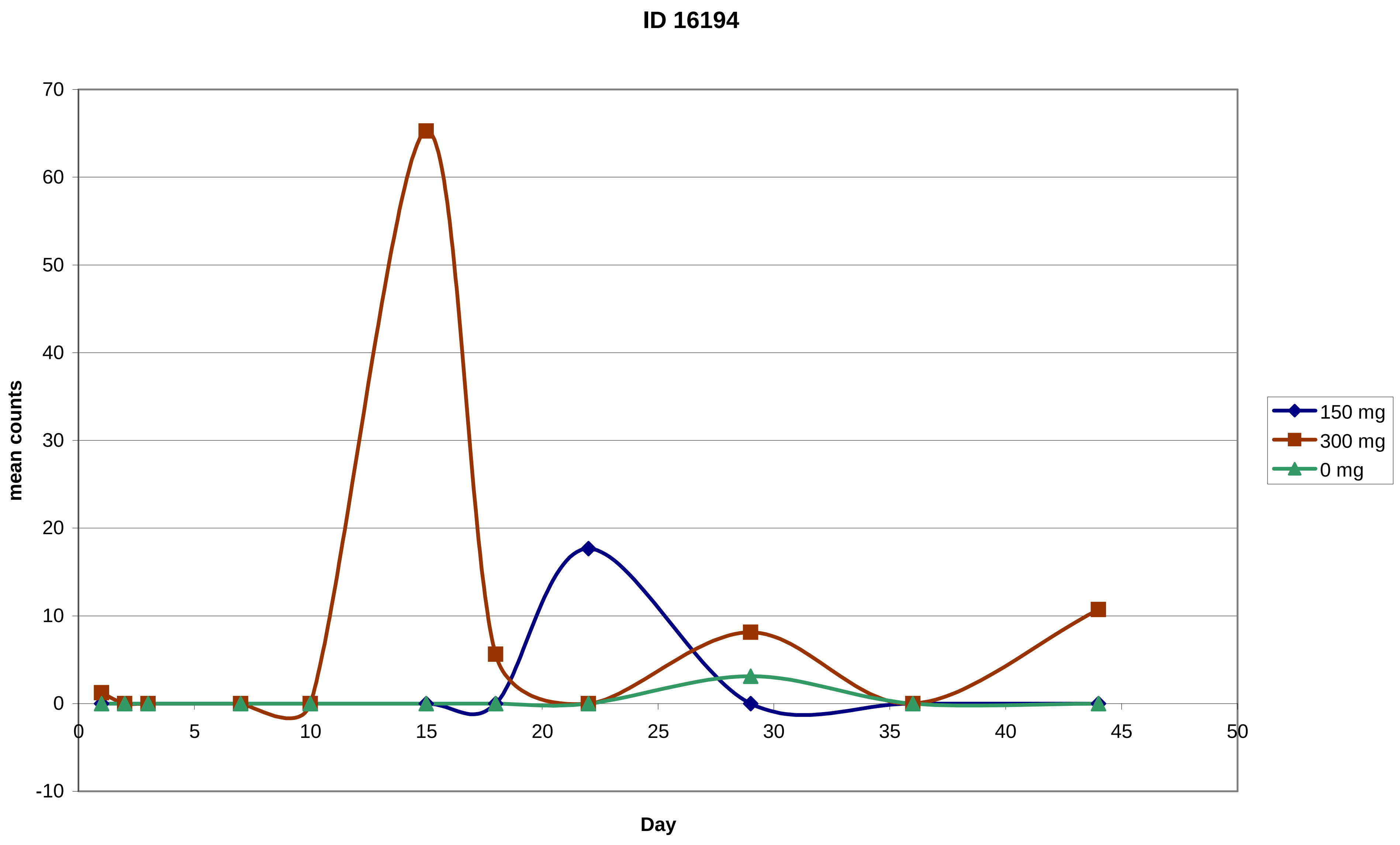

ID 16194
mean counts
70
60
50
40
30
20
10
0
-10
0
5
10
15
20
25
30
35
40
45
50
Day
150 mg
300 mg
0 mg

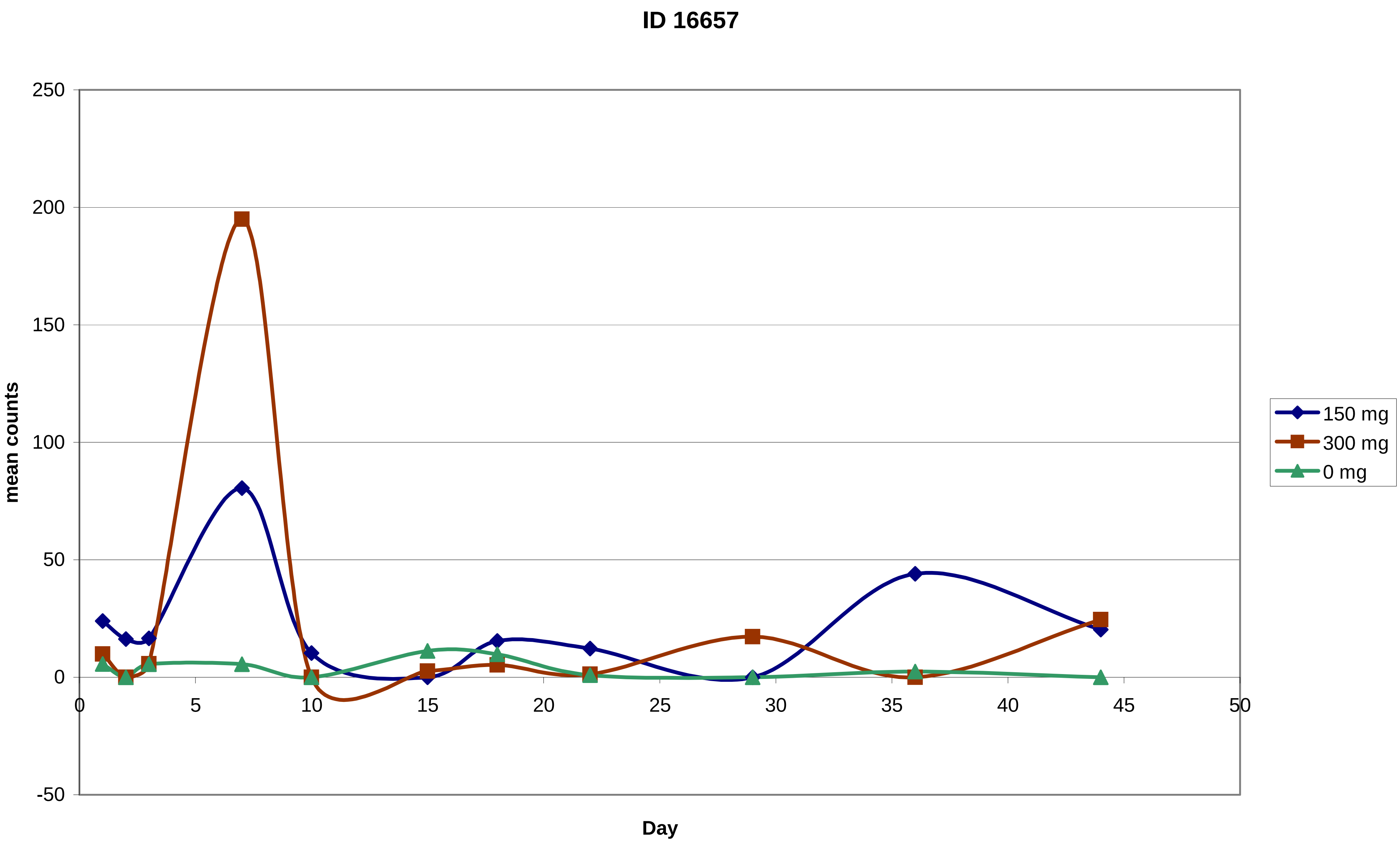
ID 16657
mean counts
250
200
150
100
50
0
-50
0
5
10
15
20
25
30
35
40
45
50
Day
150 mg
300 mg
0 mg

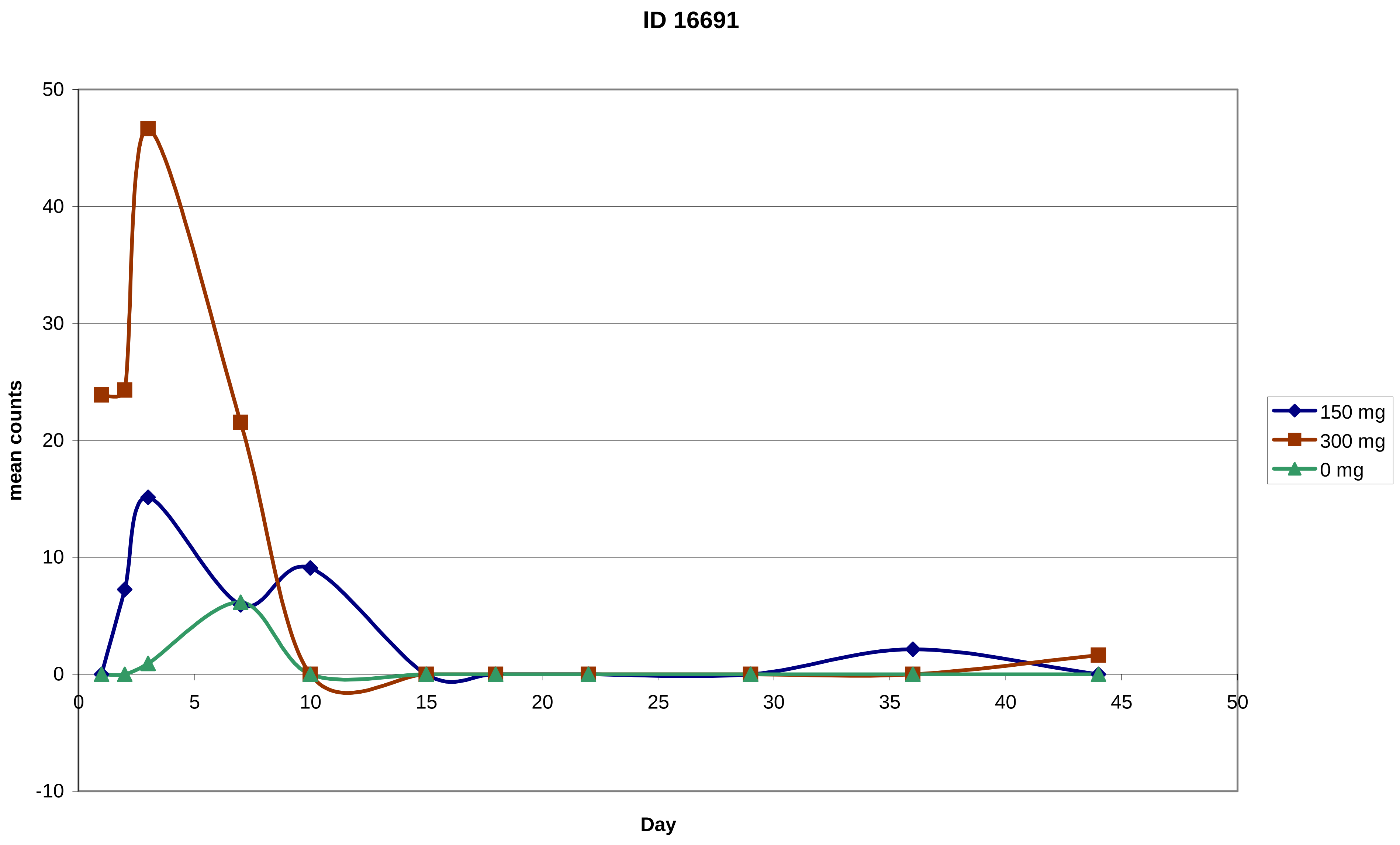
ID 16691
mean counts
Day
50
40
30
20
10
0
-10
0
5
10
15
20
25
30
35
40
45
50
150 mg
300 mg
0 mg

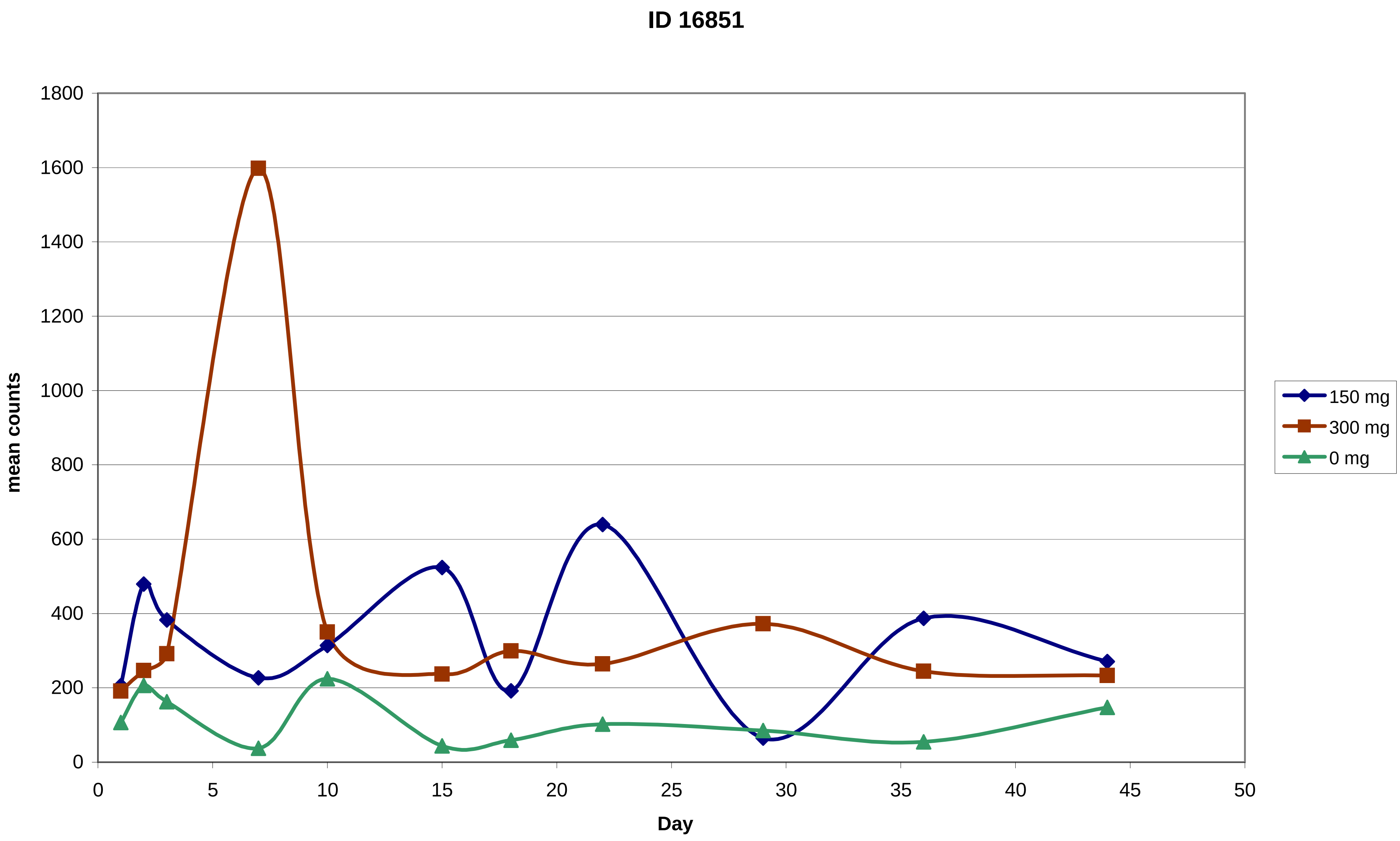

ID 16851
mean counts
1800
1600
1400
1200
1000
800
600
400
200
0
0
5
10
15
20
25
30
35
40
45
50
Day
150 mg
300 mg
0 mg

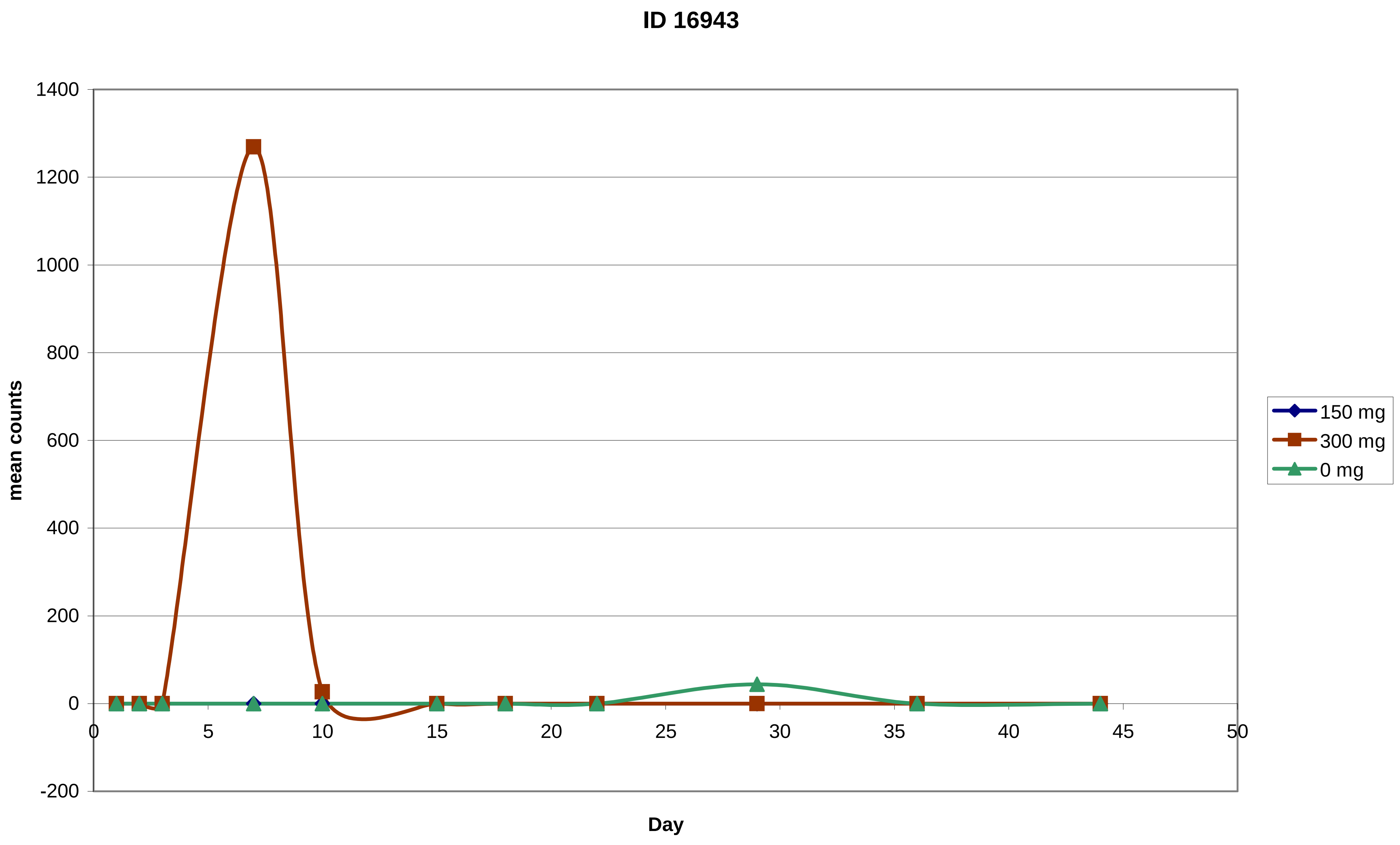
ID 16943
mean counts
Day
1400
1200
1000
800
600
400
200
0
-200
0
5
10
15
20
25
30
35
40
45
50
150 mg
300 mg
0 mg

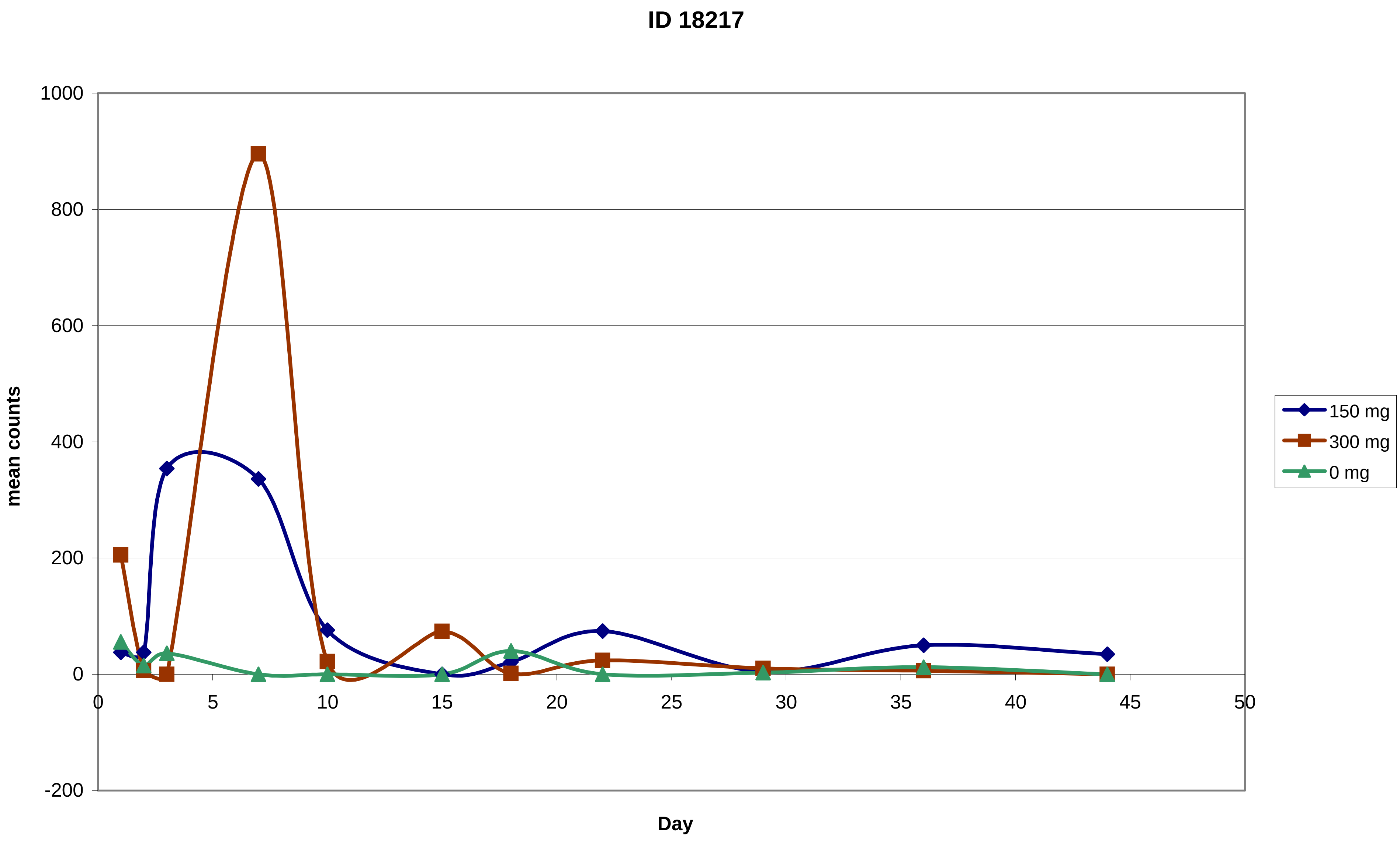

ID 18217
mean counts
1000
800
600
400
200
0
-200
0
5
10
15
20
25
30
35
40
45
50
Day
150 mg
300 mg
0 mg

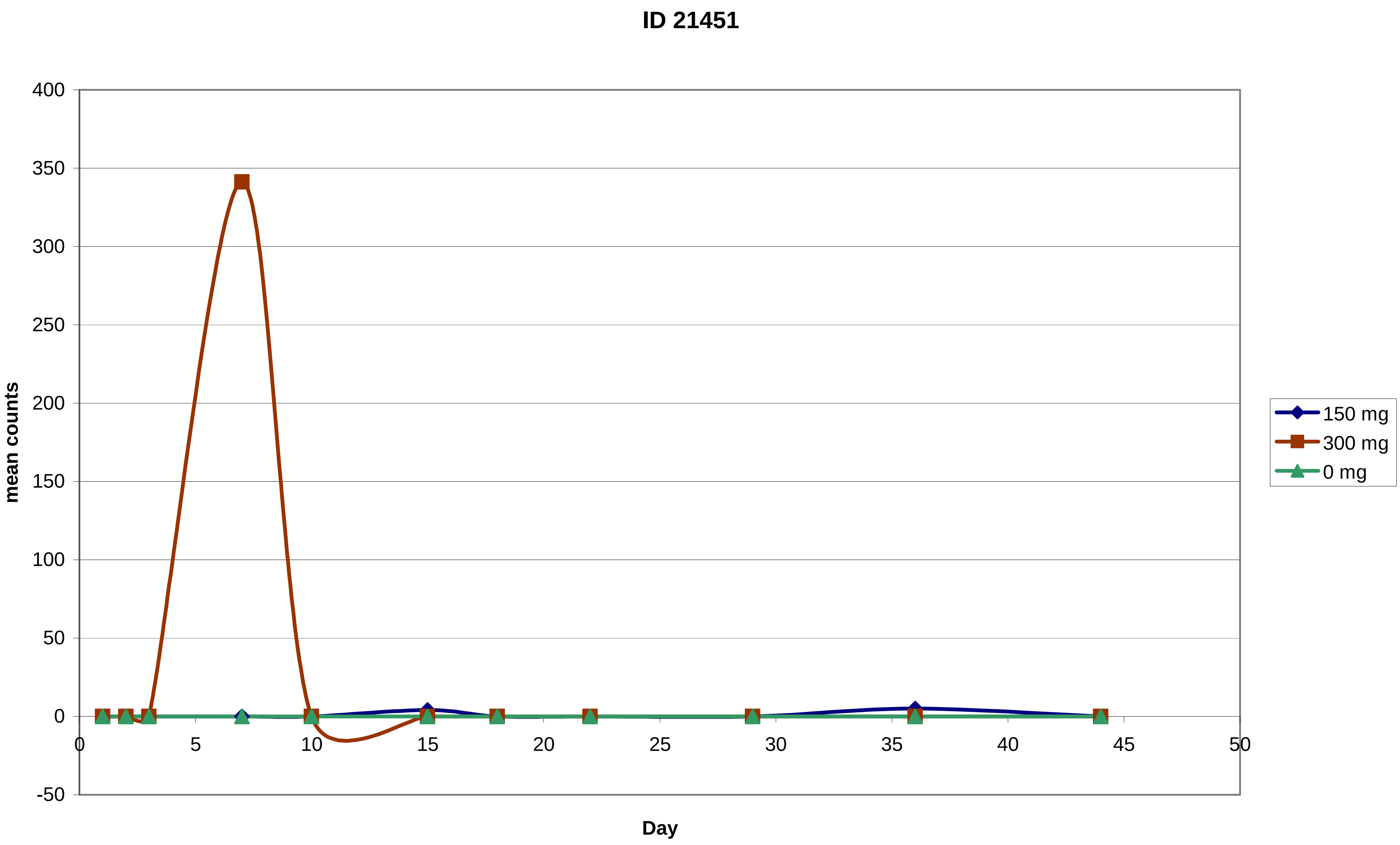

ID 21451
400
350
300
250
200
150
100
50
0
-50
mean counts
0
5
10
15
20
25
30
35
40
45
50
Day
150 mg
300 mg
0 mg

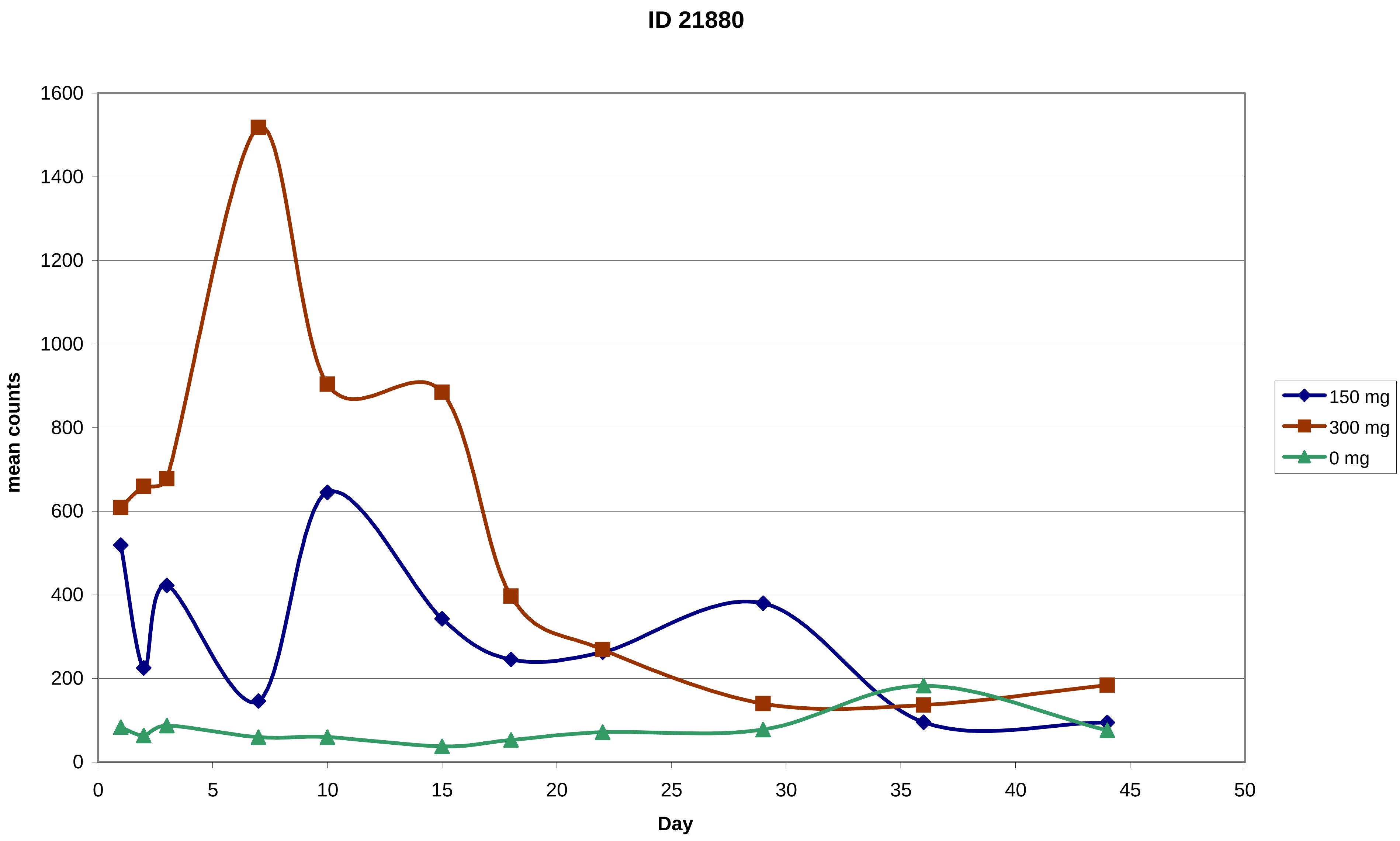
ID 21880
mean counts
1600
1400
1200
1000
800
600
400
200
0
0
5
10
15
20
25
30
35
40
45
50
Day
150 mg
300 mg
0 mg

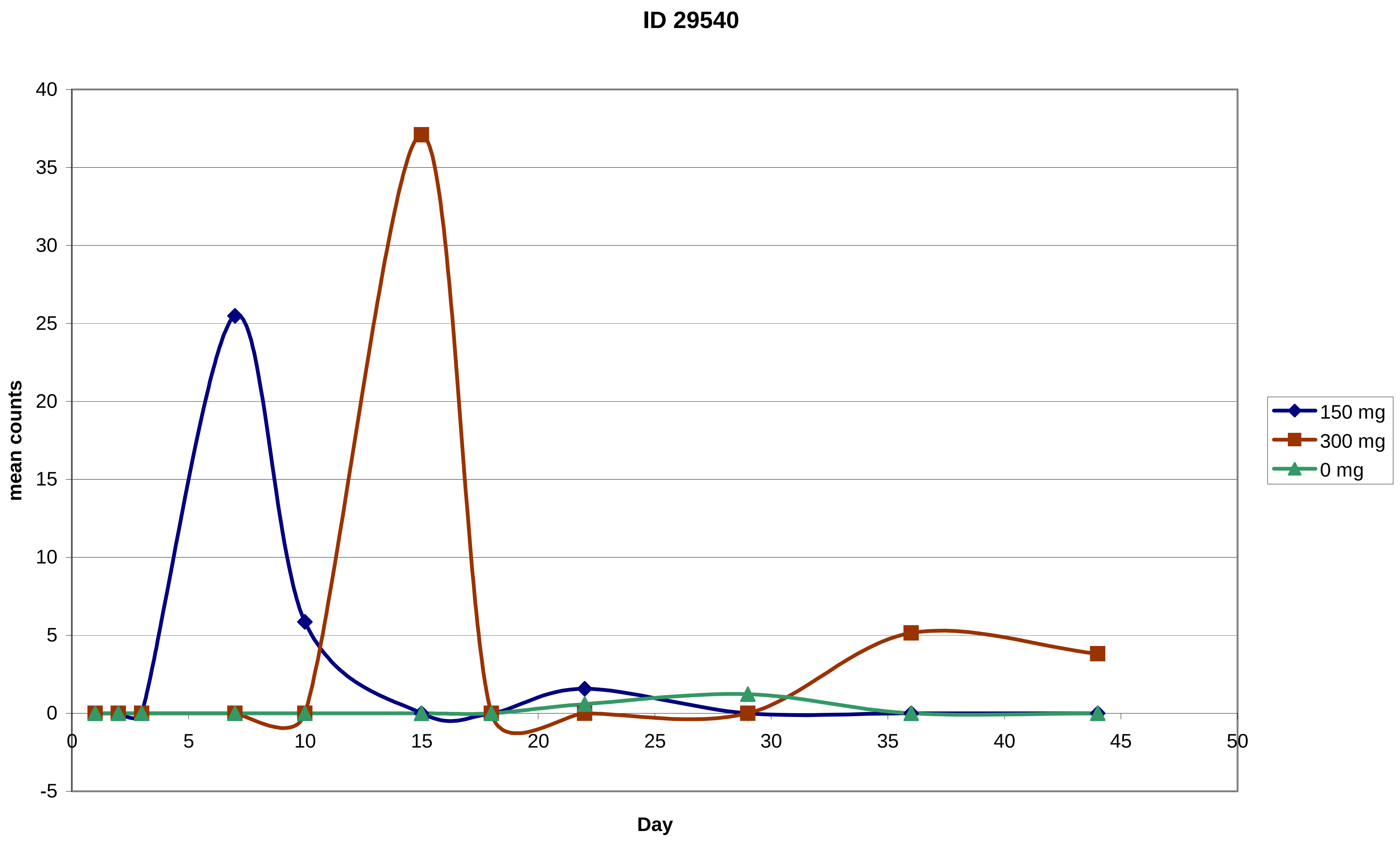
ID 29540
mean counts
Day
40
35
30
25
20
15
10
5
0
-5
0
5
10
15
20
25
30
35
40
45
50
150 mg
300 mg
0 mg

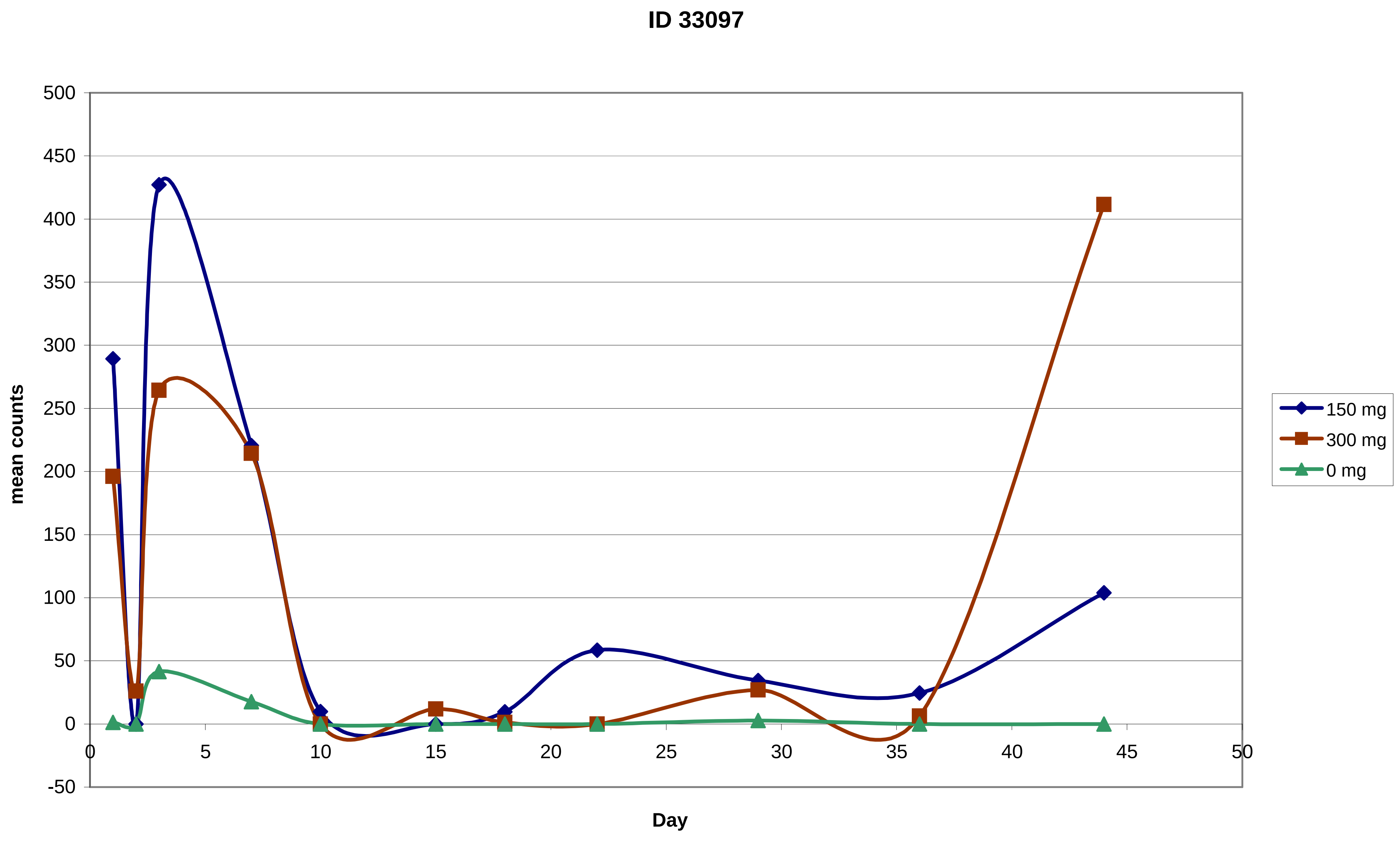

ID 33097
mean counts
500
450
400
350
300
250
200
150
100
50
0
-50
0
5
10
15
20
25
30
35
40
45
50
Day
150 mg
300 mg
0 mg

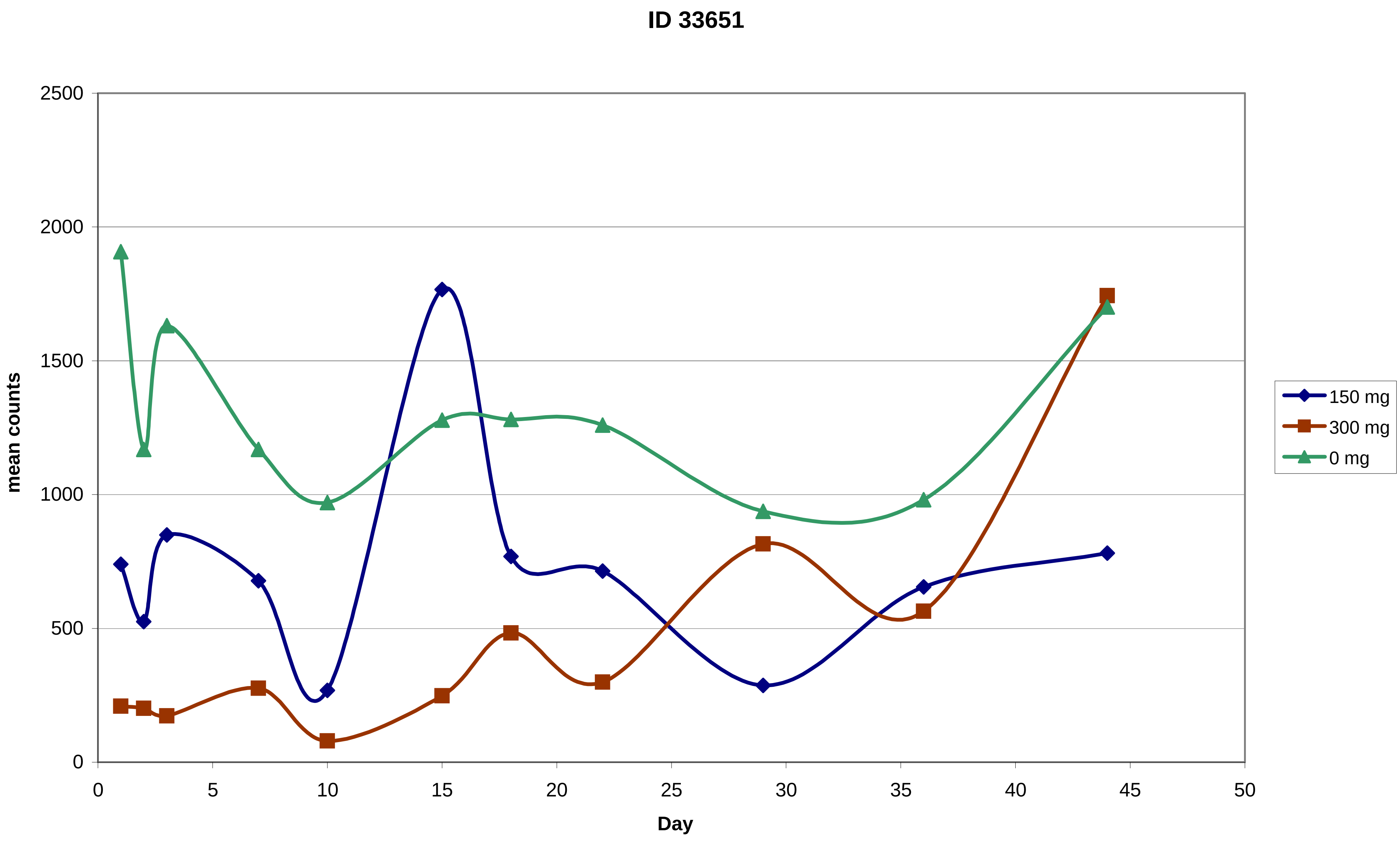

ID 33651
mean counts
0
500
1000
1500
2000
2500
Day
0
5
10
15
20
25
30
35
40
45
50
150 mg
300 mg
0 mg

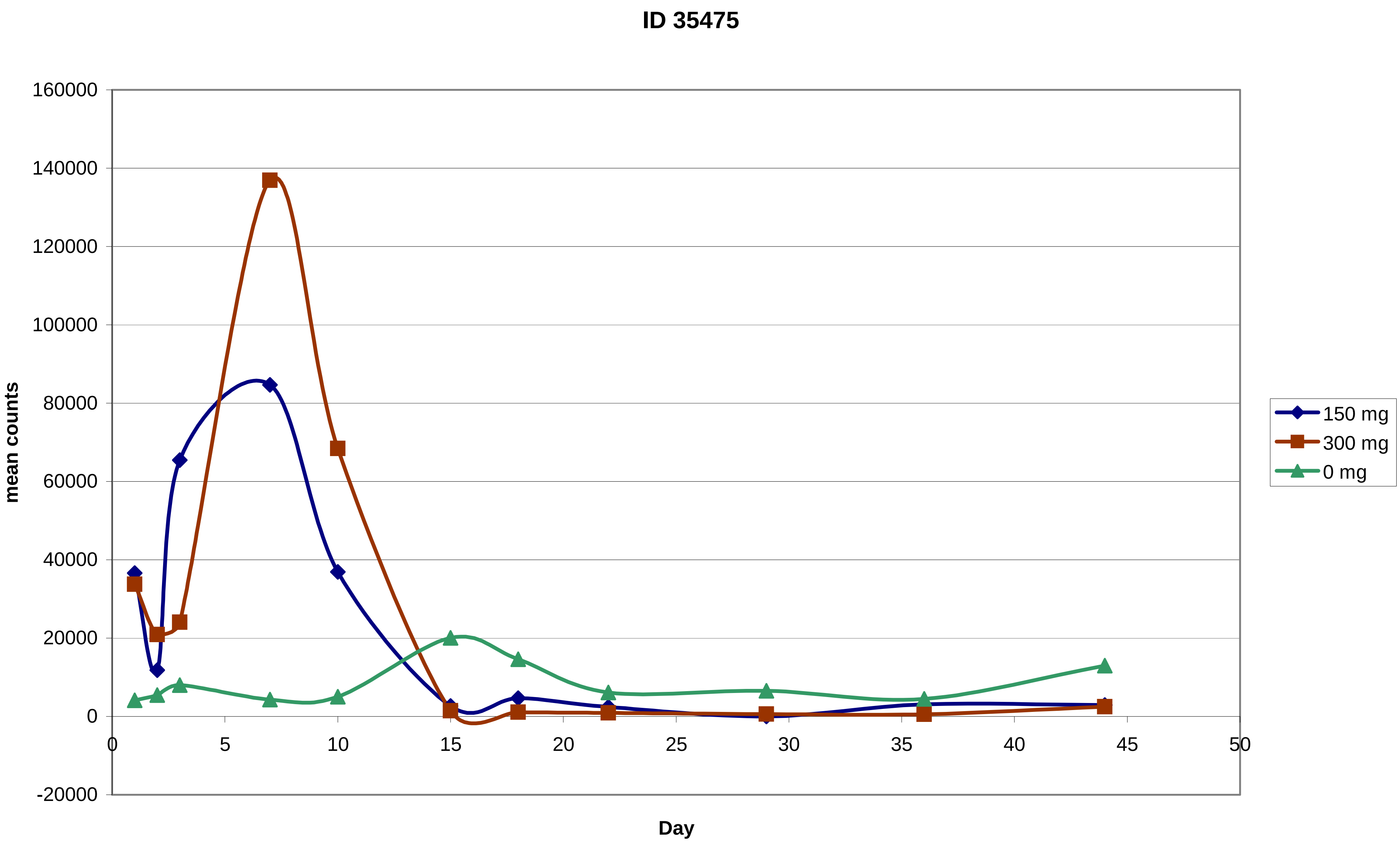
ID 35475
mean counts
160000
140000
120000
100000
80000
60000
40000
20000
0
-20000
0
5
10
15
20
25
30
35
40
45
50
Day
150 mg
300 mg
0 mg

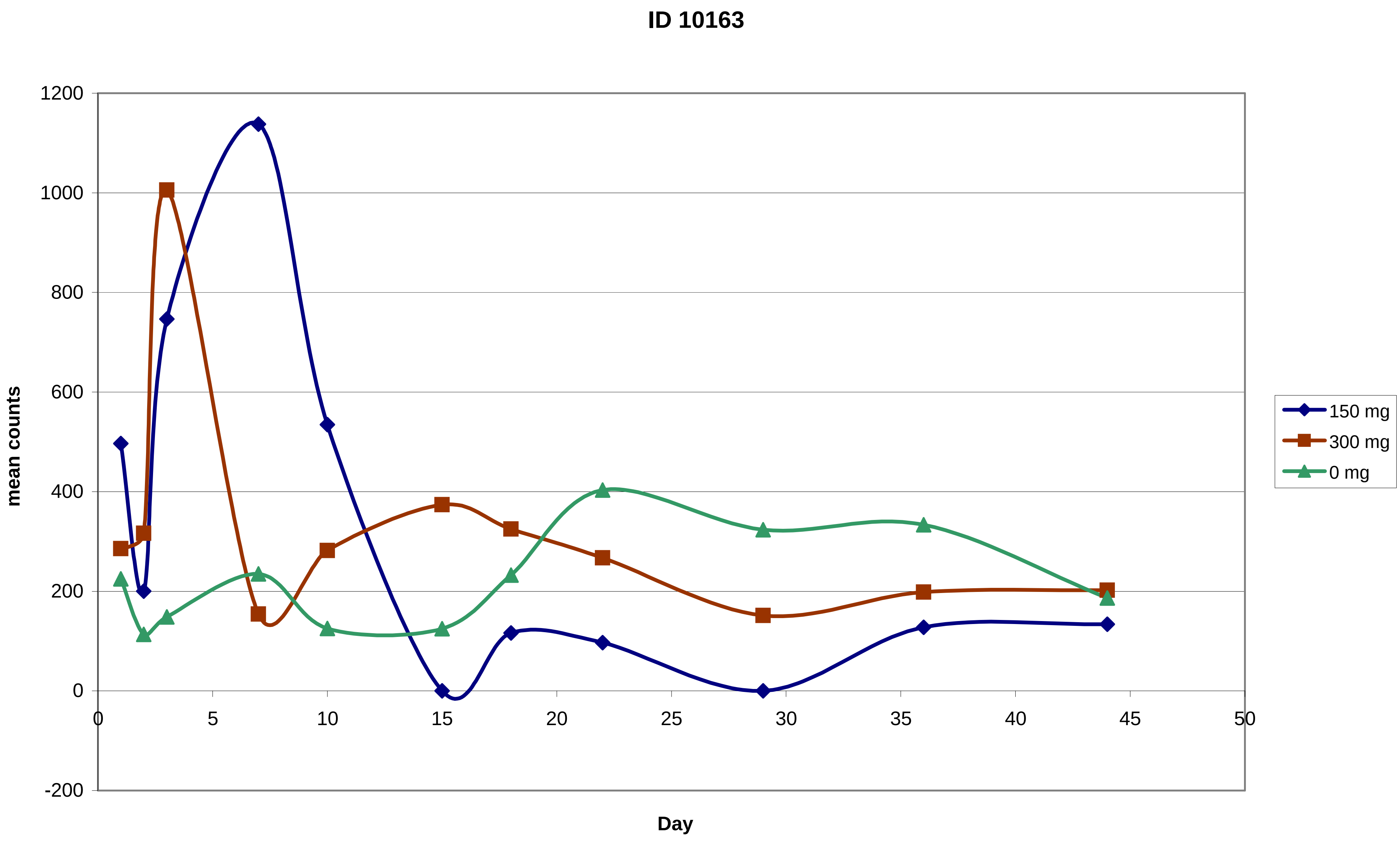

ID 10163
mean counts
1200
1000
800
600
400
200
0
-200
0
5
10
15
20
25
30
35
40
45
50
Day
150 mg
300 mg
0 mg

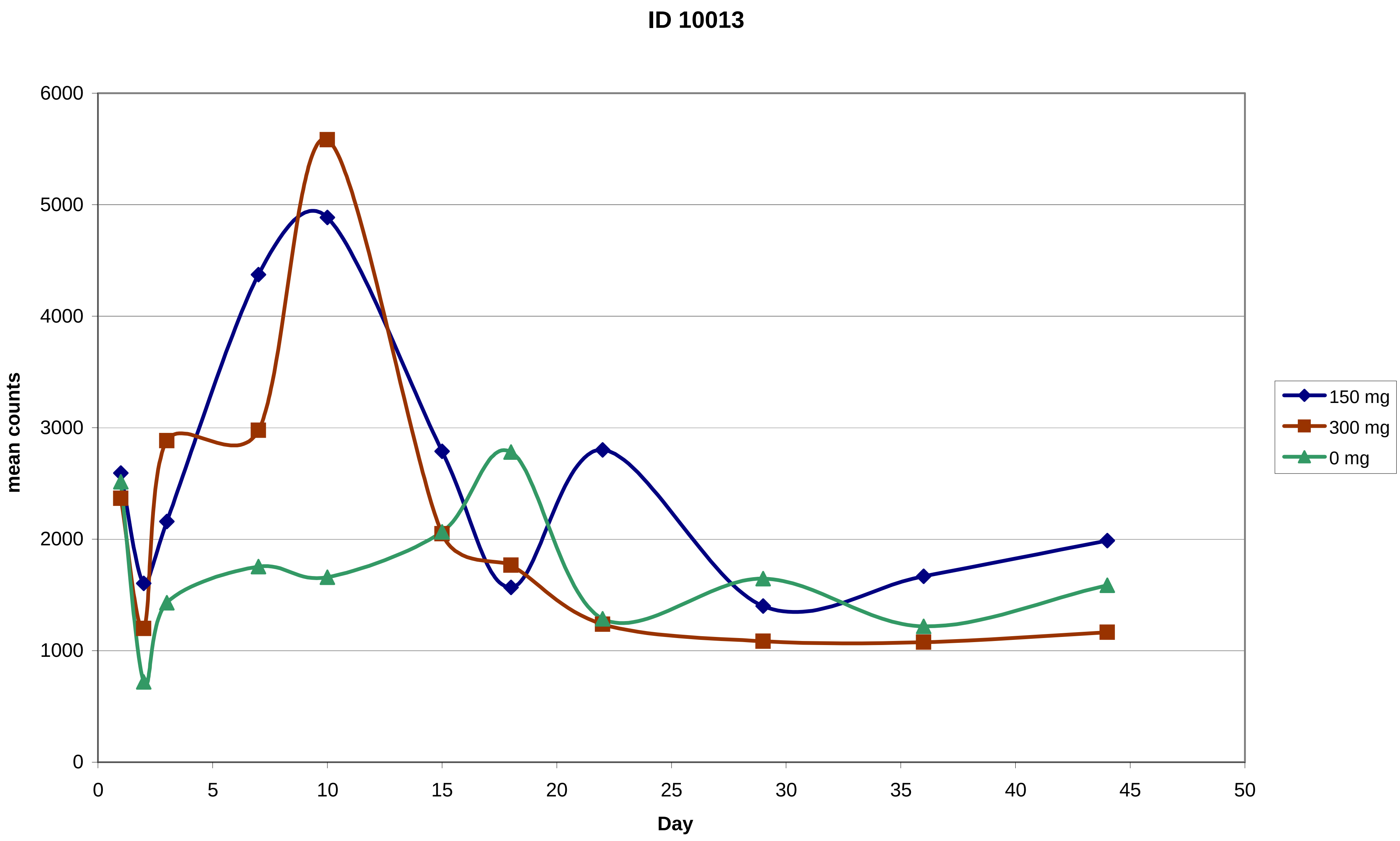
ID 10013
mean counts
Day
150 mg
300 mg
0 mg
0
1000
2000
3000
4000
5000
6000
0
5
10
15
20
25
30
35
40
45
50

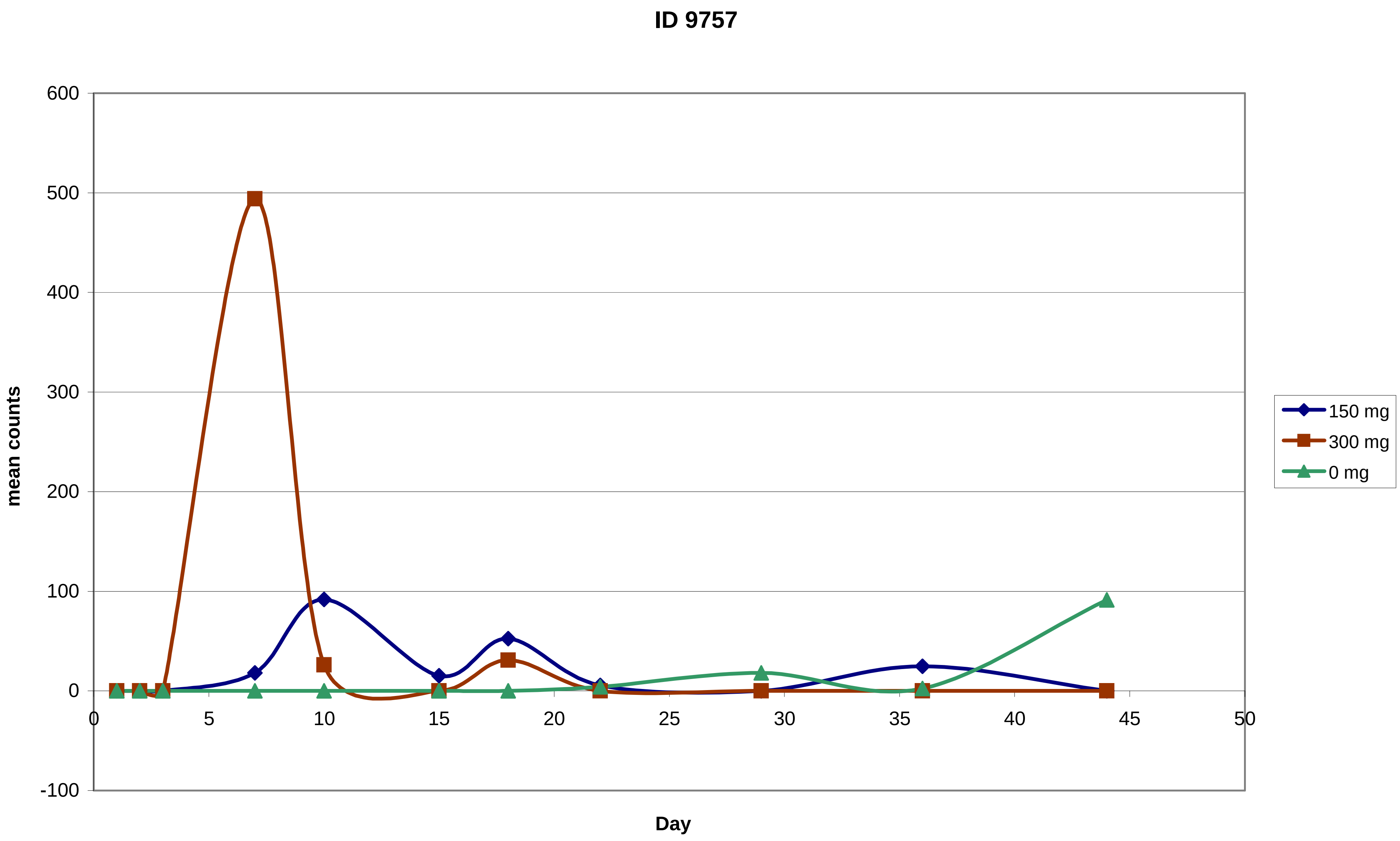

ID 9757
600
500
400
300
200
100
0
-100
mean counts
0
5
10
15
20
25
30
35
40
45
50
Day
150 mg
300 mg
0 mg

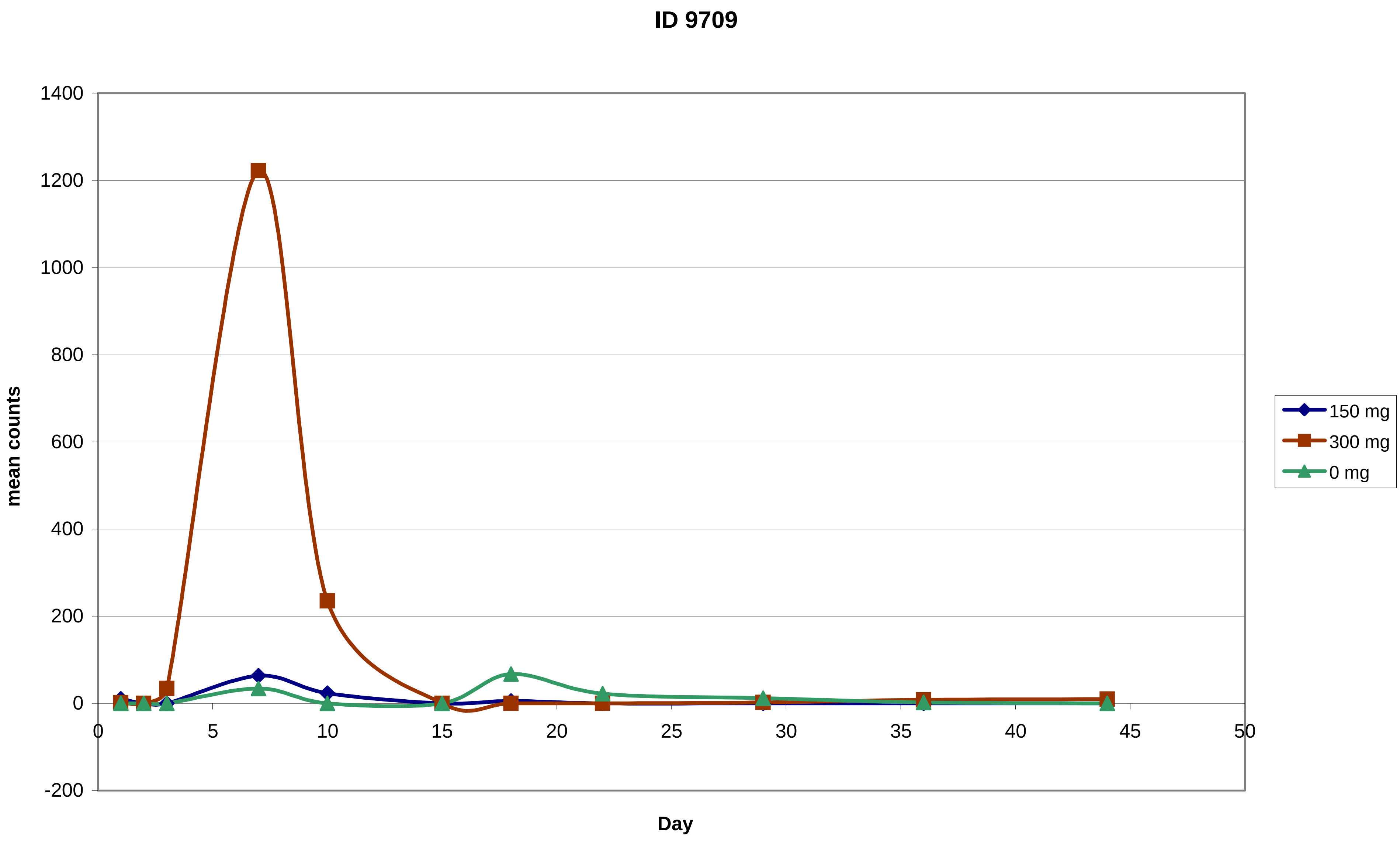

ID 9709
mean counts
Day
1400
1200
1000
800
600
400
200
0
-200
0
5
10
15
20
25
30
35
40
45
50
150 mg
300 mg
0 mg

ID 9626

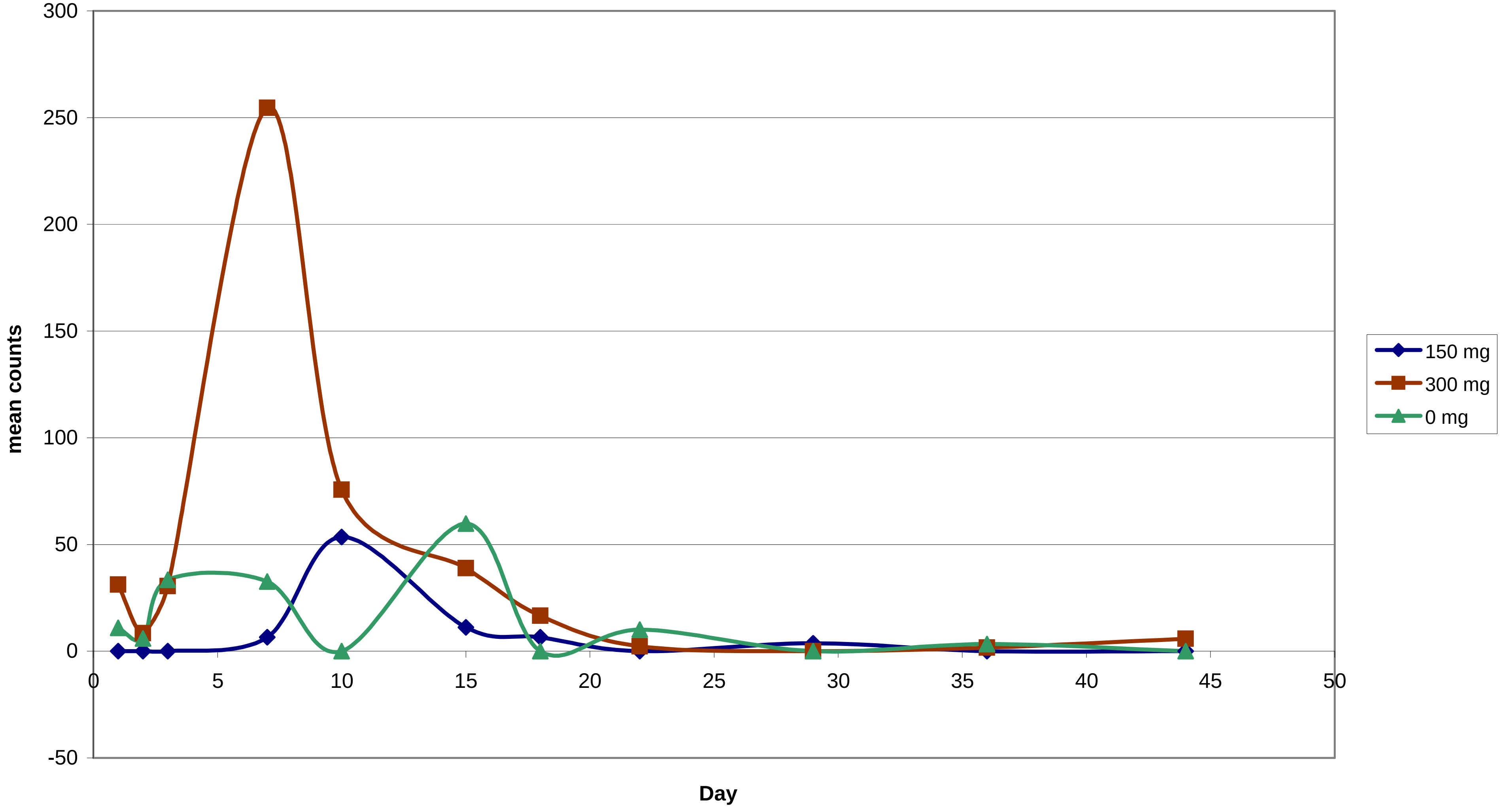

300
250
200
150
100
50
0
-50
mean counts
0
5
10
15
20
25
30
35
40
45
50
Day
150 mg
300 mg
0 mg

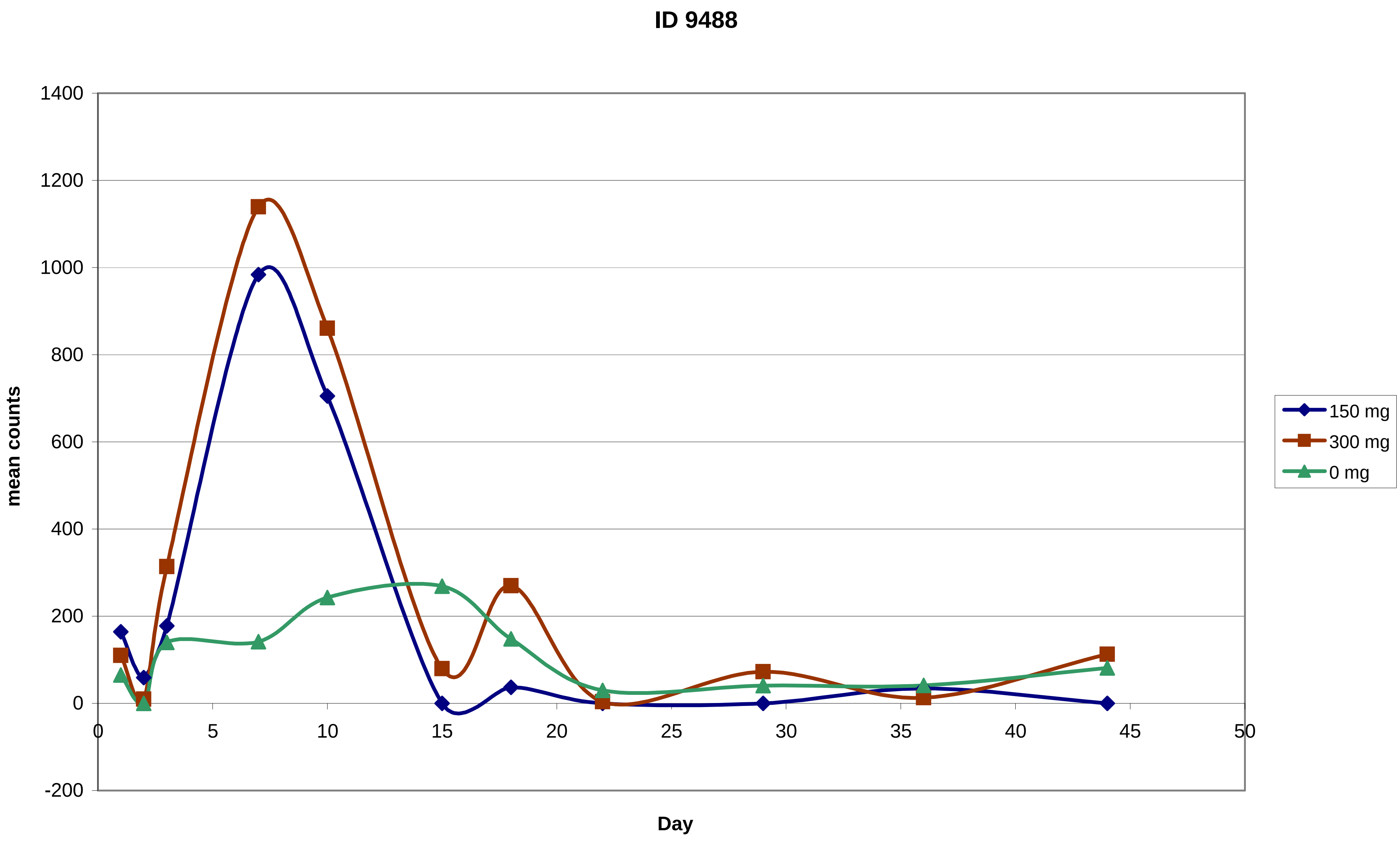

ID 9488
1400
1200
1000
800
600
400
200
0
-200
mean counts
0
5
10
15
20
25
30
35
40
45
50
Day
150 mg
300 mg
0 mg

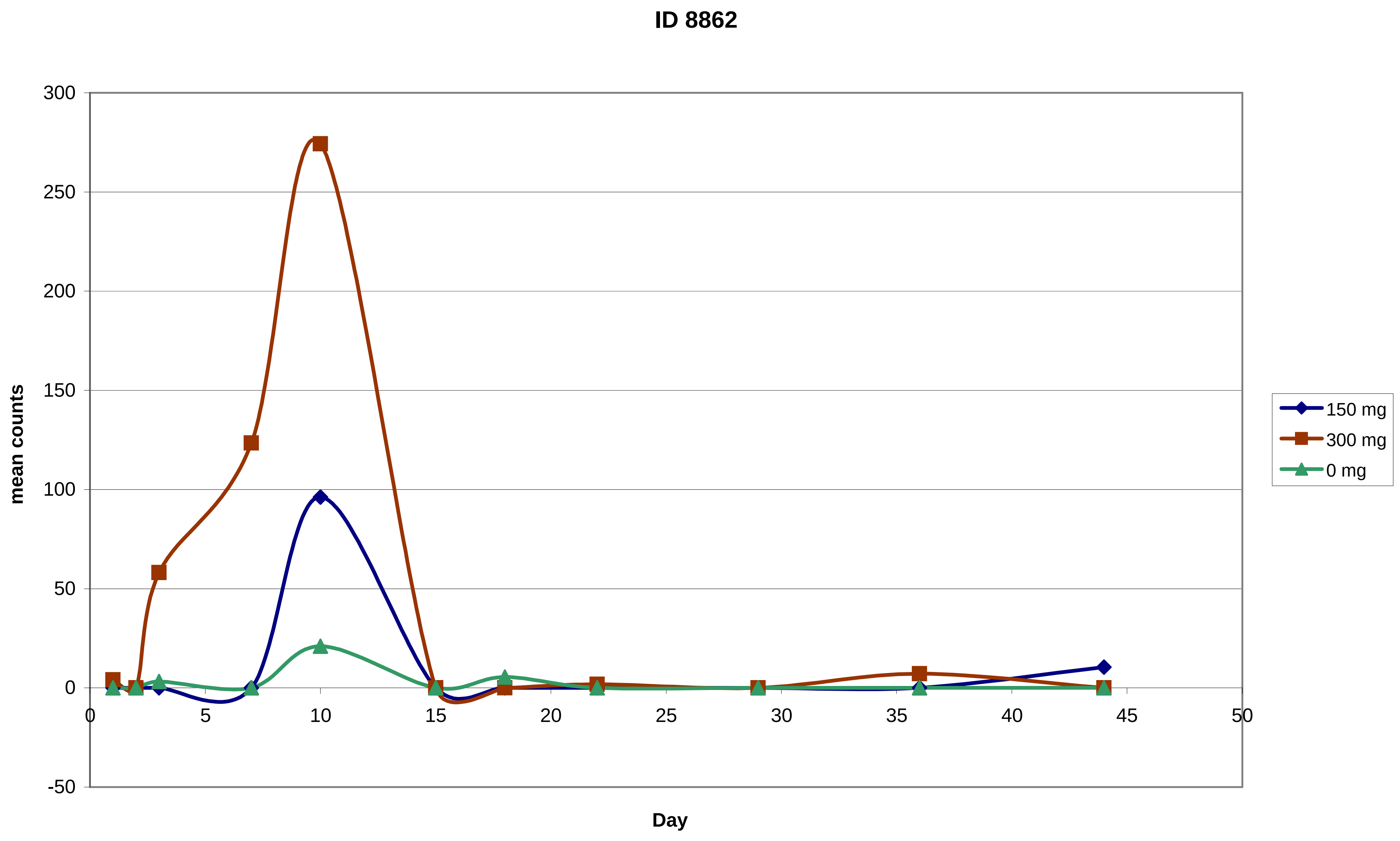

ID 8862
mean counts
300
250
200
150
100
50
0
-50
0
5
10
15
20
25
30
35
40
45
50
Day
150 mg
300 mg
0 mg

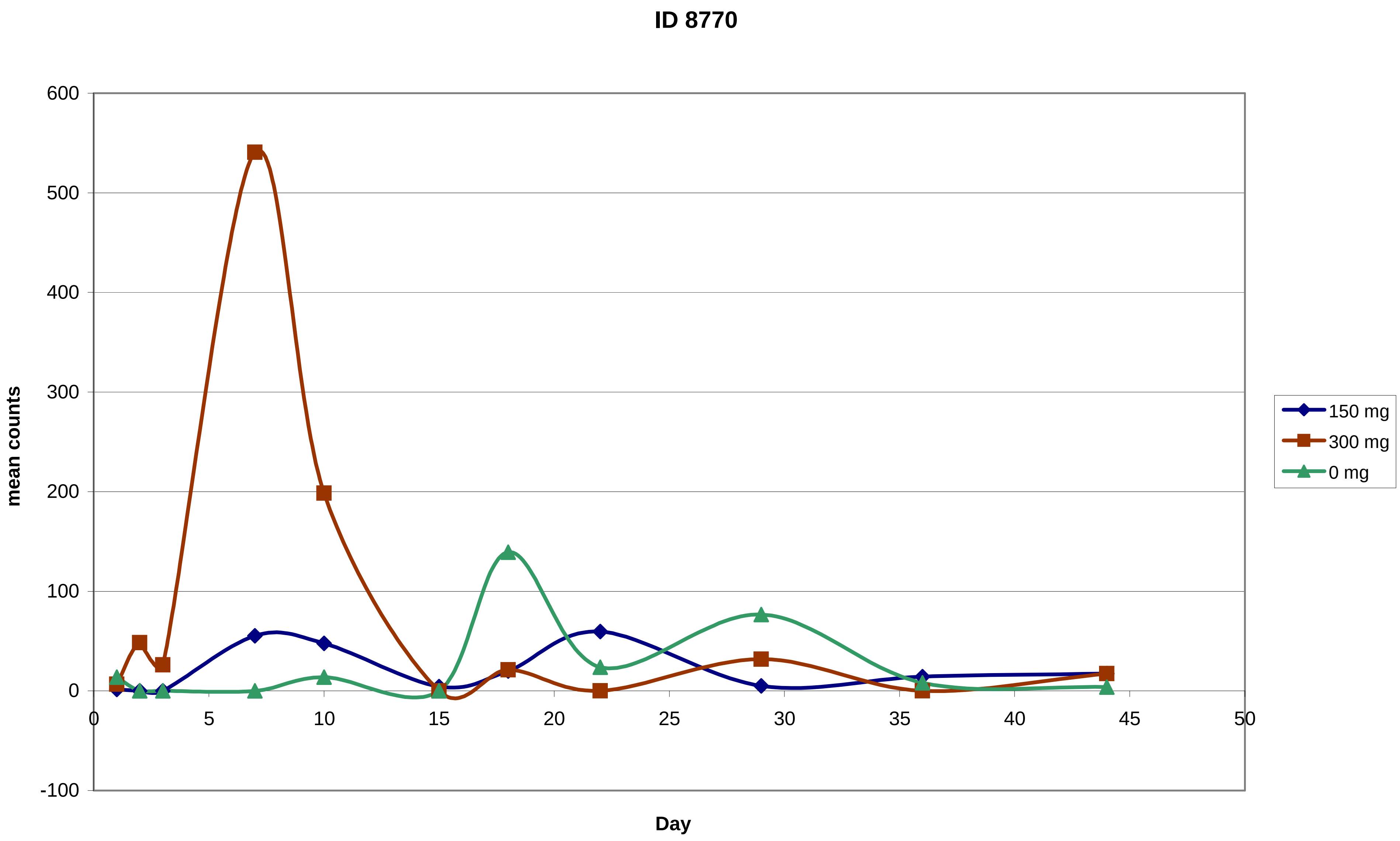
ID 8770
mean counts
600
500
400
300
200
100
0
-100
0
5
10
15
20
25
30
35
40
45
50
Day
150 mg
300 mg
0 mg

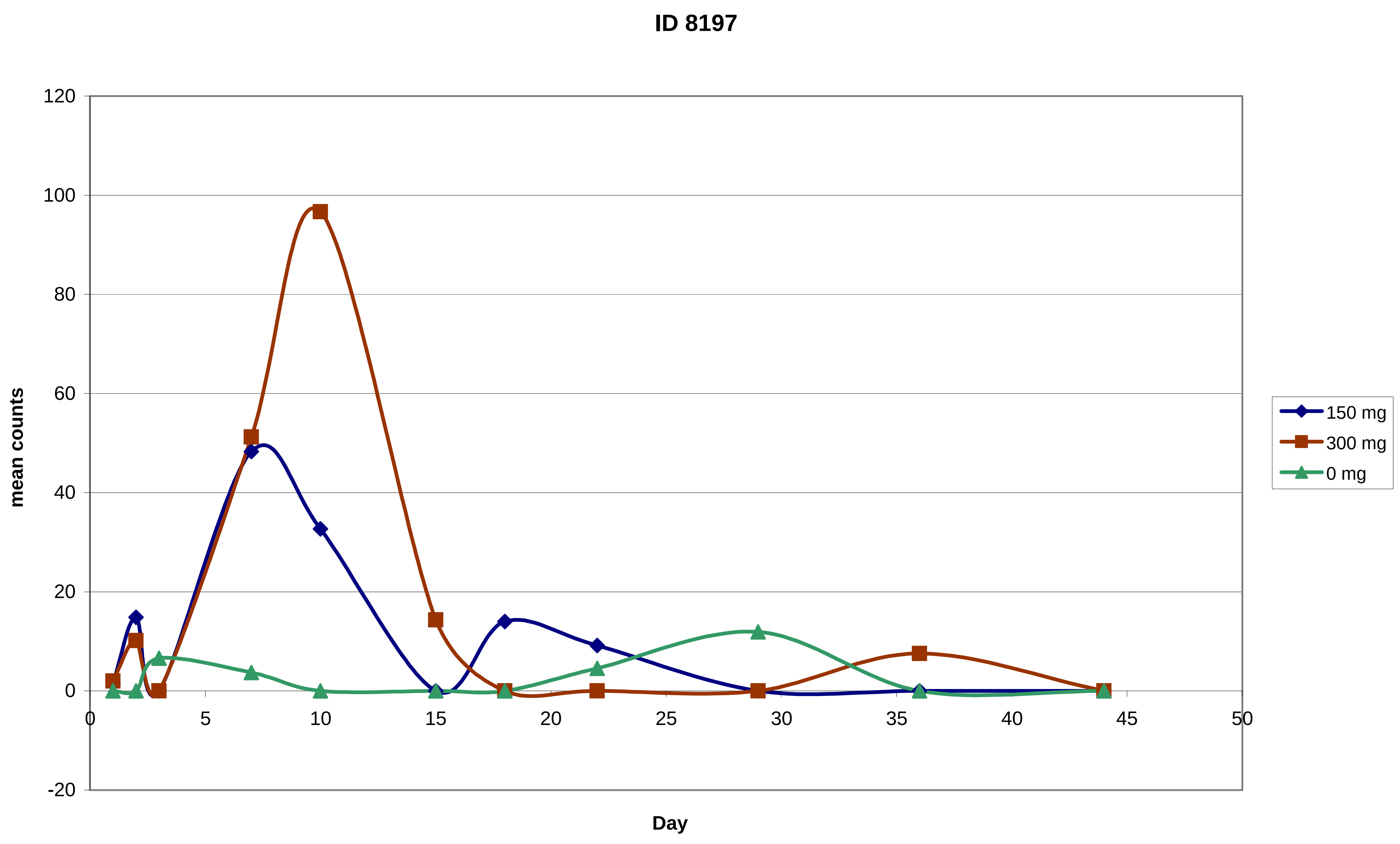

ID 8197
120
100
80
60
40
20
0
-20
mean counts
0
5
10
15
20
25
30
35
40
45
50
Day
150 mg
300 mg
0 mg

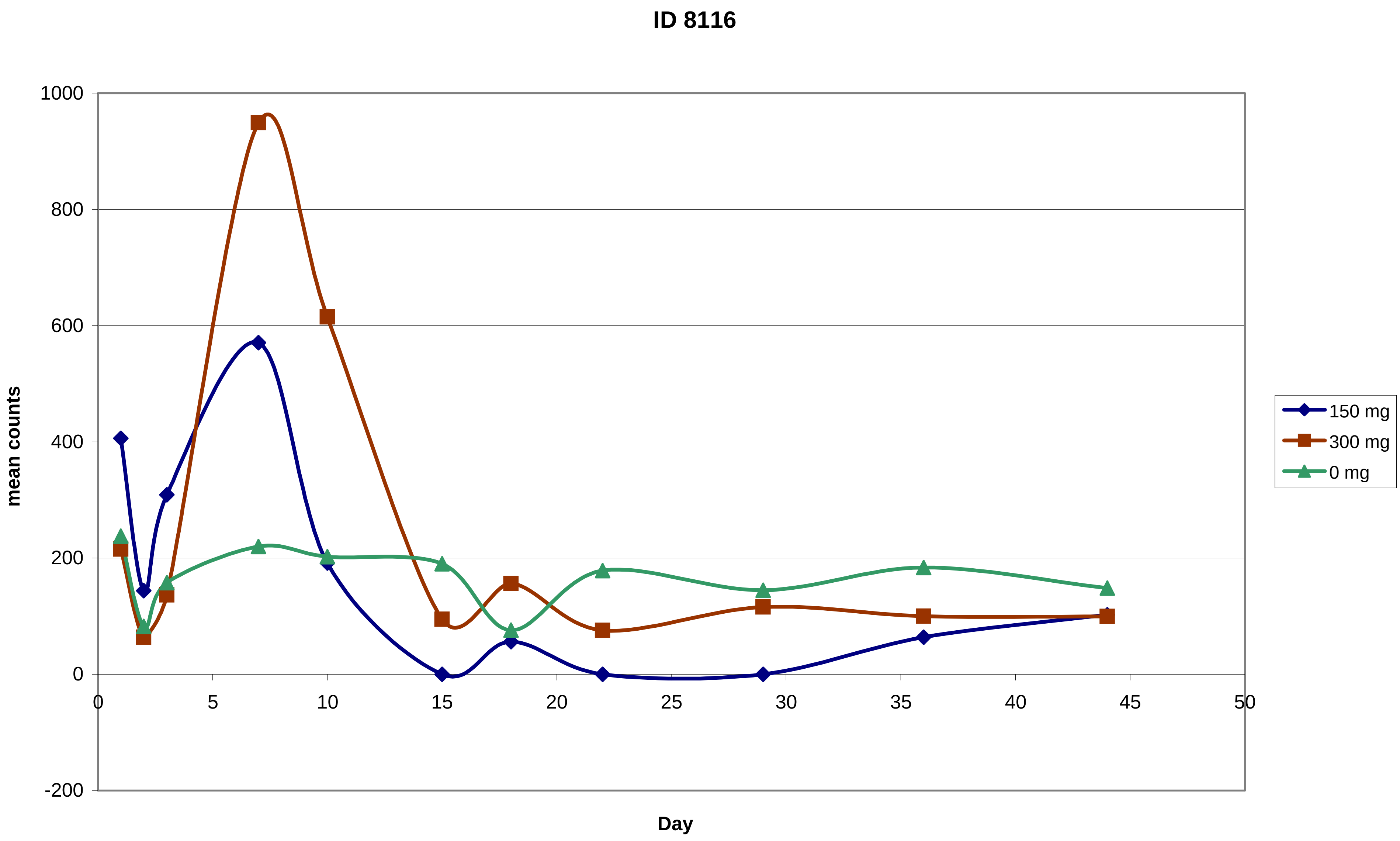

ID 8116
1000
800
600
400
200
0
-200
mean counts
0
5
10
15
20
25
30
35
40
45
50
Day
150 mg
300 mg
0 mg

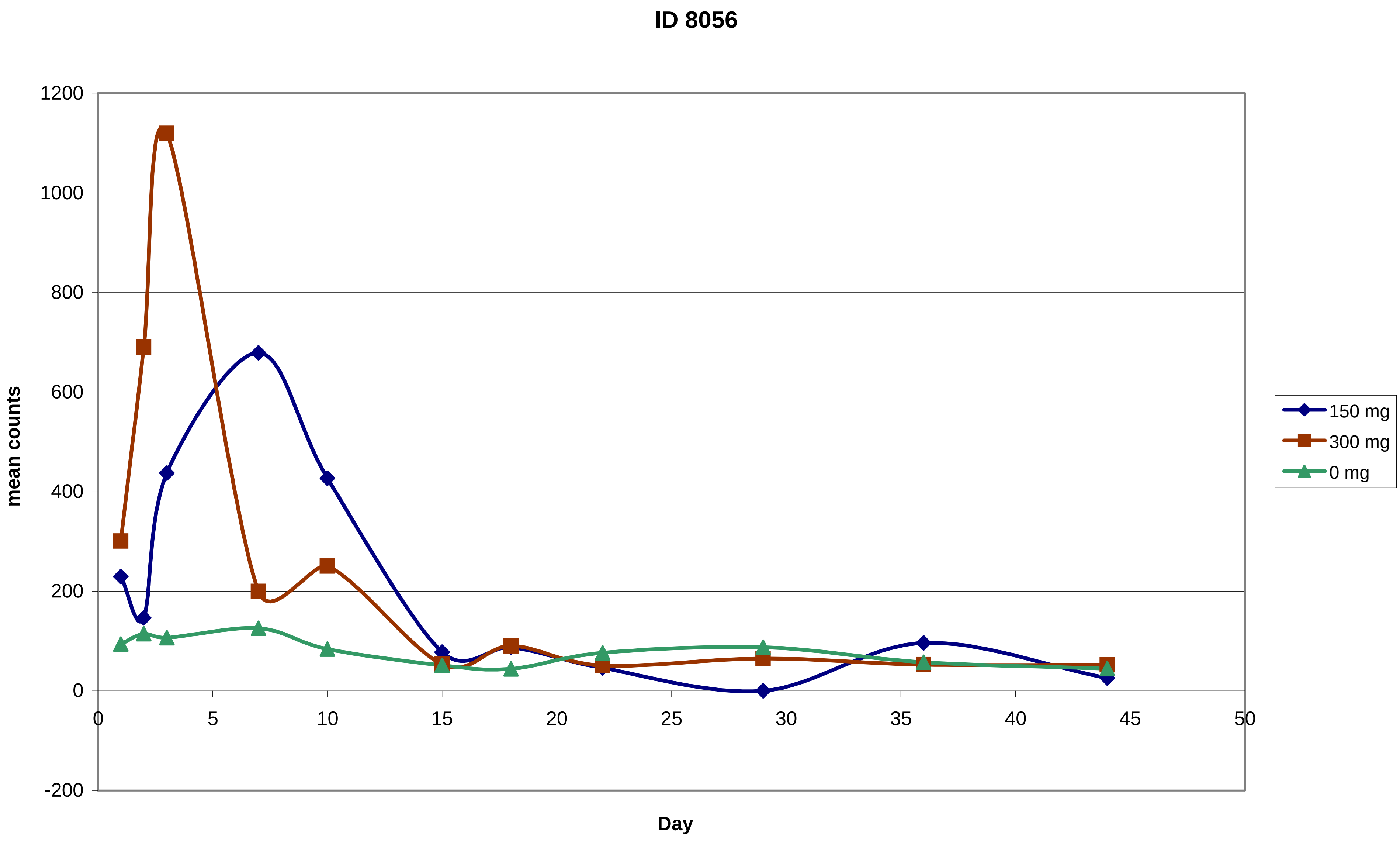
ID 8056
mean counts
Day
1200
1000
800
600
400
200
0
-200
0
5
10
15
20
25
30
35
40
45
50
150 mg
300 mg
0 mg

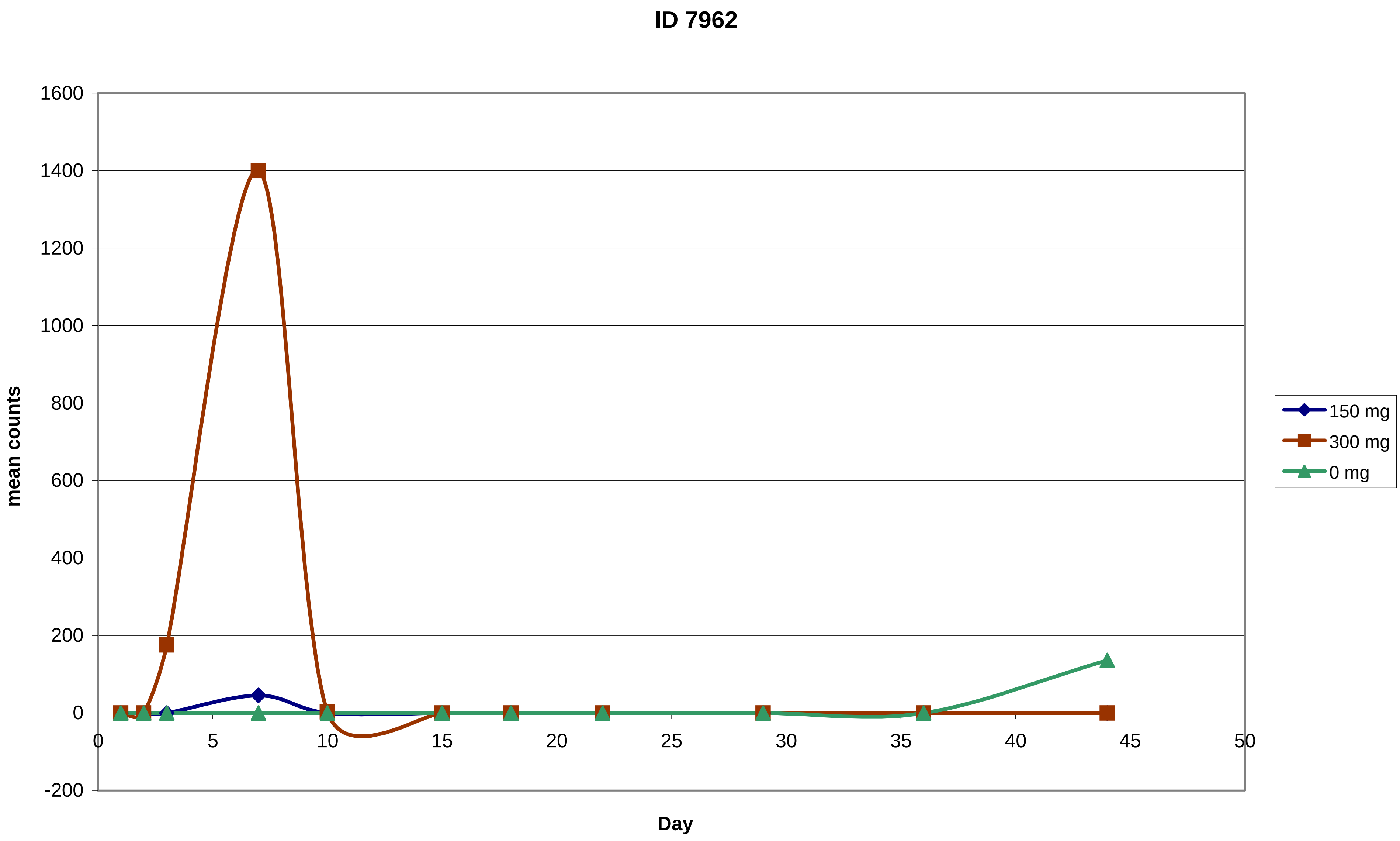

ID 7962
mean counts
1600
1400
1200
1000
800
600
400
200
0
-200
0
5
10
15
20
25
30
35
40
45
50
Day
150 mg
300 mg
0 mg

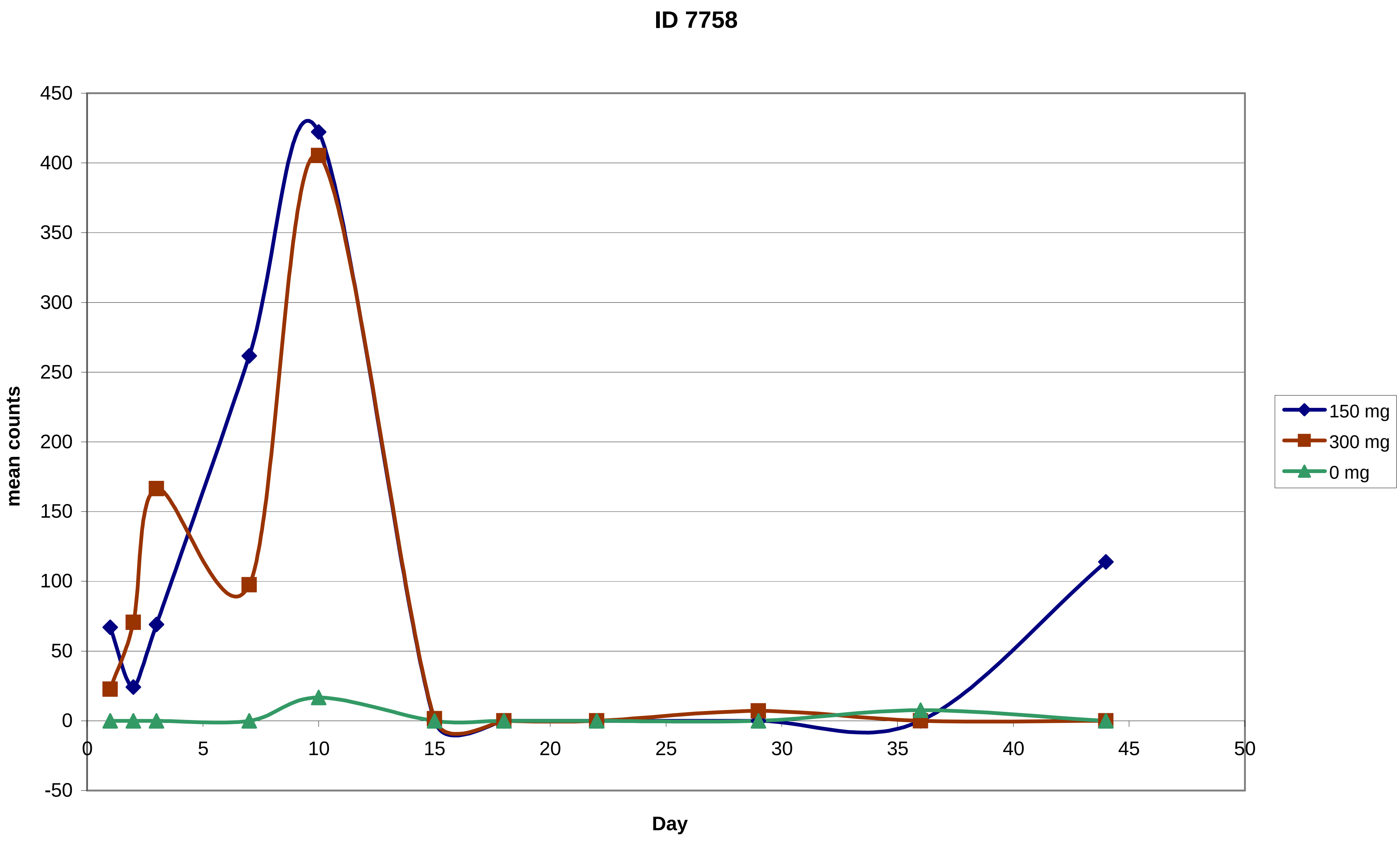
ID 7758
mean counts
450
400
350
300
250
200
150
100
50
0
-50
0
5
10
15
20
25
30
35
40
45
50
Day
150 mg
300 mg
0 mg

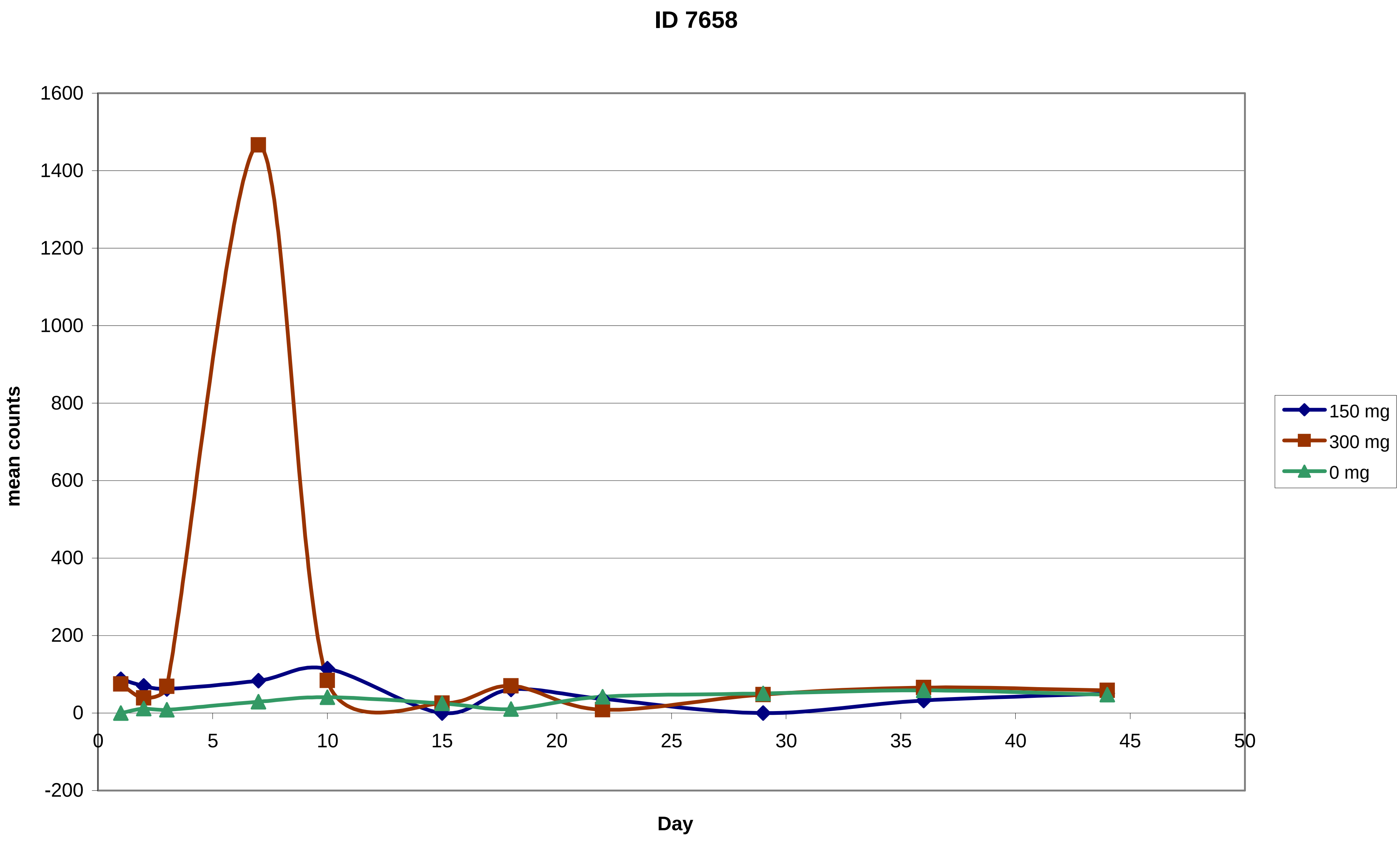
ID 7658
mean counts
1600
1400
1200
1000
800
600
400
200
0
-200
0
5
10
15
20
25
30
35
40
45
50
Day
150 mg
300 mg
0 mg

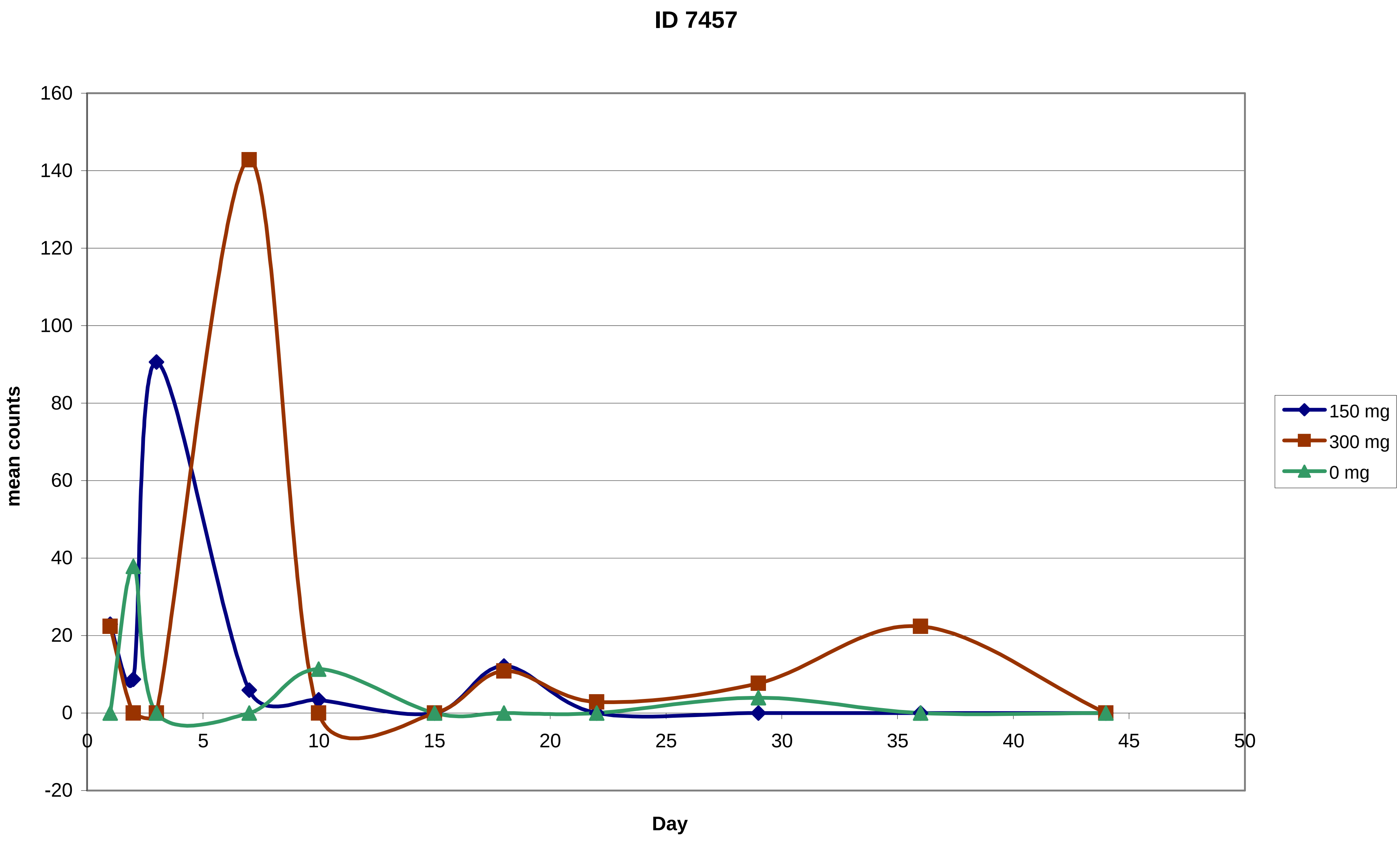

ID 7457
mean counts
Day
160
140
120
100
80
60
40
20
0
-20
0
5
10
15
20
25
30
35
40
45
50
150 mg
300 mg
0 mg

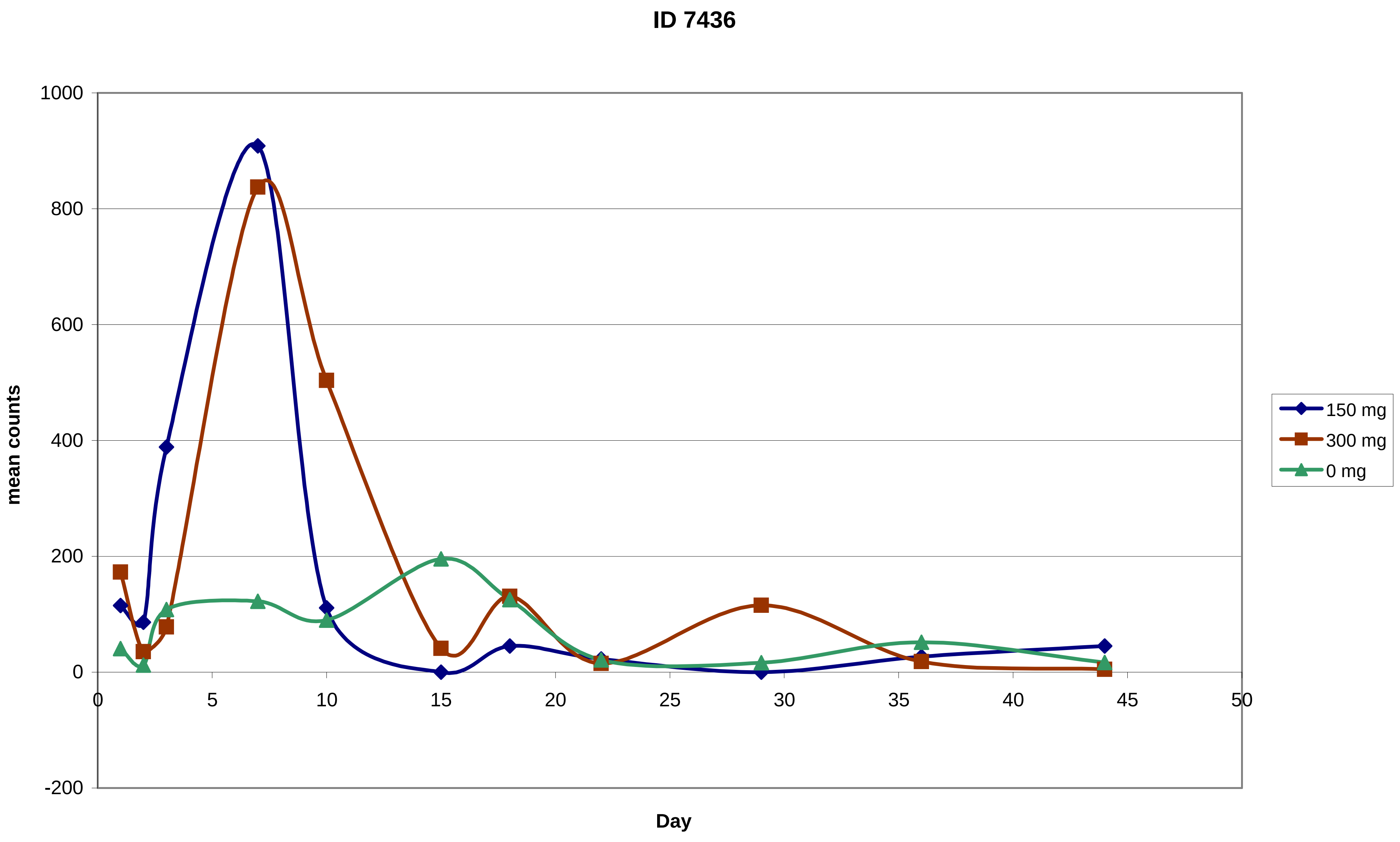

ID 7436
1000
800
600
400
200
0
-200
mean counts
0
5
10
15
20
25
30
35
40
45
50
Day
150 mg
300 mg
0 mg

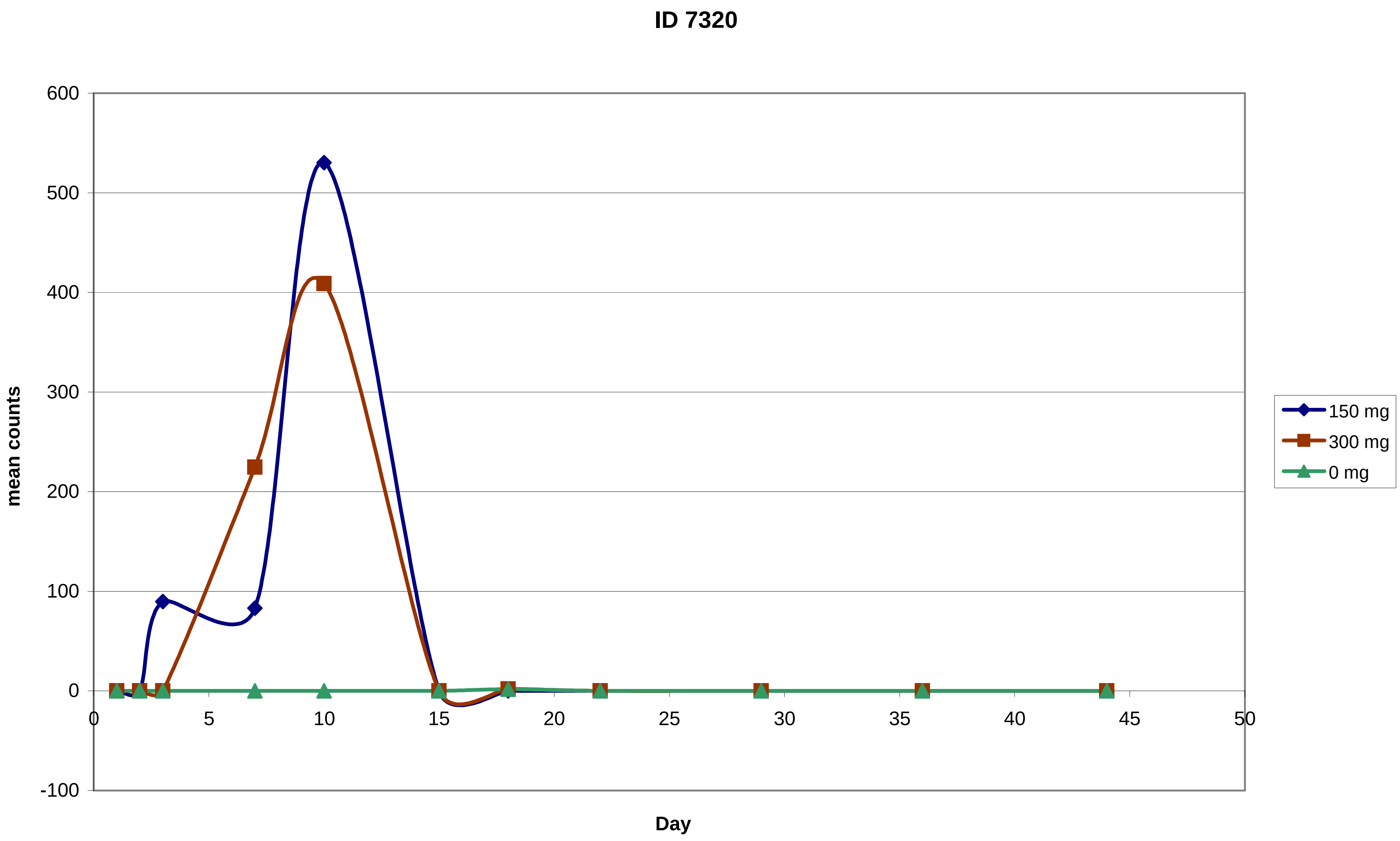
ID 7320
600
500
400
300
200
100
0
-100
mean counts
0
5
10
15
20
25
30
35
40
45
50
Day
150 mg
300 mg
0 mg

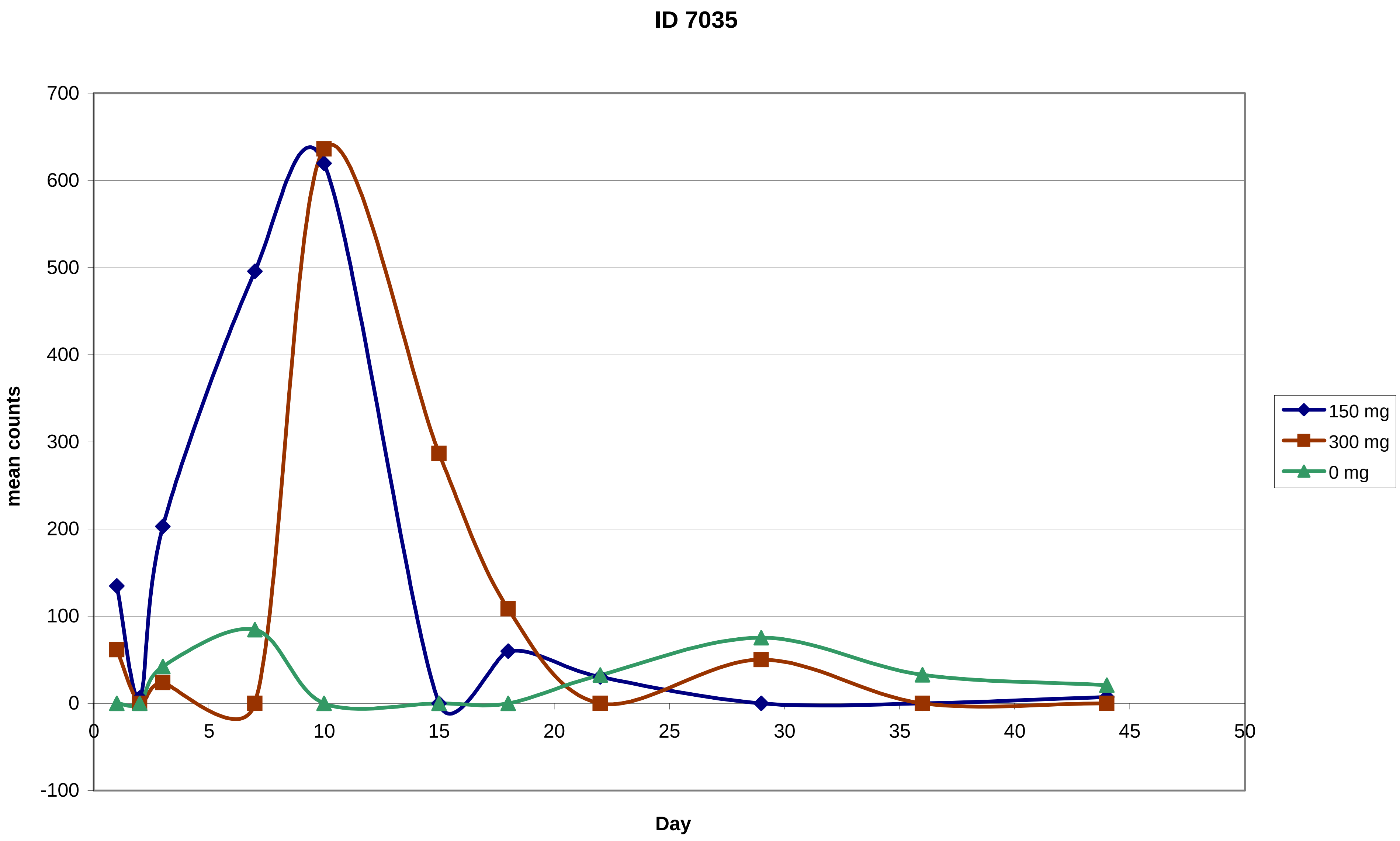

ID 7035
mean counts
700
600
500
400
300
200
100
0
-100
0
5
10
15
20
25
30
35
40
45
50
Day
150 mg
300 mg
0 mg

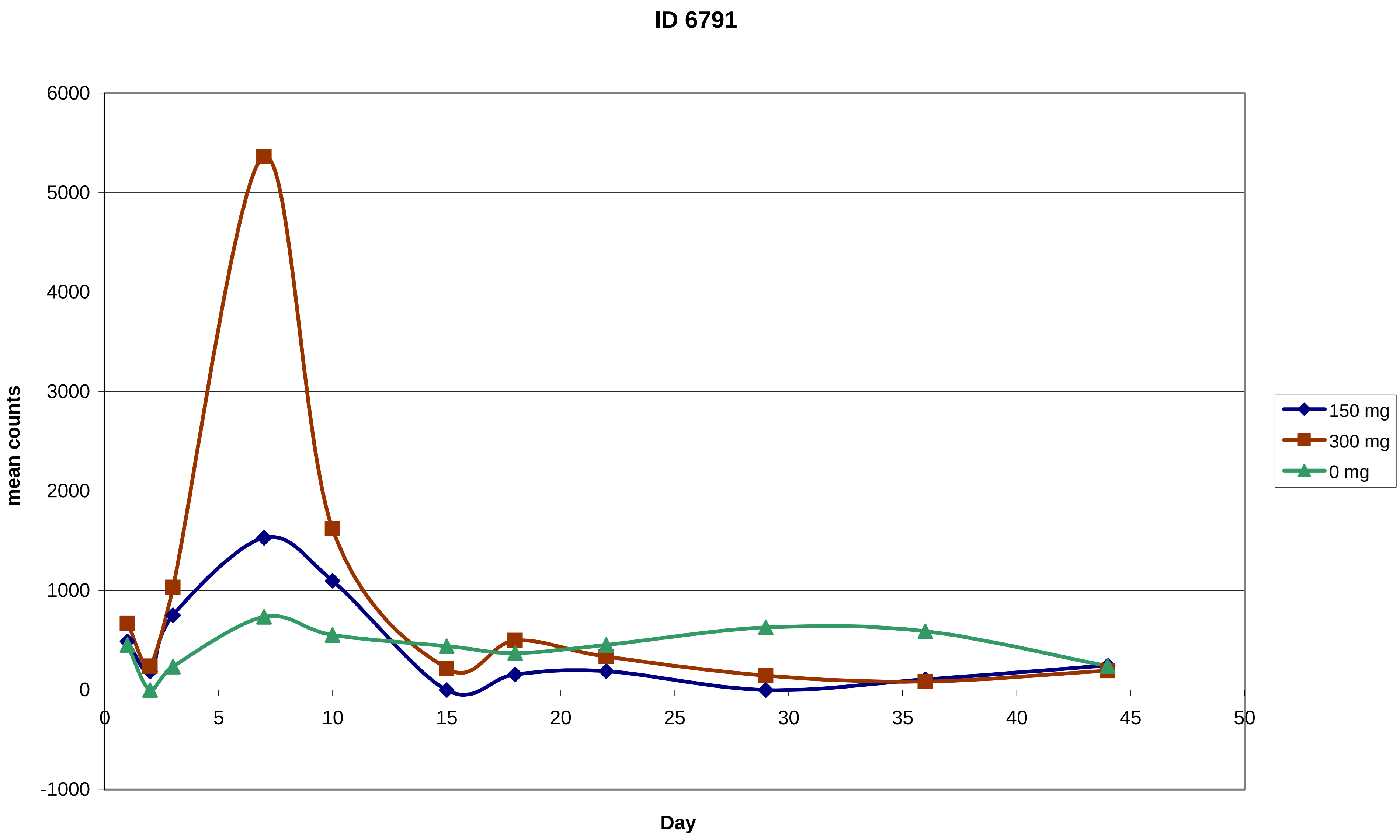
ID 6791
mean counts
6000
5000
4000
3000
2000
1000
0
-1000
0
5
10
15
20
25
30
35
40
45
50
Day
150 mg
300 mg
0 mg

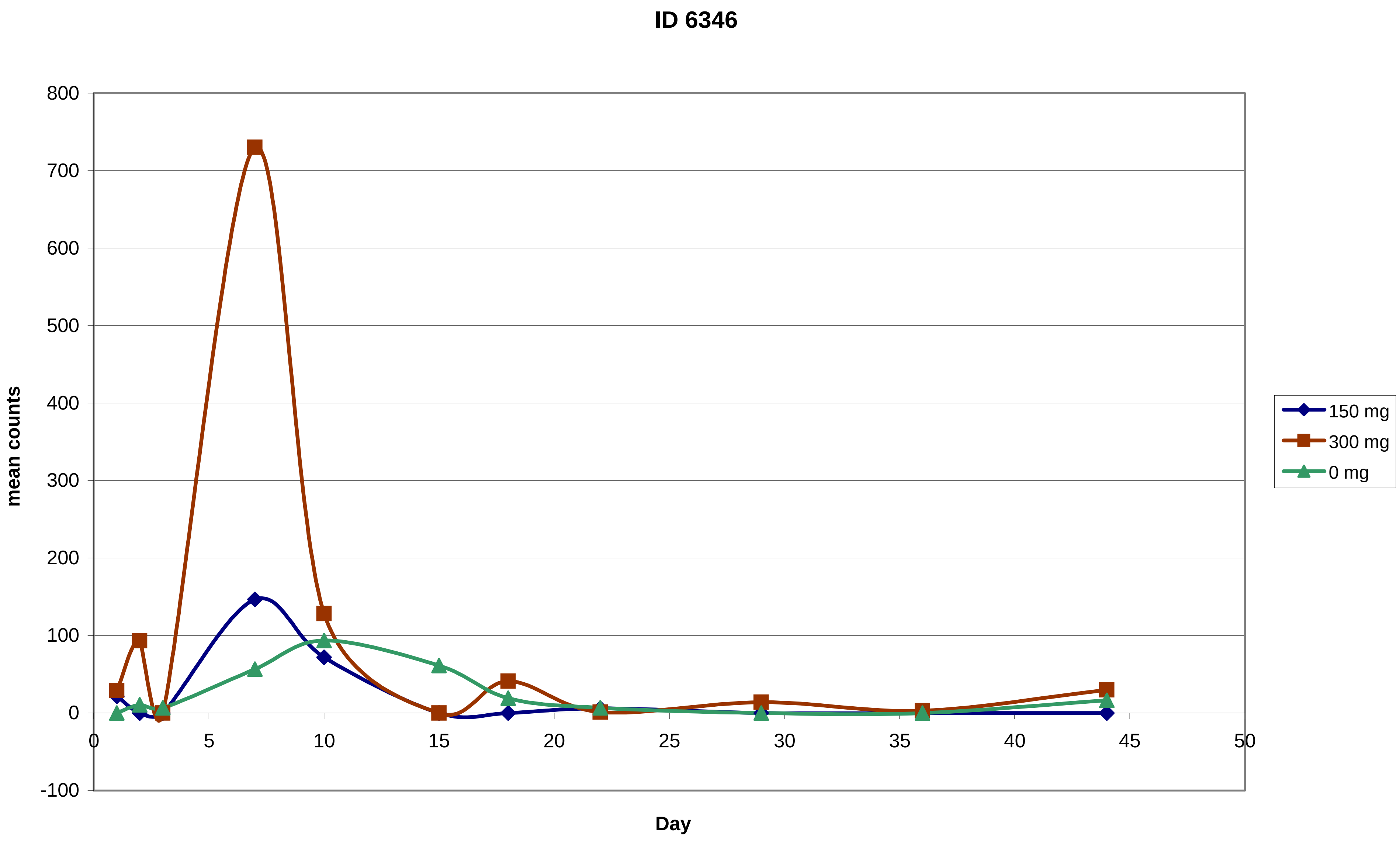
ID 6346
mean counts
800
700
600
500
400
300
200
100
0
-100
0
5
10
15
20
25
30
35
40
45
50
Day
150 mg
300 mg
0 mg

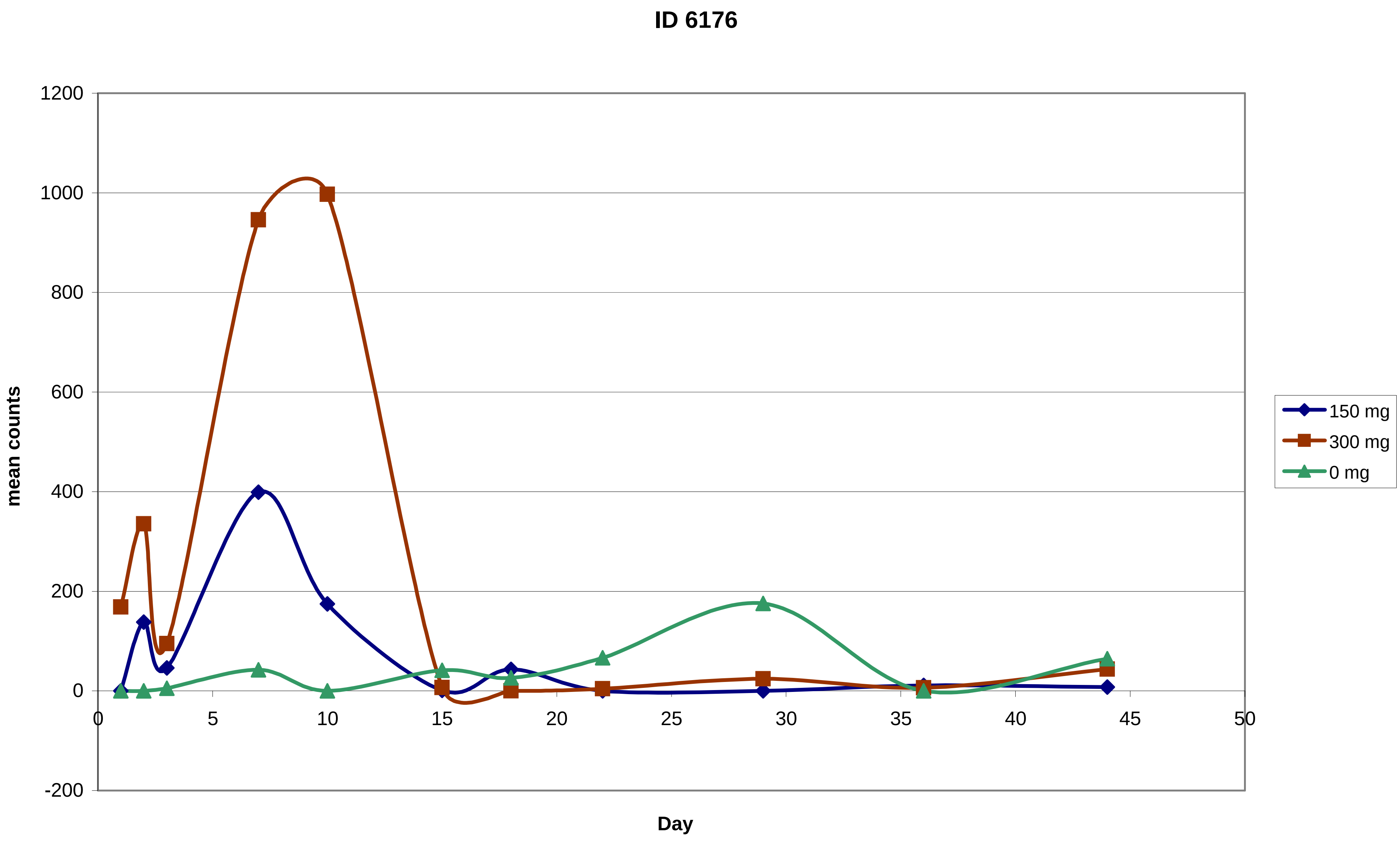

ID 6176
mean counts
1200
1000
800
600
400
200
0
-200
0
5
10
15
20
25
30
35
40
45
50
Day
150 mg
300 mg
0 mg

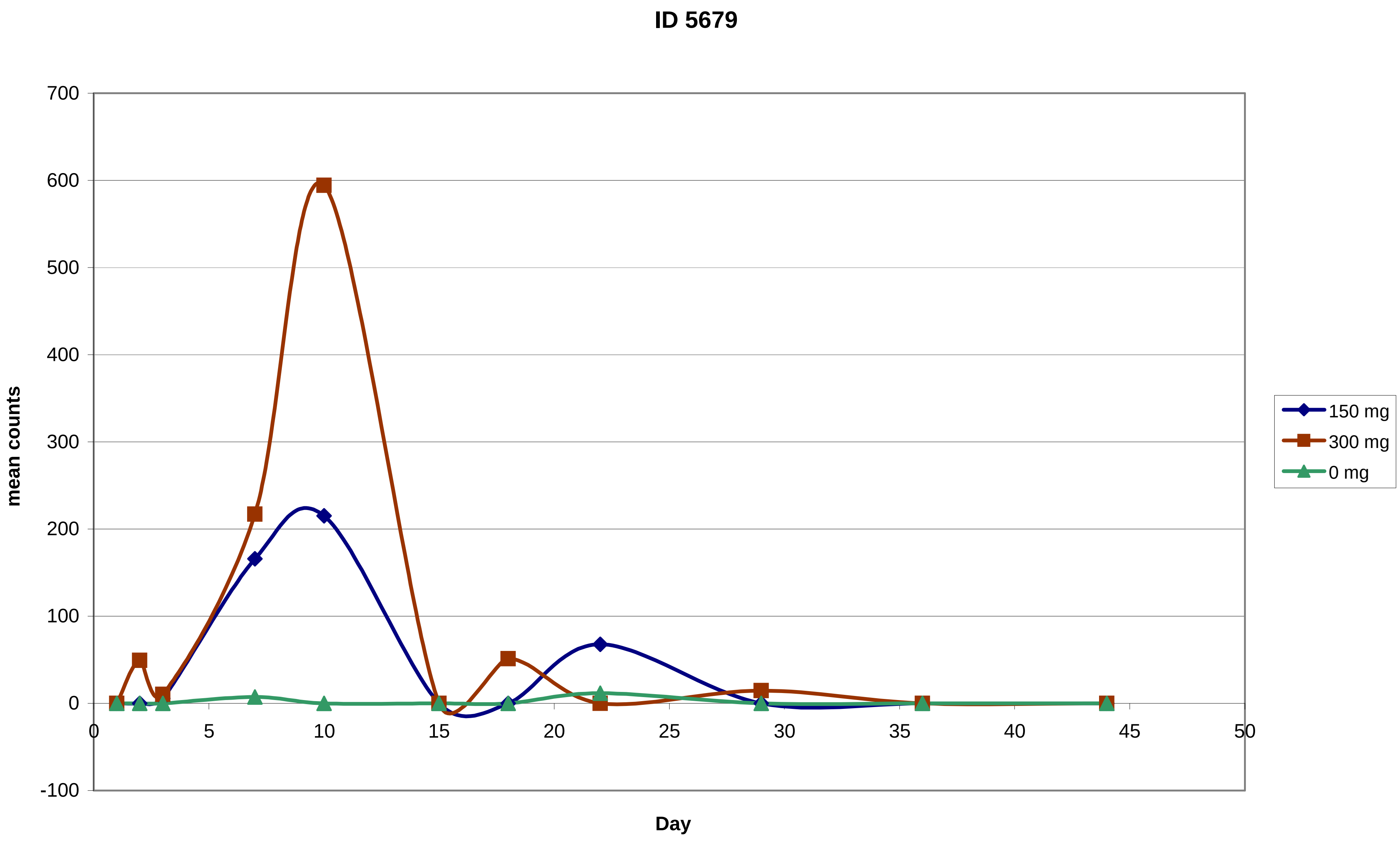

ID 5679
700
600
500
400
300
200
100
0
-100
mean counts
0
5
10
15
20
25
30
35
40
45
50
Day
150 mg
300 mg
0 mg

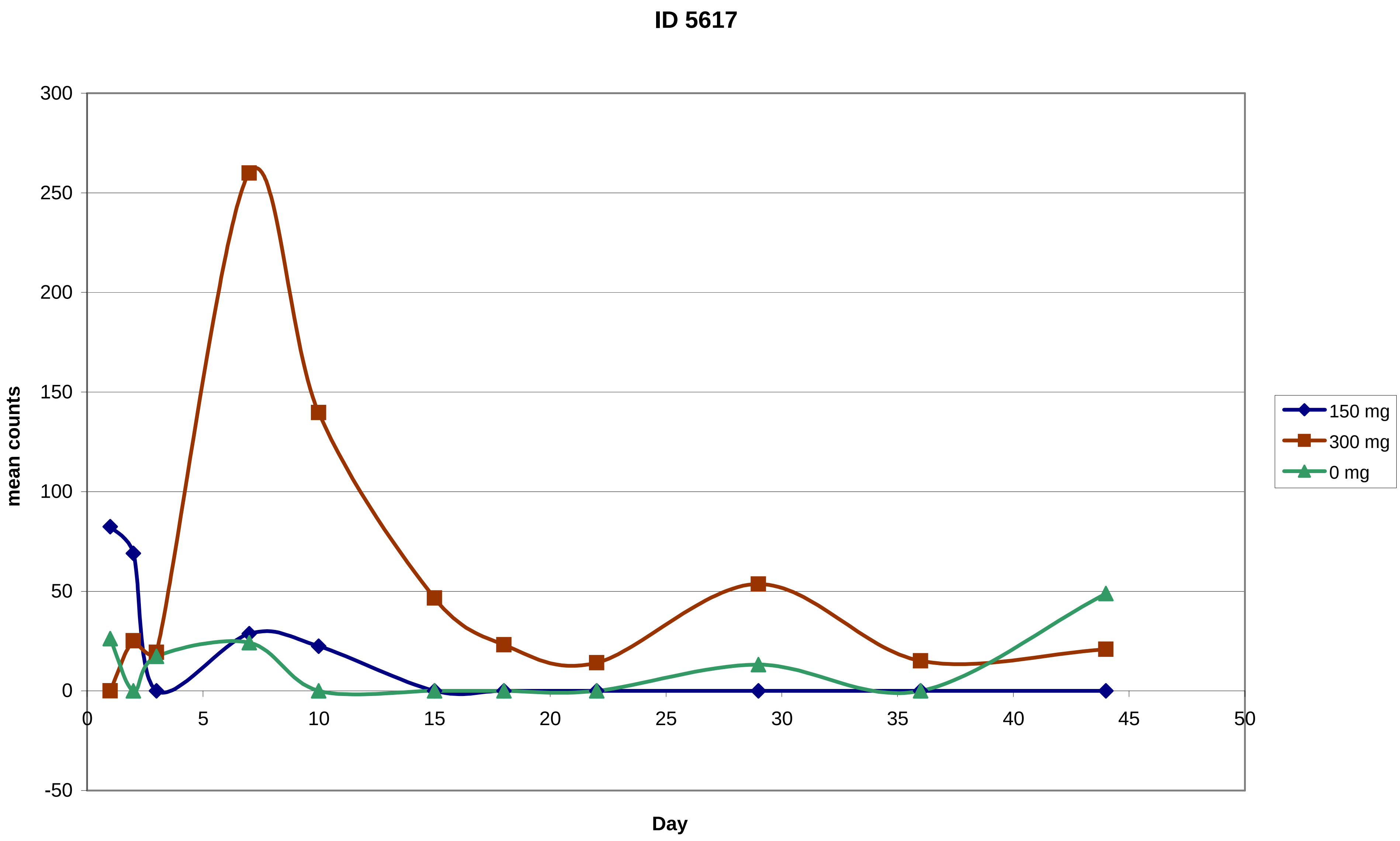
ID 5617
mean counts
300
250
200
150
100
50
0
-50
0
5
10
15
20
25
30
35
40
45
50
Day
150 mg
300 mg
0 mg

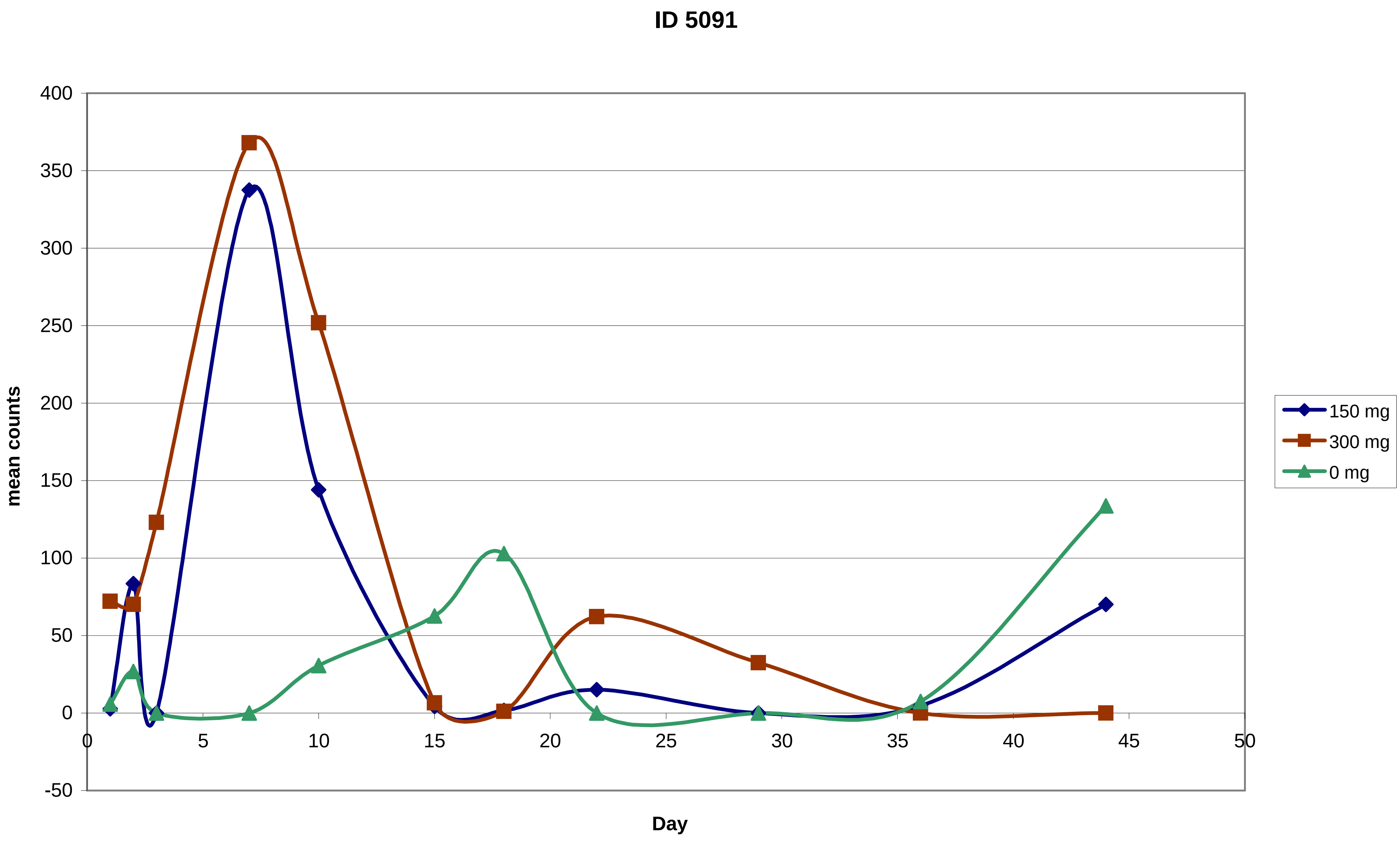
ID 5091
mean counts
400
350
300
250
200
150
100
50
0
-50
0
5
10
15
20
25
30
35
40
45
50
Day
150 mg
300 mg
0 mg

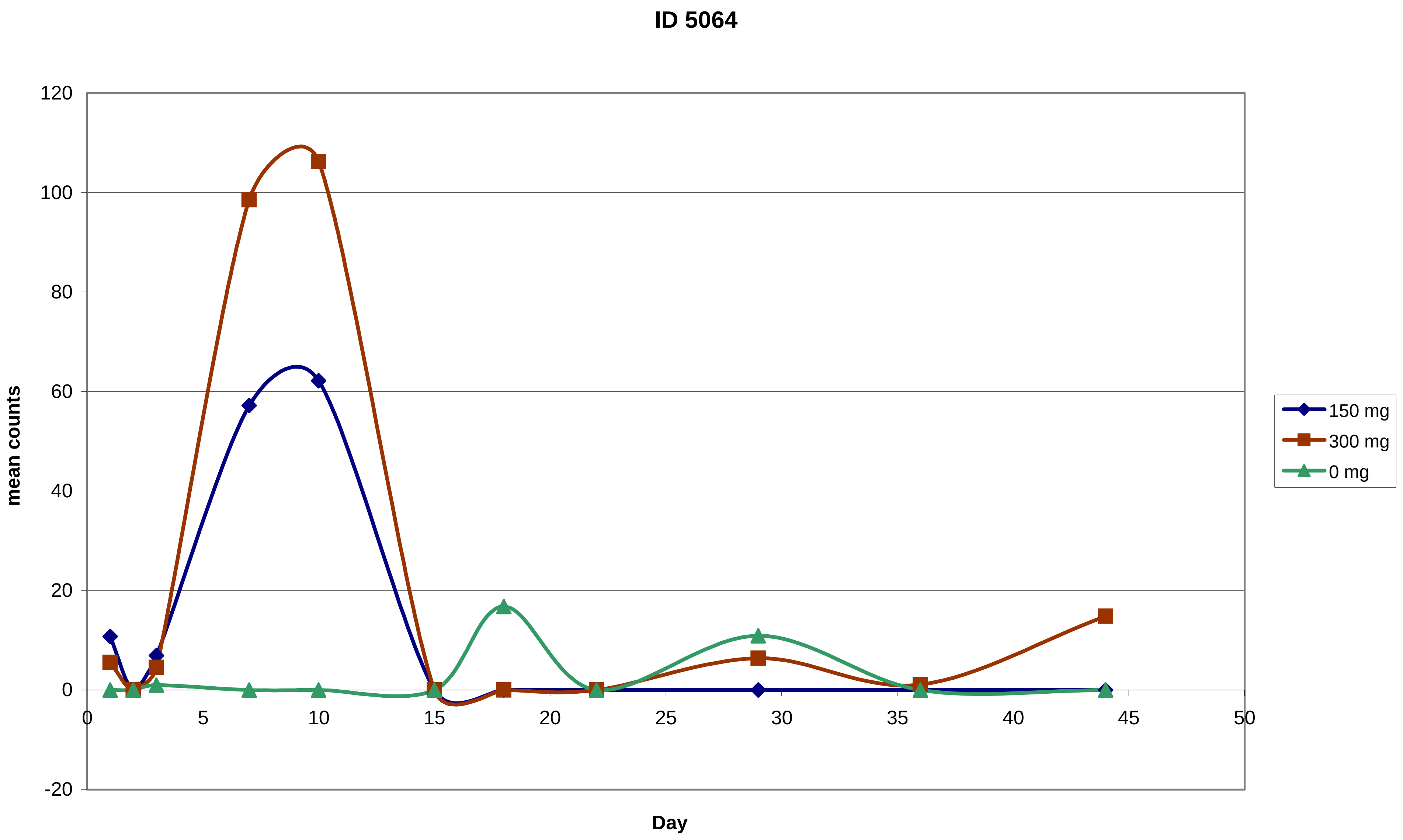

ID 5064
mean counts
120
100
80
60
40
20
0
-20
0
5
10
15
20
25
30
35
40
45
50
Day
150 mg
300 mg
0 mg

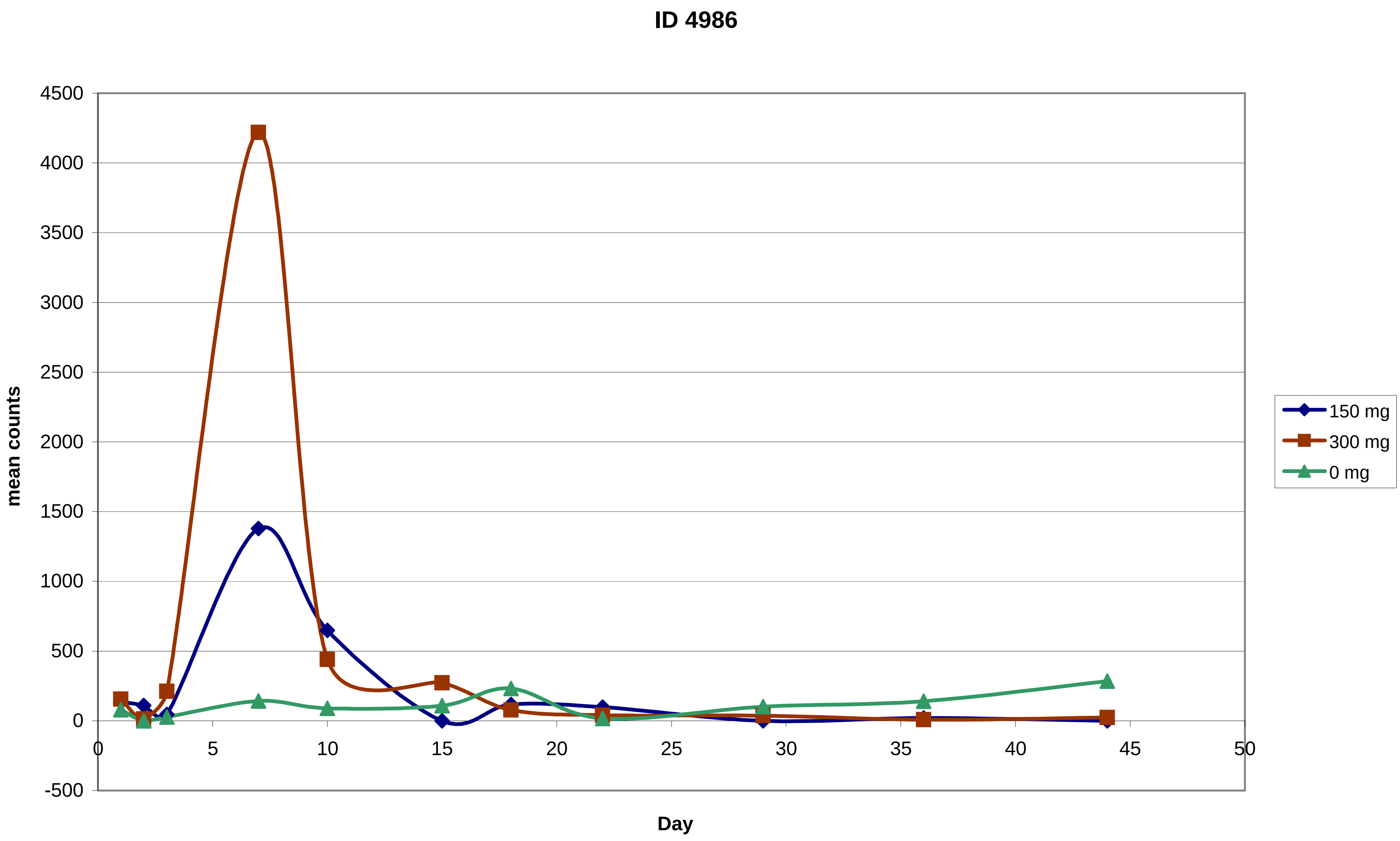

ID 4986
mean counts
4500
4000
3500
3000
2500
2000
1500
1000
500
0
-500
0
5
10
15
20
25
30
35
40
45
50
Day
150 mg
300 mg
0 mg

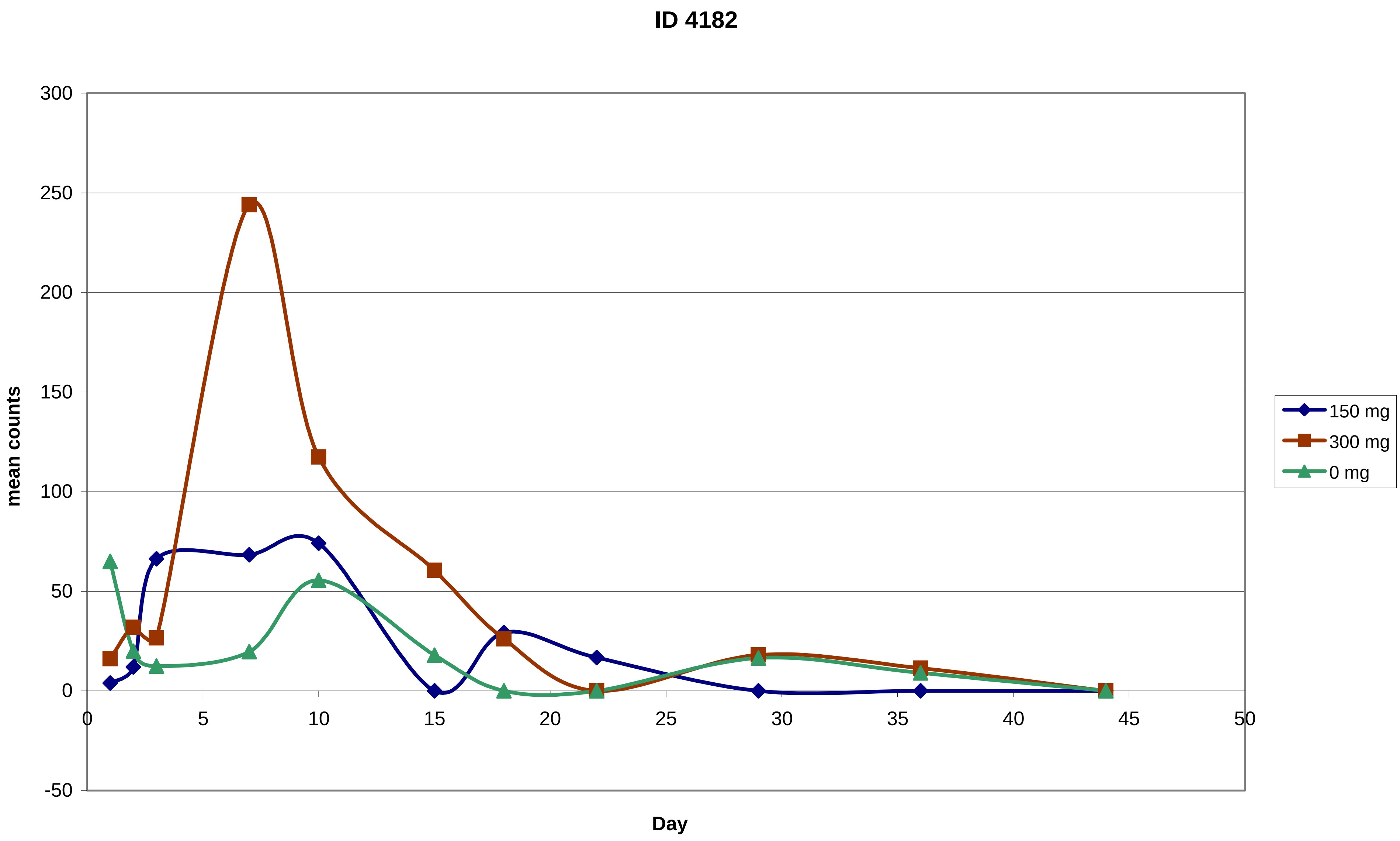

ID 4182
mean counts
Day
300
250
200
150
100
50
0
-50
0
5
10
15
20
25
30
35
40
45
50
150 mg
300 mg
0 mg

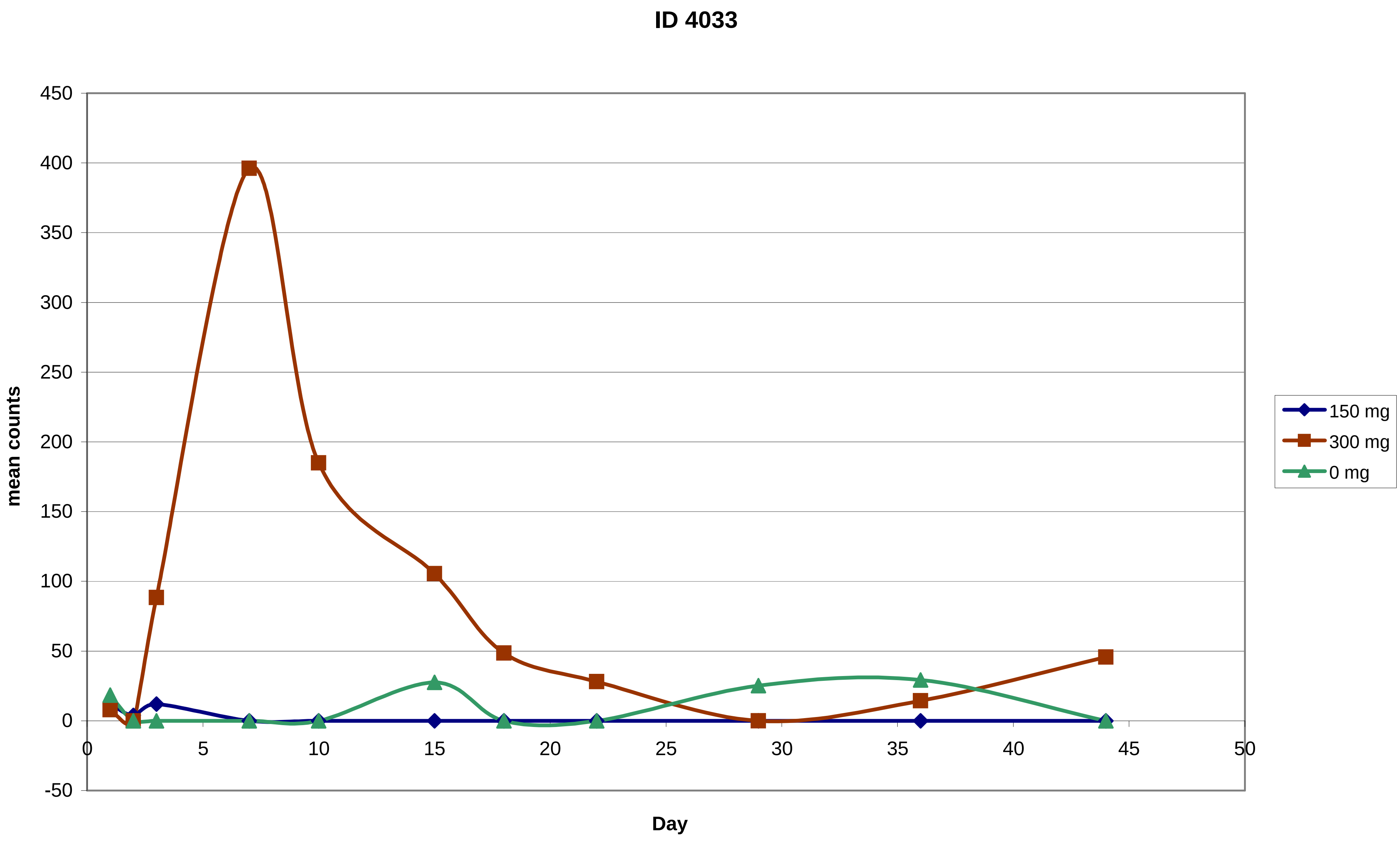
ID 4033
mean counts
450
400
350
300
250
200
150
100
50
0
-50
0
5
10
15
20
25
30
35
40
45
50
Day
150 mg
300 mg
0 mg

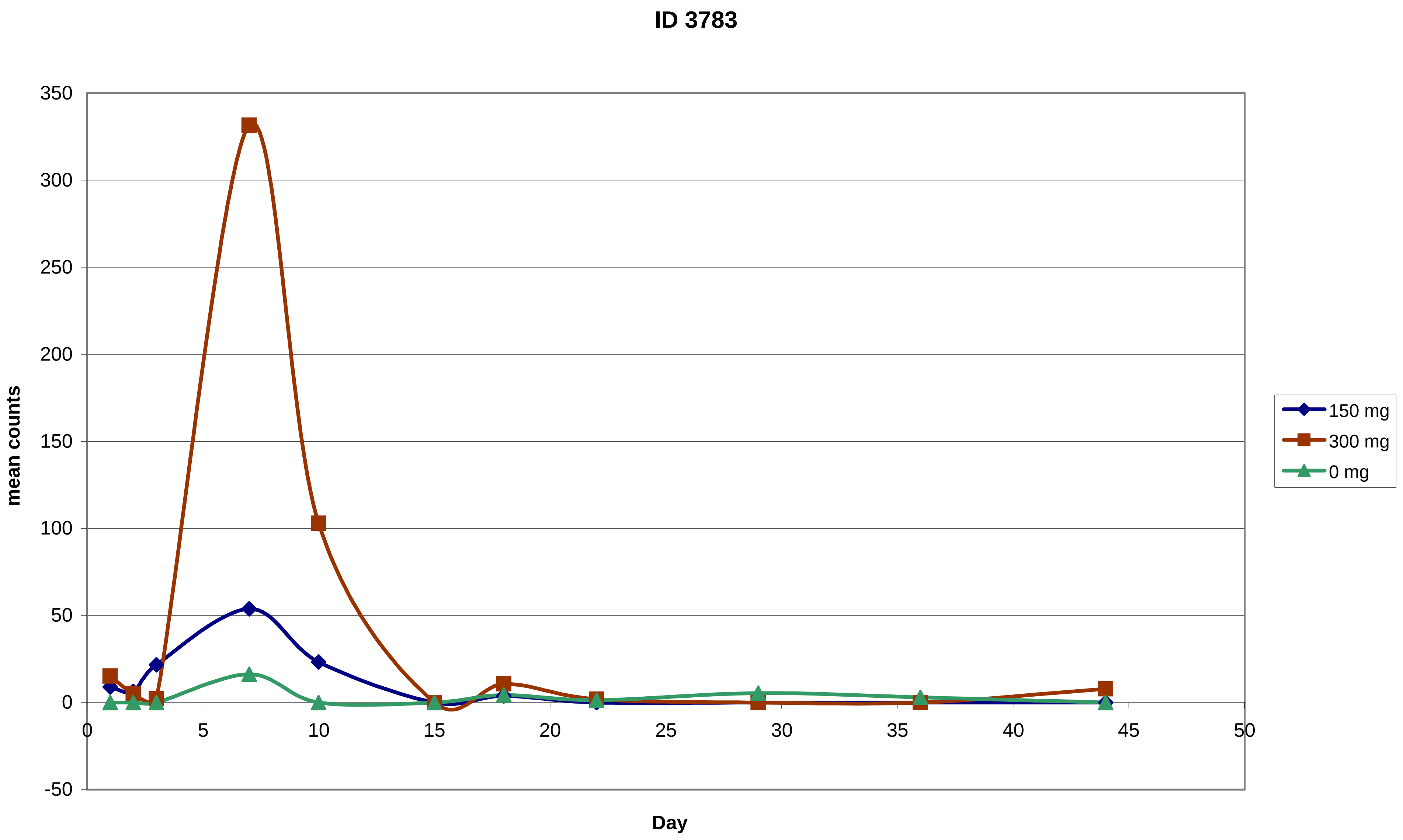
ID 3783
mean counts
Day
350
300
250
200
150
100
50
0
-50
0
5
10
15
20
25
30
35
40
45
50
150 mg
300 mg
0 mg

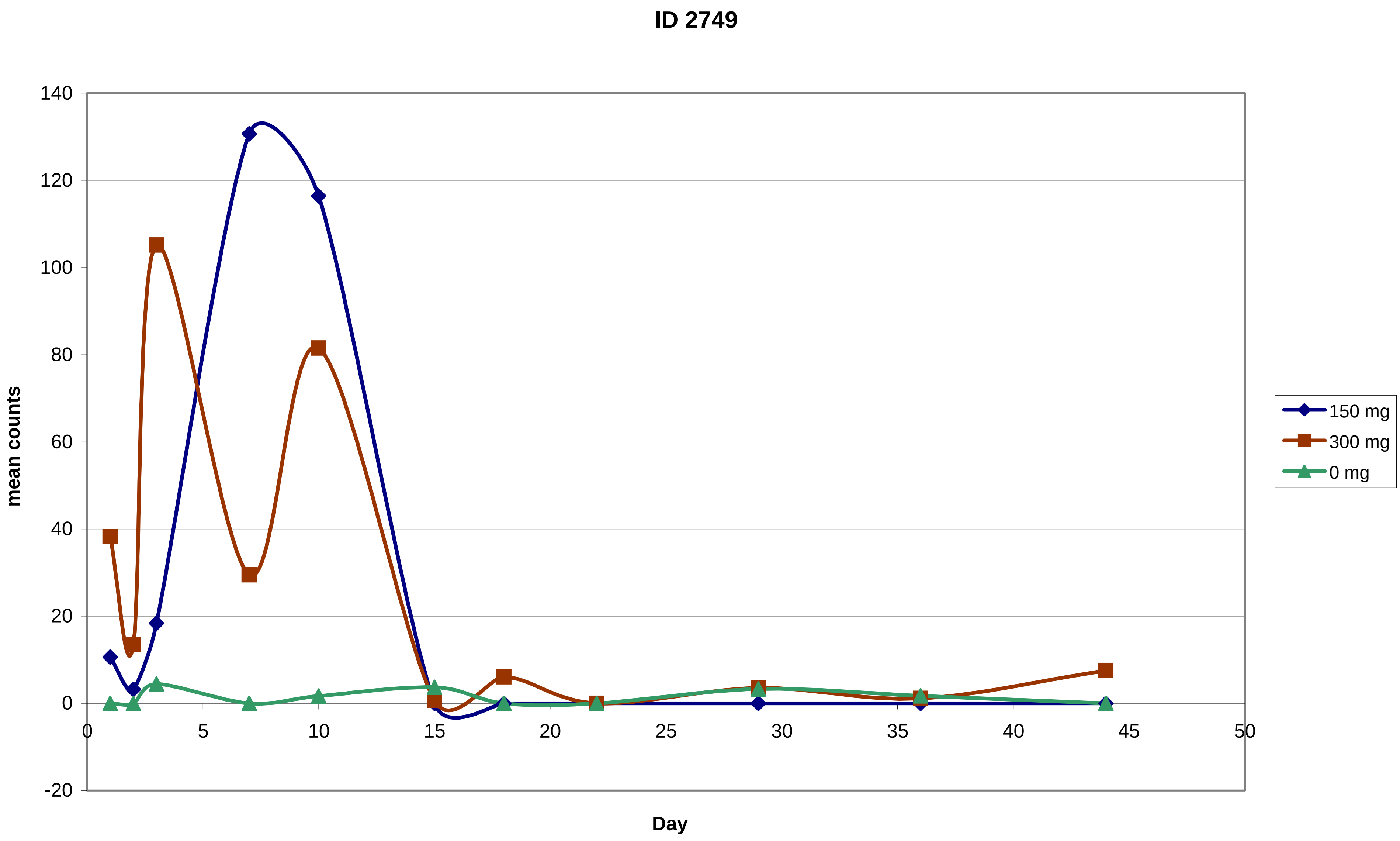
ID 2749
mean counts
140
120
100
80
60
40
20
0
-20
0
5
10
15
20
25
30
35
40
45
50
Day
150 mg
300 mg
0 mg

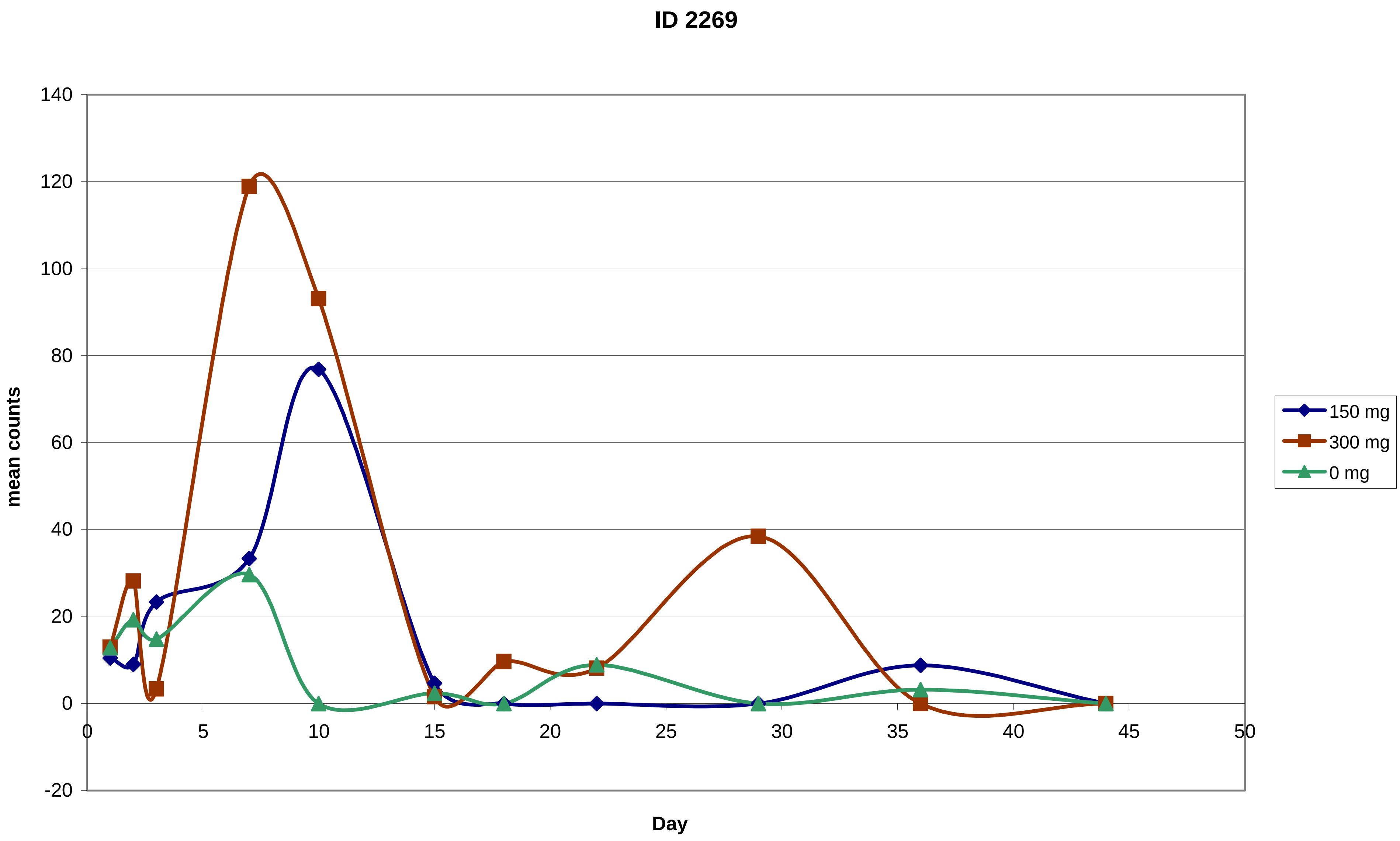
ID 2269
140
120
100
80
60
40
20
0
-20
mean counts
0
5
10
15
20
25
30
35
40
45
50
Day
150 mg
300 mg
0 mg

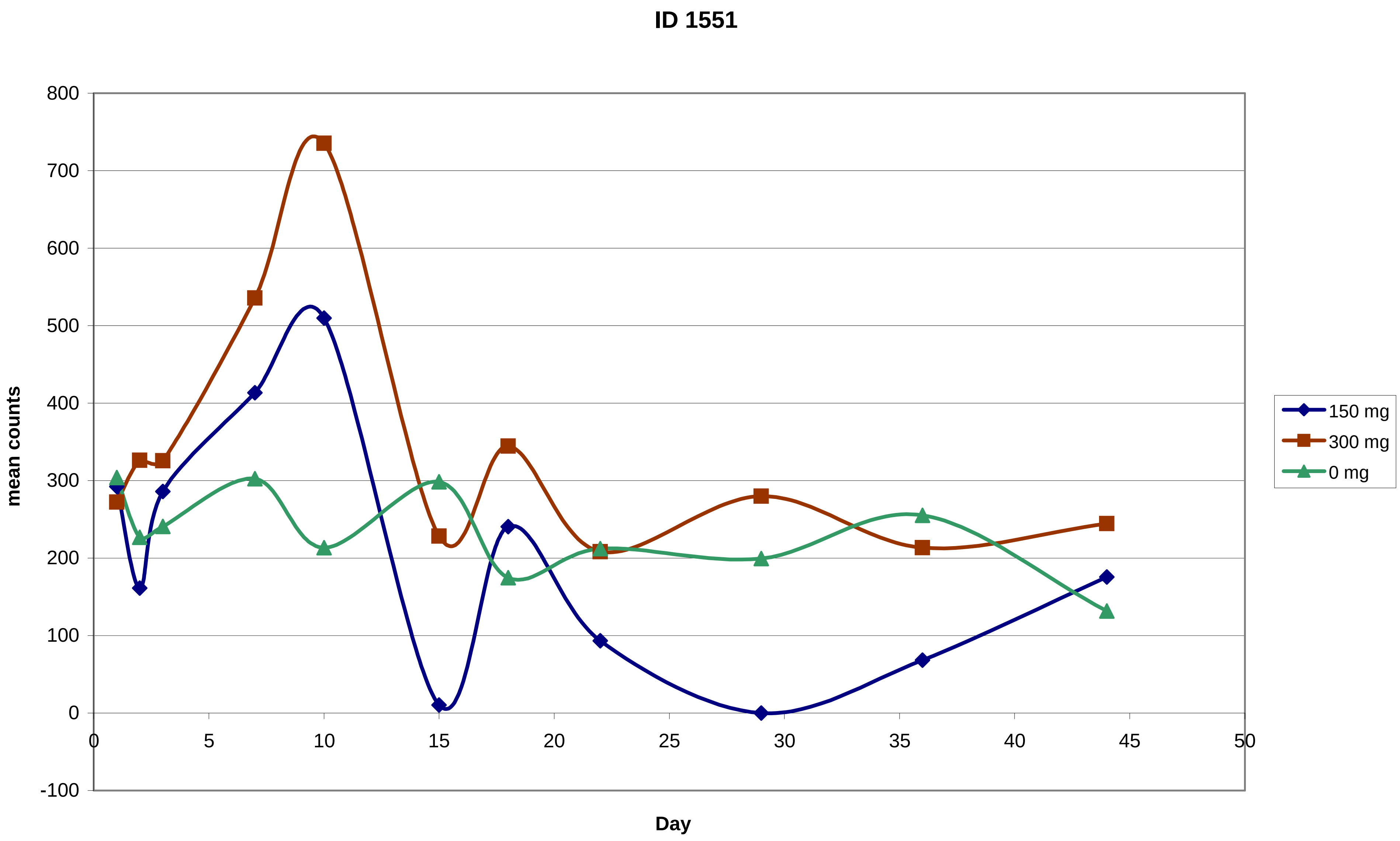

ID 1551
800
700
600
500
400
300
200
100
0
-100
mean counts
0
5
10
15
20
25
30
35
40
45
50
Day
150 mg
300 mg
0 mg

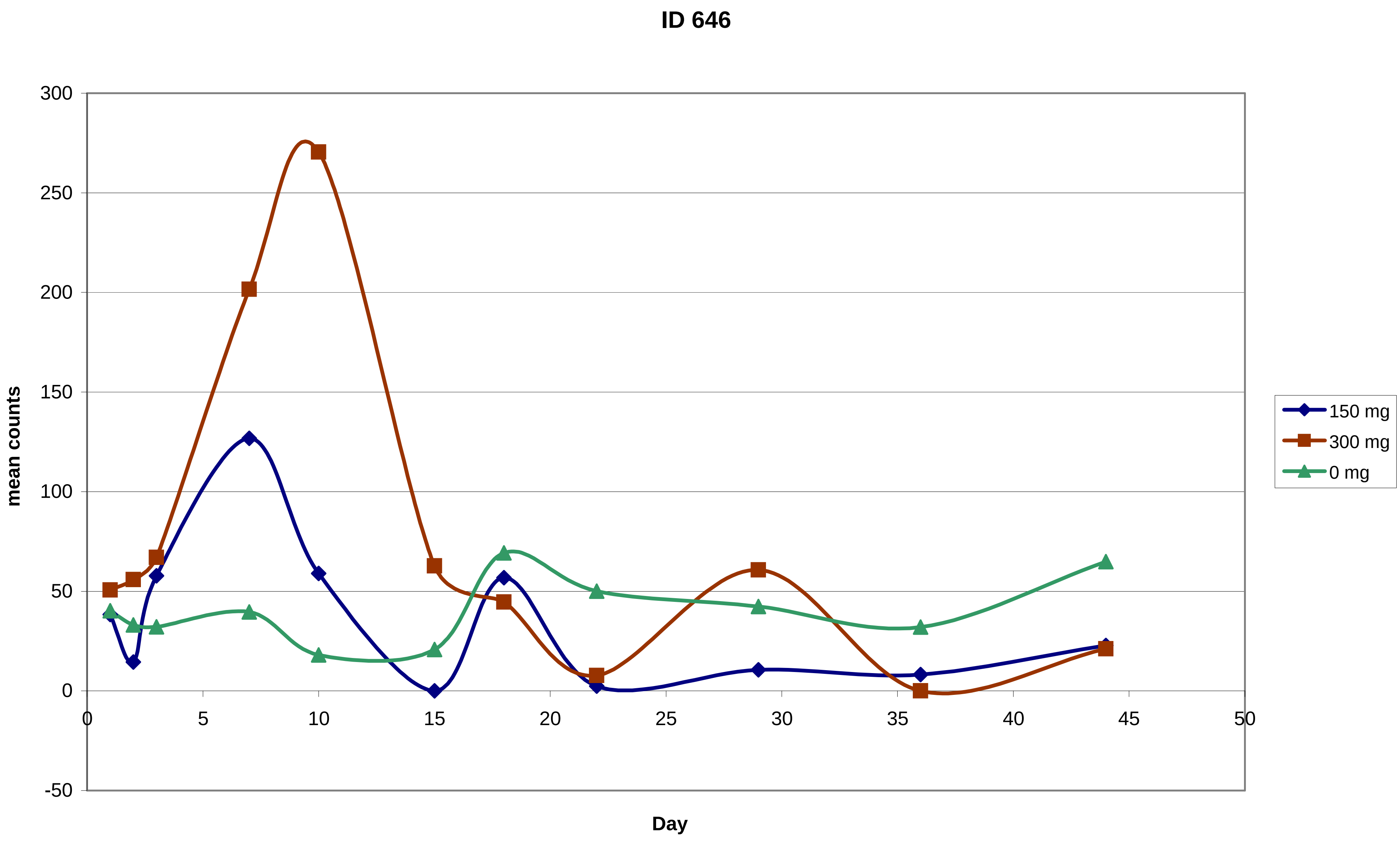

ID 646
mean counts
300
250
200
150
100
50
0
-50
0
5
10
15
20
25
30
35
40
45
50
Day
150 mg
300 mg
0 mg

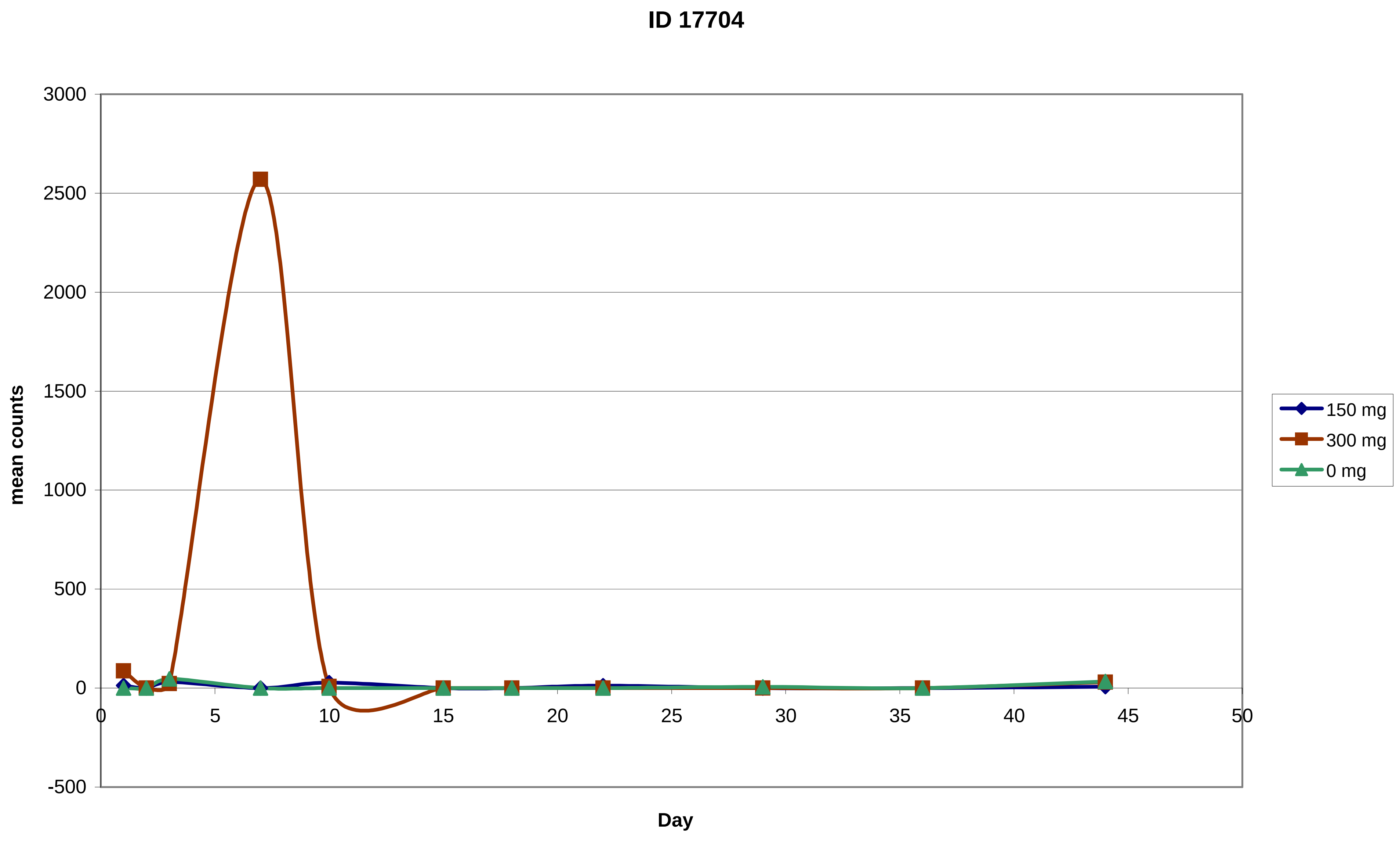

ID 17704
mean counts
3000
2500
2000
1500
1000
500
0
-500
0
5
10
15
20
25
30
35
40
45
50
Day
150 mg
300 mg
0 mg

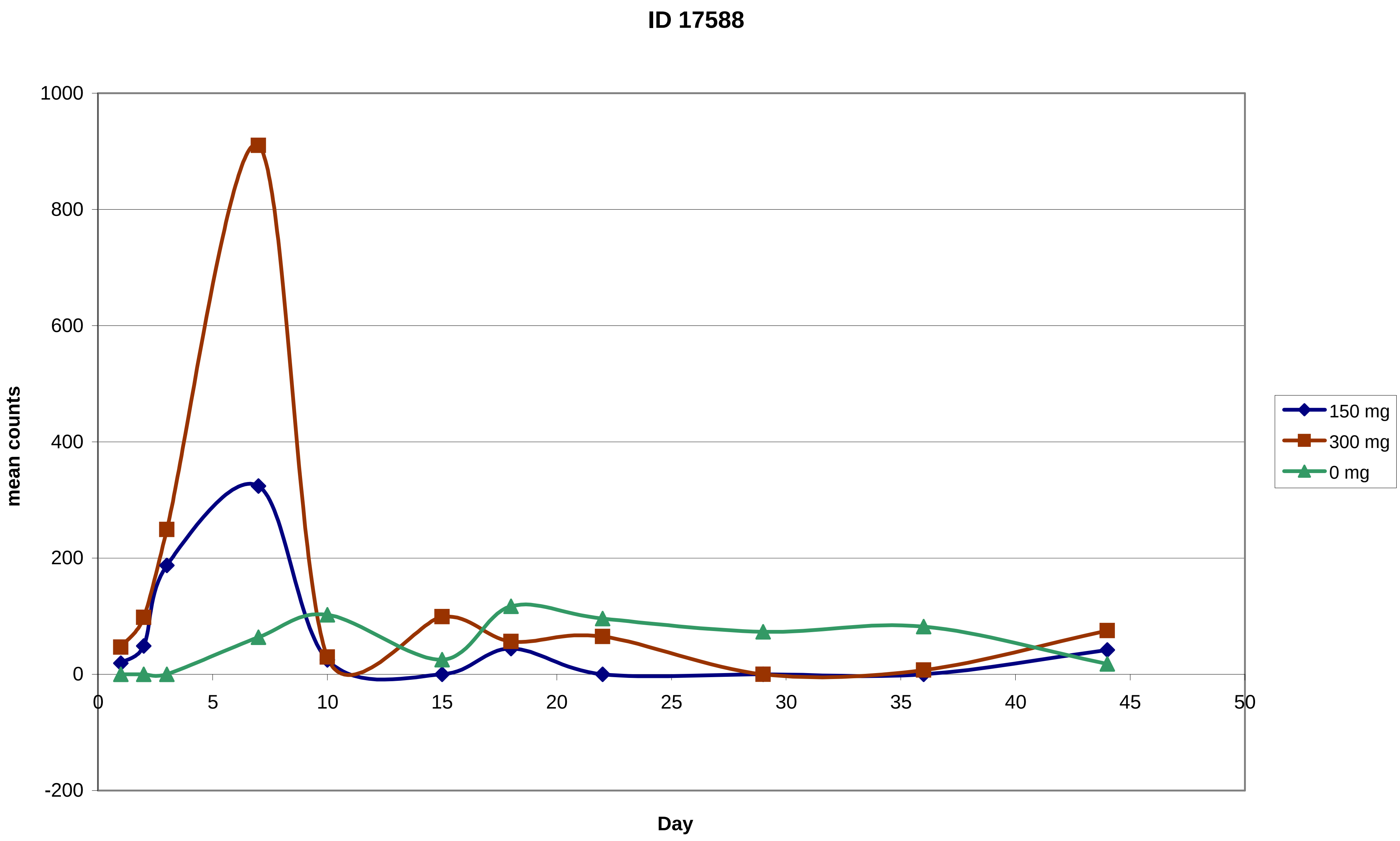

ID 17588
mean counts
1000
800
600
400
200
0
-200
0
5
10
15
20
25
30
35
40
45
50
Day
150 mg
300 mg
0 mg

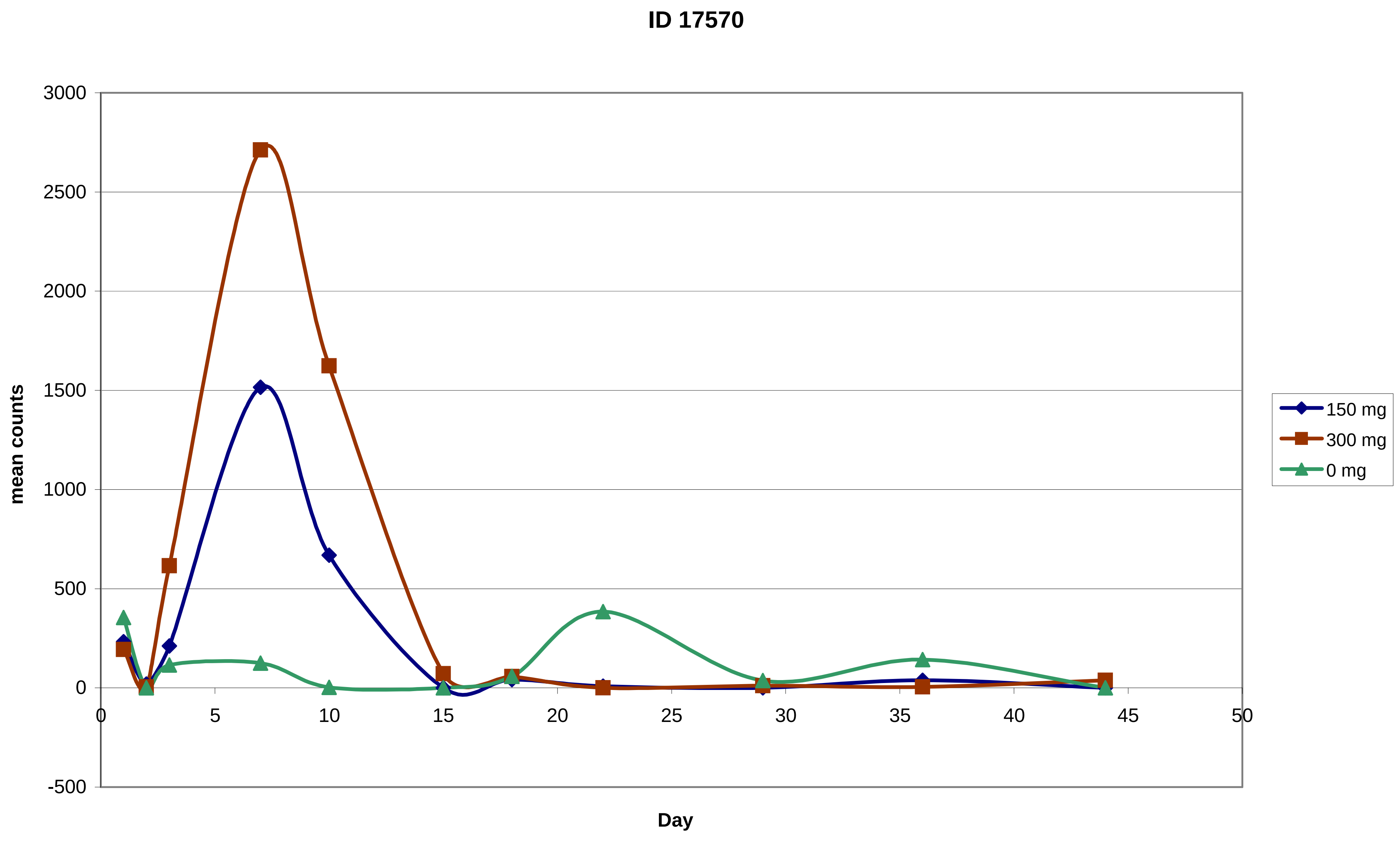
ID 17570
mean counts
Day
3000
2500
2000
1500
1000
500
0
-500
0
5
10
15
20
25
30
35
40
45
50
150 mg
300 mg
0 mg

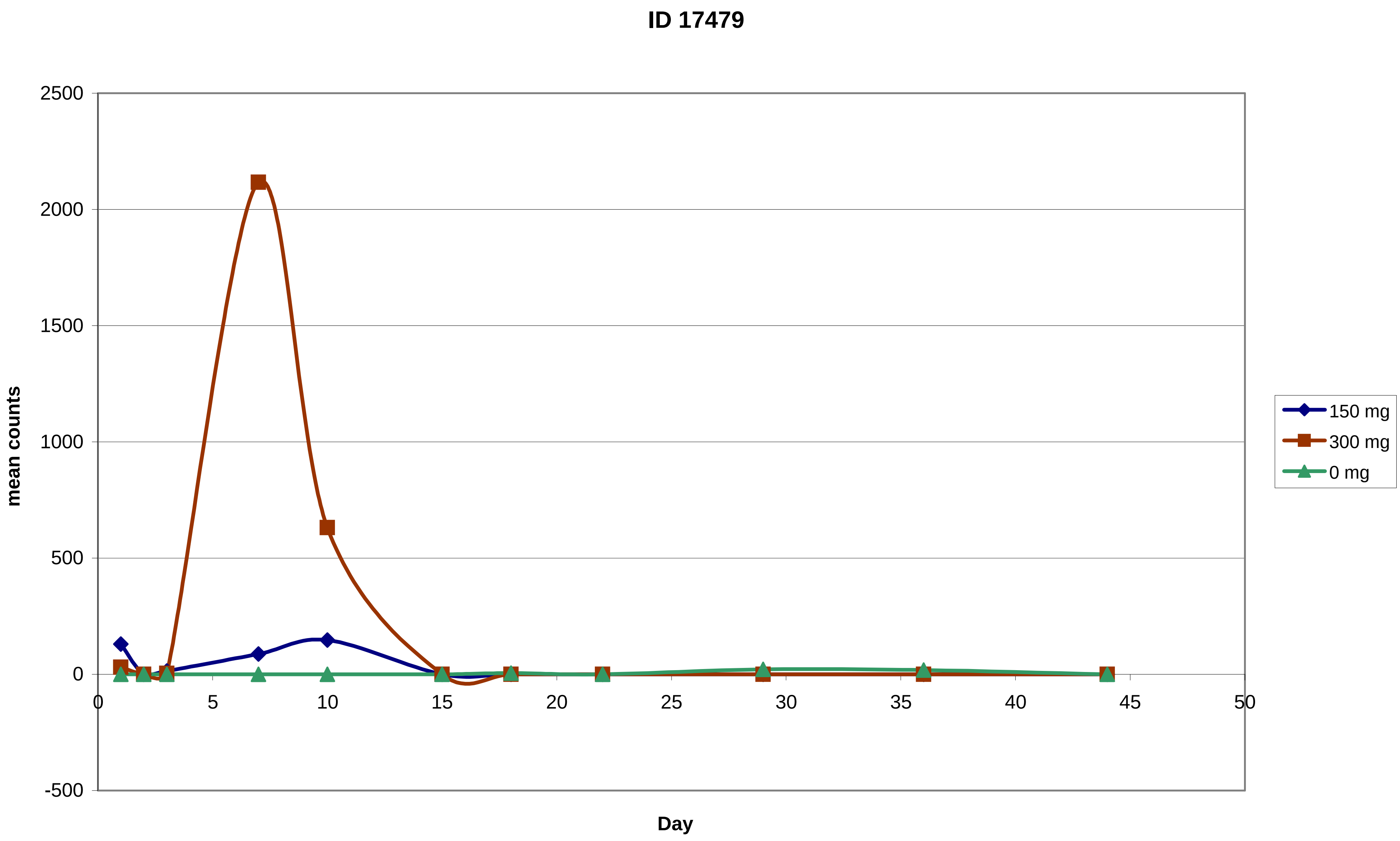

ID 17479
mean counts
2500
2000
1500
1000
500
0
-500
0
5
10
15
20
25
30
35
40
45
50
Day
150 mg
300 mg
0 mg

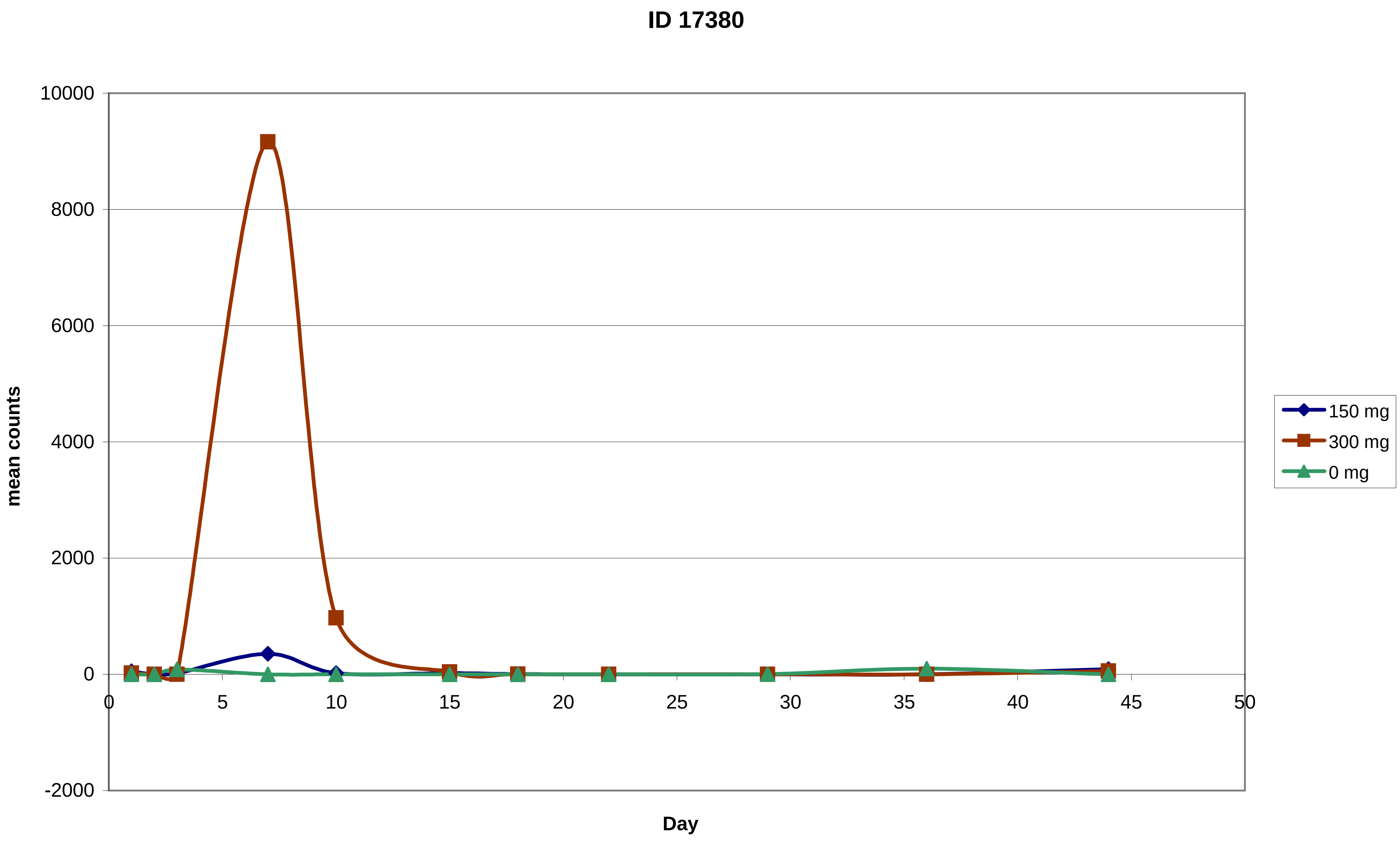
ID 17380
mean counts
10000
8000
6000
4000
2000
0
-2000
0
5
10
15
20
25
30
35
40
45
50
Day
150 mg
300 mg
0 mg

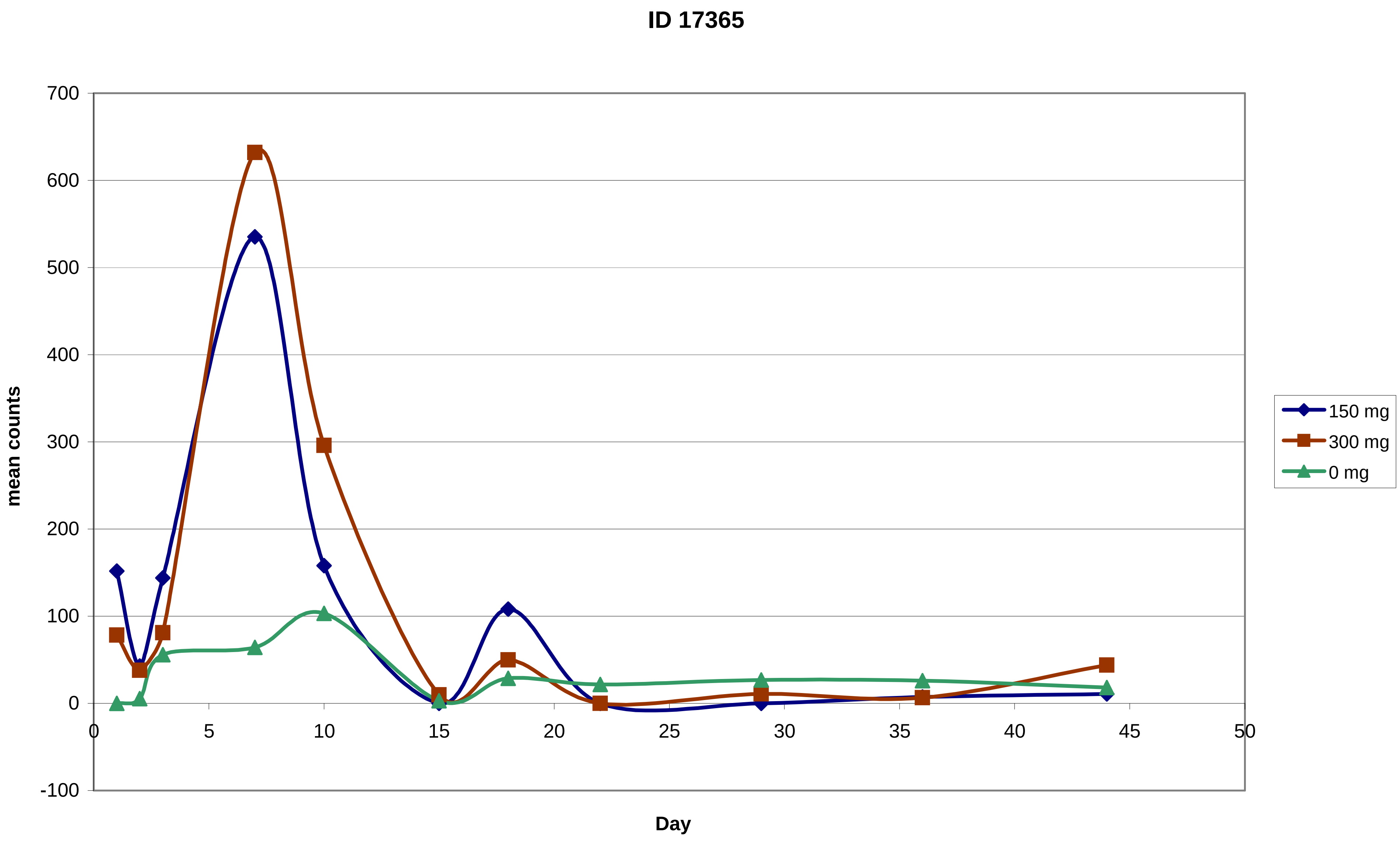
ID 17365
mean counts
700
600
500
400
300
200
100
0
-100
0
5
10
15
20
25
30
35
40
45
50
Day
150 mg
300 mg
0 mg

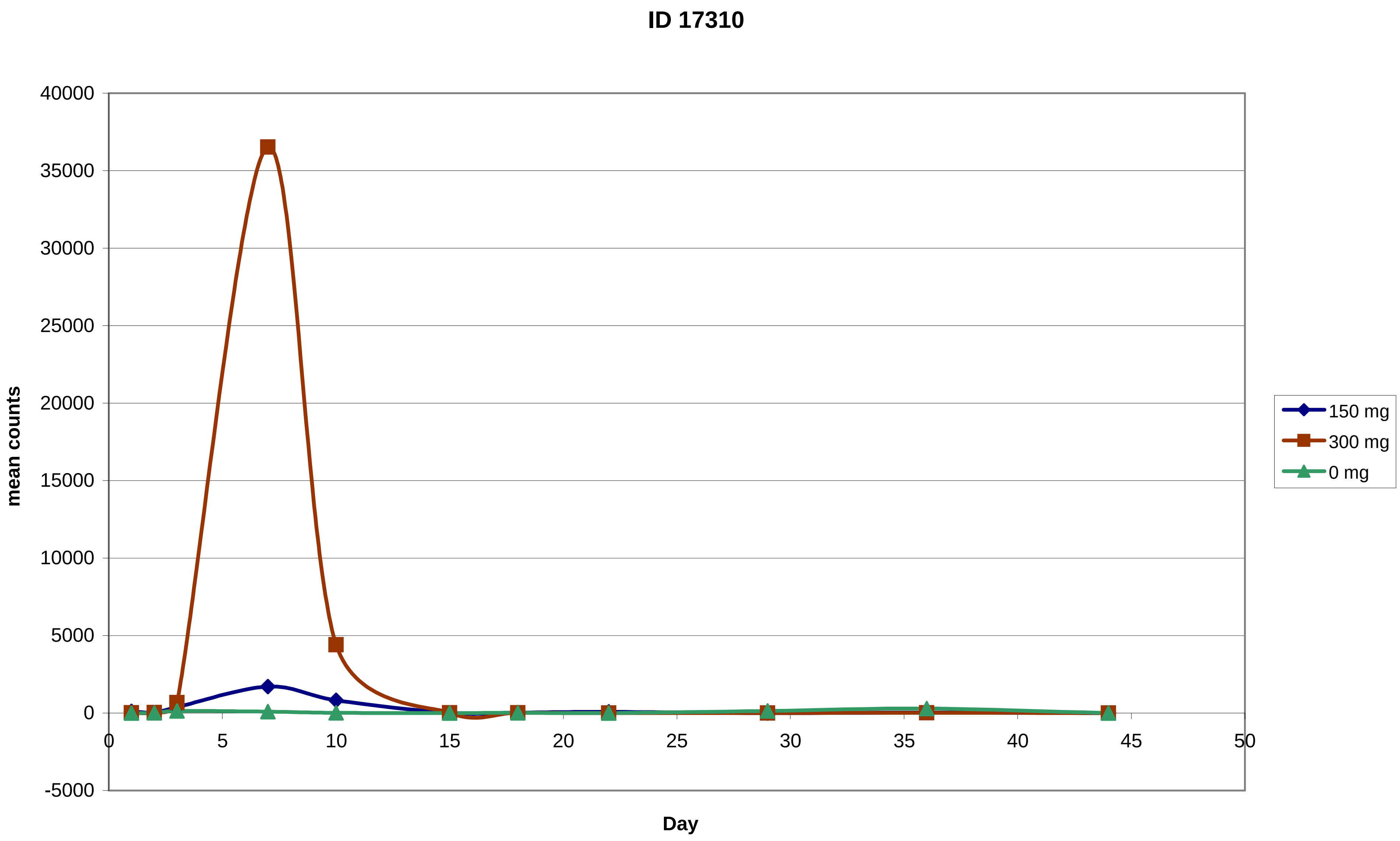

ID 17310
mean counts
40000
35000
30000
25000
20000
15000
10000
5000
0
-5000
0
5
10
15
20
25
30
35
40
45
50
Day
150 mg
300 mg
0 mg

ID 17163

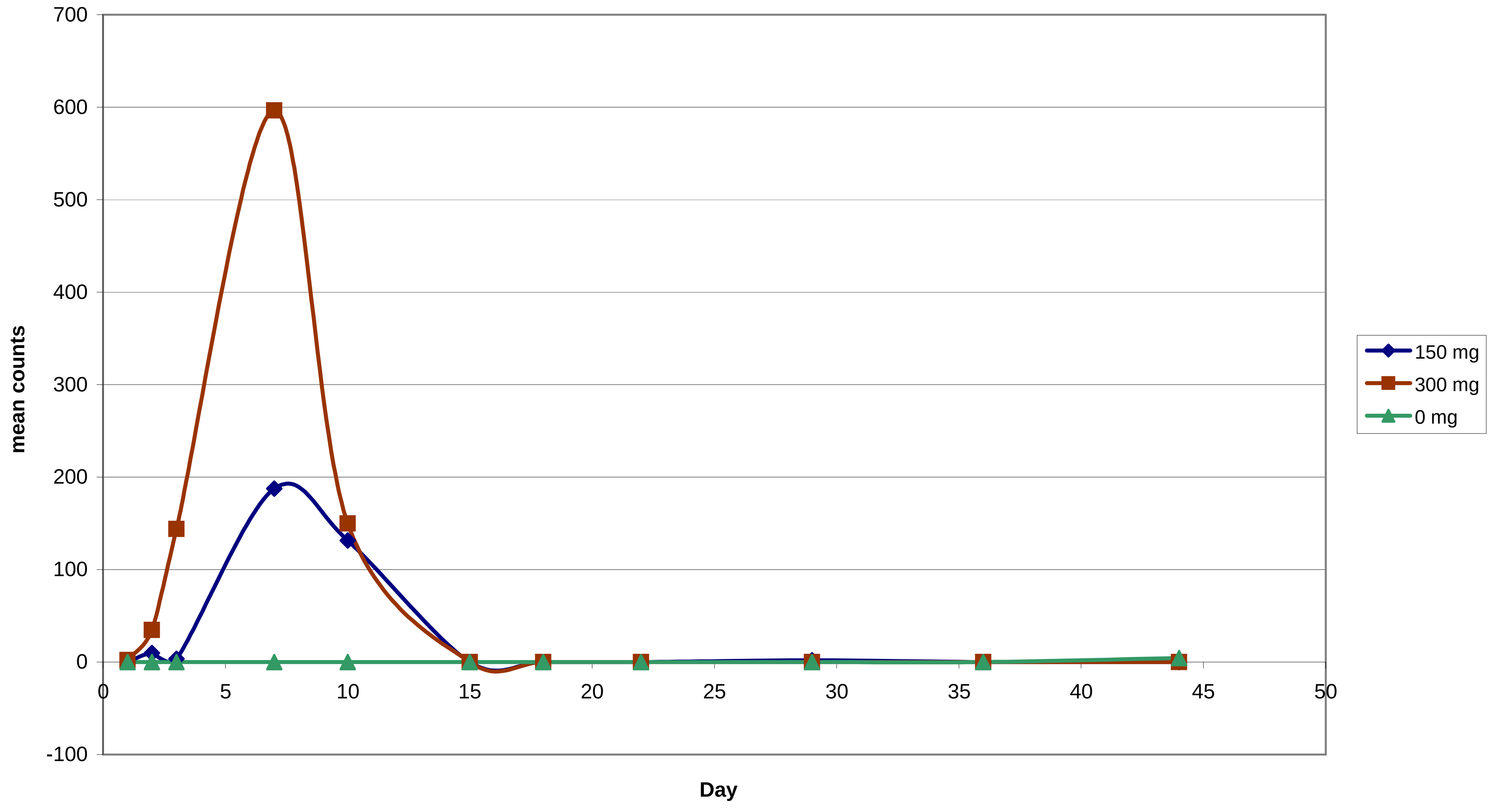

700
600
500
400
300
200
100
0
-100
mean counts
0
5
10
15
20
25
30
35
40
45
50
Day
150 mg
300 mg
0 mg

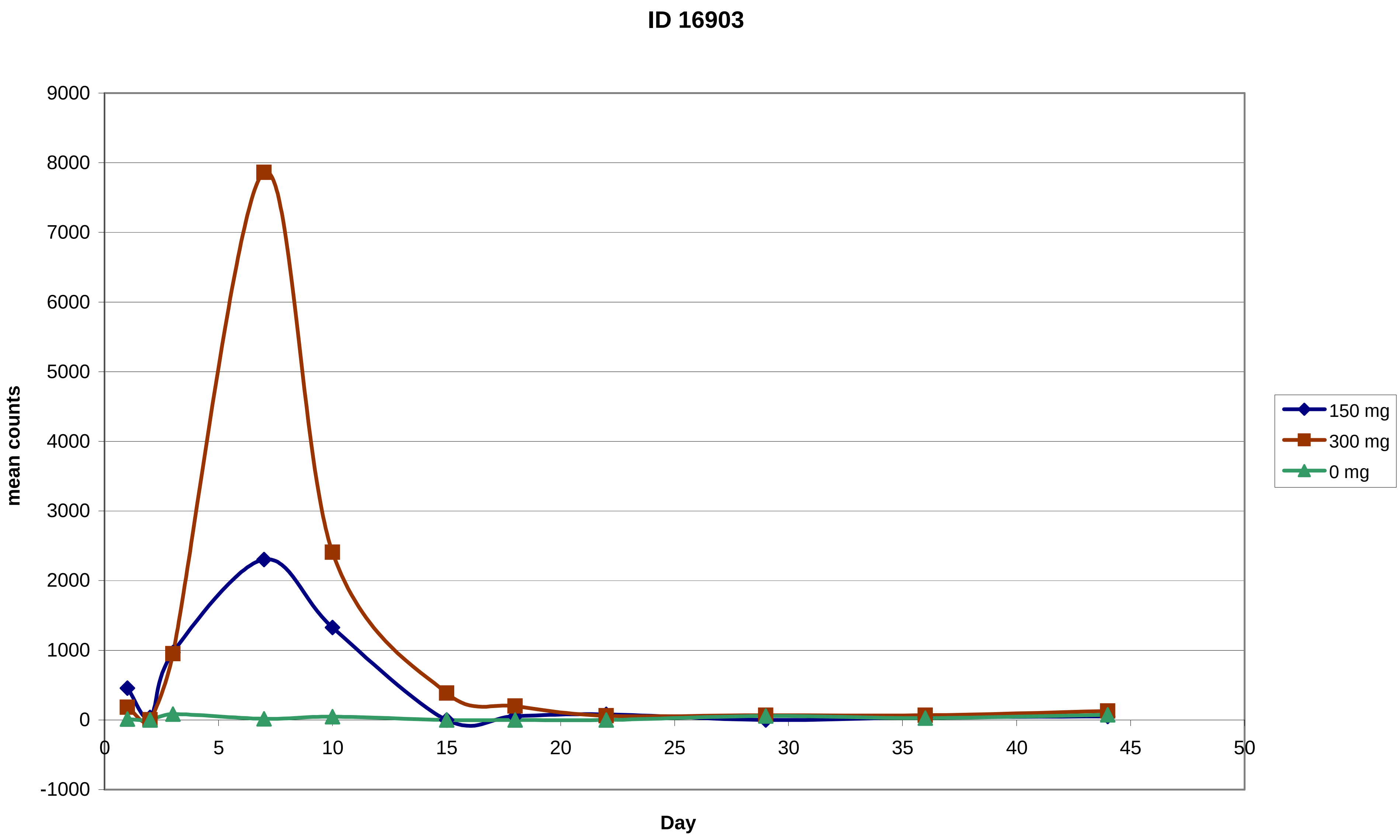
ID 16903
mean counts
9000
8000
7000
6000
5000
4000
3000
2000
1000
0
-1000
0
5
10
15
20
25
30
35
40
45
50
Day
150 mg
300 mg
0 mg

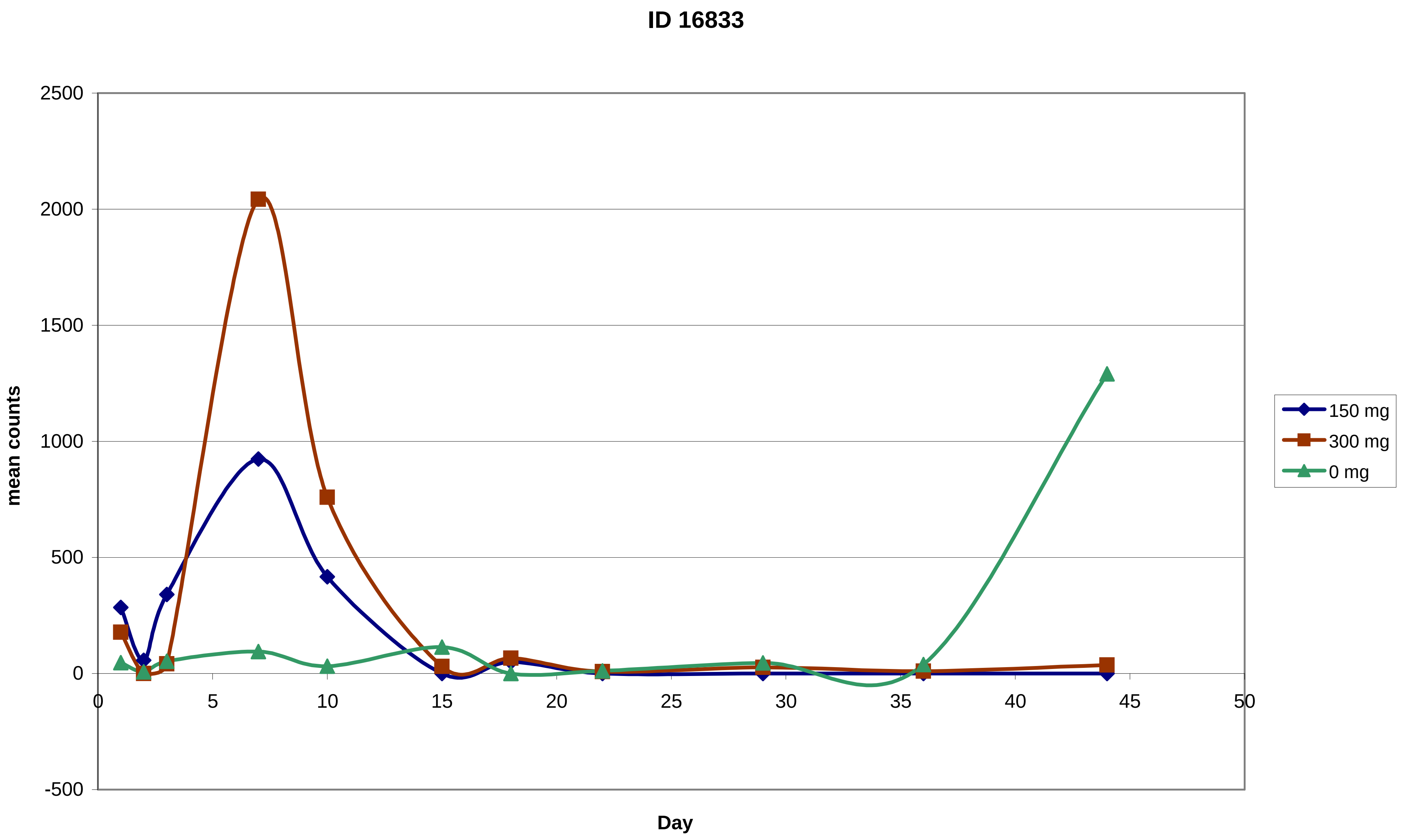

ID 16833
mean counts
2500
2000
1500
1000
500
0
-500
0
5
10
15
20
25
30
35
40
45
50
Day
150 mg
300 mg
0 mg

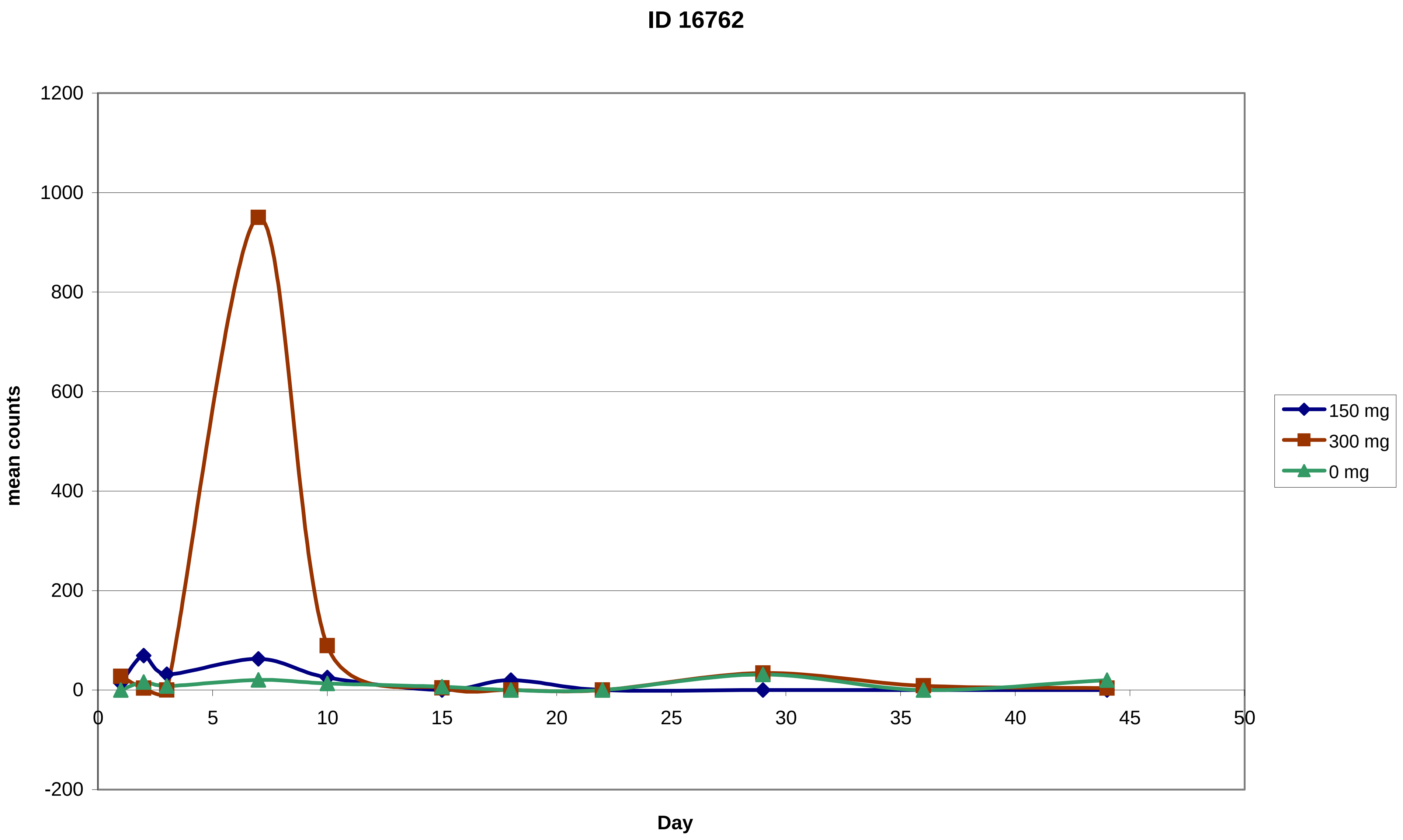
ID 16762
1200
1000
800
600
400
200
0
-200
mean counts
0
5
10
15
20
25
30
35
40
45
50
Day
150 mg
300 mg
0 mg

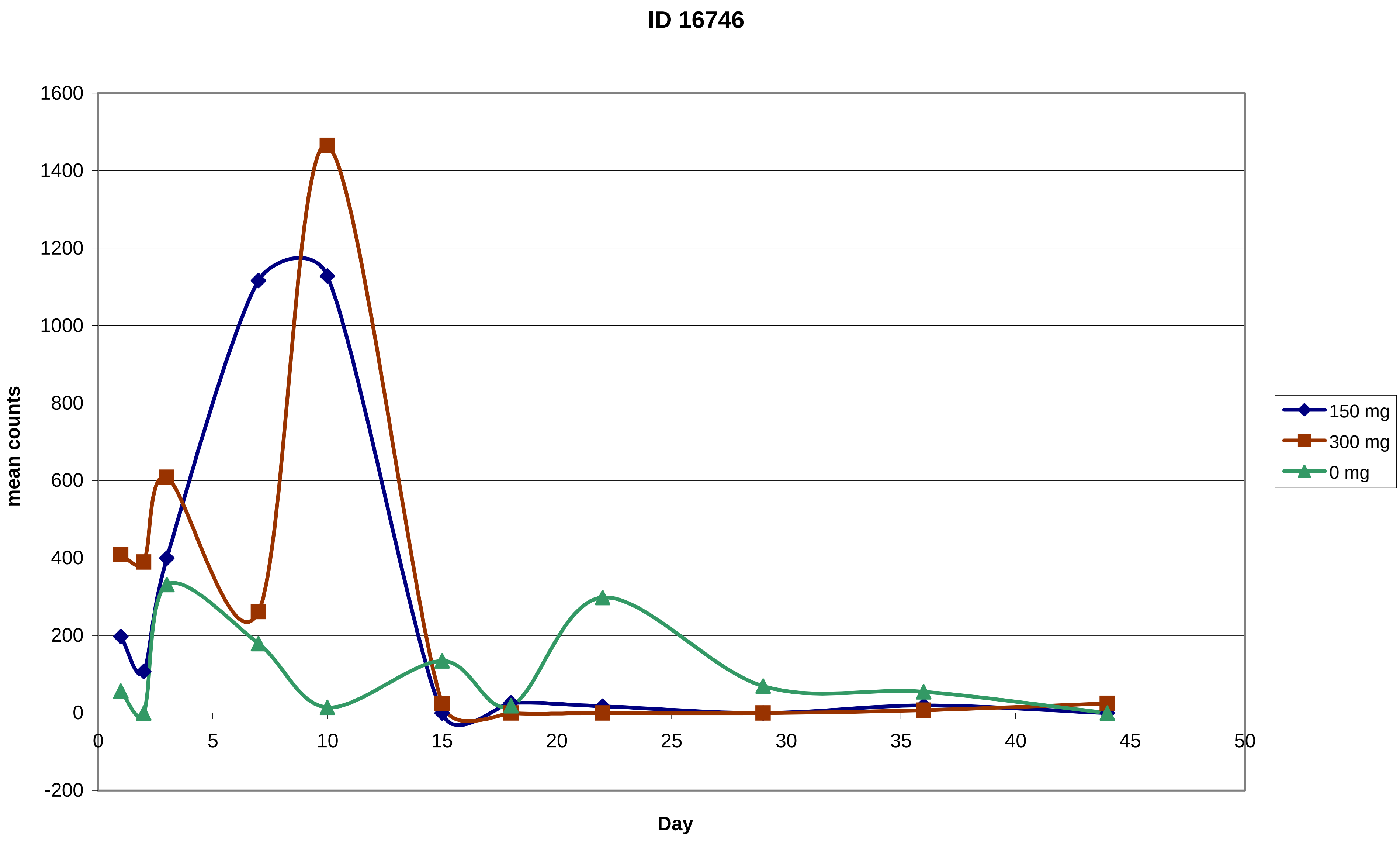
ID 16746
mean counts
Day
1600
1400
1200
1000
800
600
400
200
0
-200
0
5
10
15
20
25
30
35
40
45
50
150 mg
300 mg
0 mg

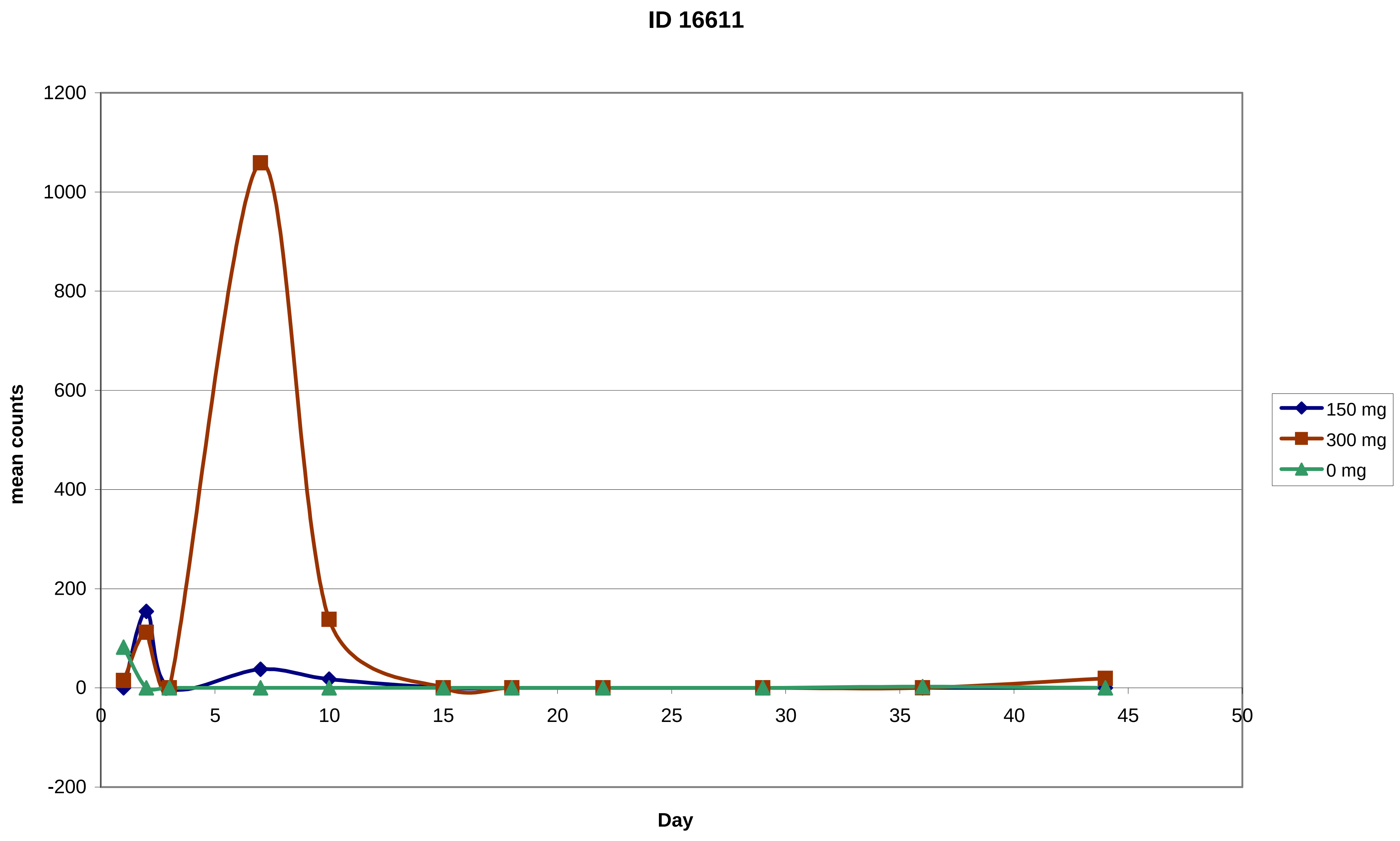

ID 16611
mean counts
1200
1000
800
600
400
200
0
-200
0
5
10
15
20
25
30
35
40
45
50
Day
150 mg
300 mg
0 mg

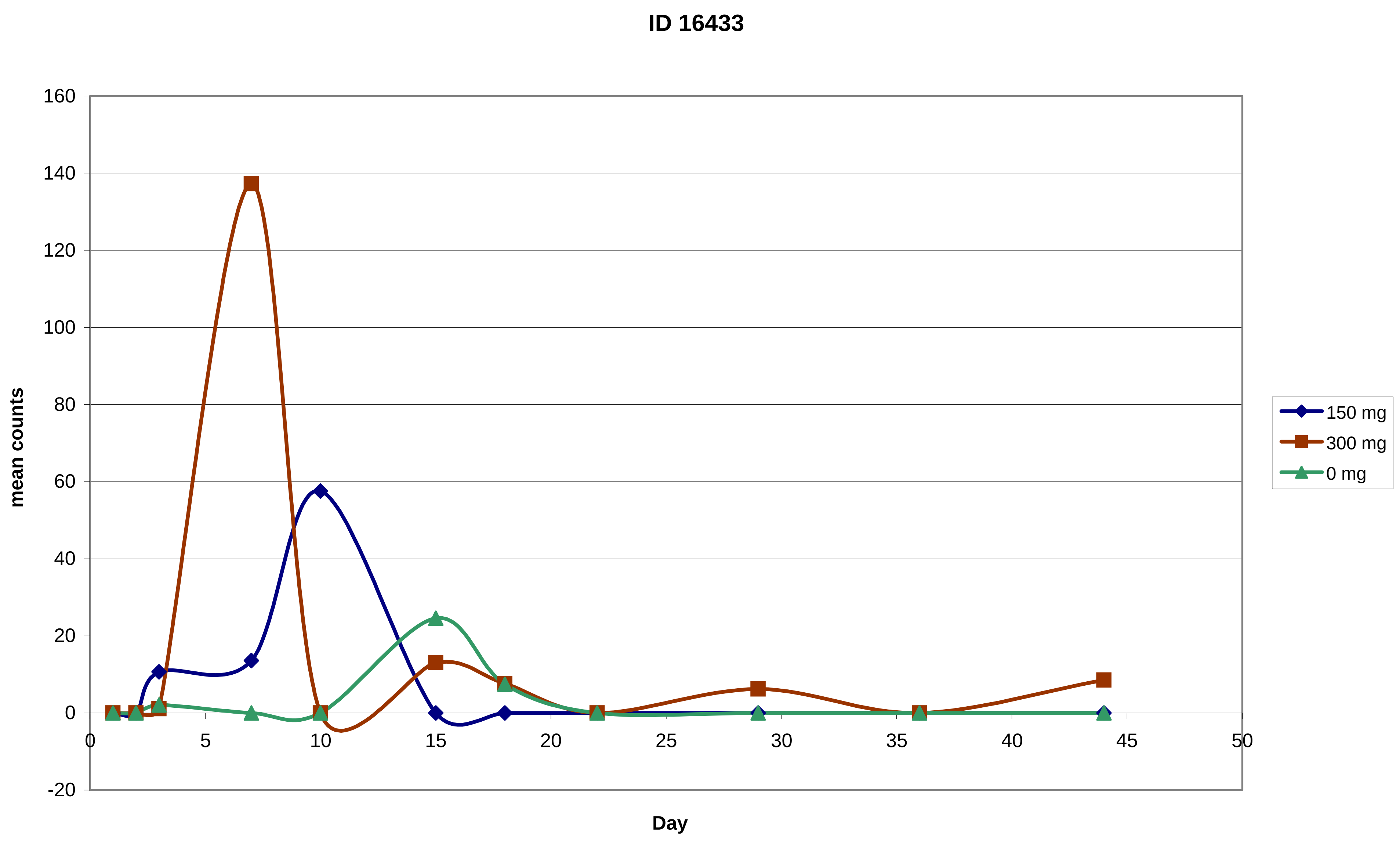

ID 16433
160
140
120
100
80
60
40
20
0
-20
mean counts
0
5
10
15
20
25
30
35
40
45
50
Day
150 mg
300 mg
0 mg

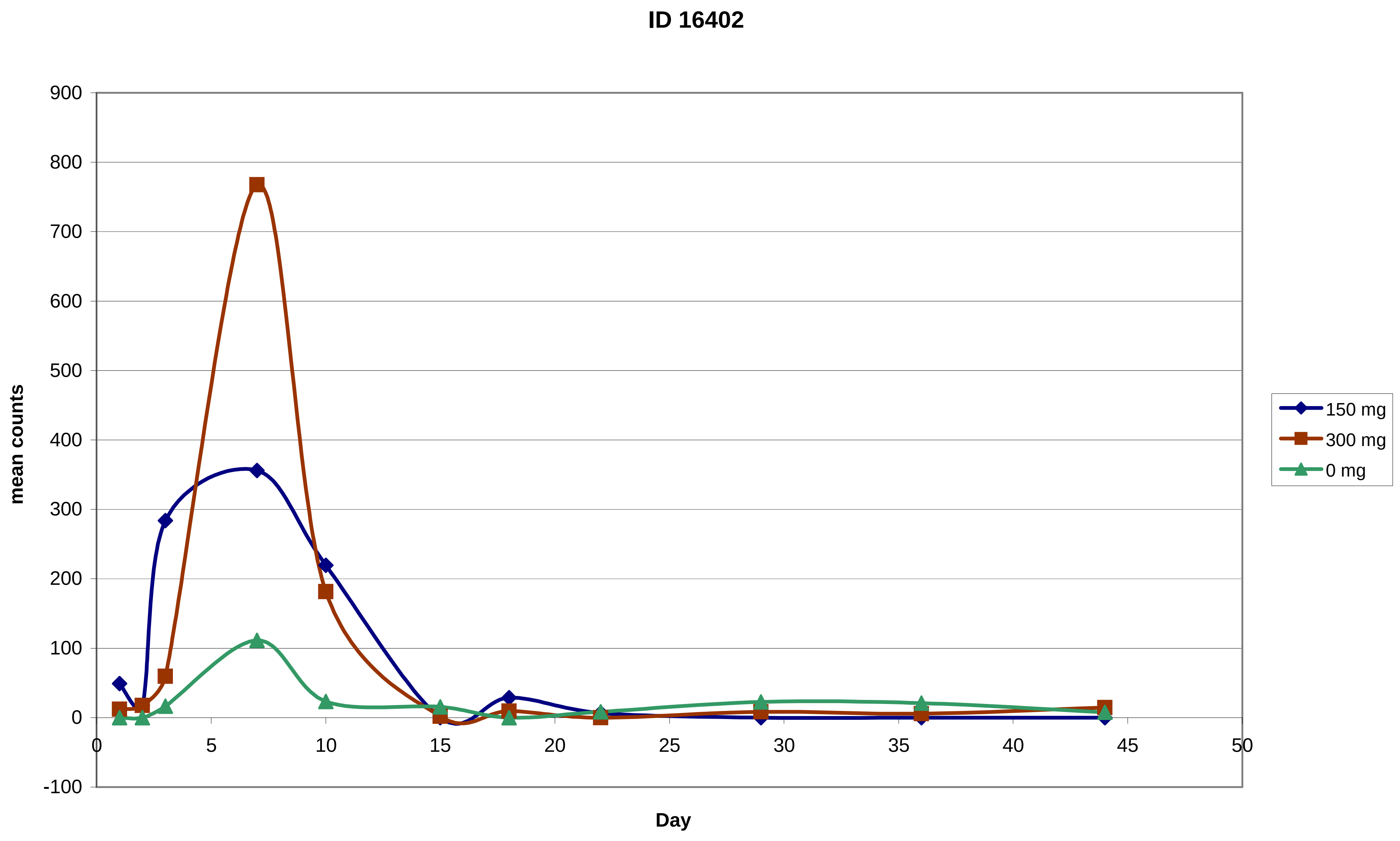
ID 16402
mean counts
Day
900
800
700
600
500
400
300
200
100
0
-100
0
5
10
15
20
25
30
35
40
45
50
150 mg
300 mg
0 mg

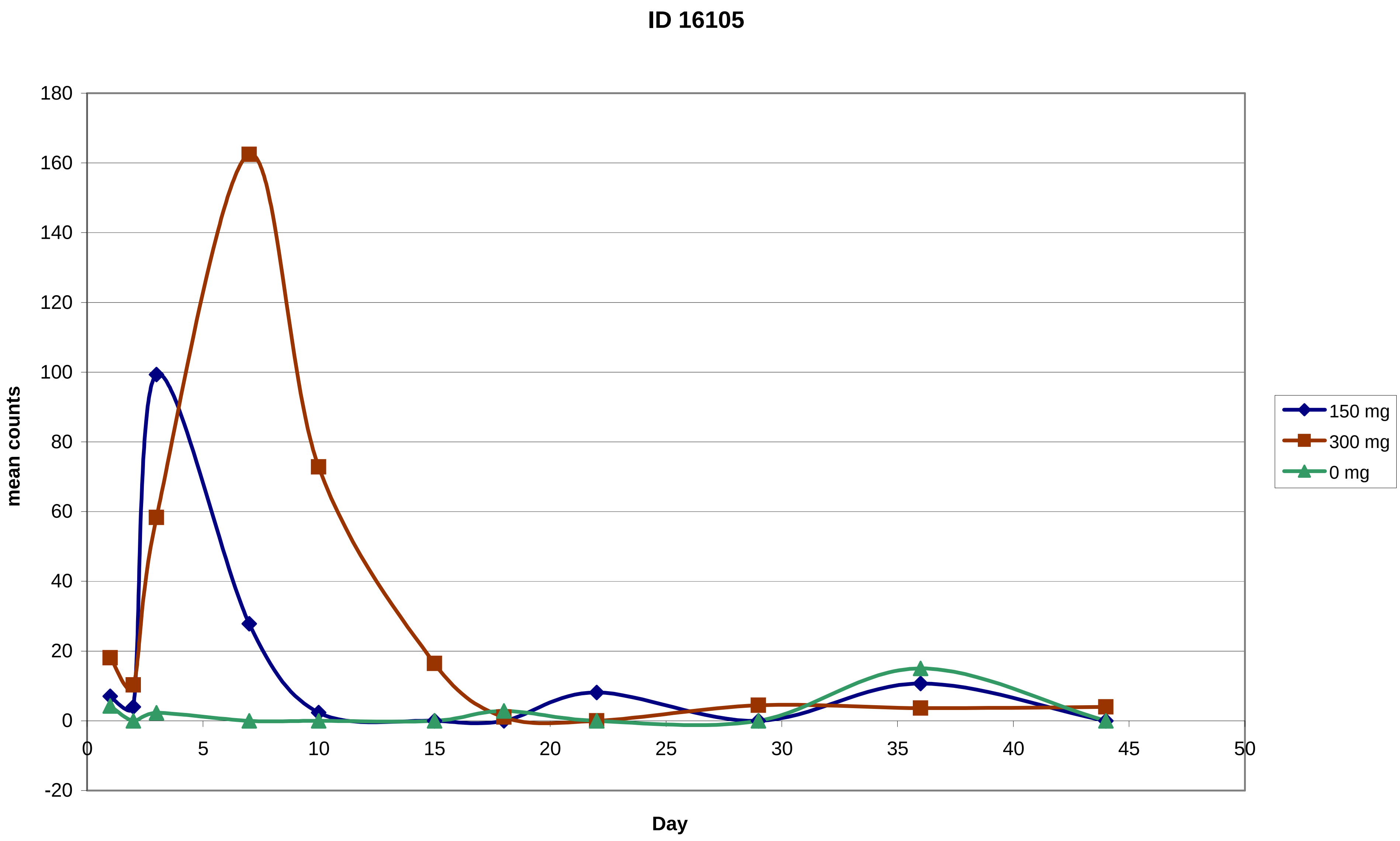

ID 16105
mean counts
Day
180
160
140
120
100
80
60
40
20
0
-20
0
5
10
15
20
25
30
35
40
45
50
150 mg
300 mg
0 mg

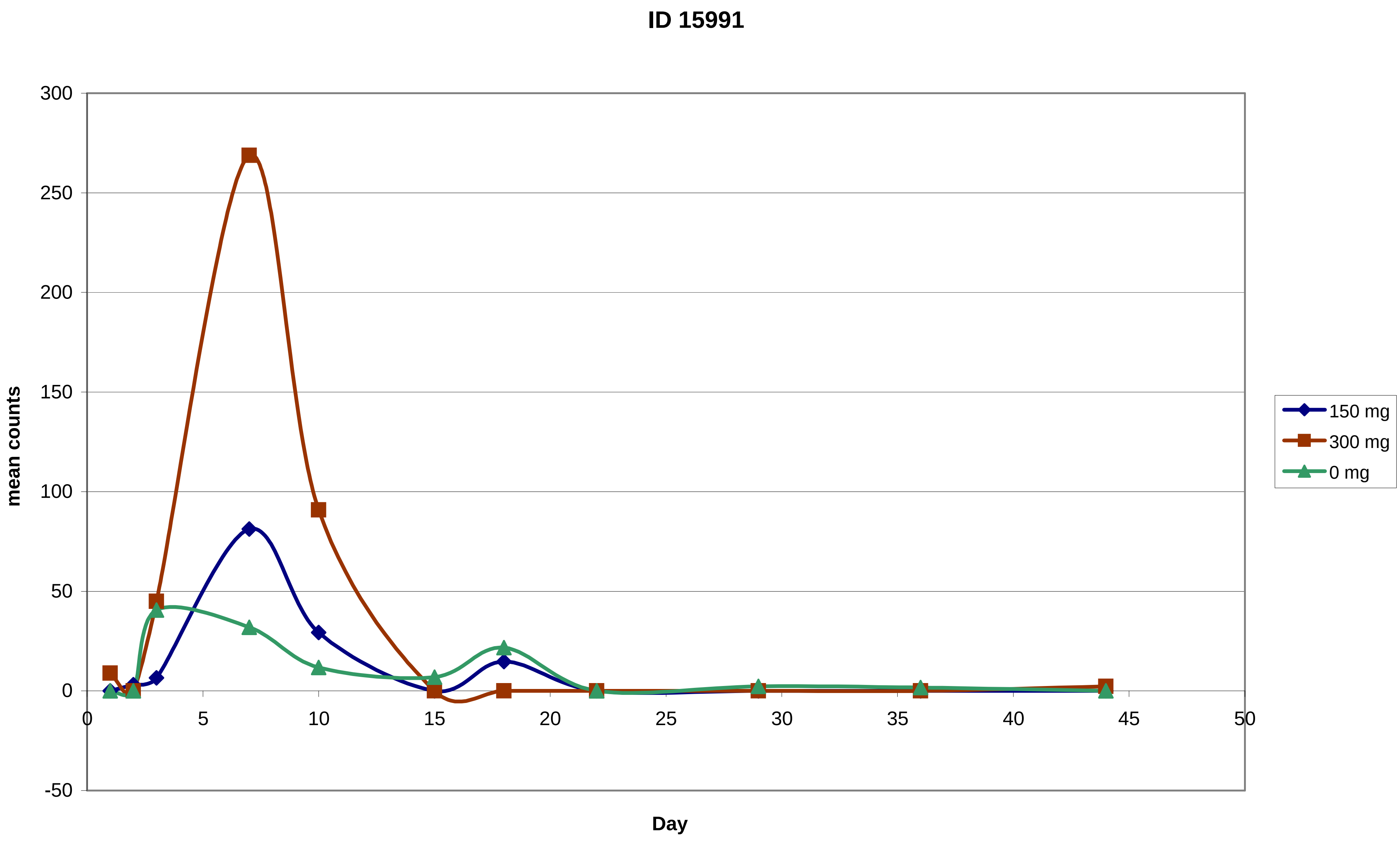
ID 15991
mean counts
300
250
200
150
100
50
0
-50
0
5
10
15
20
25
30
35
40
45
50
Day
150 mg
300 mg
0 mg

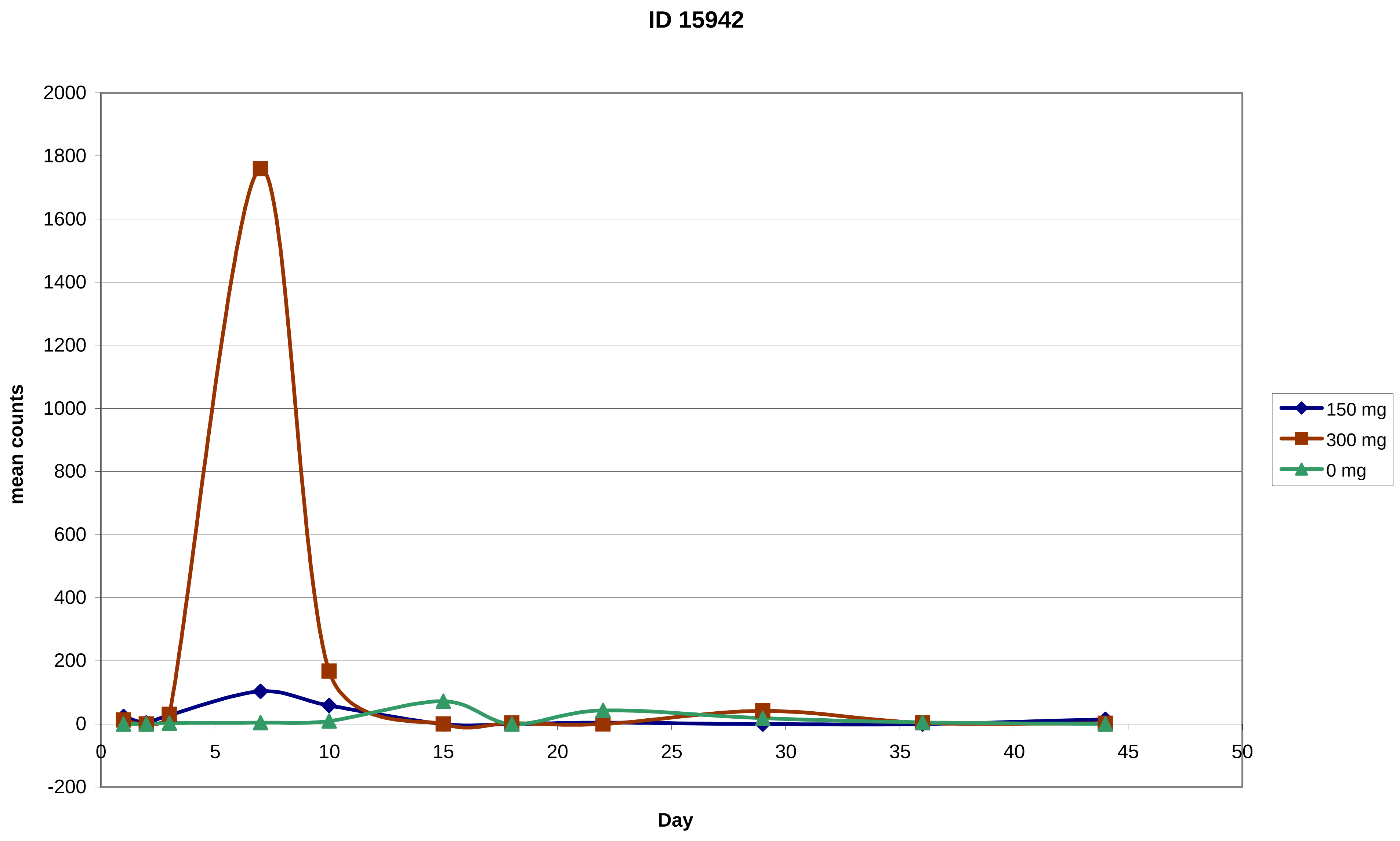
ID 15942
mean counts
2000
1800
1600
1400
1200
1000
800
600
400
200
0
-200
0
5
10
15
20
25
30
35
40
45
50
Day
150 mg
300 mg
0 mg

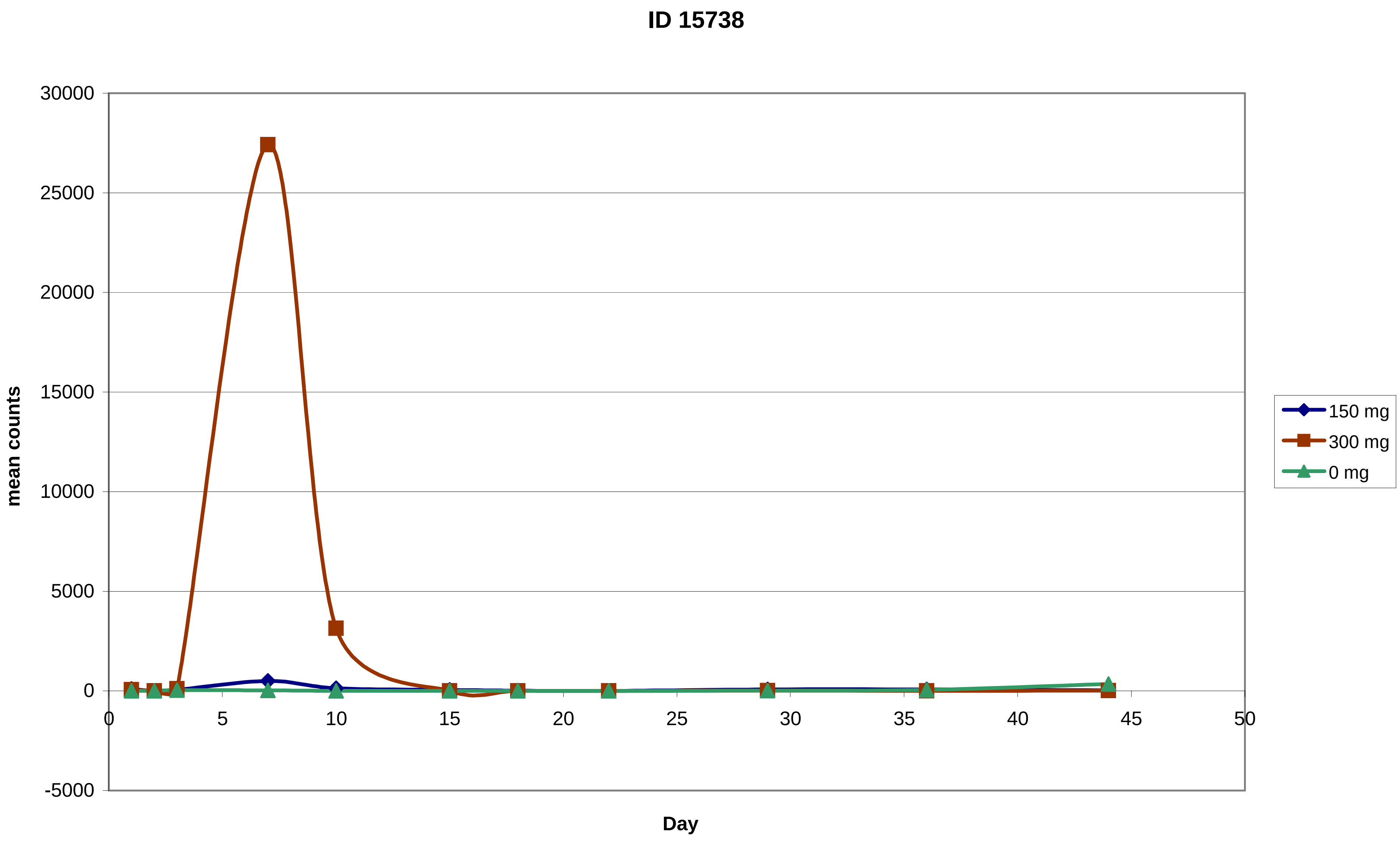
ID 15738
mean counts
30000
25000
20000
15000
10000
5000
0
-5000
0
5
10
15
20
25
30
35
40
45
50
Day
150 mg
300 mg
0 mg

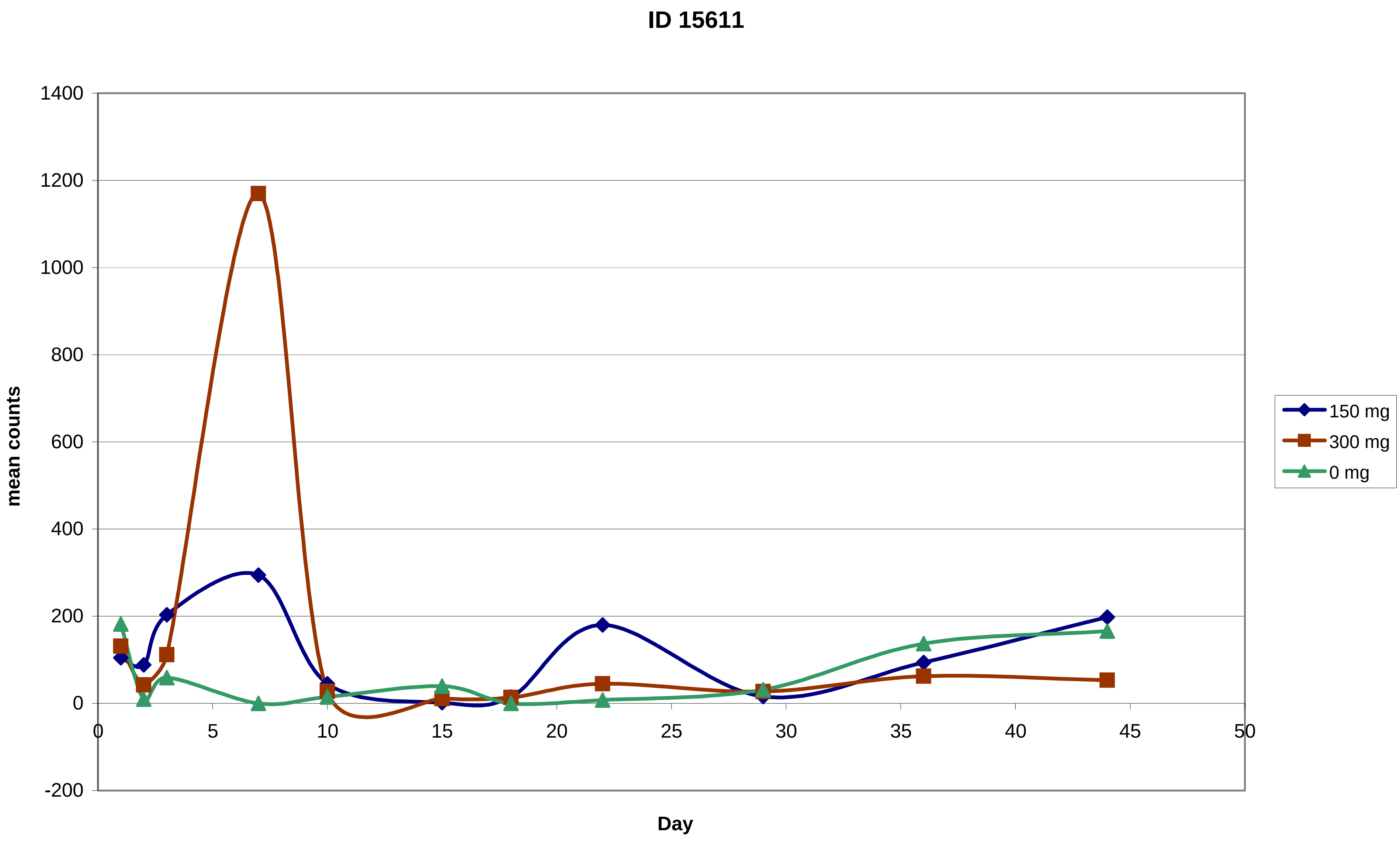
ID 15611
mean counts
1400
1200
1000
800
600
400
200
0
-200
0
5
10
15
20
25
30
35
40
45
50
Day
150 mg
300 mg
0 mg

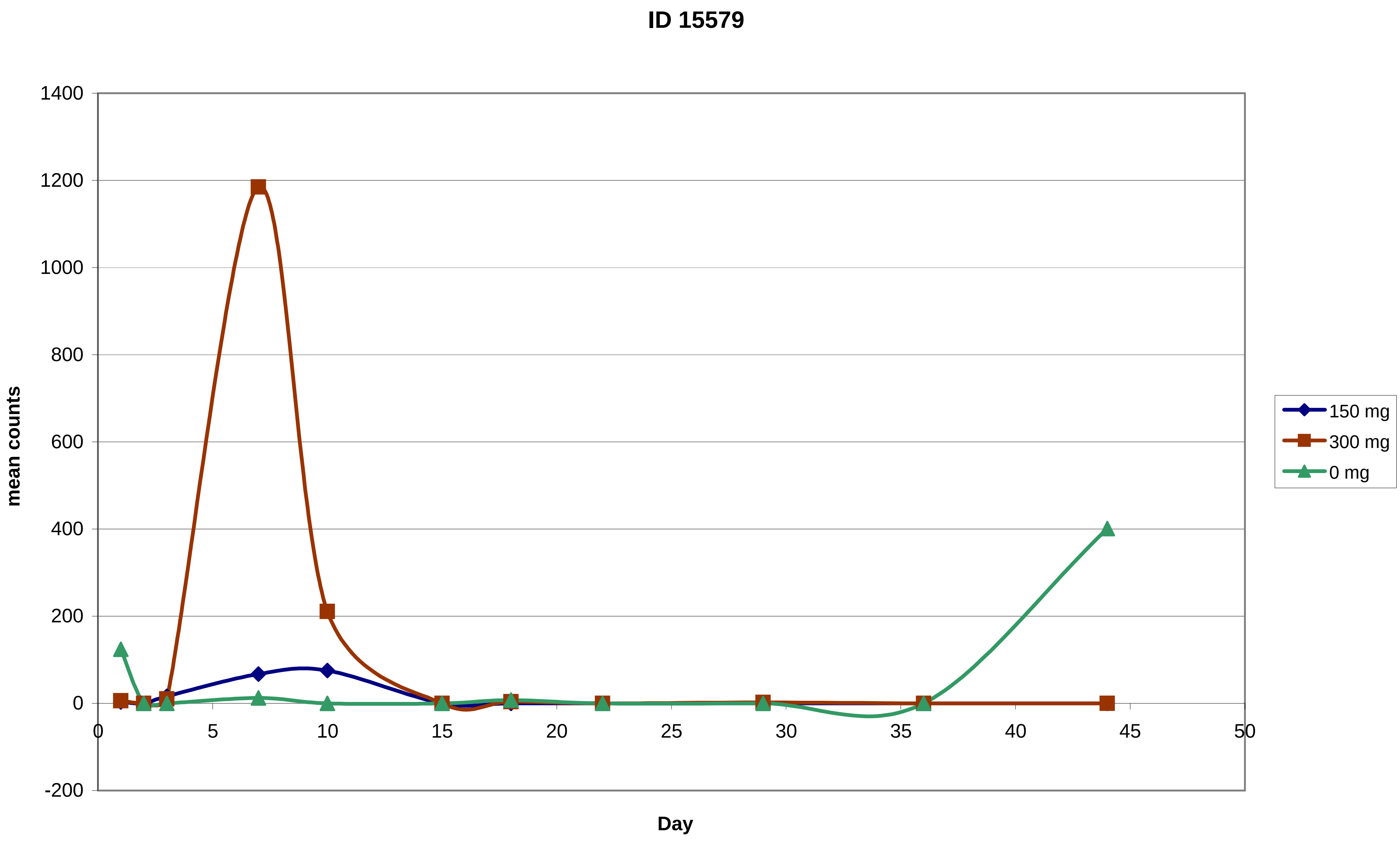
ID 15579
mean counts
1400
1200
1000
800
600
400
200
0
-200
0
5
10
15
20
25
30
35
40
45
50
Day
150 mg
300 mg
0 mg

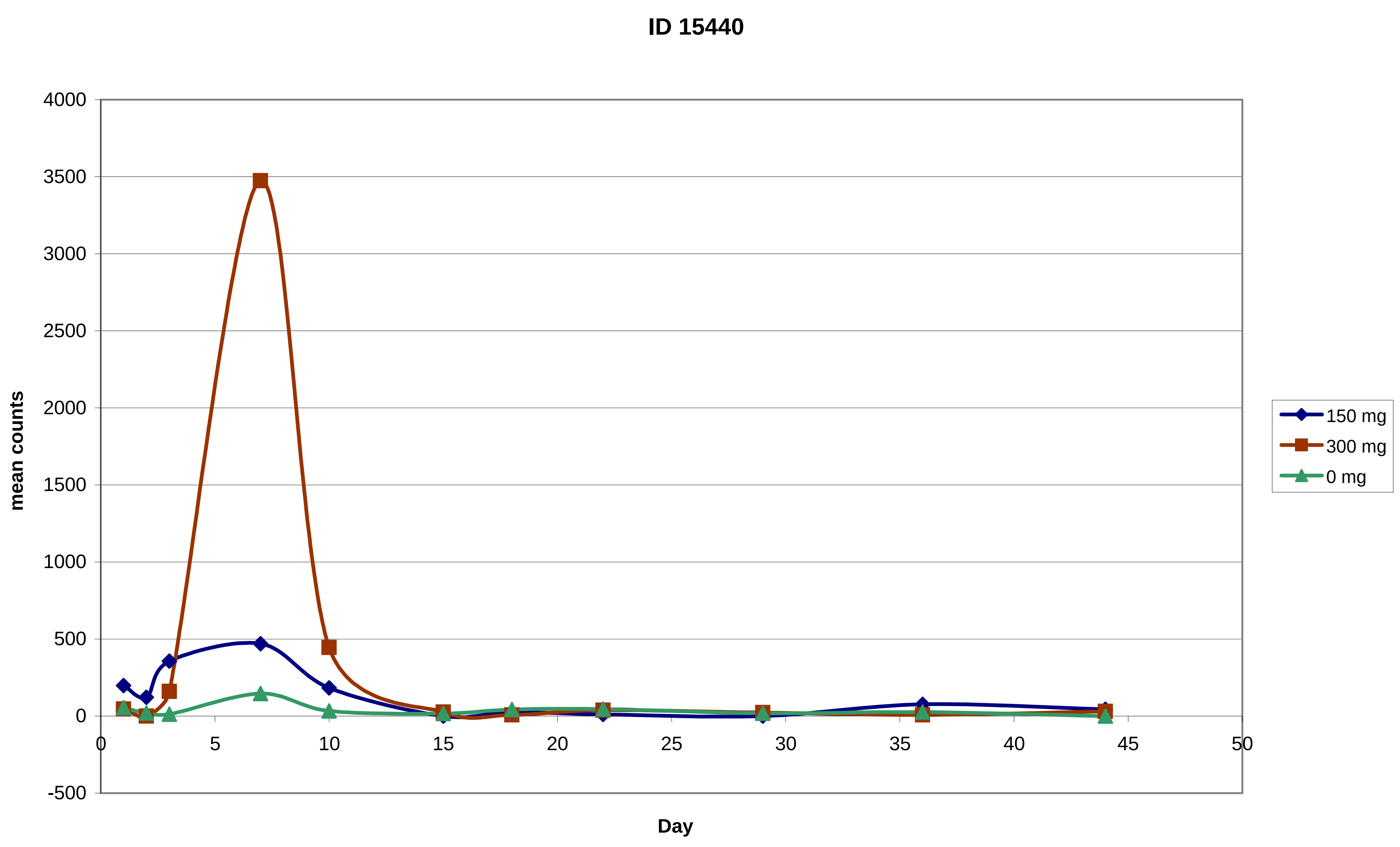

ID 15440
4000
3500
3000
2500
2000
1500
1000
500
0
-500
mean counts
0
5
10
15
20
25
30
35
40
45
50
Day
150 mg
300 mg
0 mg

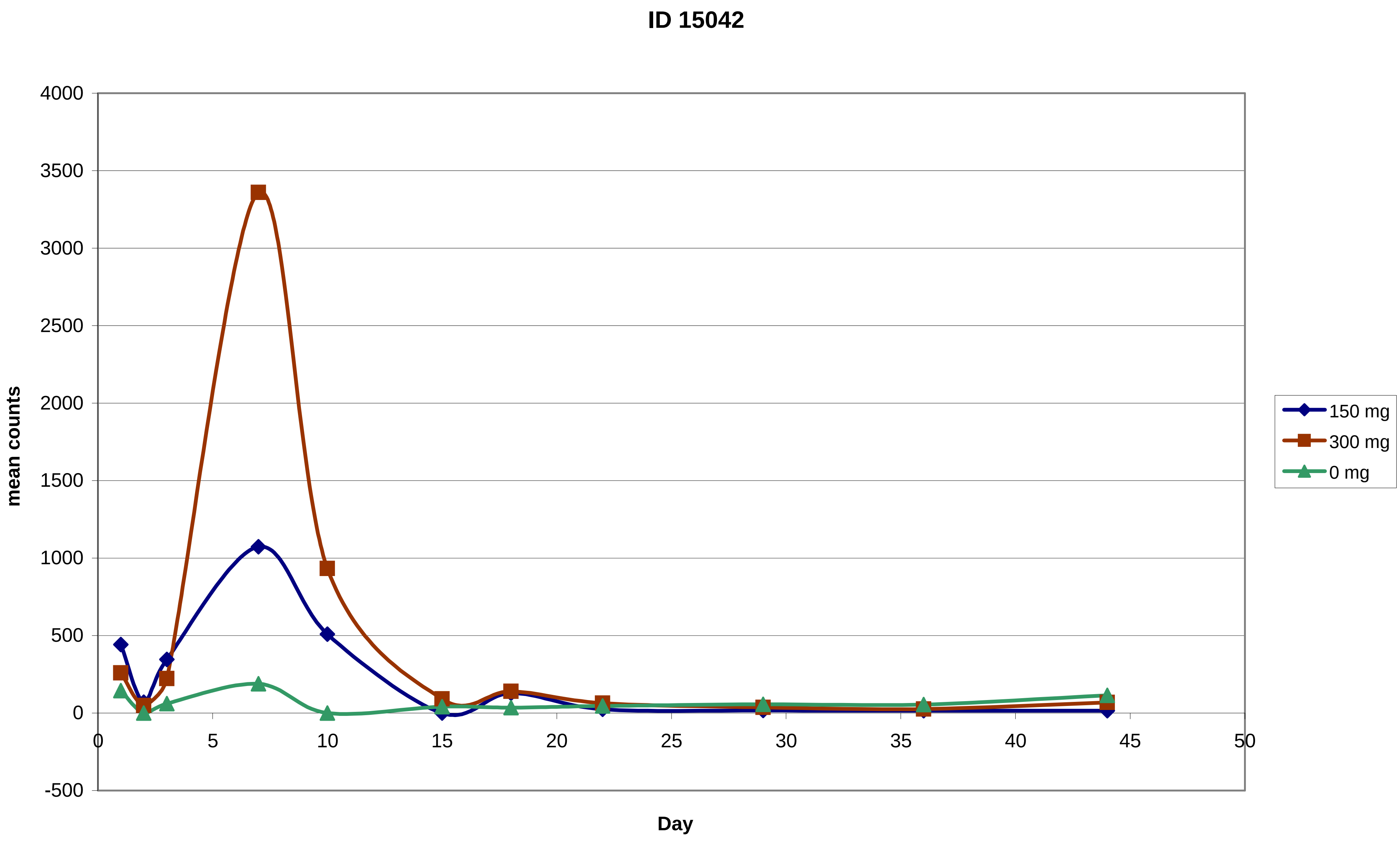
ID 15042
mean counts
4000
3500
3000
2500
2000
1500
1000
500
0
-500
0
5
10
15
20
25
30
35
40
45
50
Day
150 mg
300 mg
0 mg

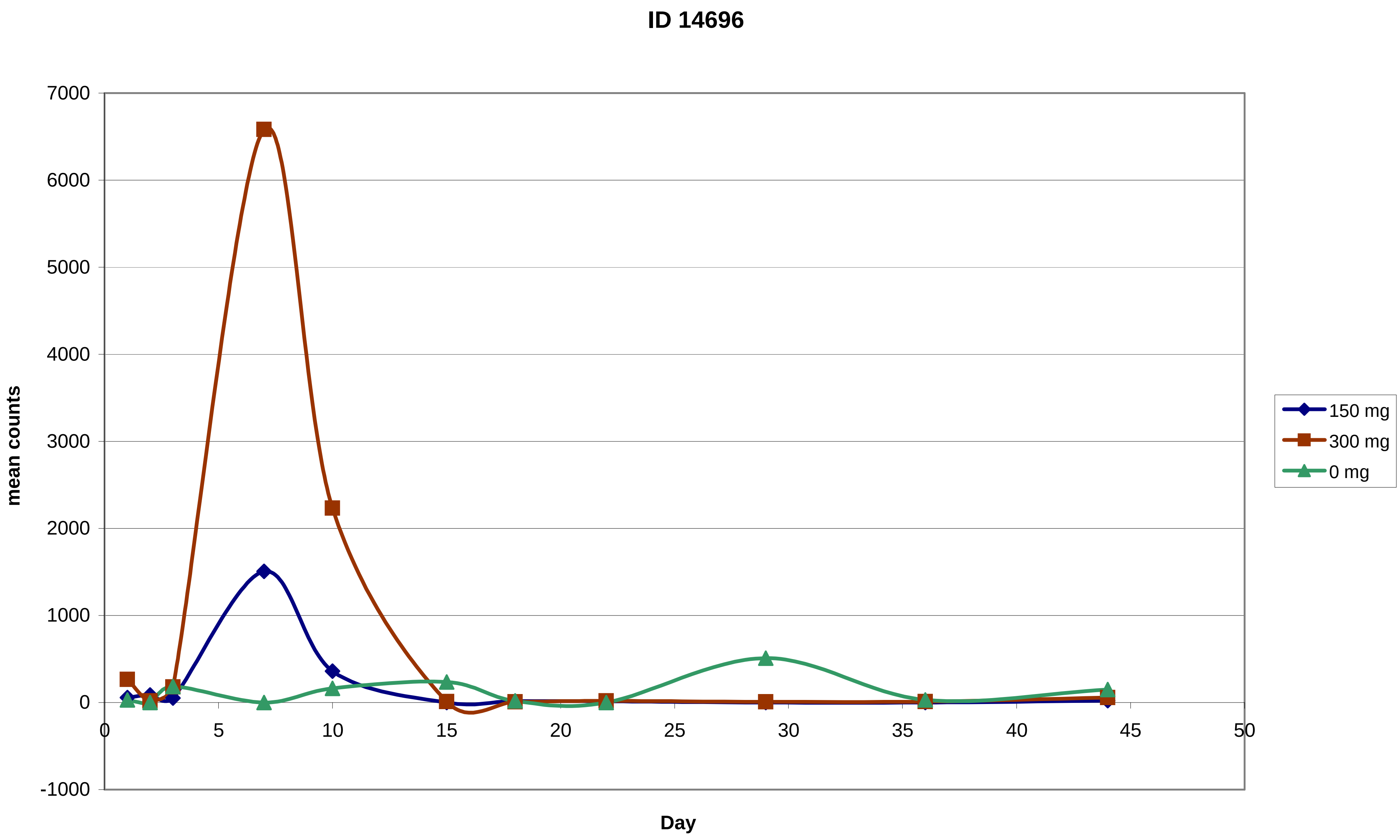

ID 14696
mean counts
7000
6000
5000
4000
3000
2000
1000
0
-1000
0
5
10
15
20
25
30
35
40
45
50
Day
150 mg
300 mg
0 mg

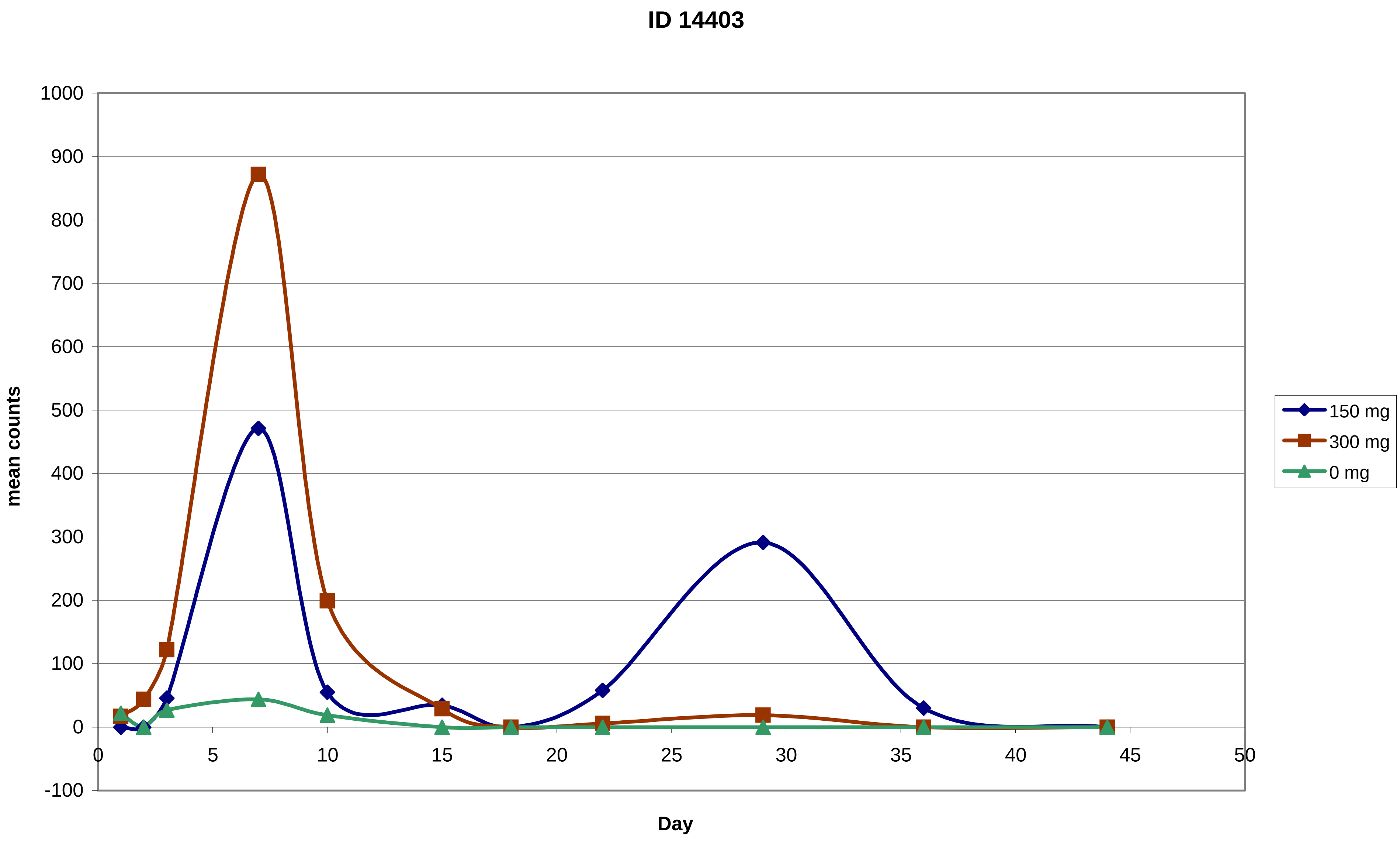
ID 14403
mean counts
1000
900
800
700
600
500
400
300
200
100
0
-100
0
5
10
15
20
25
30
35
40
45
50
Day
150 mg
300 mg
0 mg

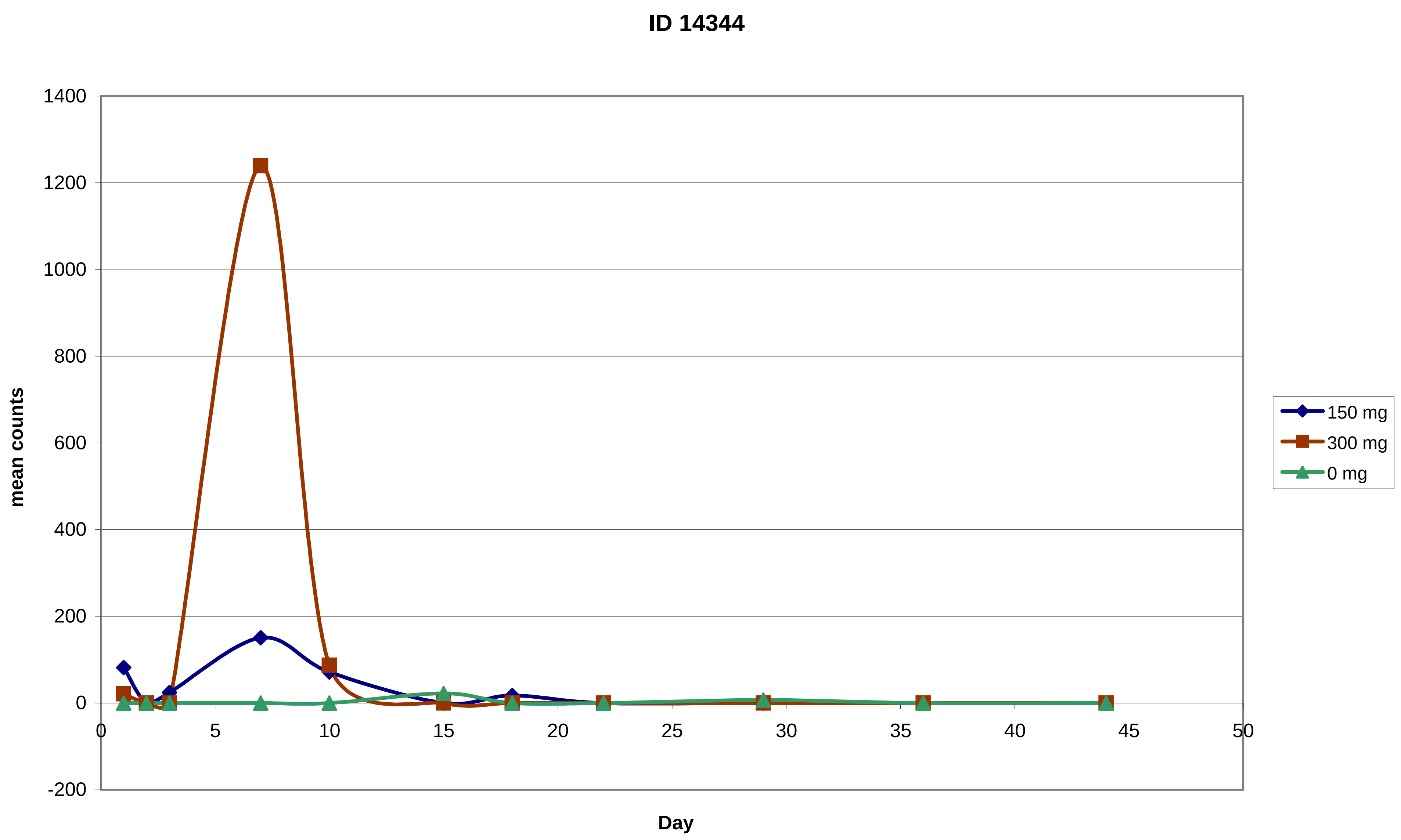

ID 14344
1400
1200
1000
800
600
400
200
0
-200
mean counts
0
5
10
15
20
25
30
35
40
45
50
Day
150 mg
300 mg
0 mg

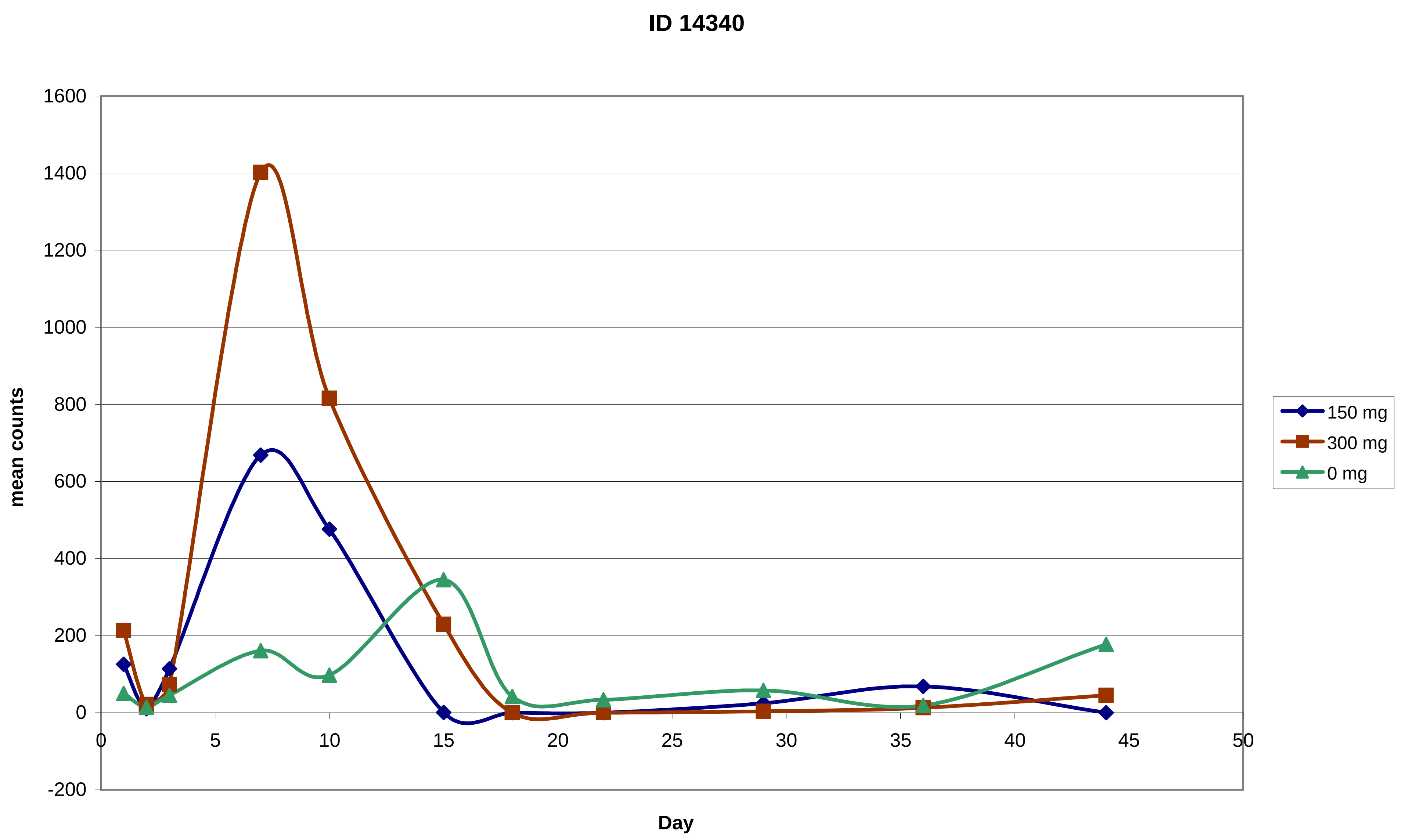

ID 14340
mean counts
Day
1600
1400
1200
1000
800
600
400
200
0
-200
0
5
10
15
20
25
30
35
40
45
50
150 mg
300 mg
0 mg

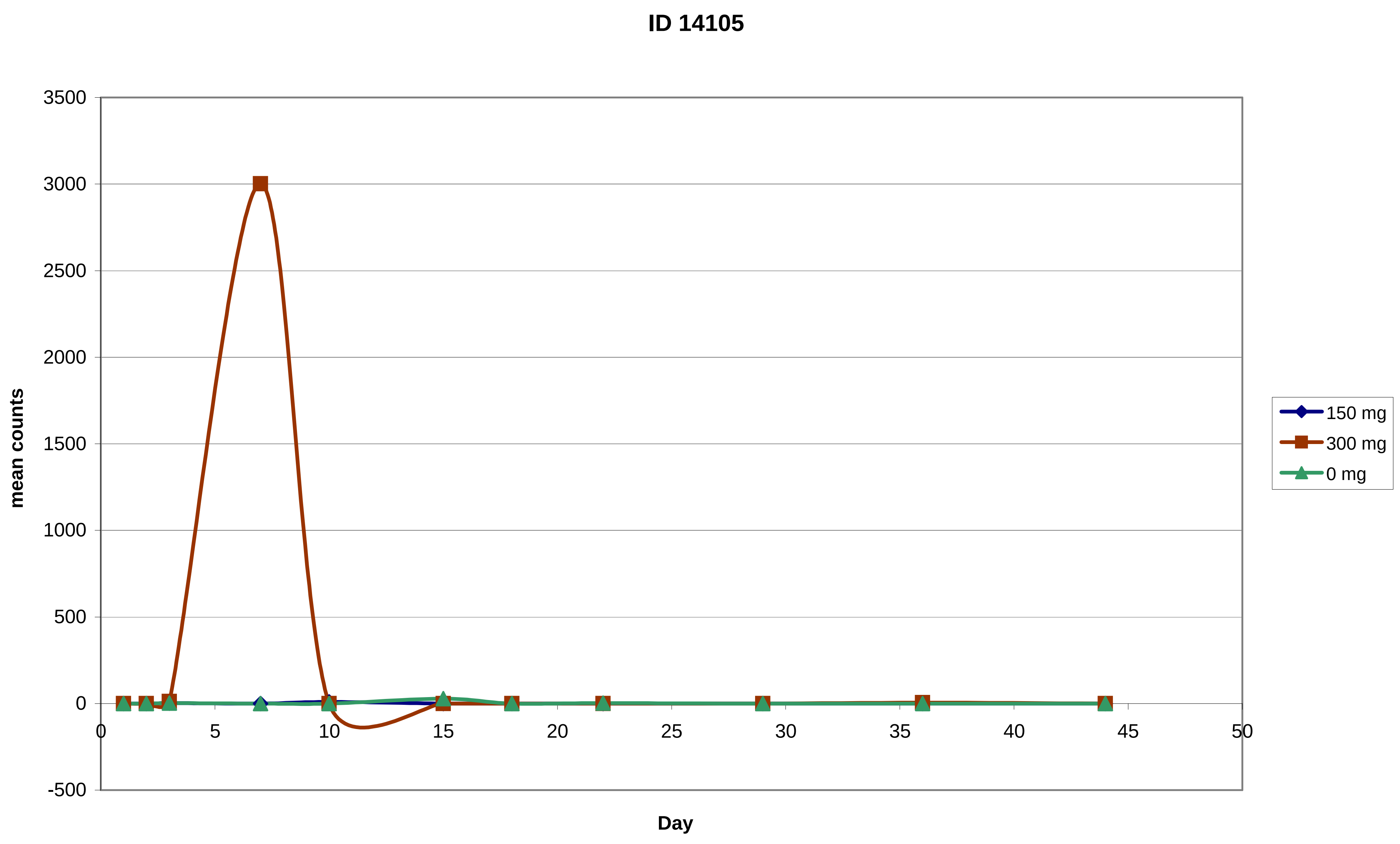
ID 14105
mean counts
Day
3500
3000
2500
2000
1500
1000
500
0
-500
0
5
10
15
20
25
30
35
40
45
50
150 mg
300 mg
0 mg

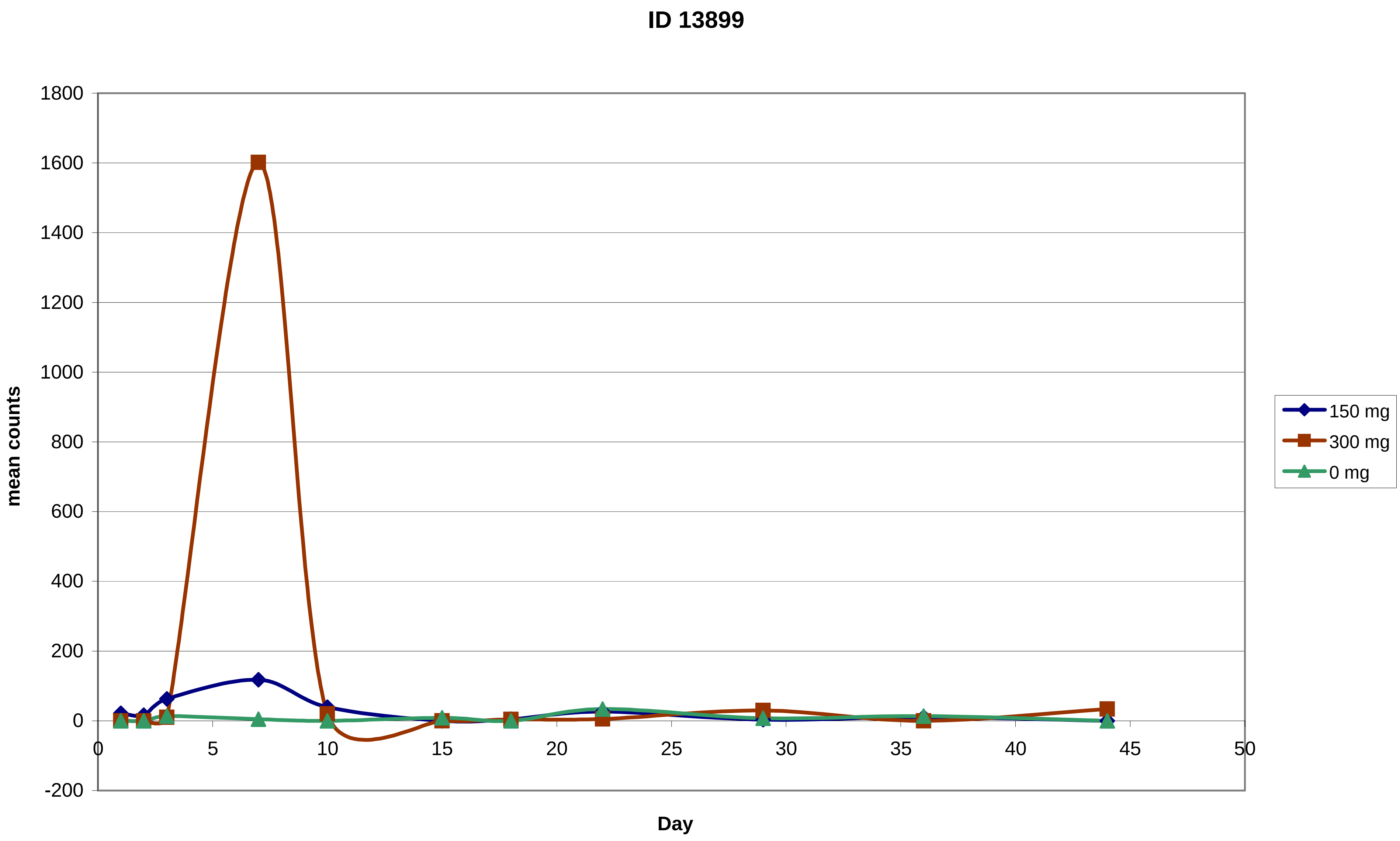
ID 13899
mean counts
1800
1600
1400
1200
1000
800
600
400
200
0
-200
0
5
10
15
20
25
30
35
40
45
50
Day
150 mg
300 mg
0 mg

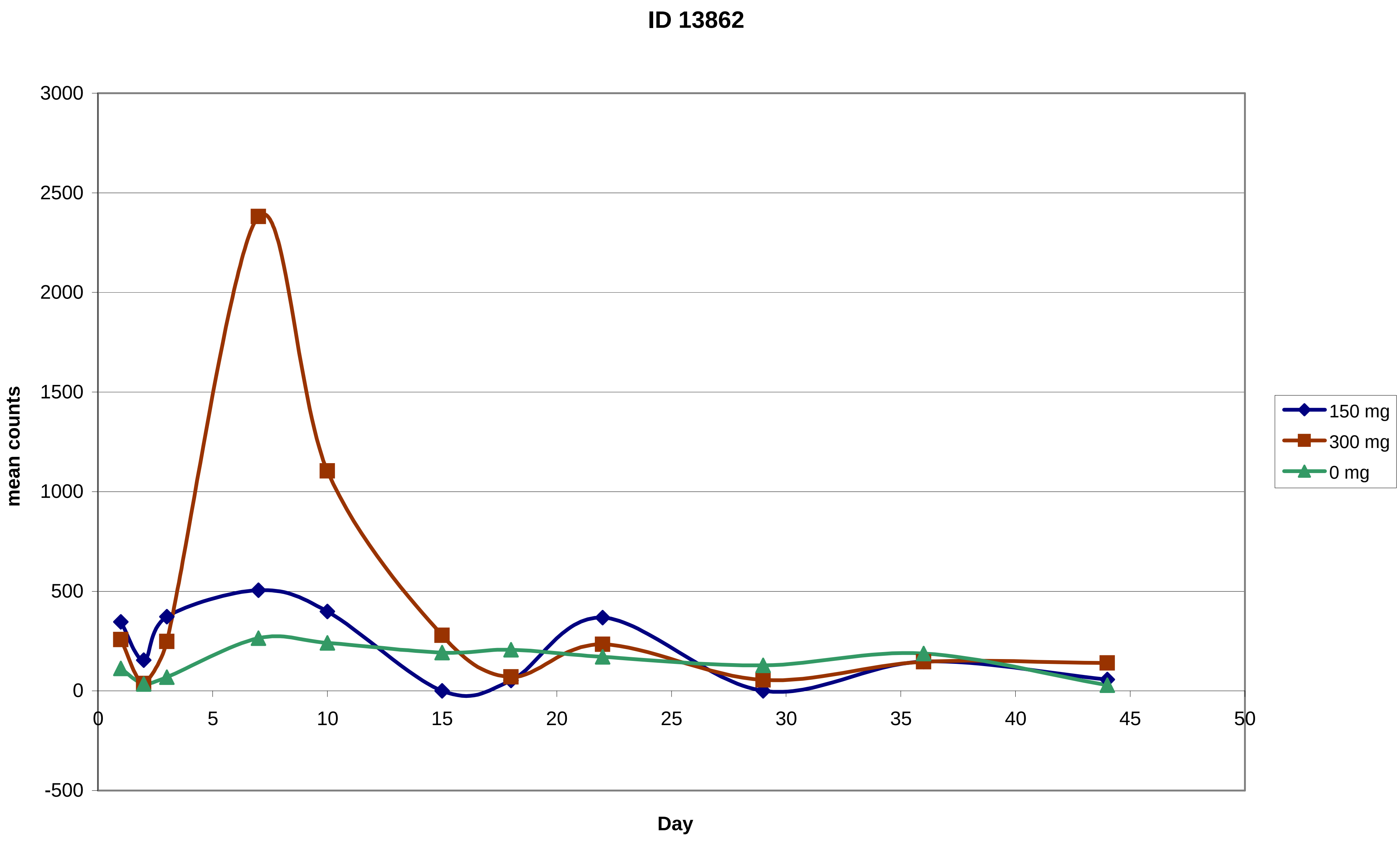
ID 13862
mean counts
3000
2500
2000
1500
1000
500
0
-500
0
5
10
15
20
25
30
35
40
45
50
Day
150 mg
300 mg
0 mg

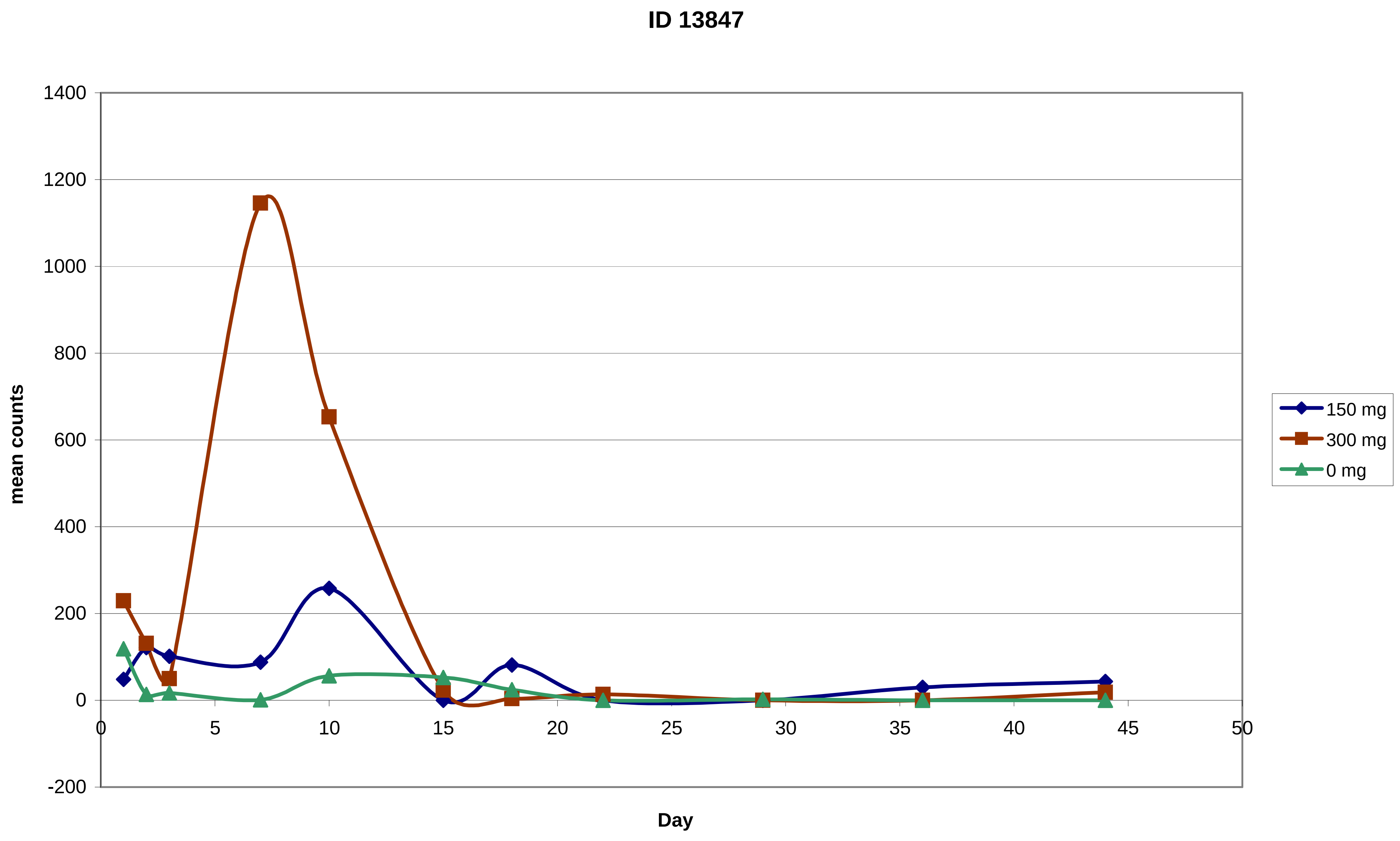
ID 13847
mean counts
1400
1200
1000
800
600
400
200
0
-200
0
5
10
15
20
25
30
35
40
45
50
Day
150 mg
300 mg
0 mg

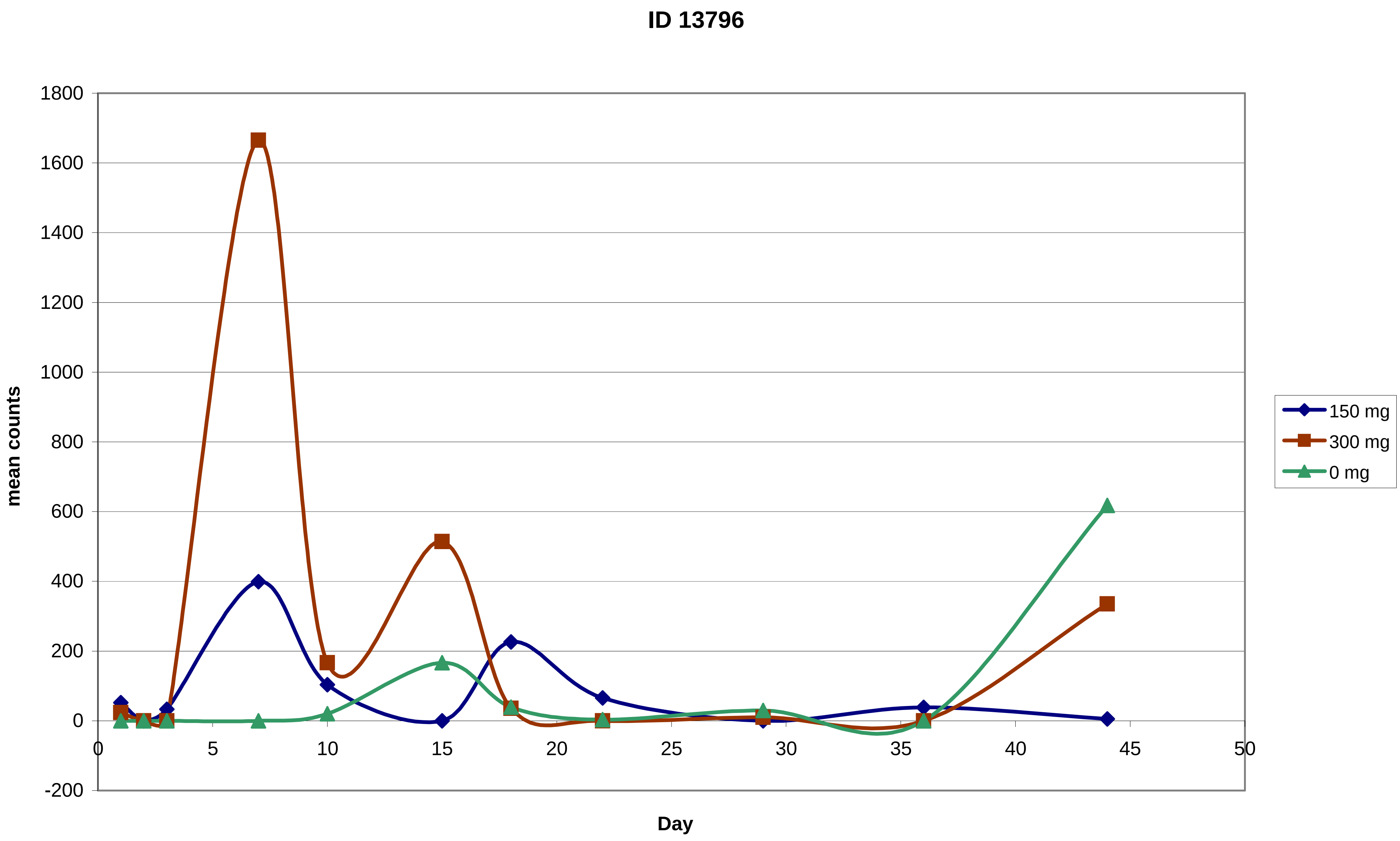

ID 13796
mean counts
1800
1600
1400
1200
1000
800
600
400
200
0
-200
0
5
10
15
20
25
30
35
40
45
50
Day
150 mg
300 mg
0 mg

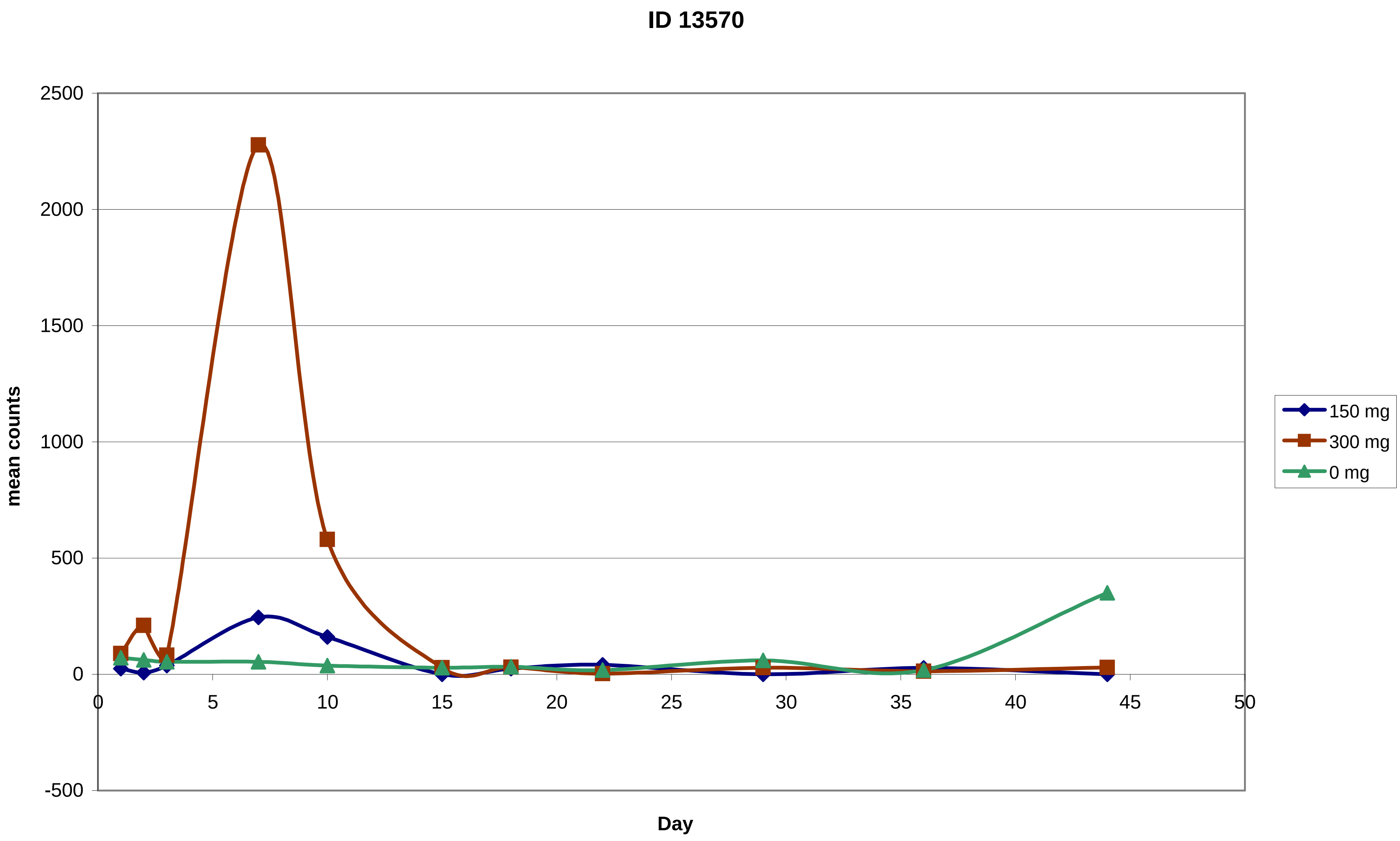
ID 13570
mean counts
2500
2000
1500
1000
500
0
-500
0
5
10
15
20
25
30
35
40
45
50
Day
150 mg
300 mg
0 mg

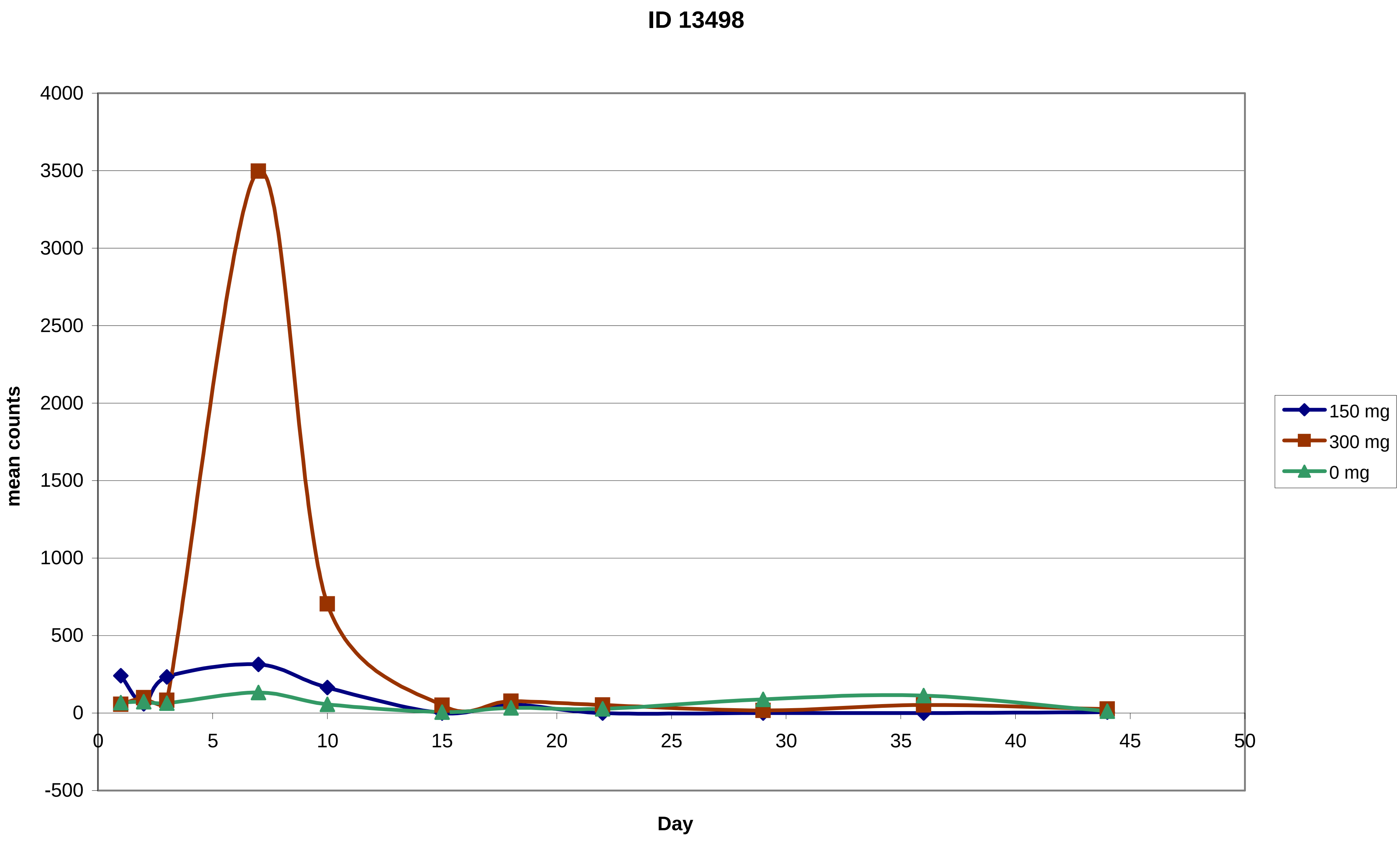
ID 13498
mean counts
4000
3500
3000
2500
2000
1500
1000
500
0
-500
0
5
10
15
20
25
30
35
40
45
50
Day
150 mg
300 mg
0 mg

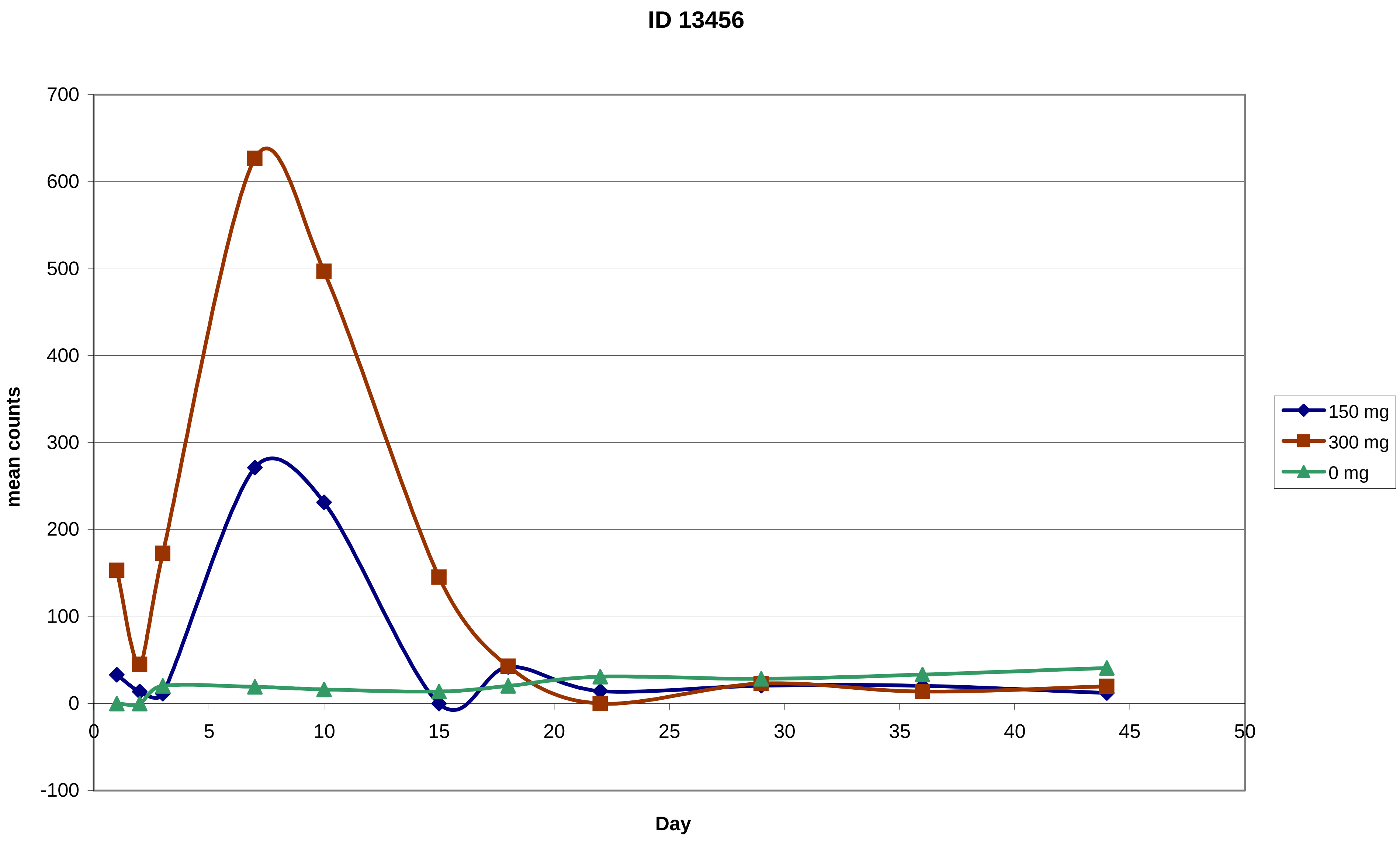
ID 13456
mean counts
700
600
500
400
300
200
100
0
-100
0
5
10
15
20
25
30
35
40
45
50
Day
150 mg
300 mg
0 mg

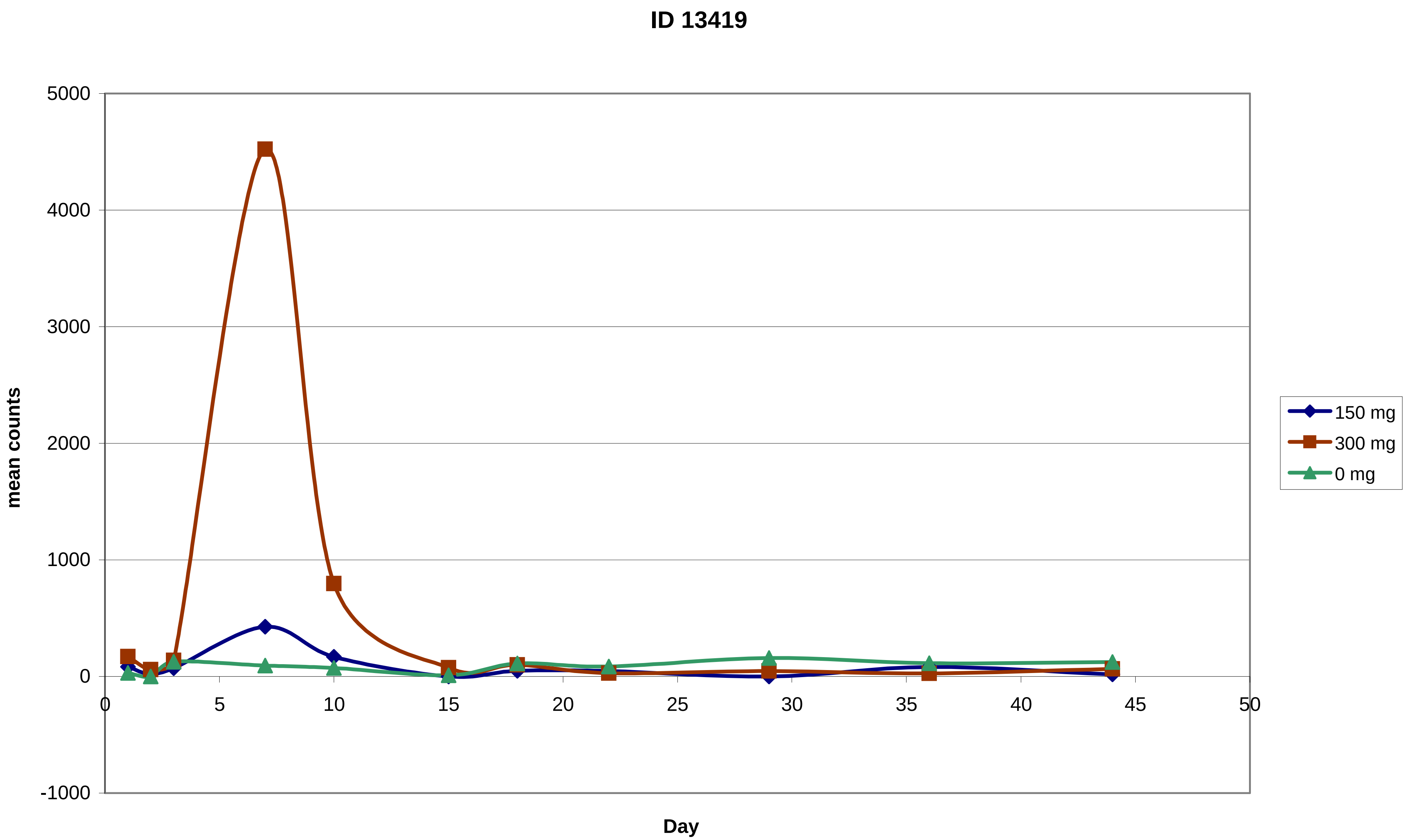

ID 13419
mean counts
5000
4000
3000
2000
1000
0
-1000
0
5
10
15
20
25
30
35
40
45
50
Day
150 mg
300 mg
0 mg

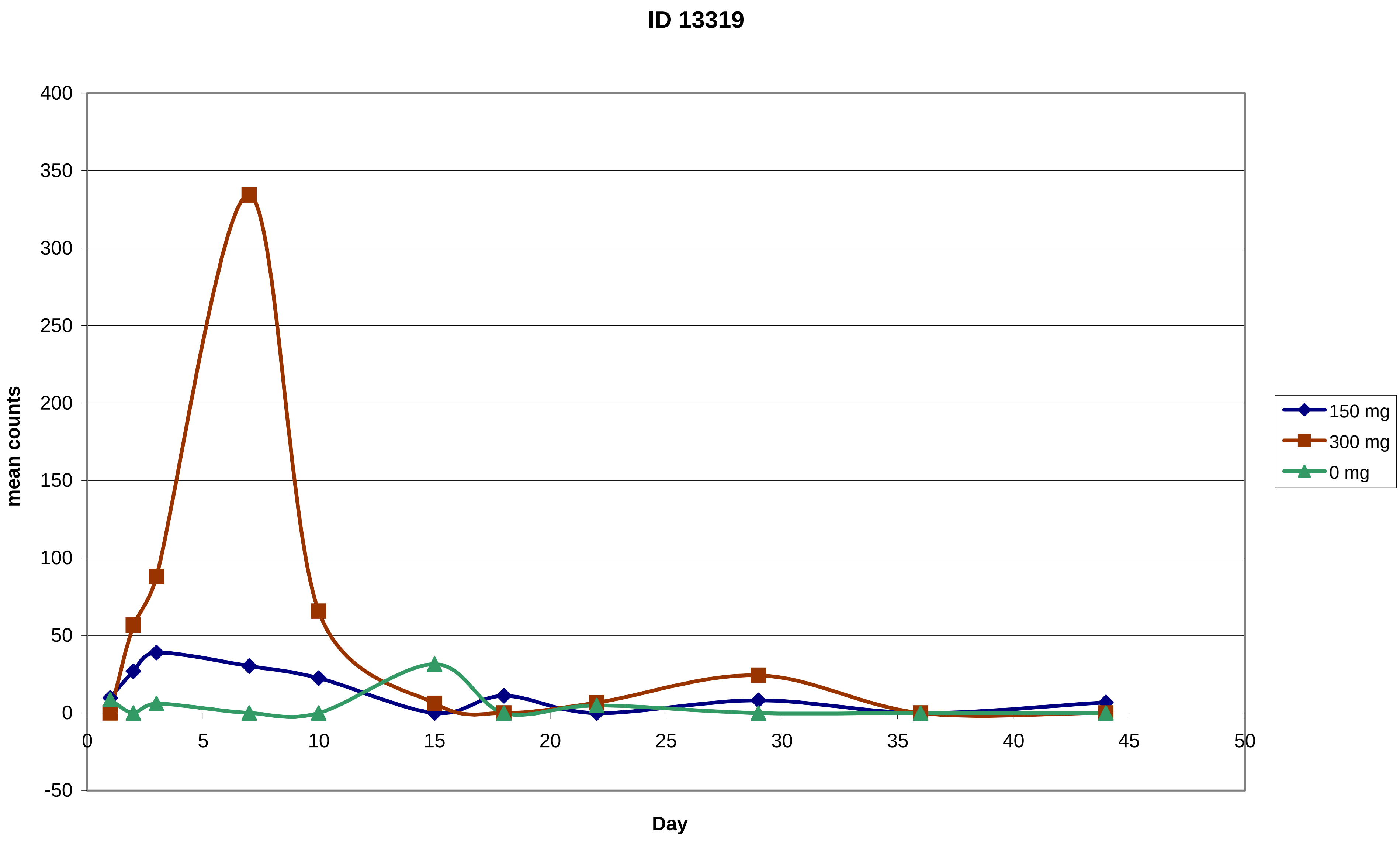
ID 13319
mean counts
400
350
300
250
200
150
100
50
0
-50
0
5
10
15
20
25
30
35
40
45
50
Day
150 mg
300 mg
0 mg

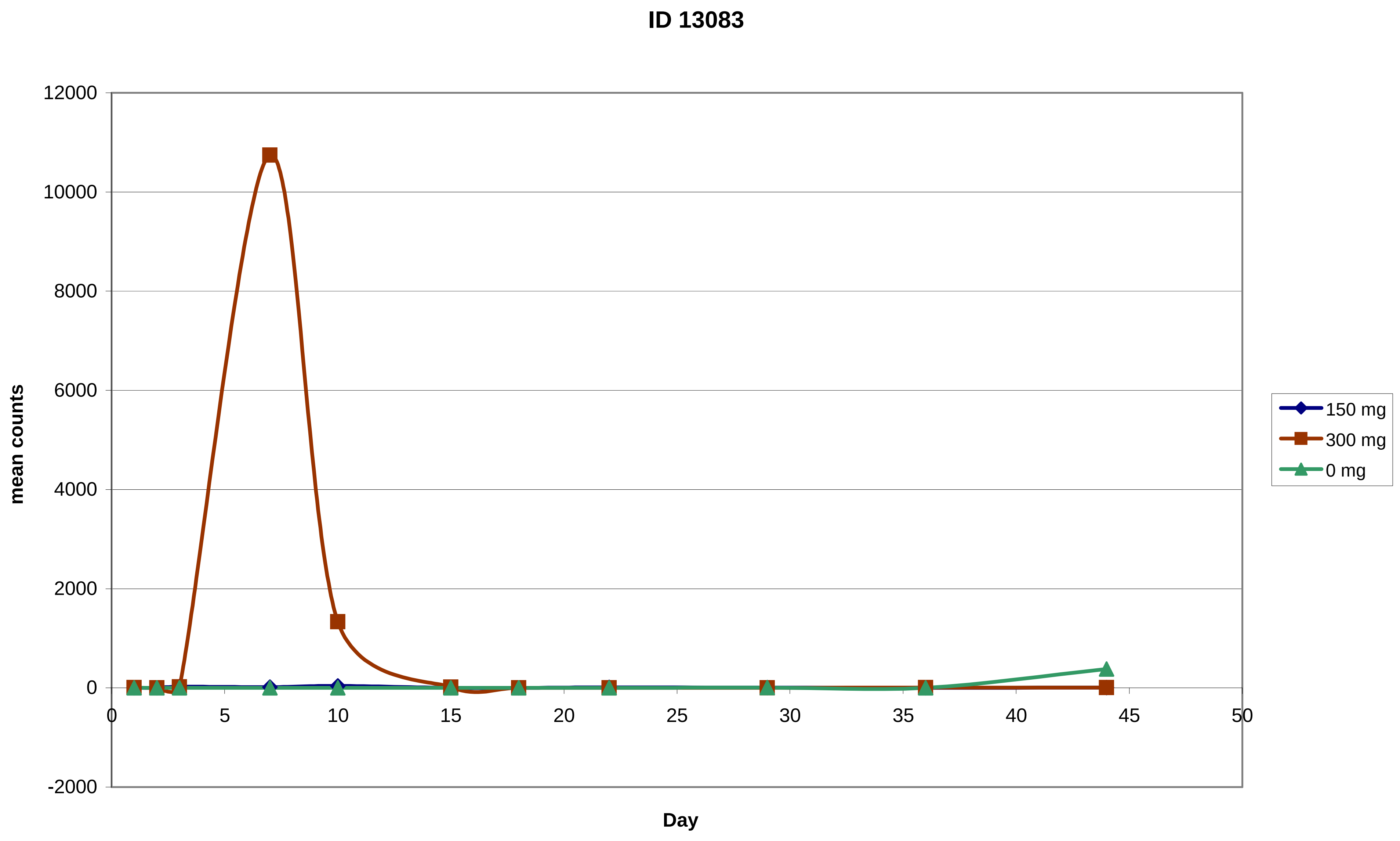
ID 13083
mean counts
12000
10000
8000
6000
4000
2000
0
-2000
0
5
10
15
20
25
30
35
40
45
50
Day
150 mg
300 mg
0 mg

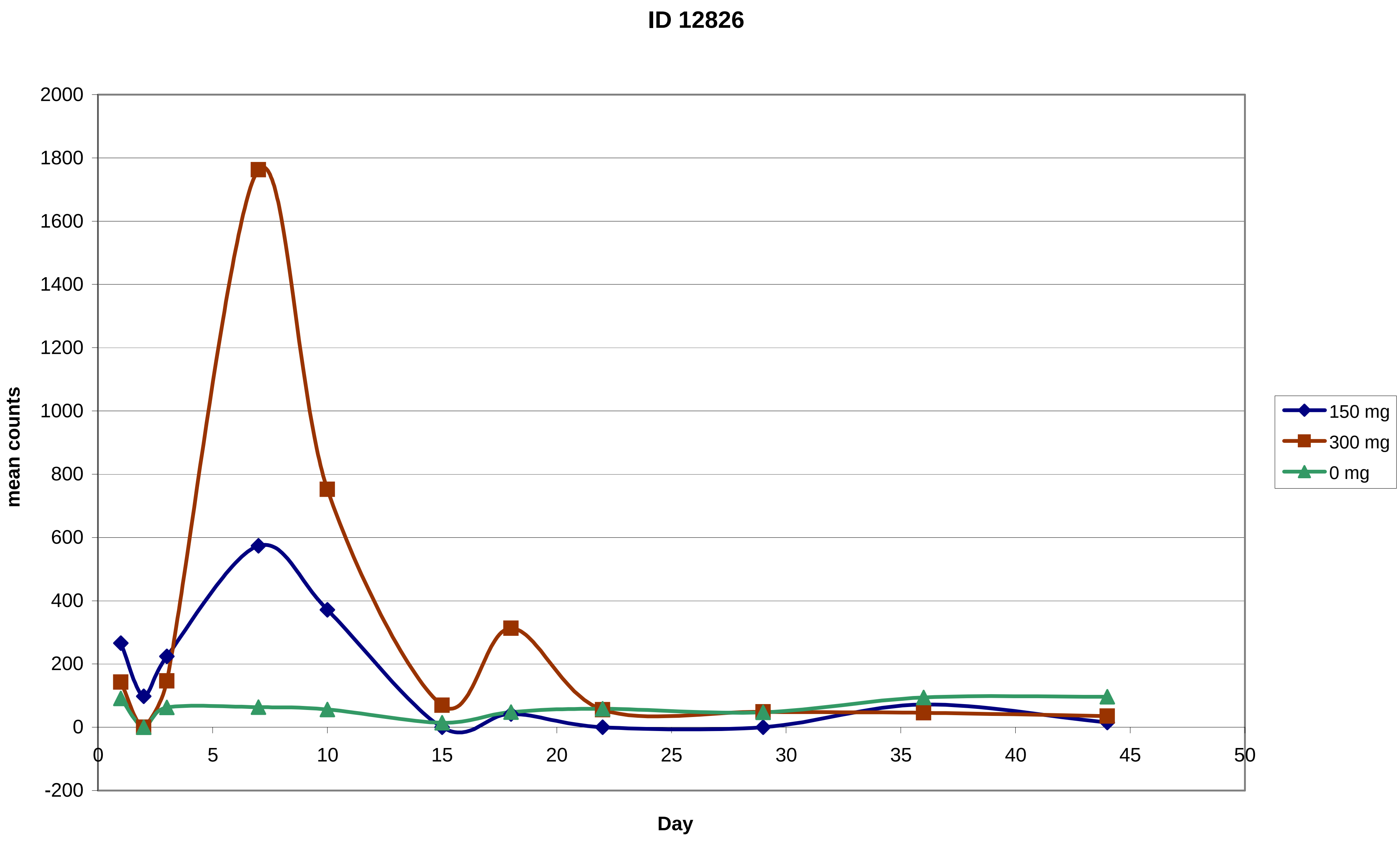
ID 12826
mean counts
2000
1800
1600
1400
1200
1000
800
600
400
200
0
-200
0
5
10
15
20
25
30
35
40
45
50
Day
150 mg
300 mg
0 mg

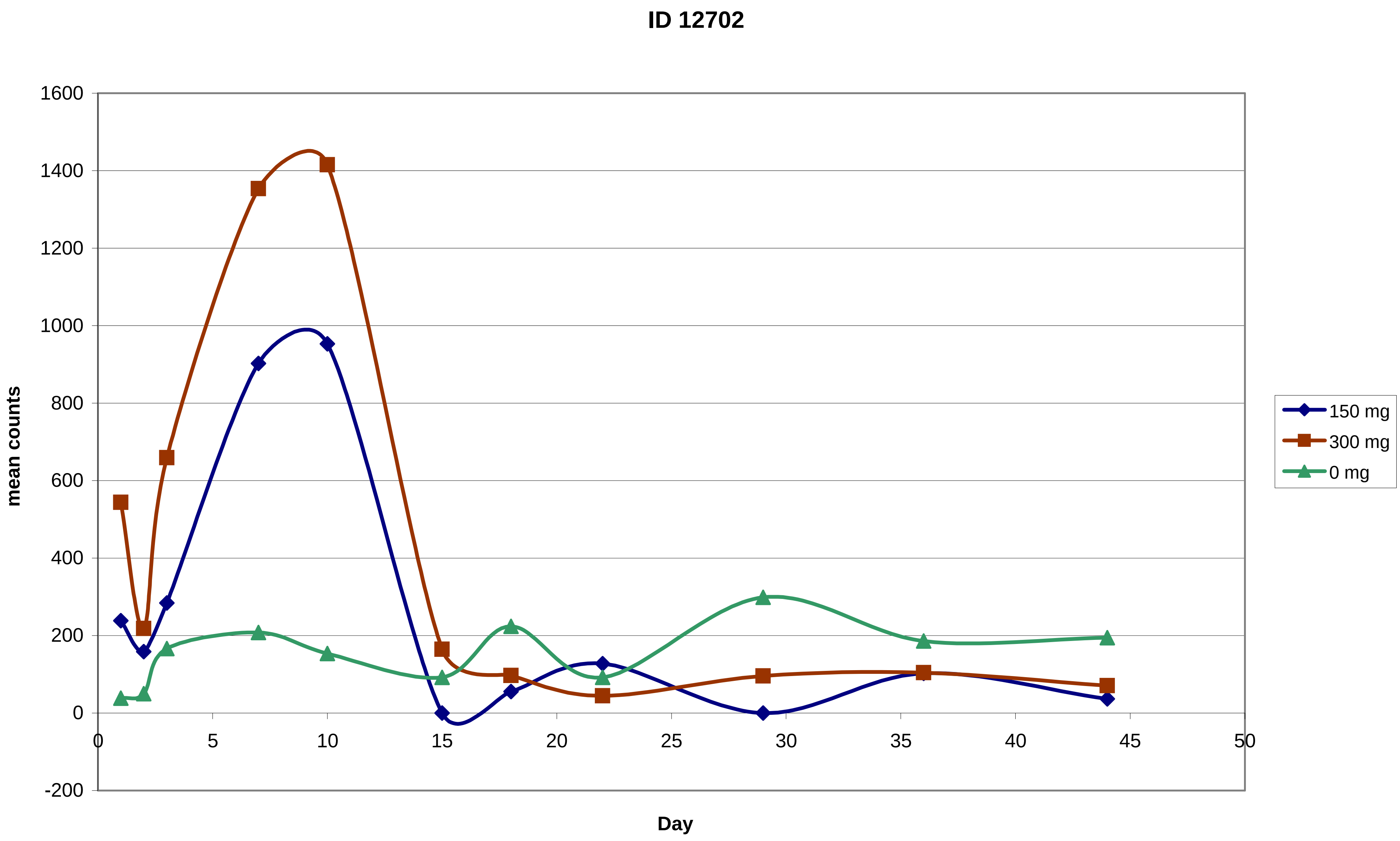
ID 12702
mean counts
1600
1400
1200
1000
800
600
400
200
0
-200
0
5
10
15
20
25
30
35
40
45
50
Day
150 mg
300 mg
0 mg

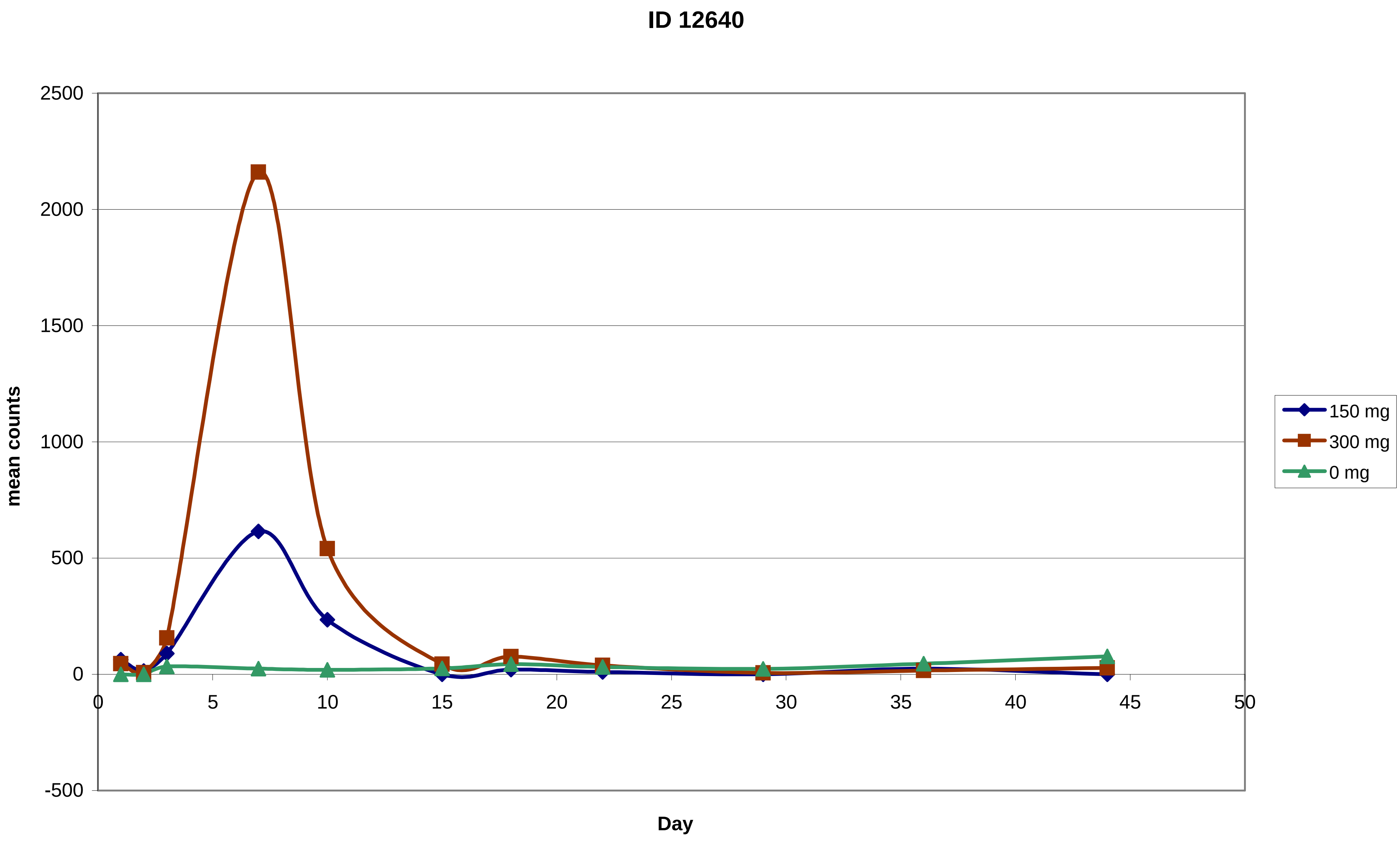
ID 12640
mean counts
2500
2000
1500
1000
500
0
-500
0
5
10
15
20
25
30
35
40
45
50
Day
150 mg
300 mg
0 mg

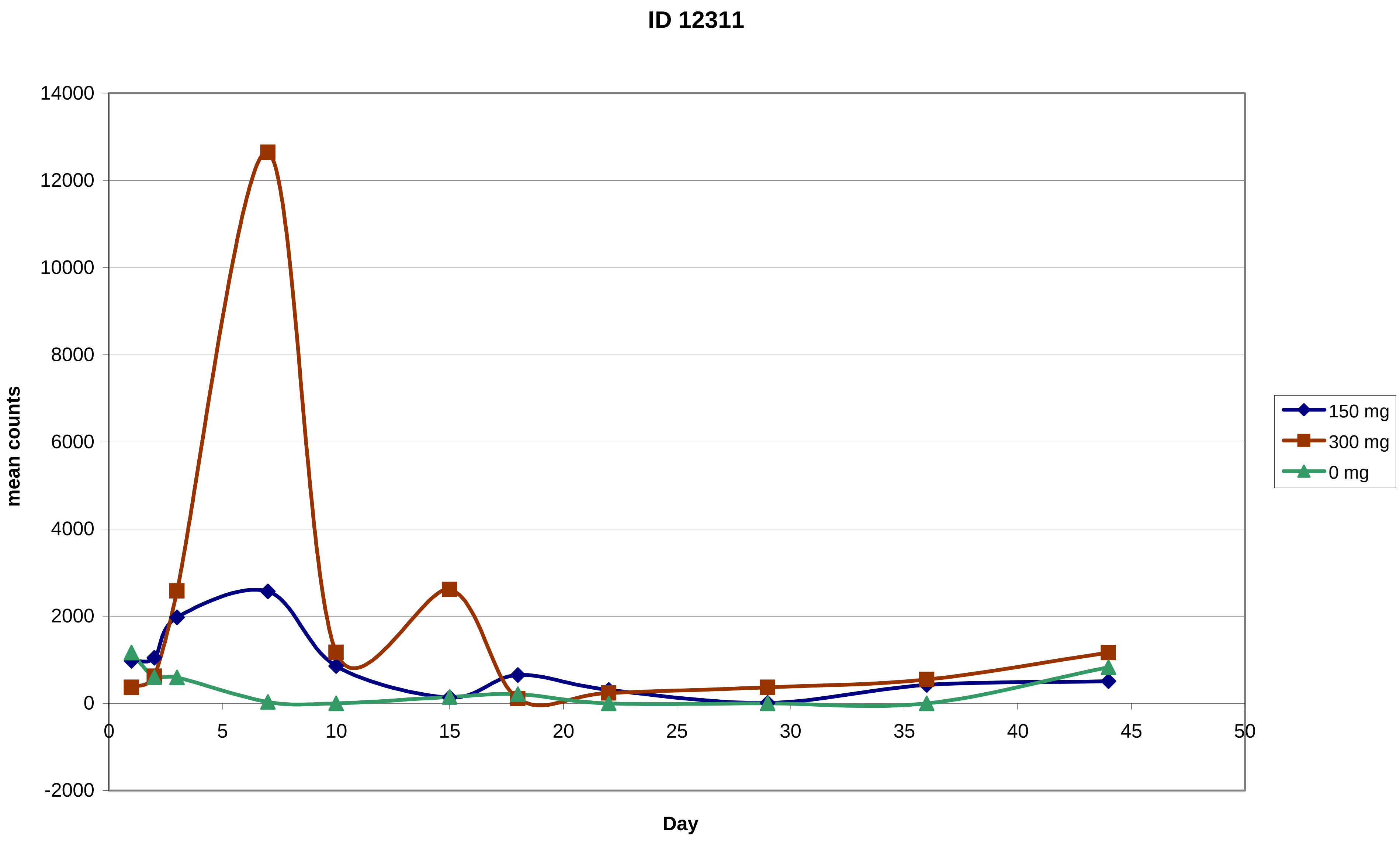
ID 12311
mean counts
14000
12000
10000
8000
6000
4000
2000
0
-2000
0
5
10
15
20
25
30
35
40
45
50
Day
150 mg
300 mg
0 mg

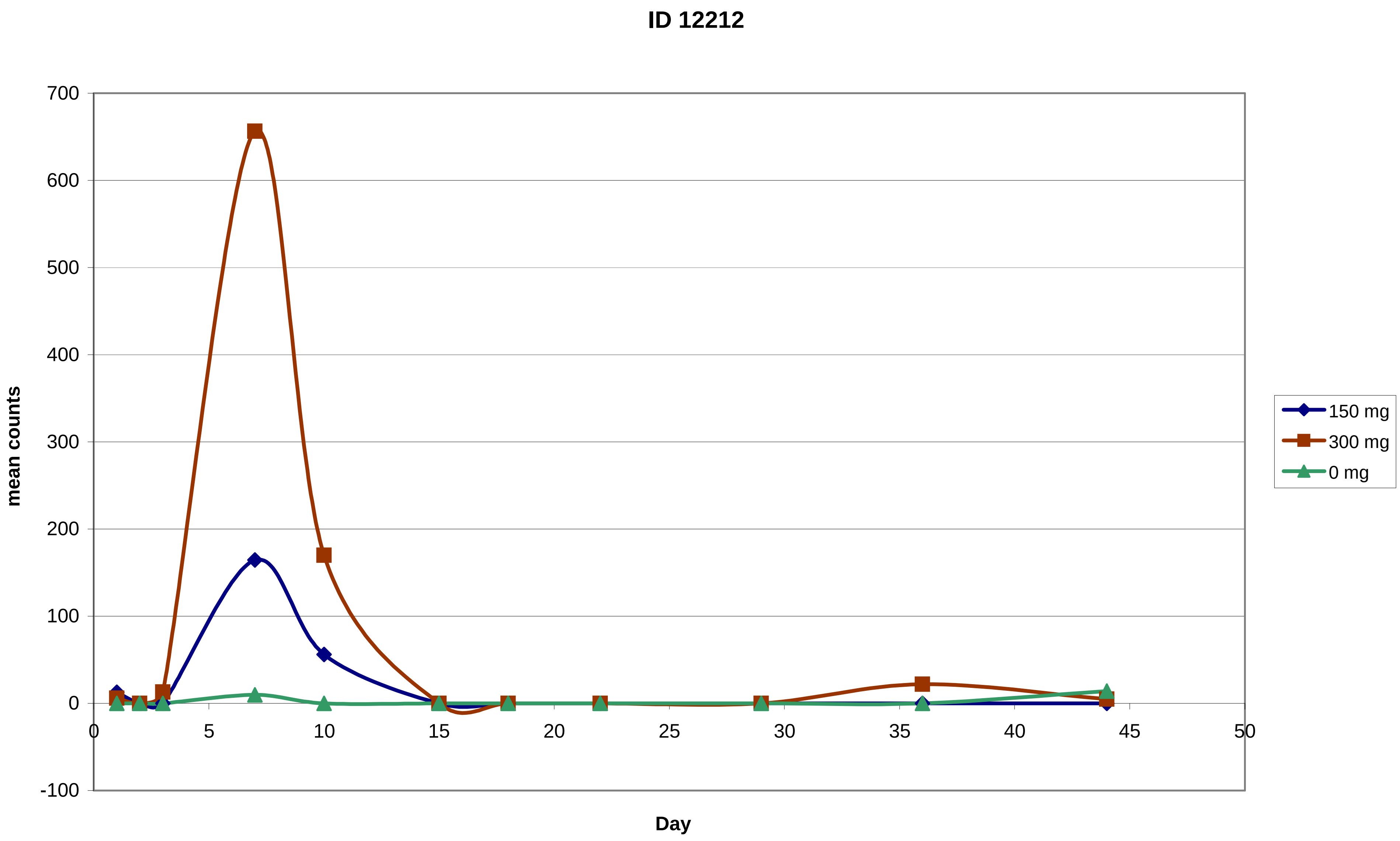
ID 12212
mean counts
700
600
500
400
300
200
100
0
-100
0
5
10
15
20
25
30
35
40
45
50
Day
150 mg
300 mg
0 mg

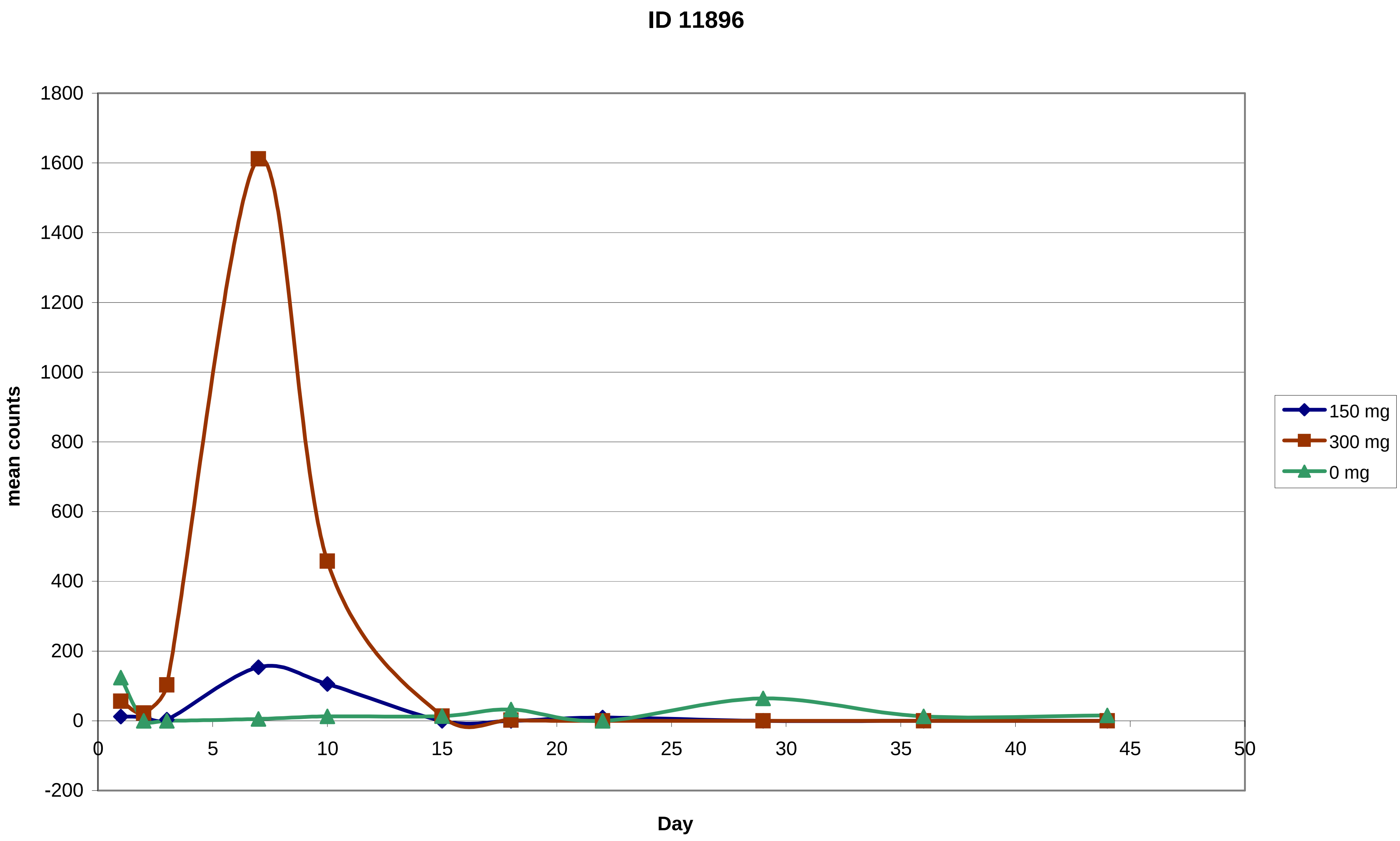
ID 11896
mean counts
1800
1600
1400
1200
1000
800
600
400
200
0
-200
0
5
10
15
20
25
30
35
40
45
50
Day
150 mg
300 mg
0 mg

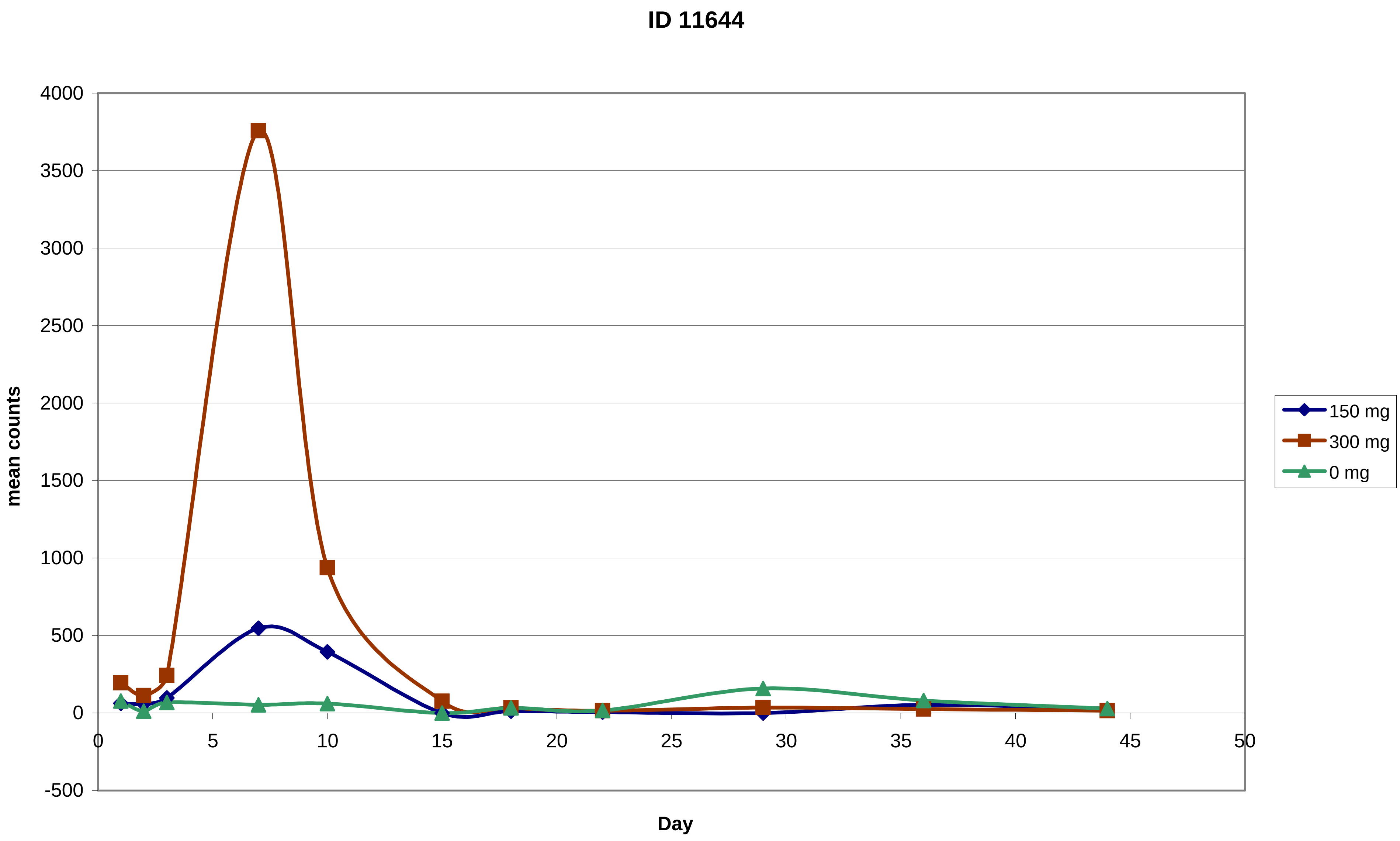
ID 11644
mean counts
4000
3500
3000
2500
2000
1500
1000
500
0
-500
0
5
10
15
20
25
30
35
40
45
50
Day
150 mg
300 mg
0 mg

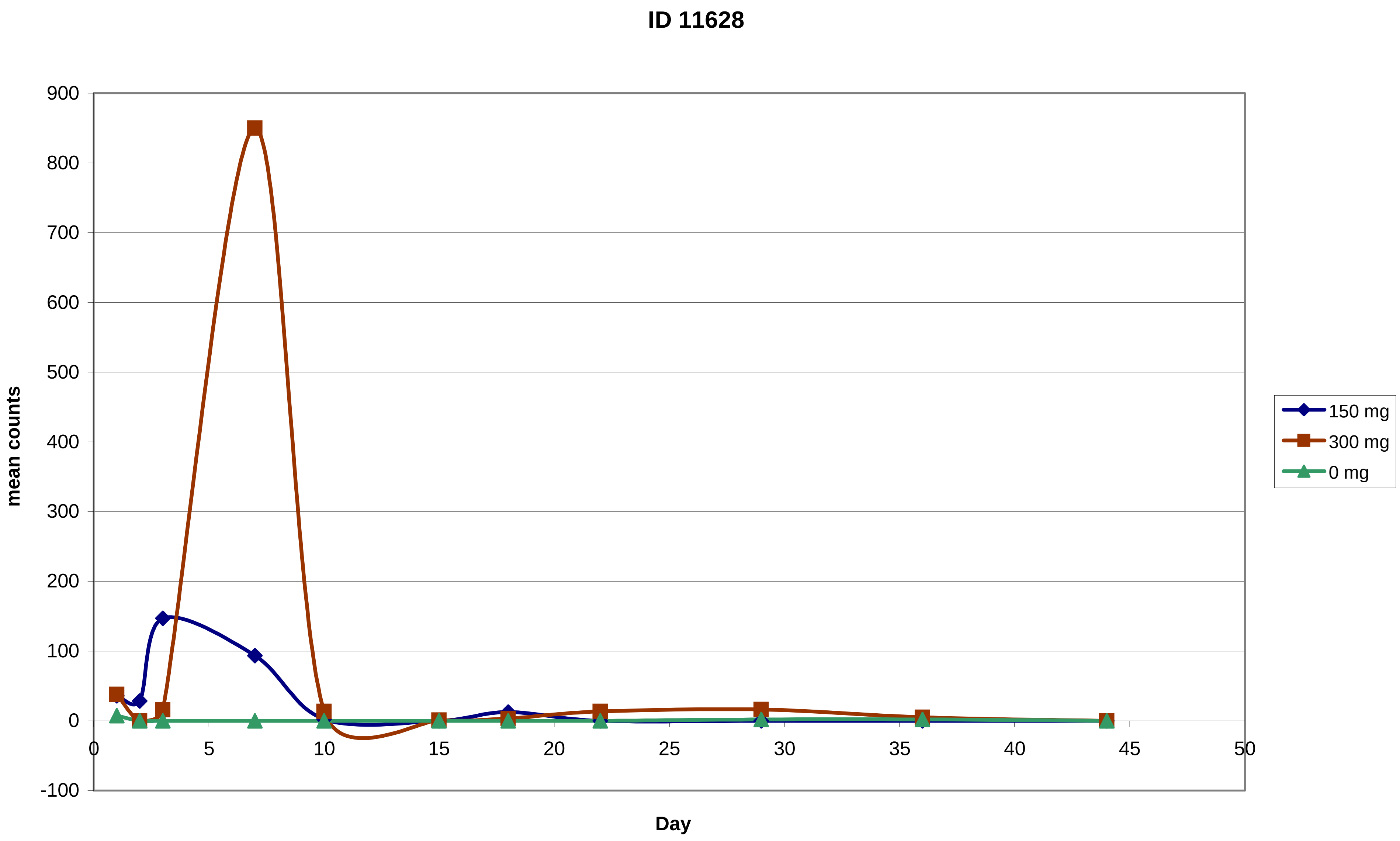
ID 11628
mean counts
900
800
700
600
500
400
300
200
100
0
-100
0
5
10
15
20
25
30
35
40
45
50
Day
150 mg
300 mg
0 mg

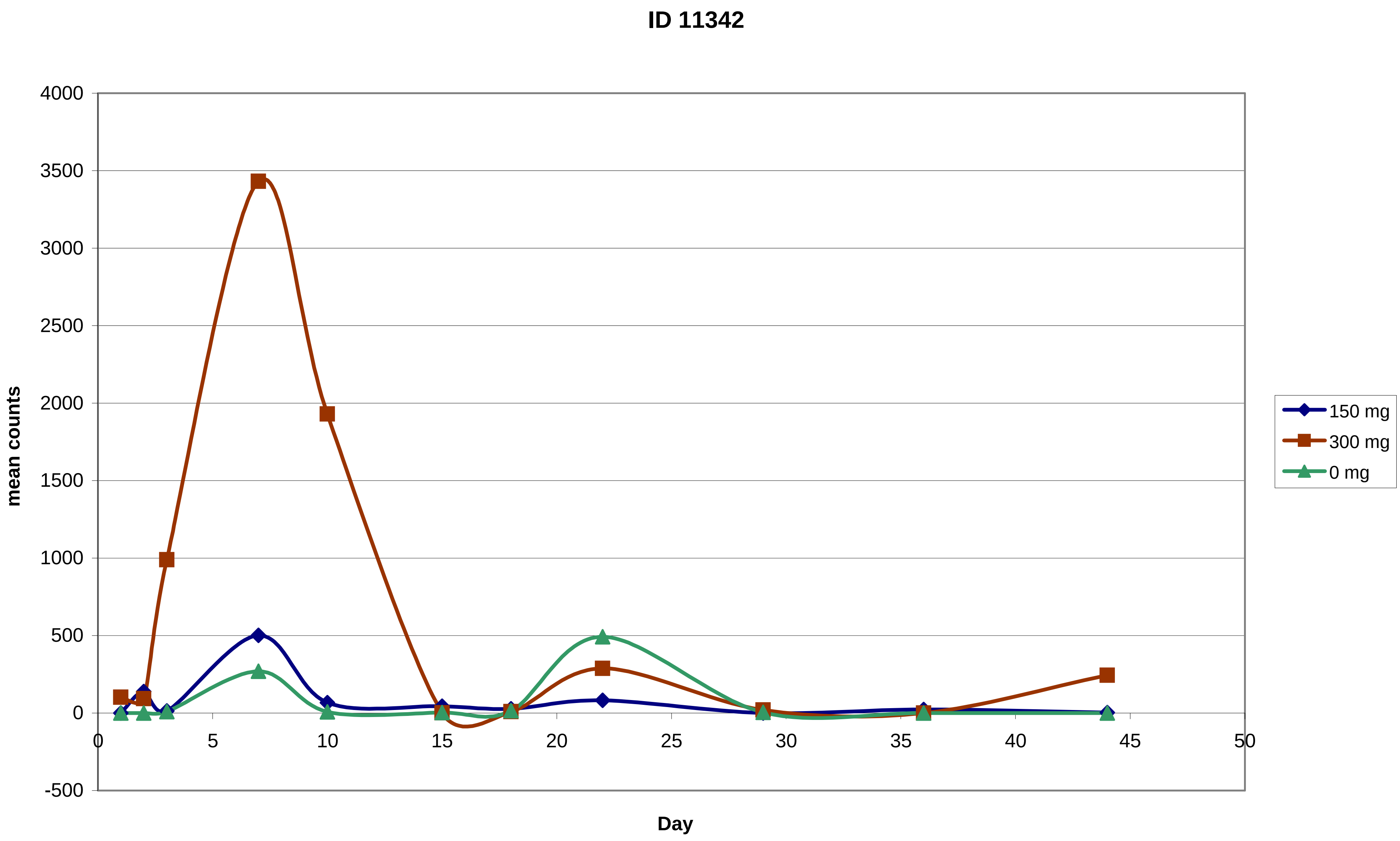
ID 11342
mean counts
4000
3500
3000
2500
2000
1500
1000
500
0
-500
0
5
10
15
20
25
30
35
40
45
50
Day
150 mg
300 mg
0 mg

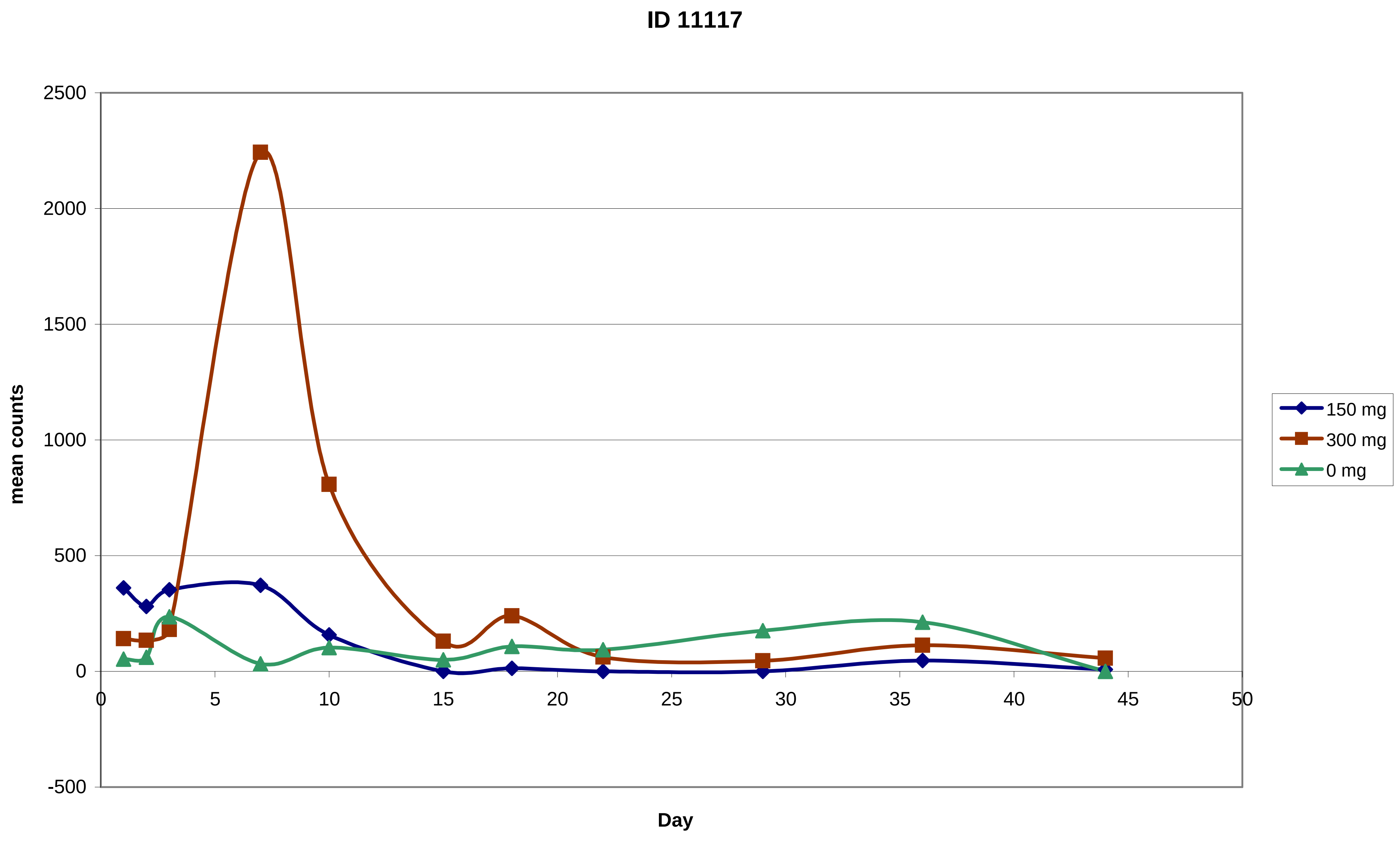
ID 11117
mean counts
2500
2000
1500
1000
500
0
-500
0
5
10
15
20
25
30
35
40
45
50
Day
150 mg
300 mg
0 mg

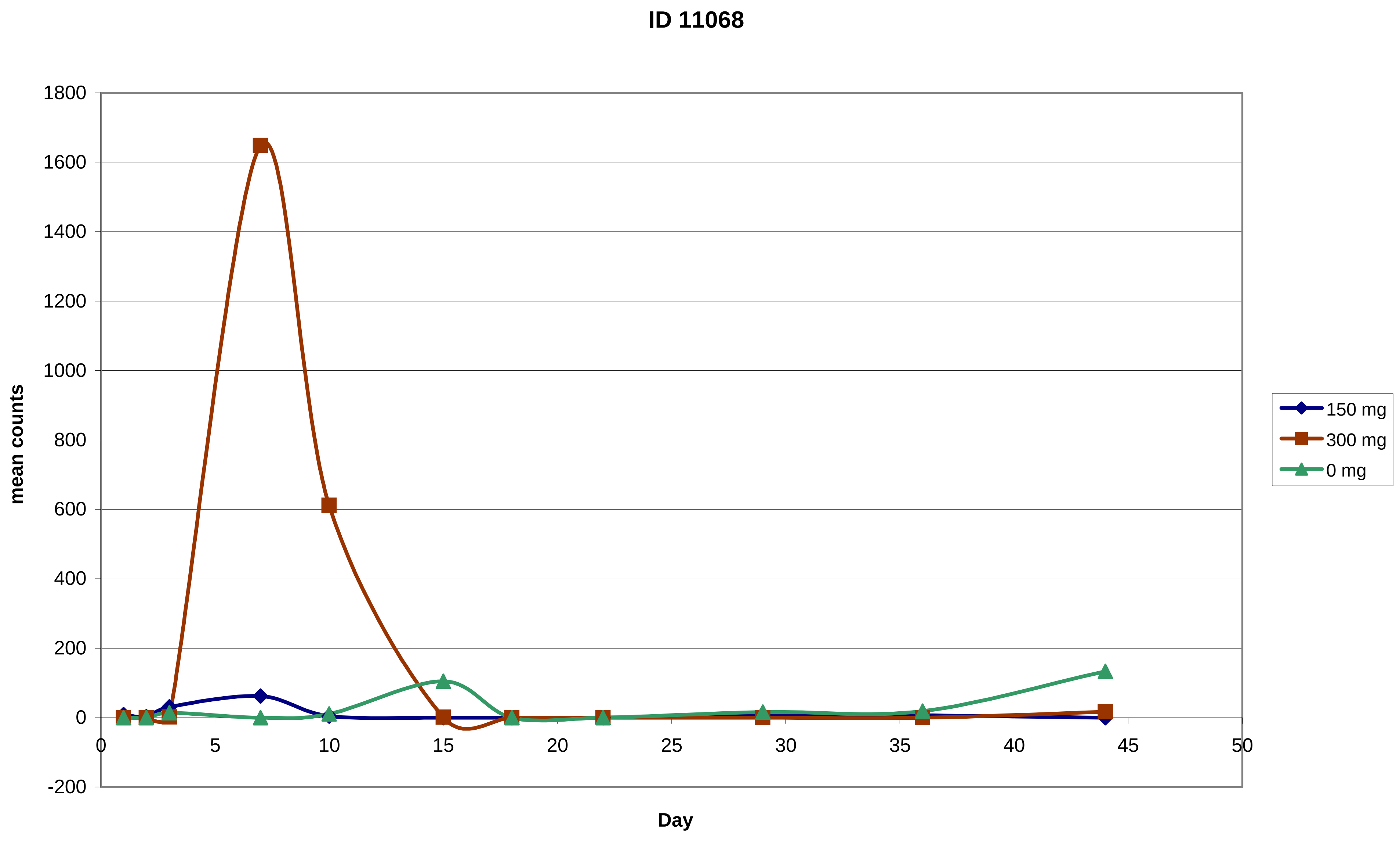
ID 11068
mean counts
Day
1800
1600
1400
1200
1000
800
600
400
200
0
-200
0
5
10
15
20
25
30
35
40
45
50
150 mg
300 mg
0 mg

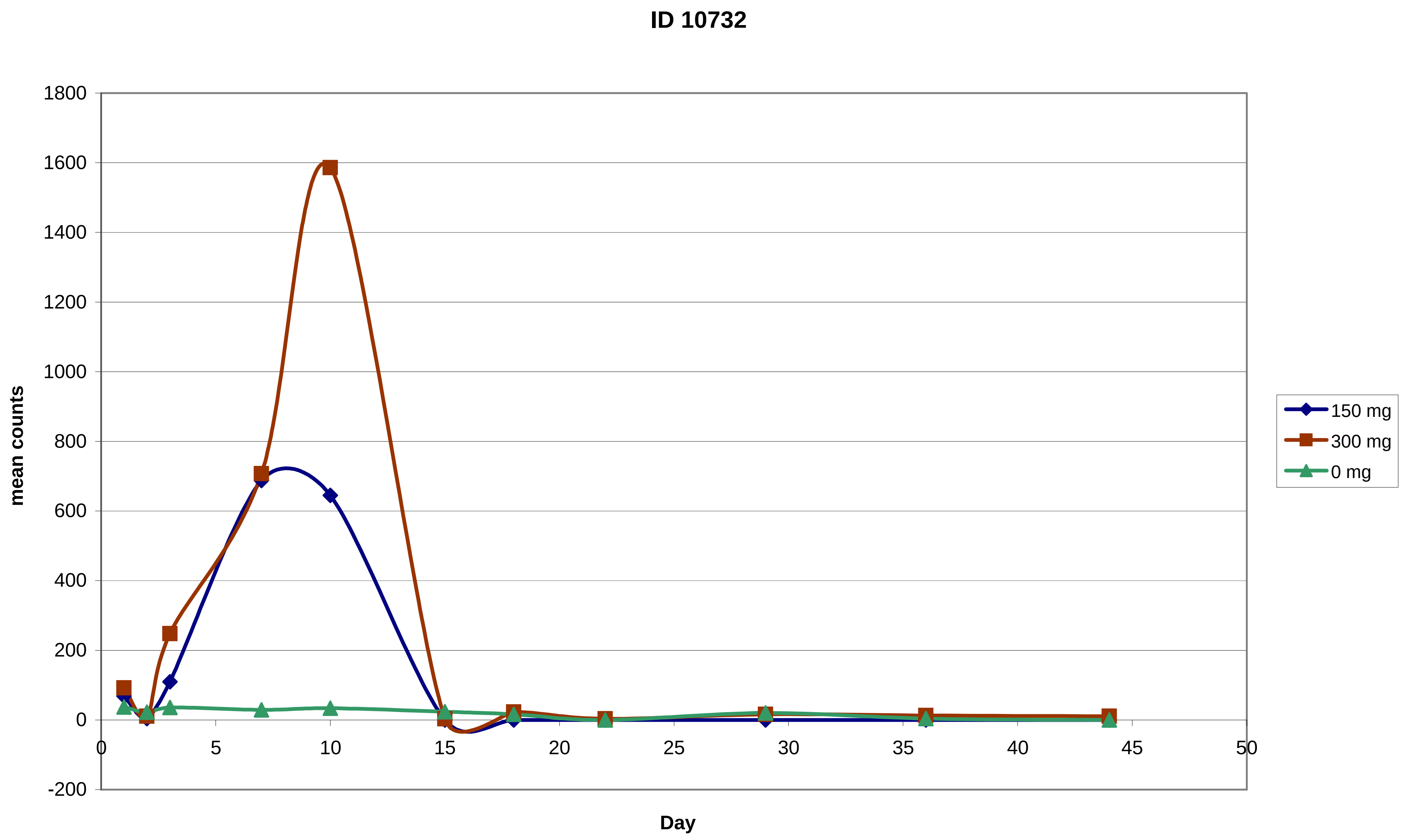

ID 10732
mean counts
1800
1600
1400
1200
1000
800
600
400
200
0
-200
0
5
10
15
20
25
30
35
40
45
50
Day
150 mg
300 mg
0 mg

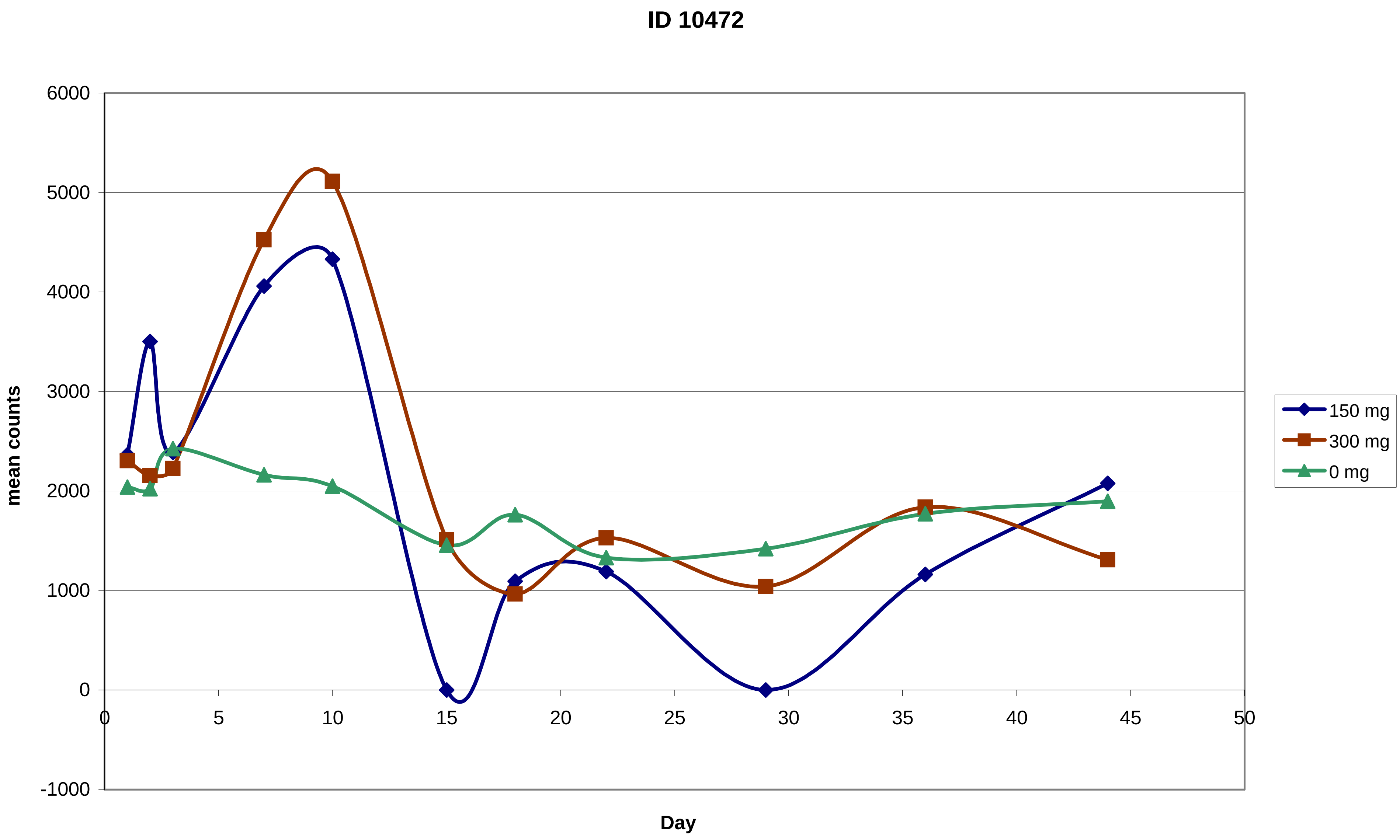
ID 10472
mean counts
6000
5000
4000
3000
2000
1000
0
-1000
0
5
10
15
20
25
30
35
40
45
50
Day
150 mg
300 mg
0 mg

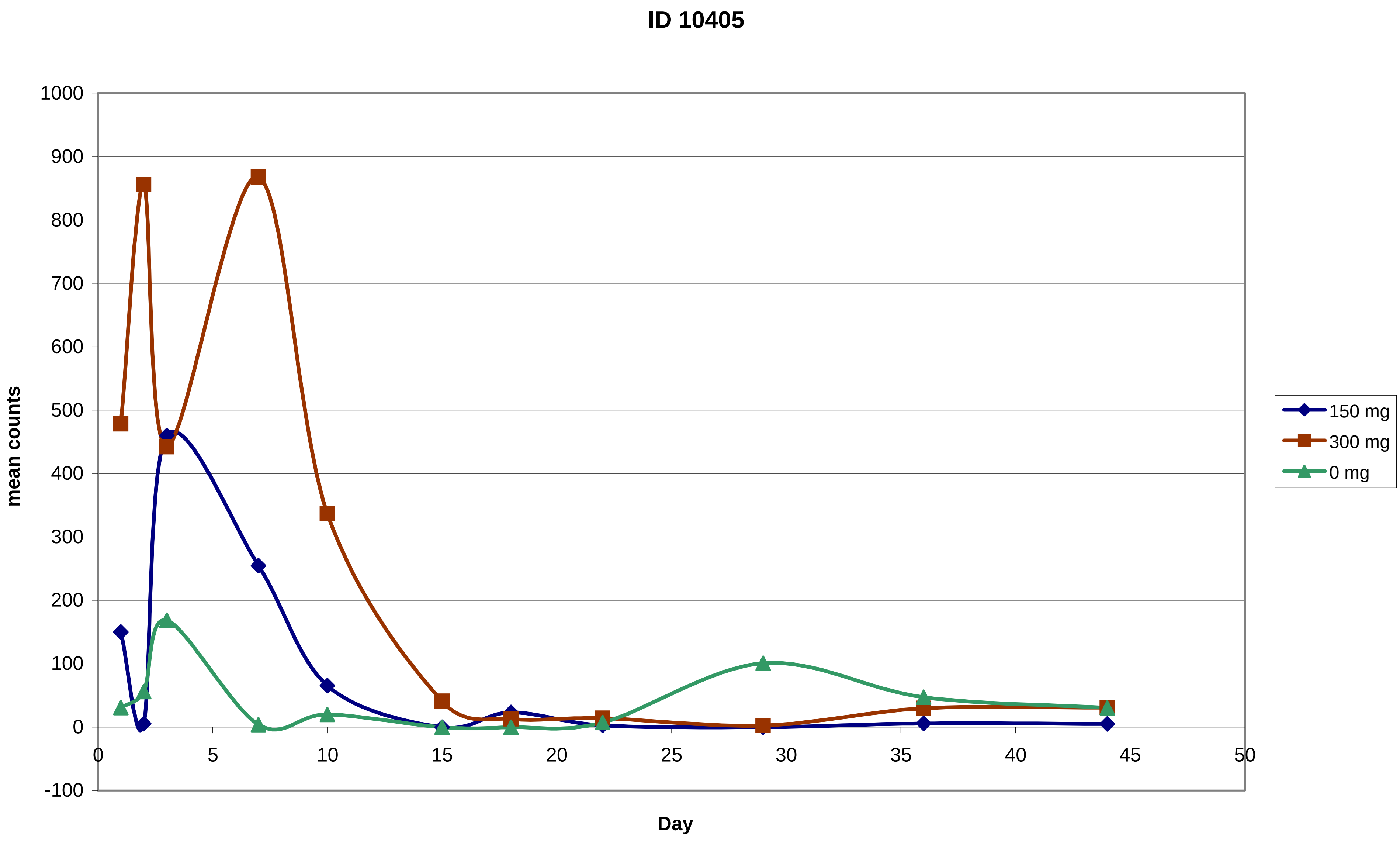

ID 10405
mean counts
1000
900
800
700
600
500
400
300
200
100
0
-100
0
5
10
15
20
25
30
35
40
45
50
Day
150 mg
300 mg
0 mg

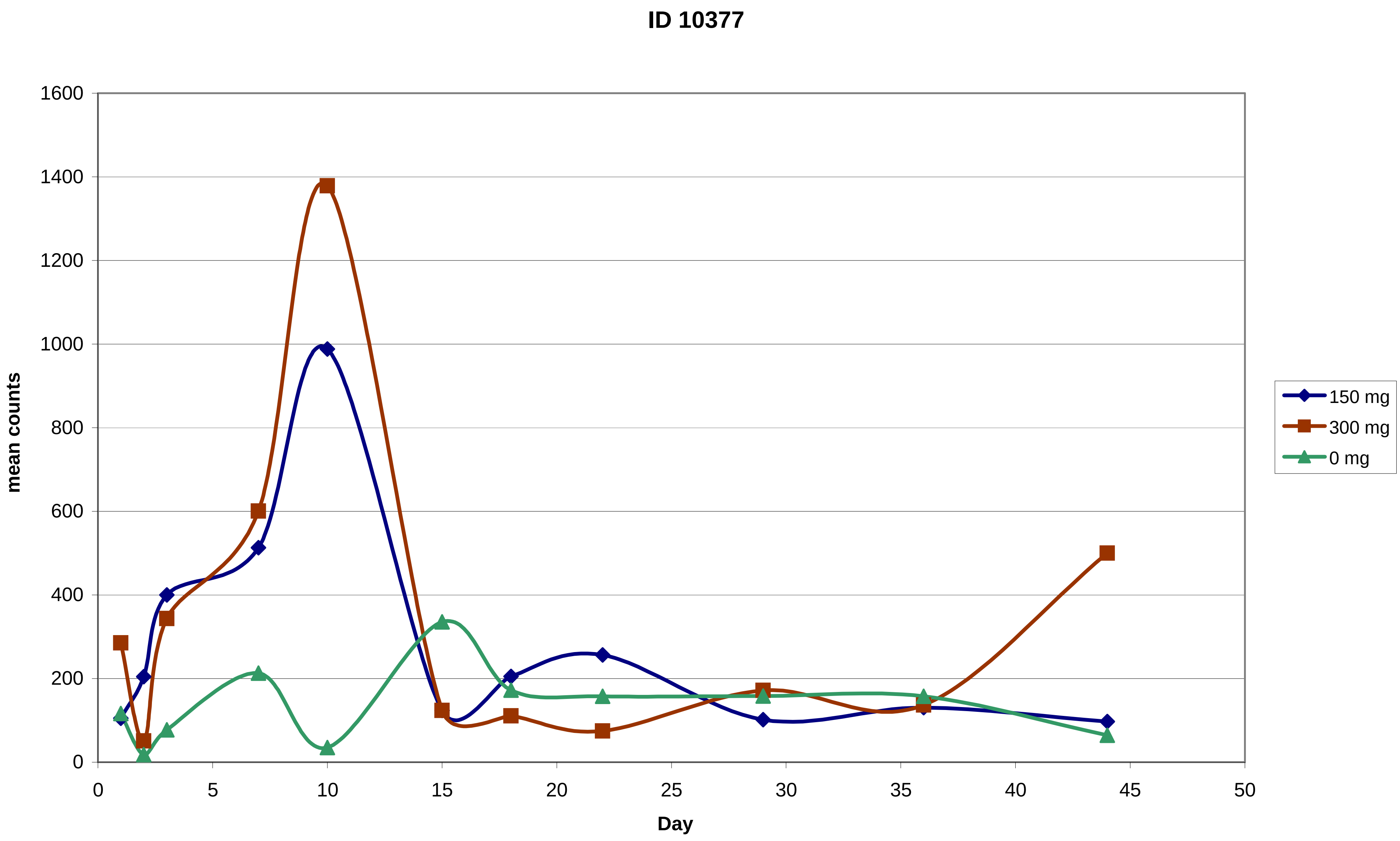

ID 10377
mean counts
1600
1400
1200
1000
800
600
400
200
0
0
5
10
15
20
25
30
35
40
45
50
Day
150 mg
300 mg
0 mg

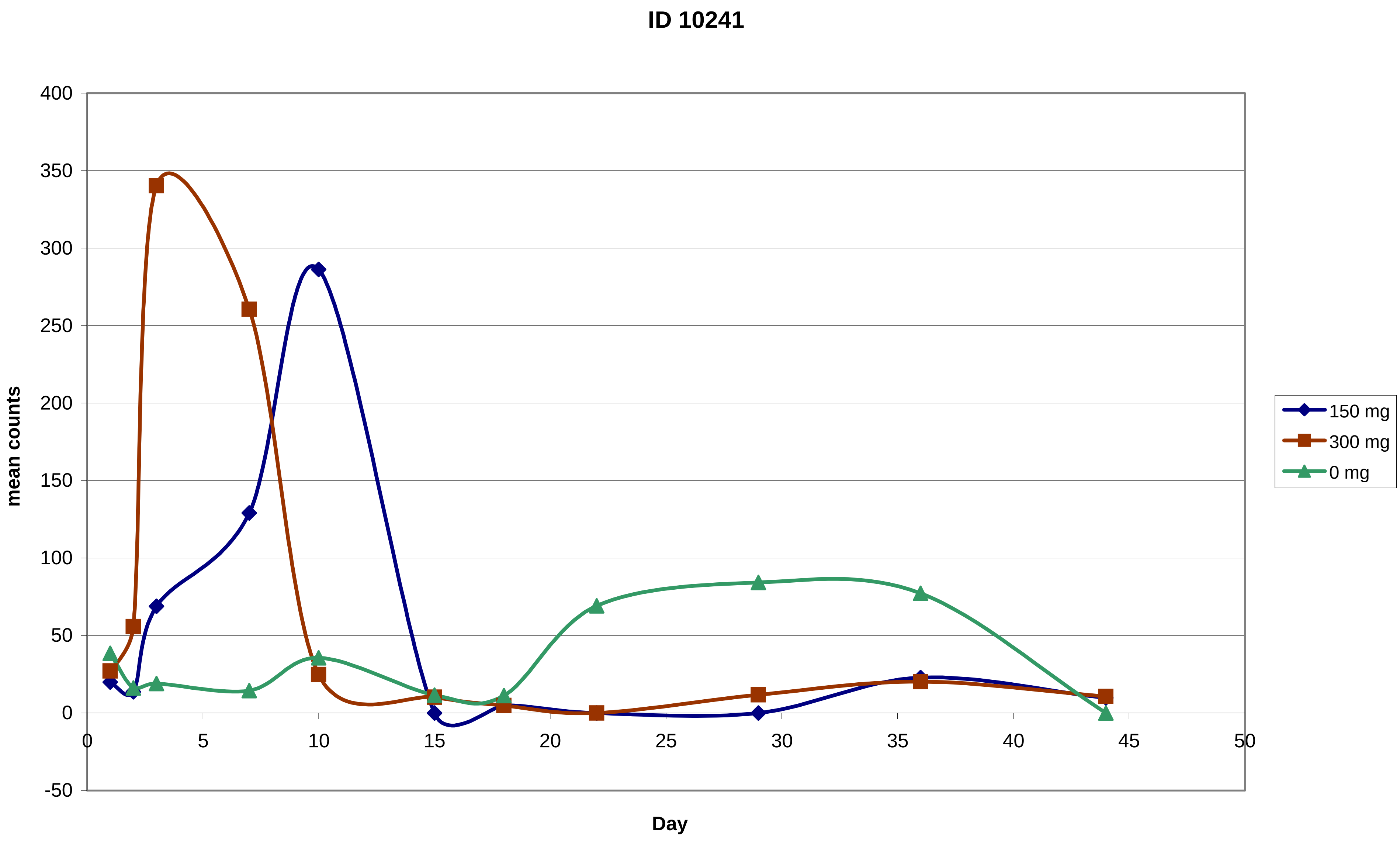
ID 10241
mean counts
400
350
300
250
200
150
100
50
0
-50
0
5
10
15
20
25
30
35
40
45
50
Day
150 mg
300 mg
0 mg

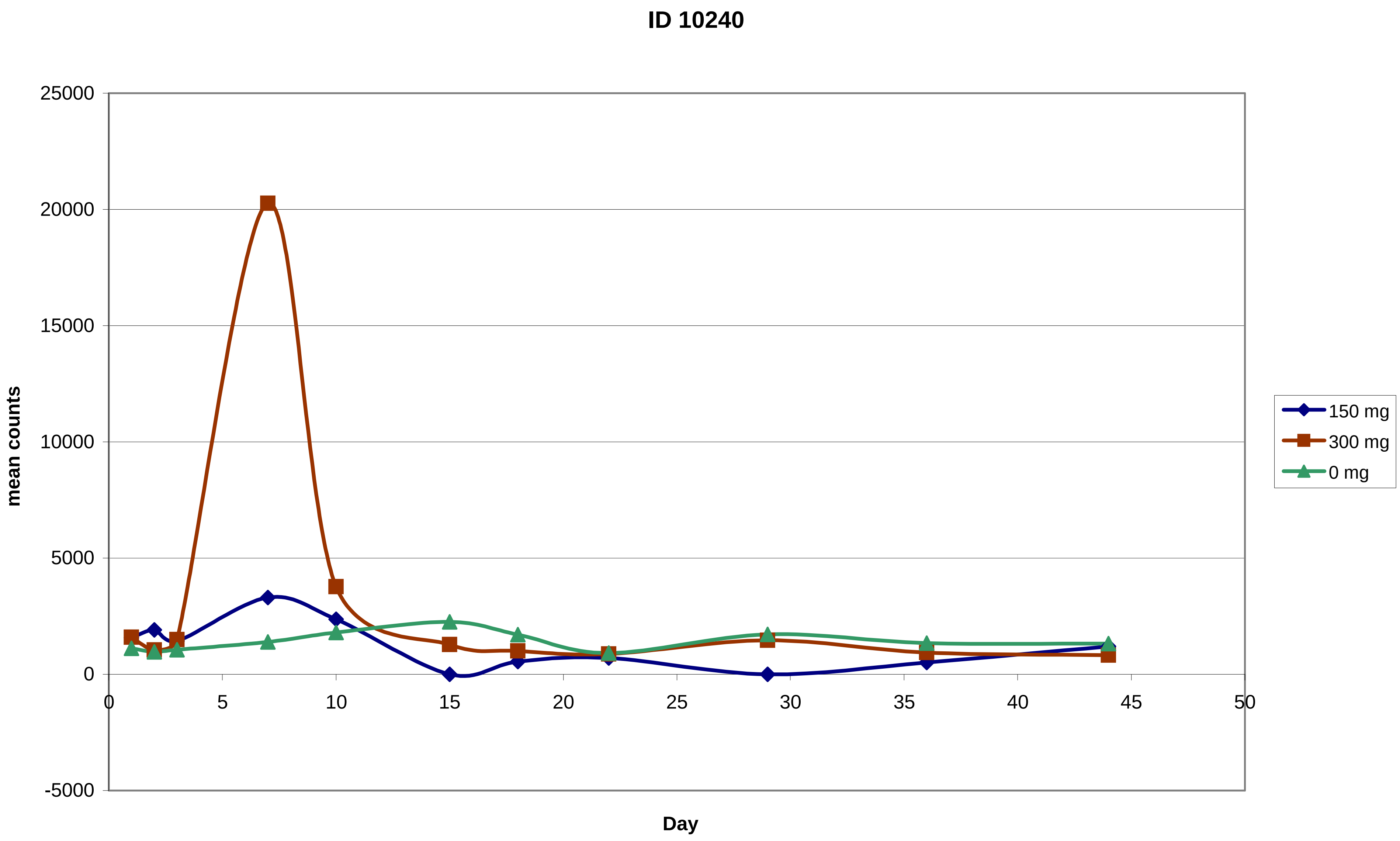
ID 10240
mean counts
25000
20000
15000
10000
5000
0
-5000
0
5
10
15
20
25
30
35
40
45
50
Day
150 mg
300 mg
0 mg

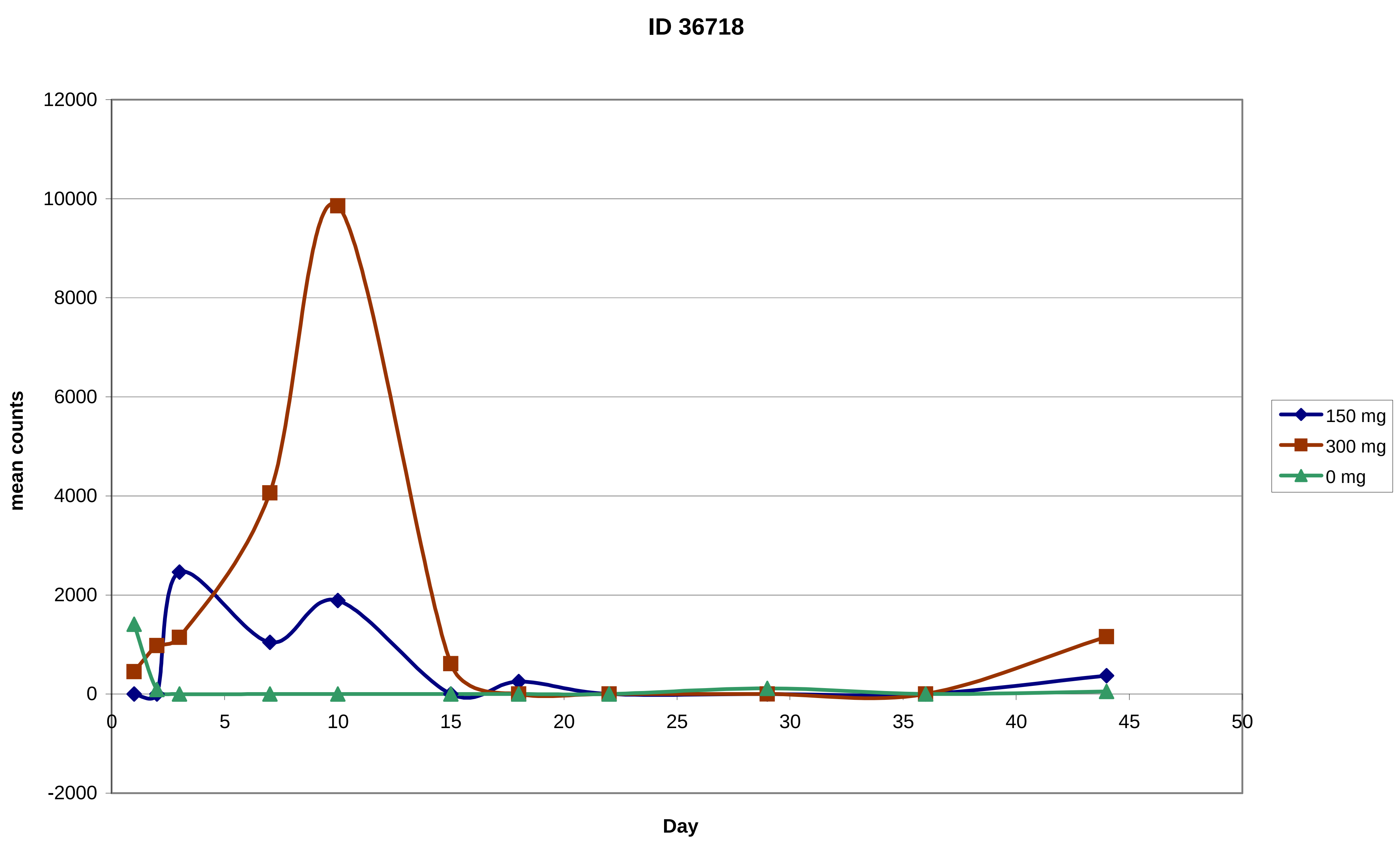
ID 36718
12000
10000
8000
6000
4000
2000
0
-2000
mean counts
0
5
10
15
20
25
30
35
40
45
50
Day
150 mg
300 mg
0 mg

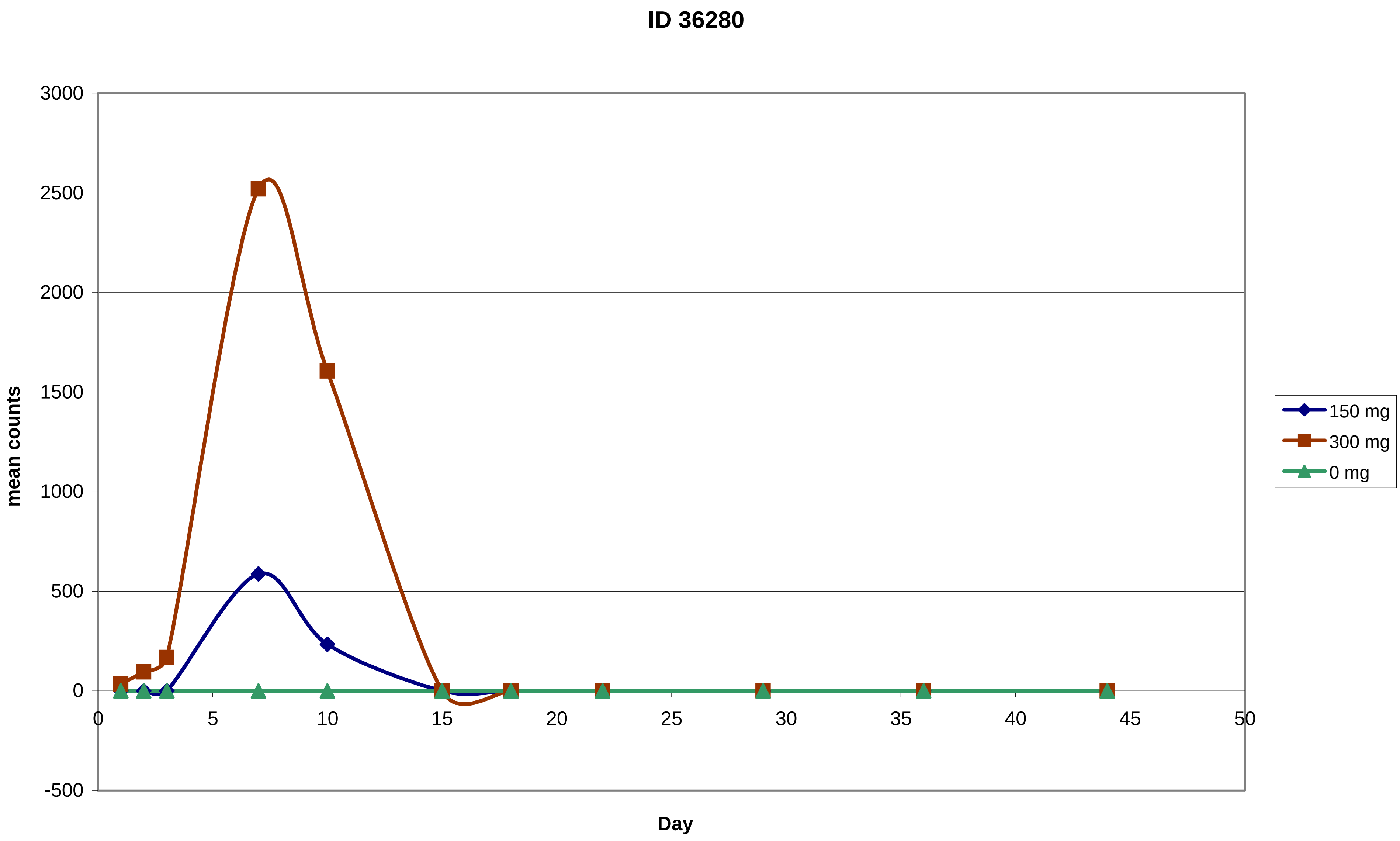

ID 36280
mean counts
3000
2500
2000
1500
1000
500
0
-500
0
5
10
15
20
25
30
35
40
45
50
Day
150 mg
300 mg
0 mg

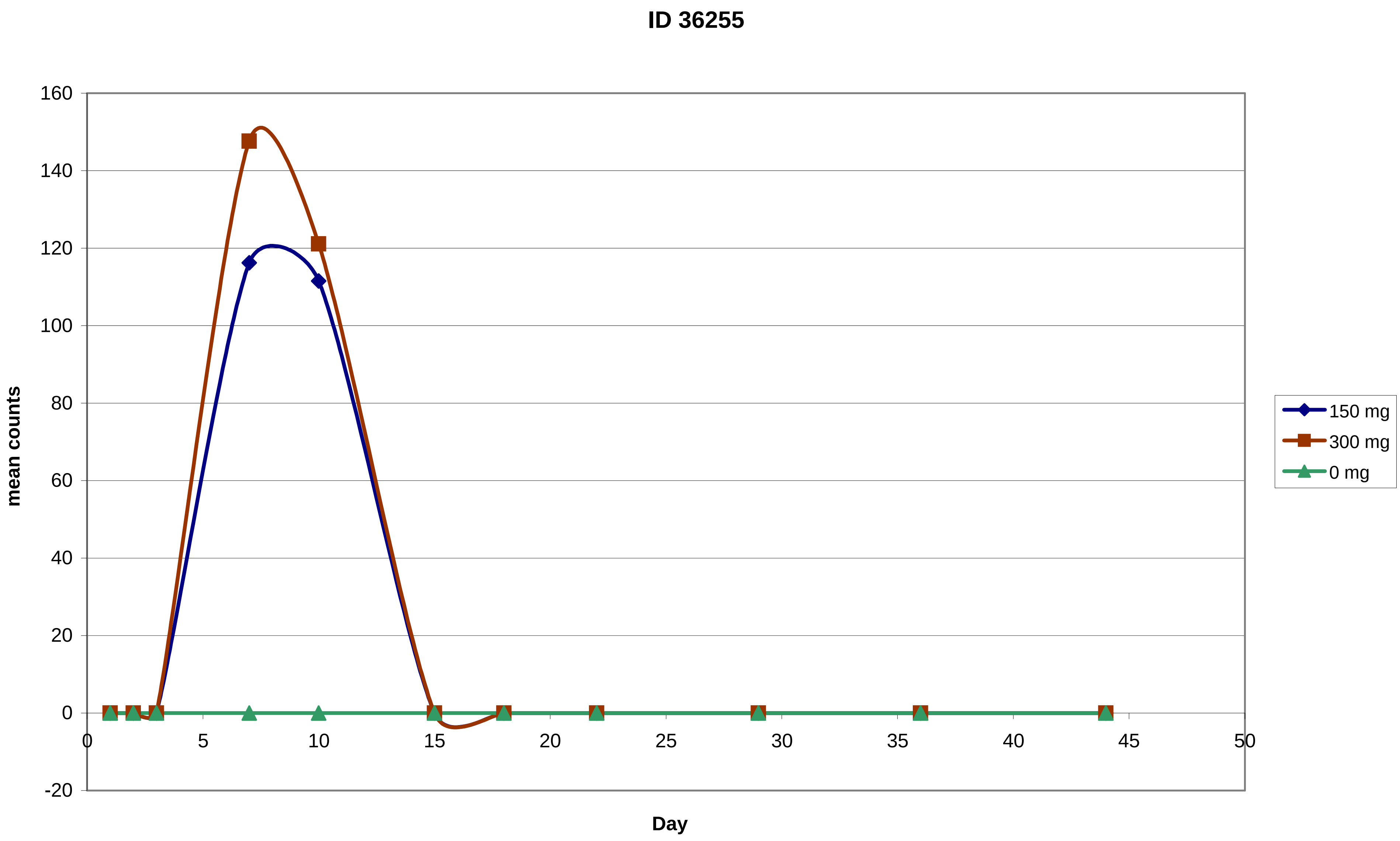
ID 36255
mean counts
160
140
120
100
80
60
40
20
0
-20
0
5
10
15
20
25
30
35
40
45
50
Day
150 mg
300 mg
0 mg

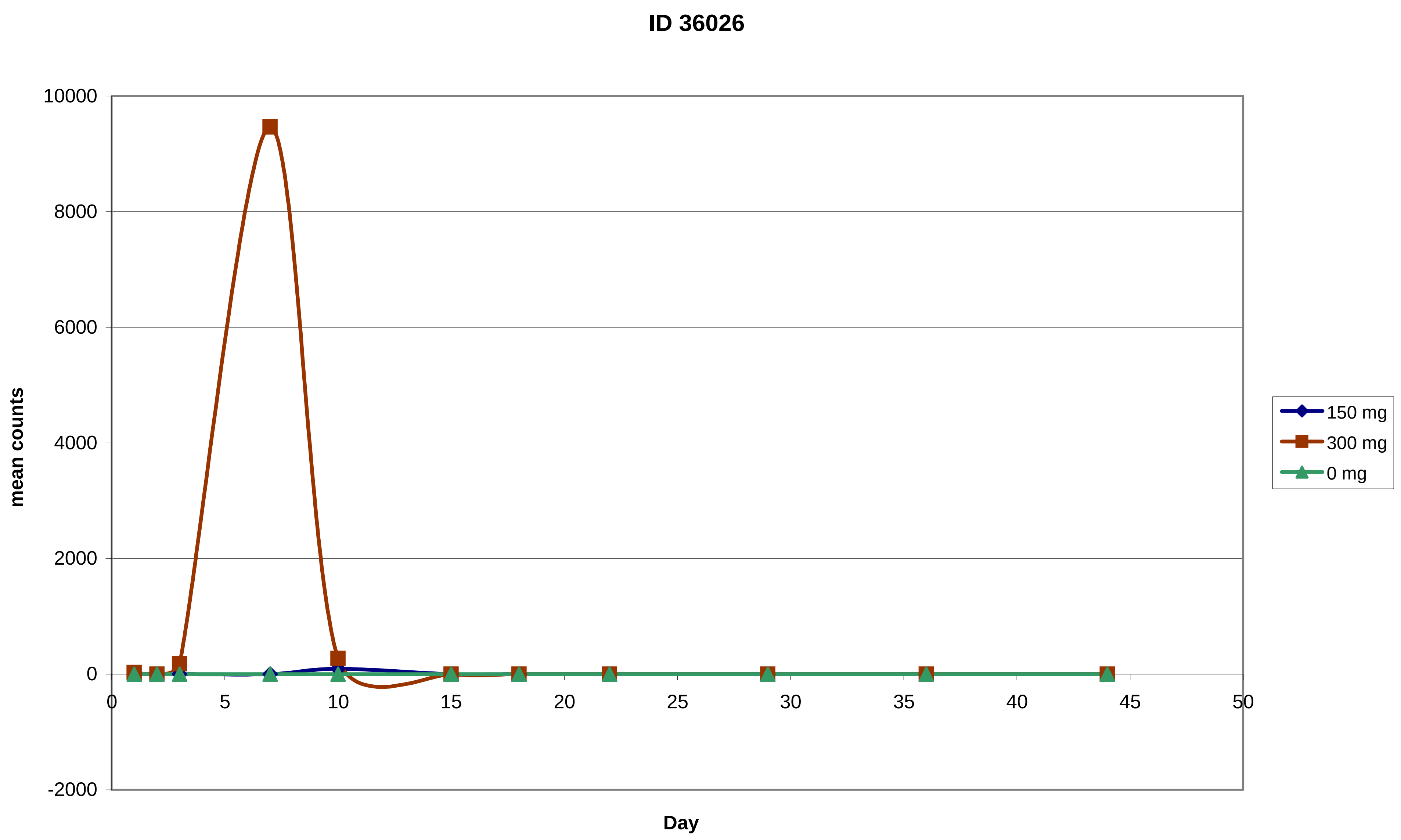
ID 36026
mean counts
10000
8000
6000
4000
2000
0
-2000
0
5
10
15
20
25
30
35
40
45
50
Day
150 mg
300 mg
0 mg

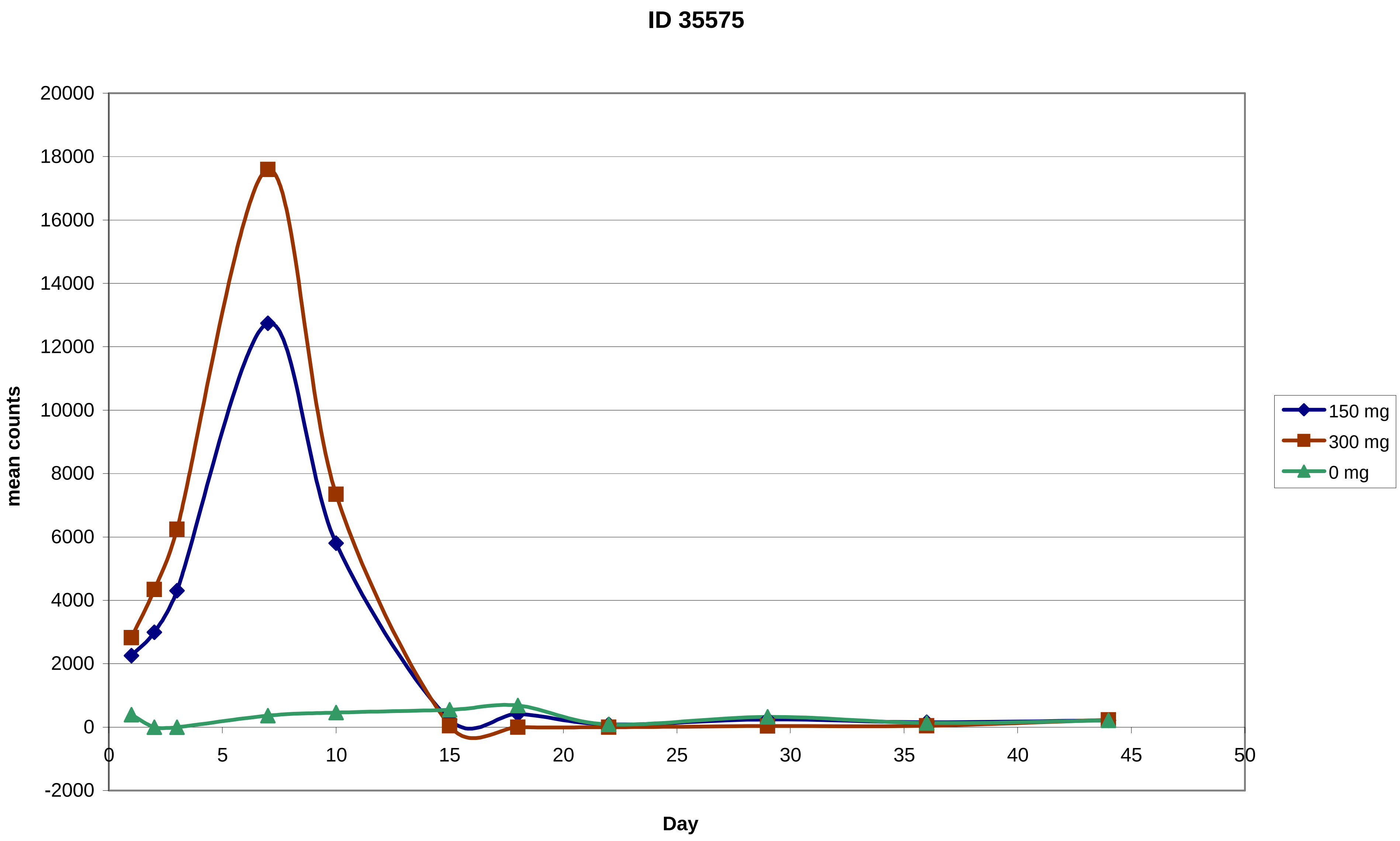

ID 35575
mean counts
20000
18000
16000
14000
12000
10000
8000
6000
4000
2000
0
-2000
0
5
10
15
20
25
30
35
40
45
50
Day
150 mg
300 mg
0 mg

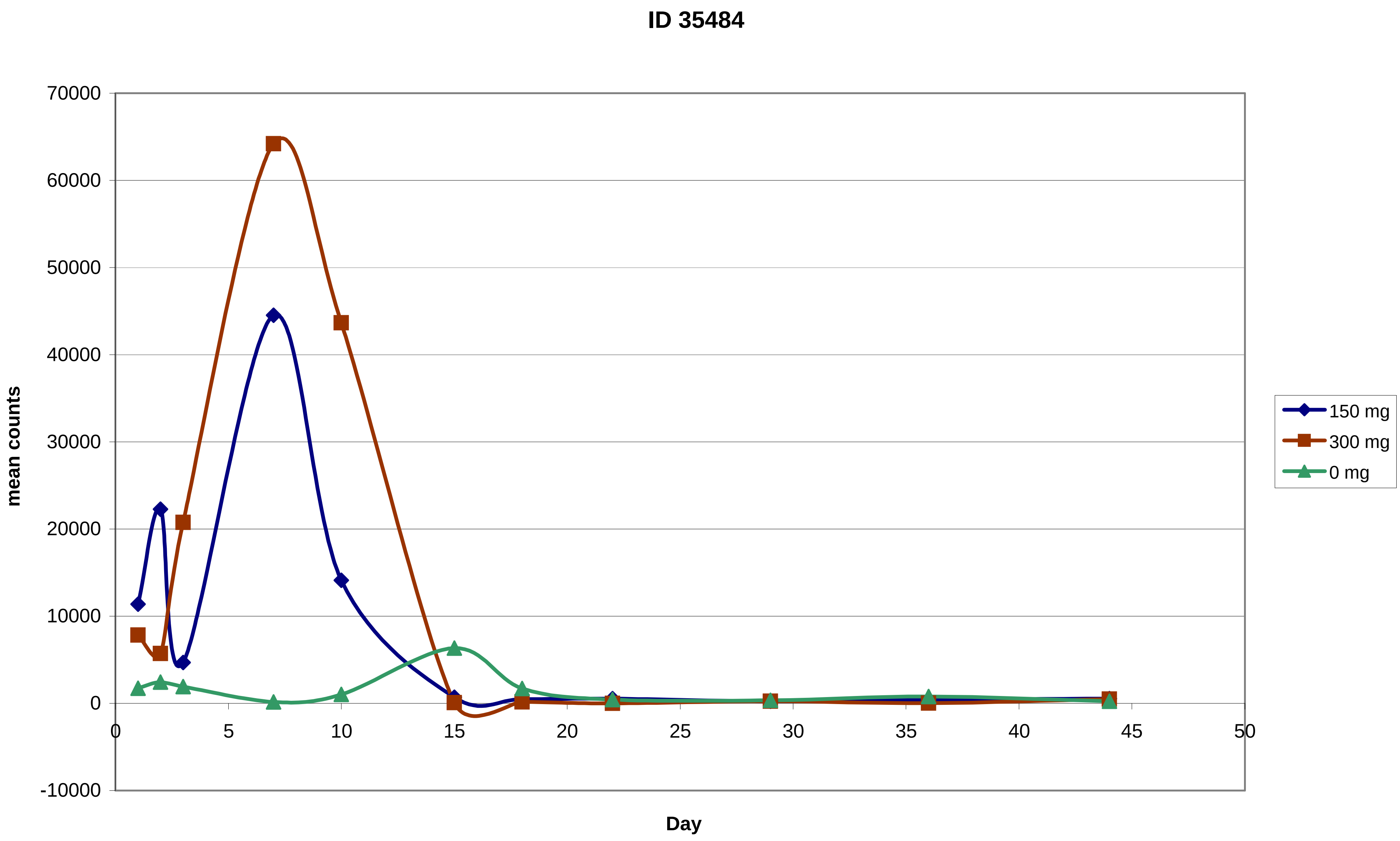

ID 35484
mean counts
70000
60000
50000
40000
30000
20000
10000
0
-10000
0
5
10
15
20
25
30
35
40
45
50
Day
150 mg
300 mg
0 mg

ID 34960

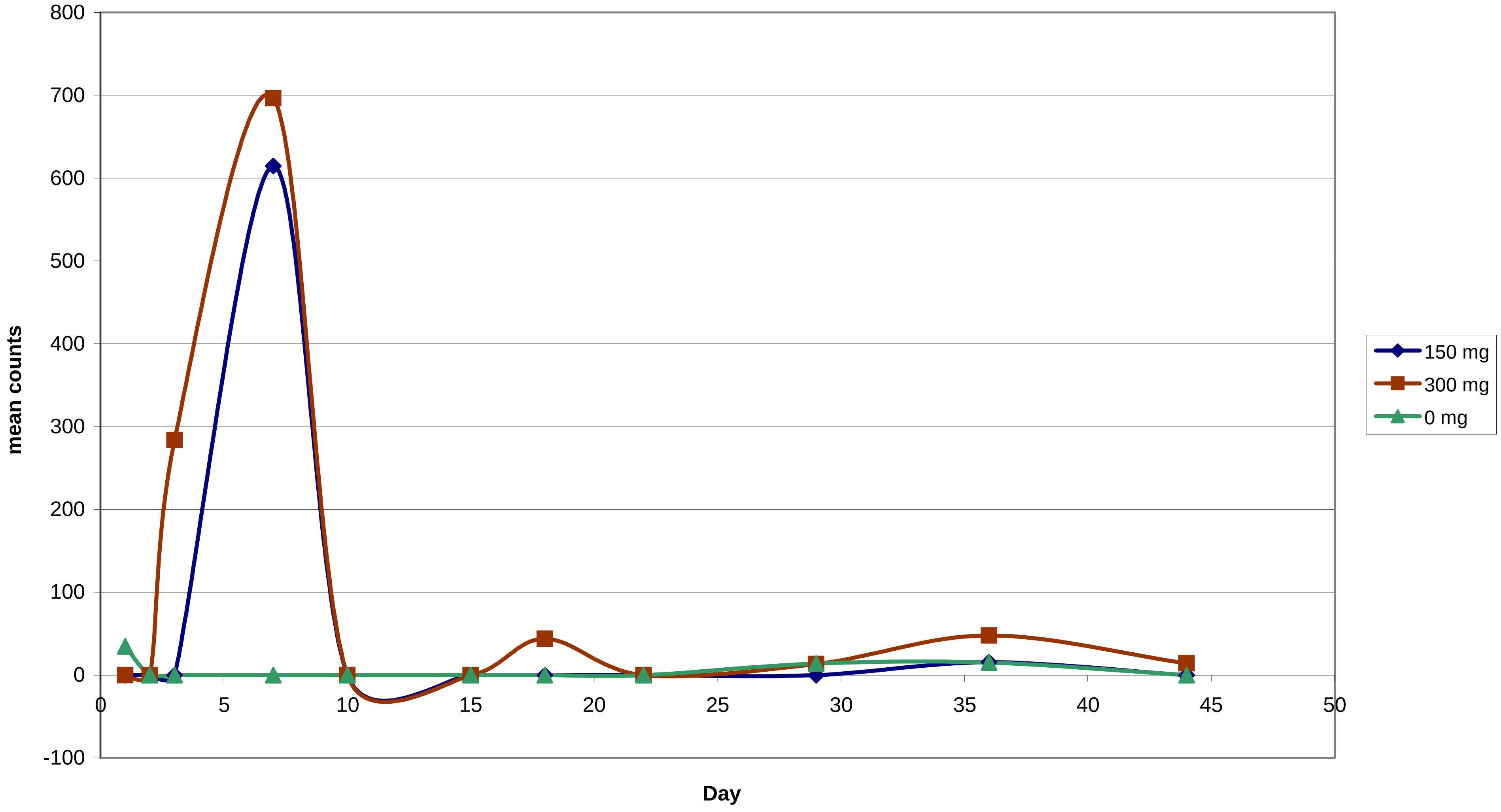

mean counts
800
700
600
500
400
300
200
100
0
-100
0
5
10
15
20
25
30
35
40
45
50
Day
150 mg
300 mg
0 mg

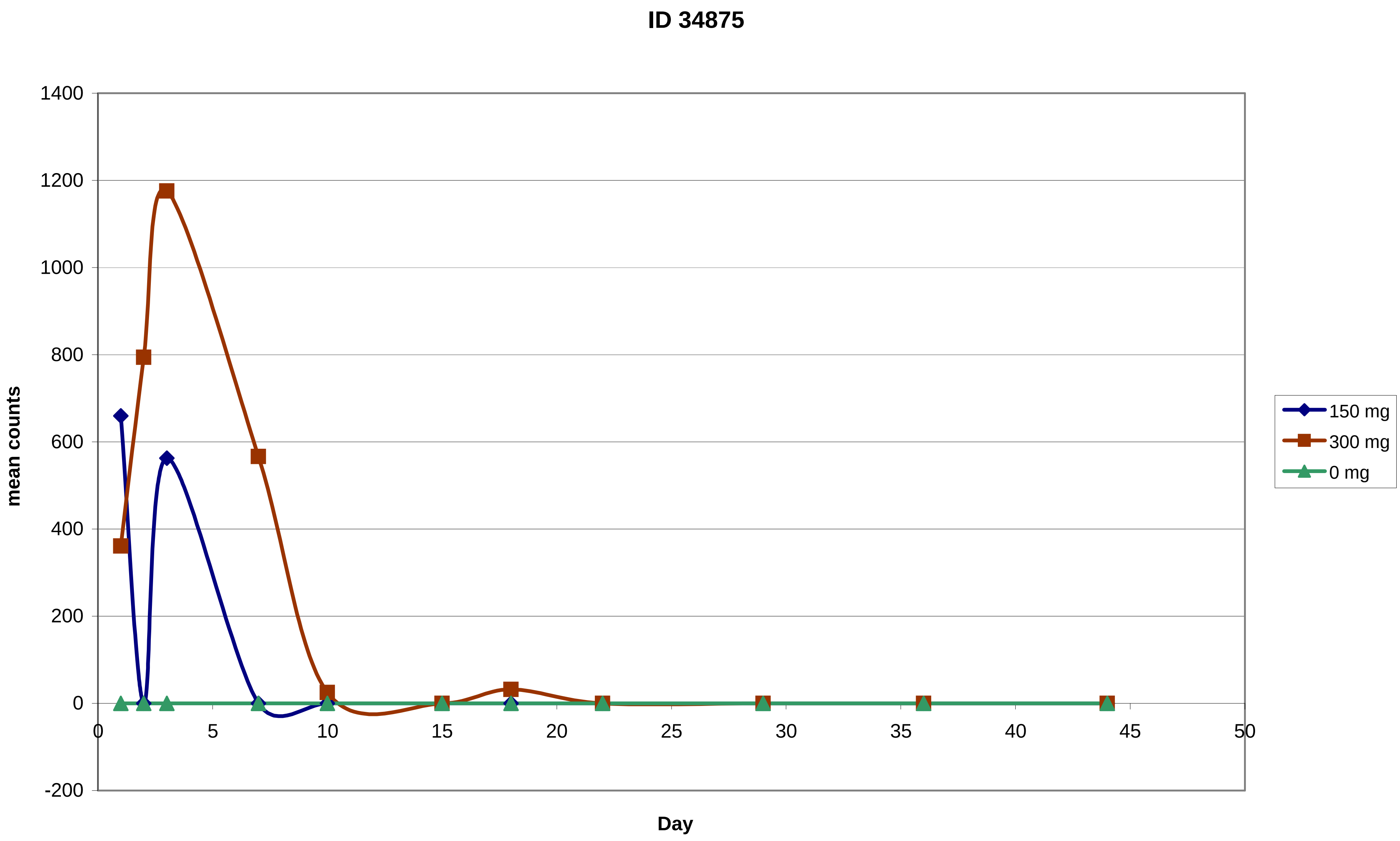
ID 34875
mean counts
1400
1200
1000
800
600
400
200
0
-200
0
5
10
15
20
25
30
35
40
45
50
Day
150 mg
300 mg
0 mg

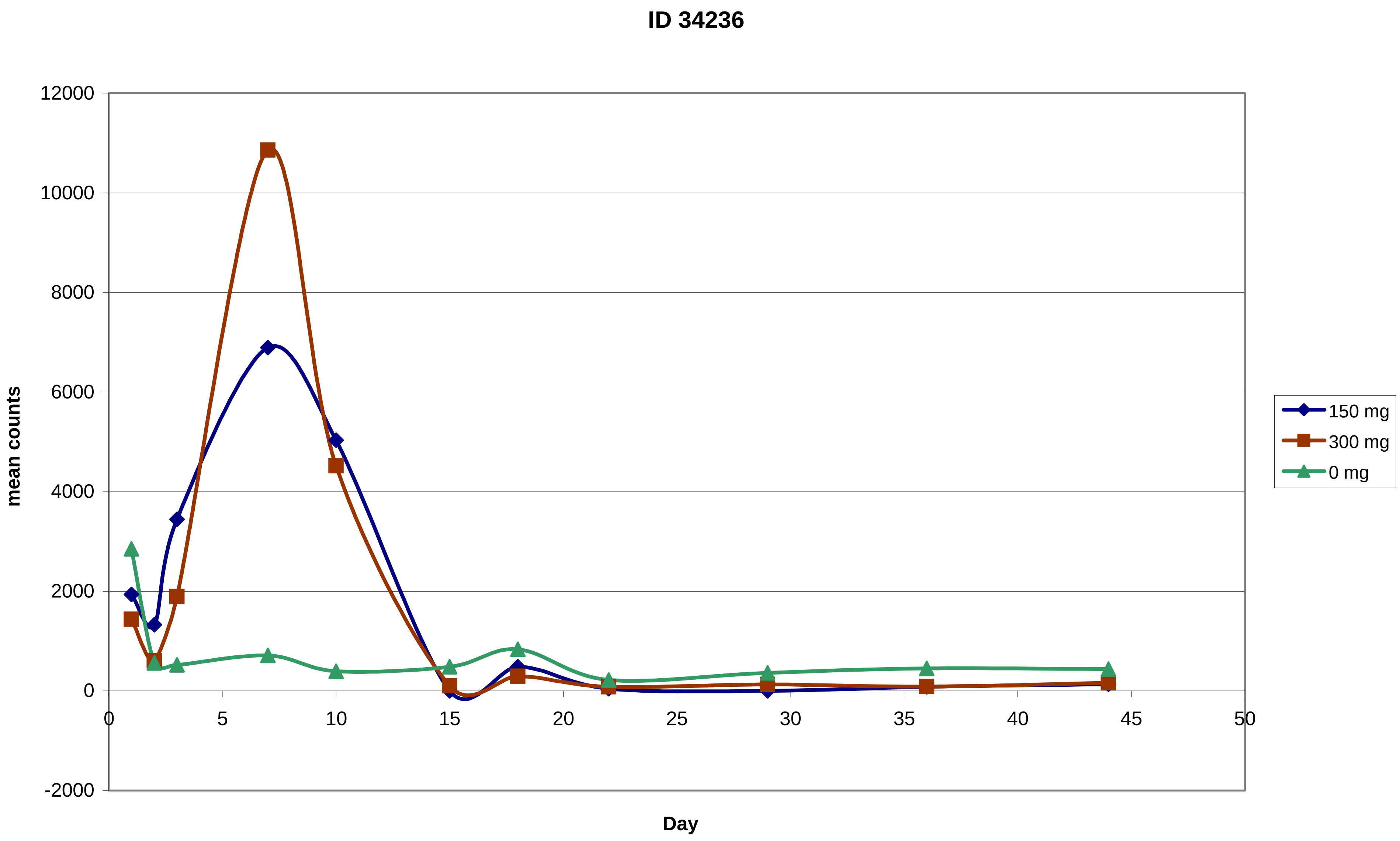

ID 34236
mean counts
12000
10000
8000
6000
4000
2000
0
-2000
0
5
10
15
20
25
30
35
40
45
50
Day
150 mg
300 mg
0 mg

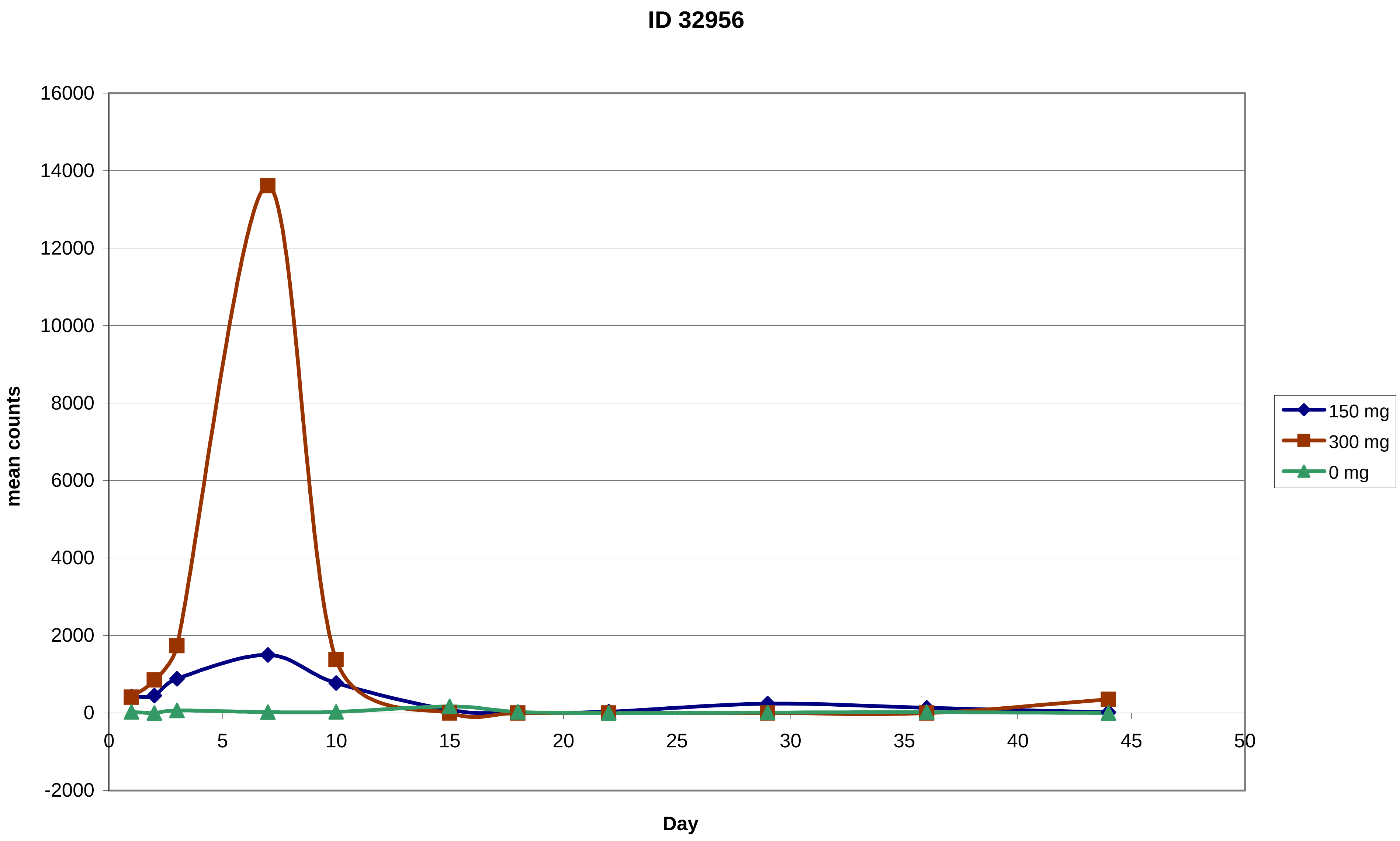

ID 32956
16000
14000
12000
10000
8000
6000
4000
2000
0
-2000
mean counts
0
5
10
15
20
25
30
35
40
45
50
Day
150 mg
300 mg
0 mg

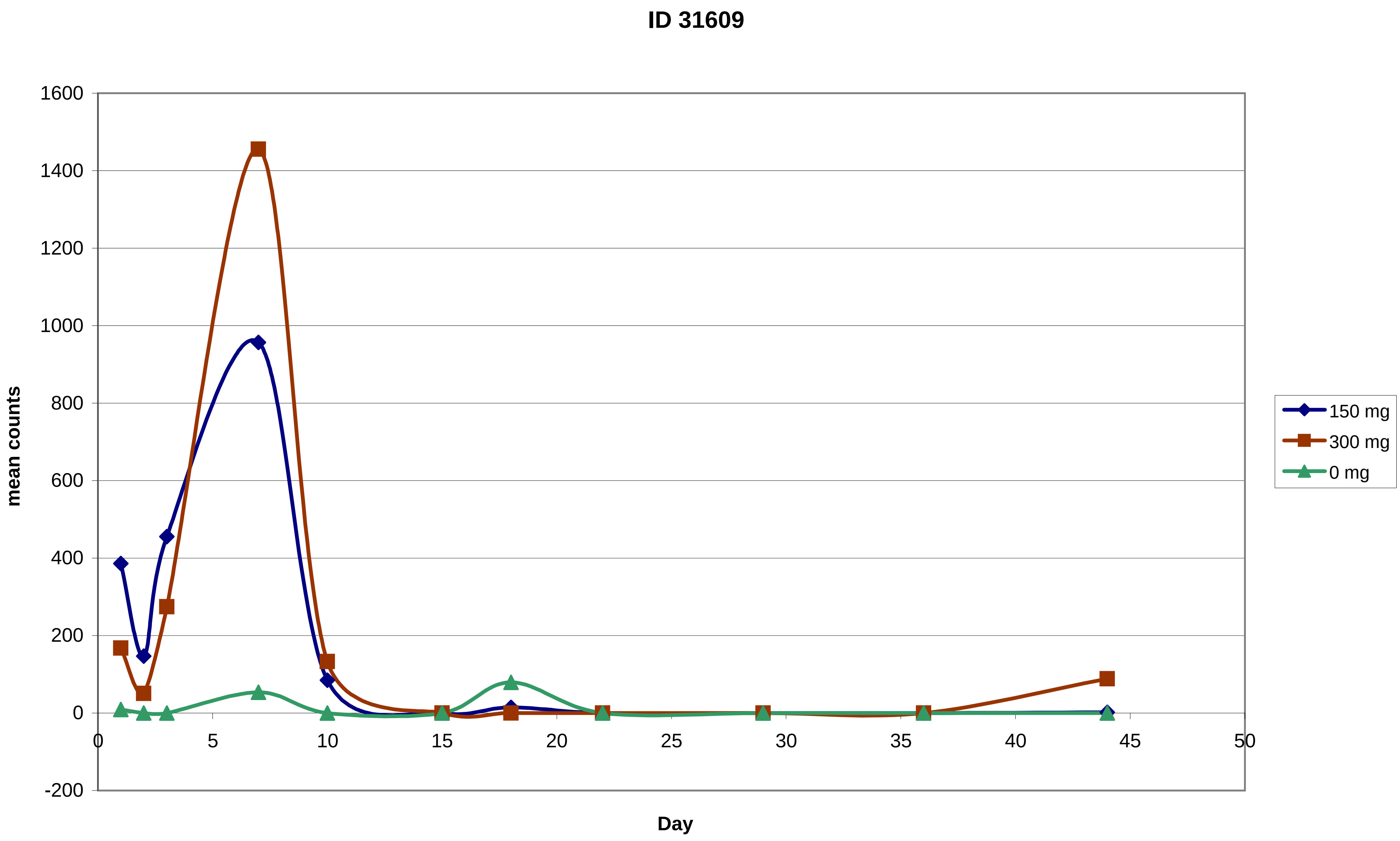
ID 31609
mean counts
1600
1400
1200
1000
800
600
400
200
0
-200
0
5
10
15
20
25
30
35
40
45
50
Day
150 mg
300 mg
0 mg

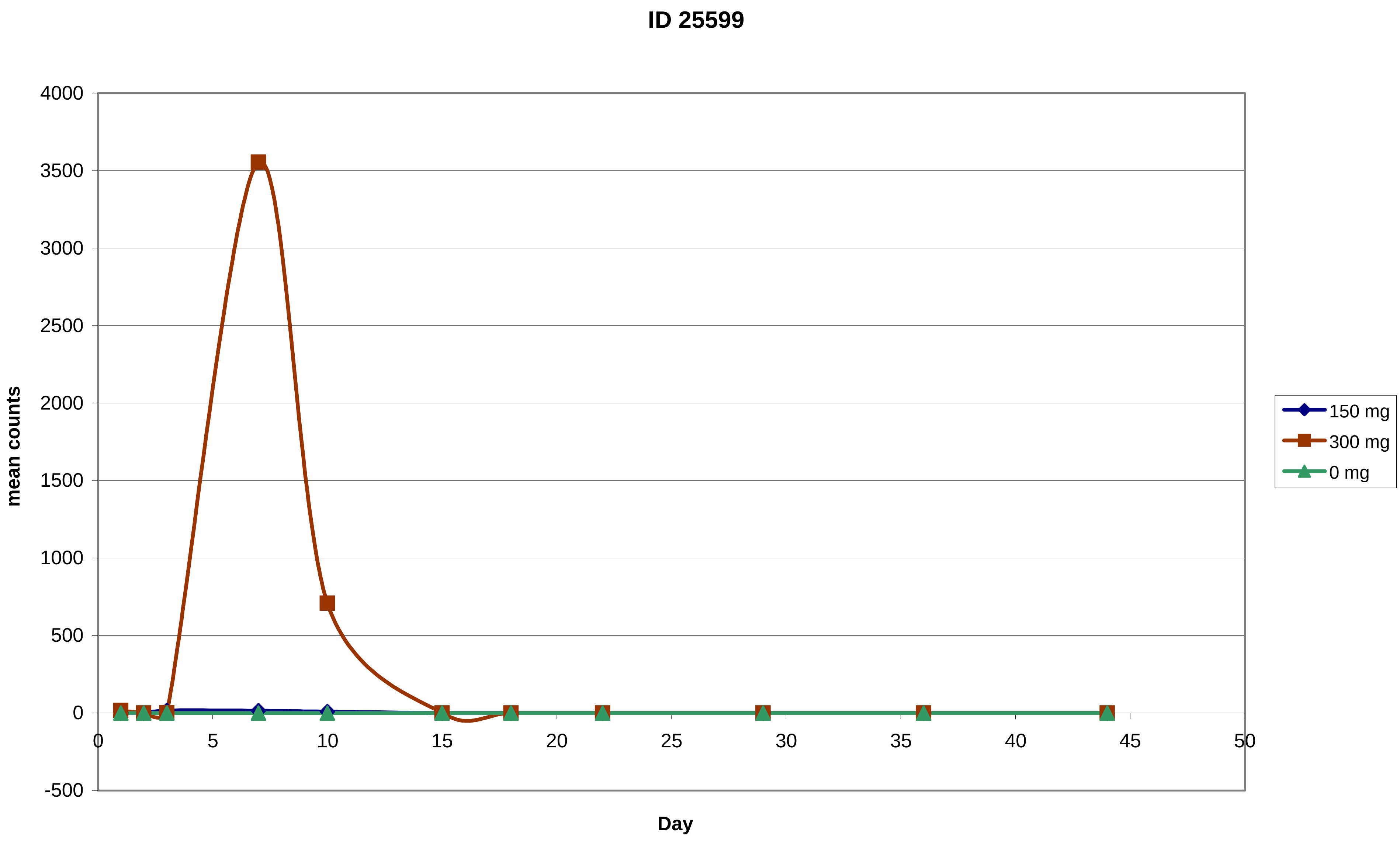
ID 25599
mean counts
4000
3500
3000
2500
2000
1500
1000
500
0
-500
0
5
10
15
20
25
30
35
40
45
50
Day
150 mg
300 mg
0 mg

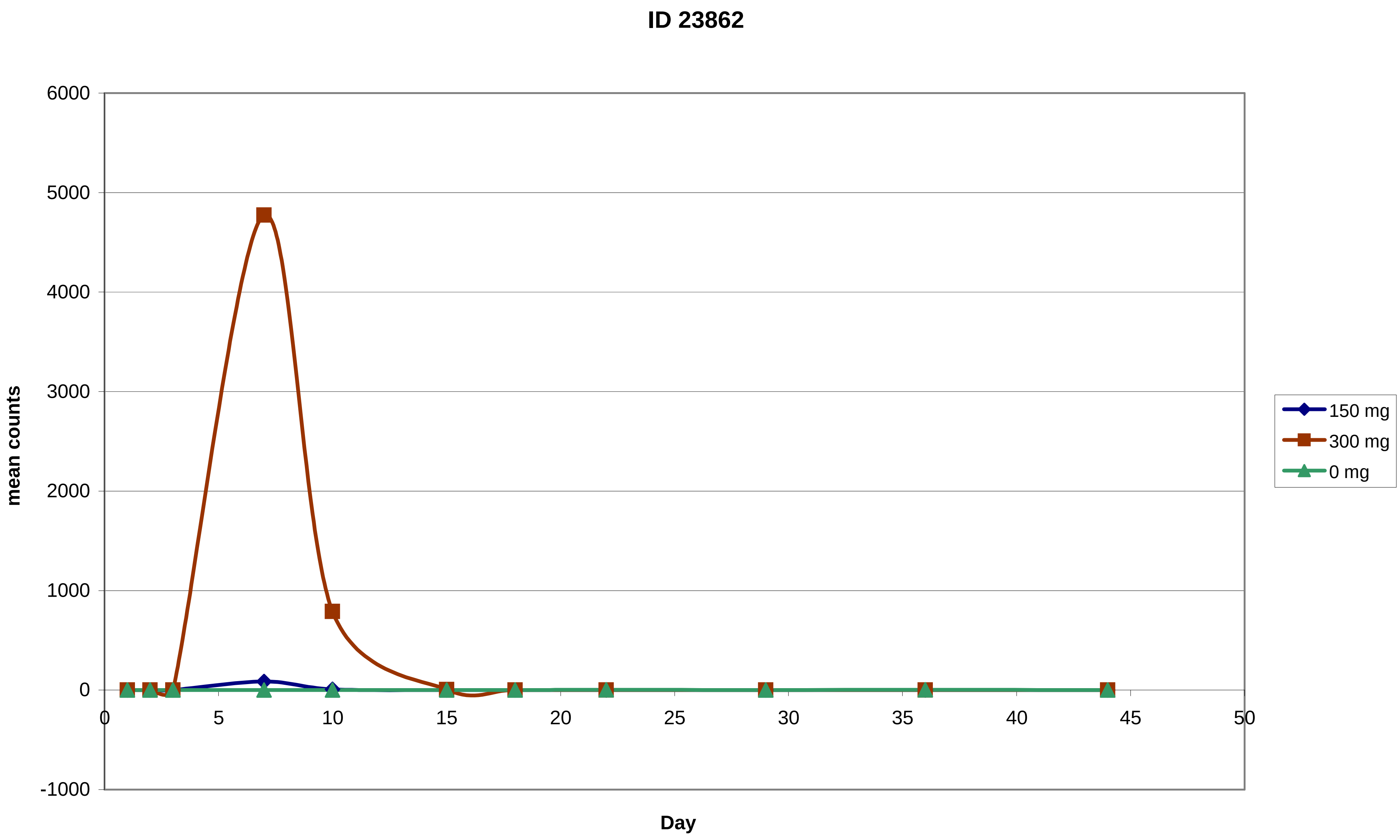
ID 23862
6000
5000
4000
3000
2000
1000
0
-1000
mean counts
0
5
10
15
20
25
30
35
40
45
50
Day
150 mg
300 mg
0 mg

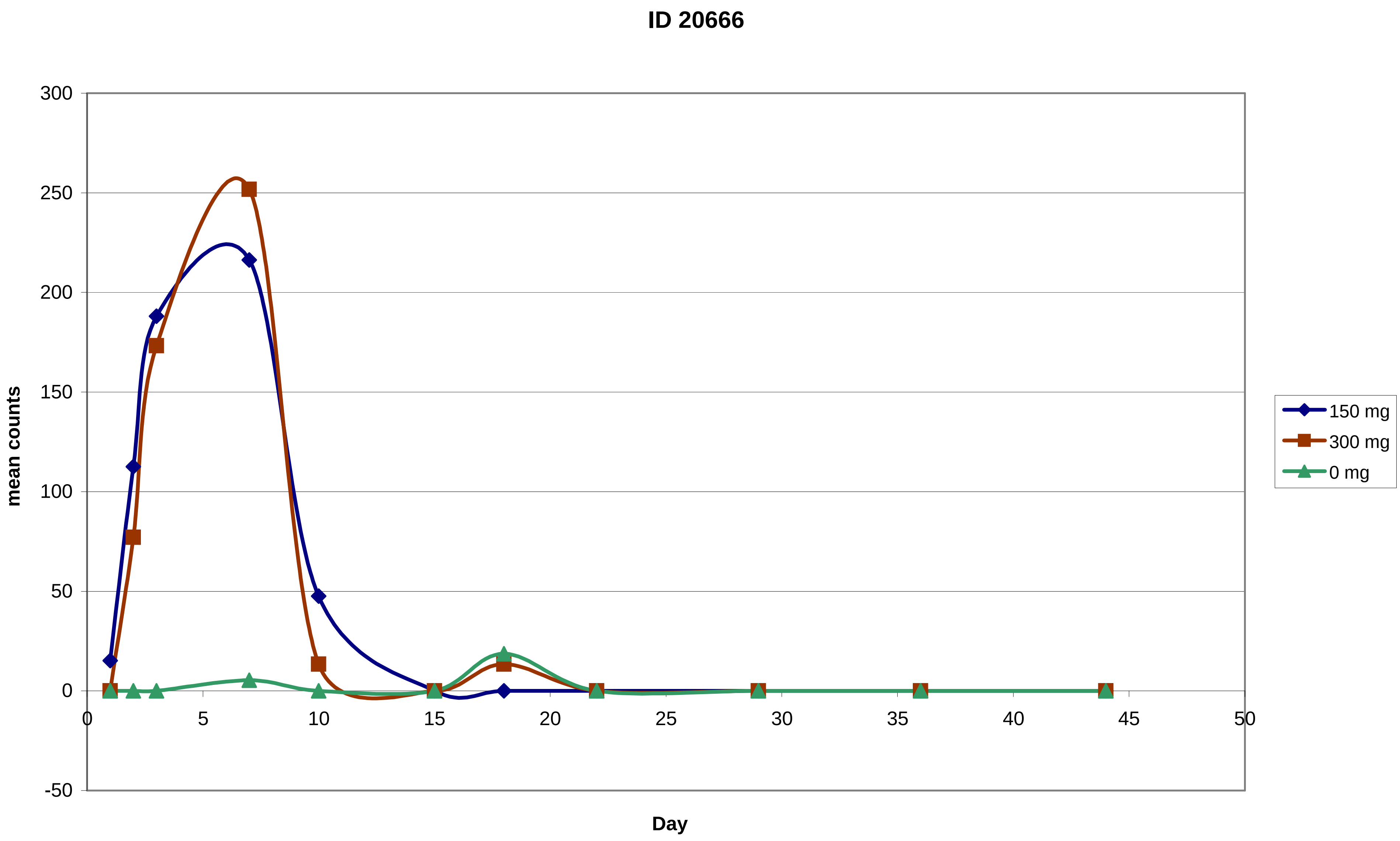

ID 20666
mean counts
300
250
200
150
100
50
0
-50
0
5
10
15
20
25
30
35
40
45
50
Day
150 mg
300 mg
0 mg

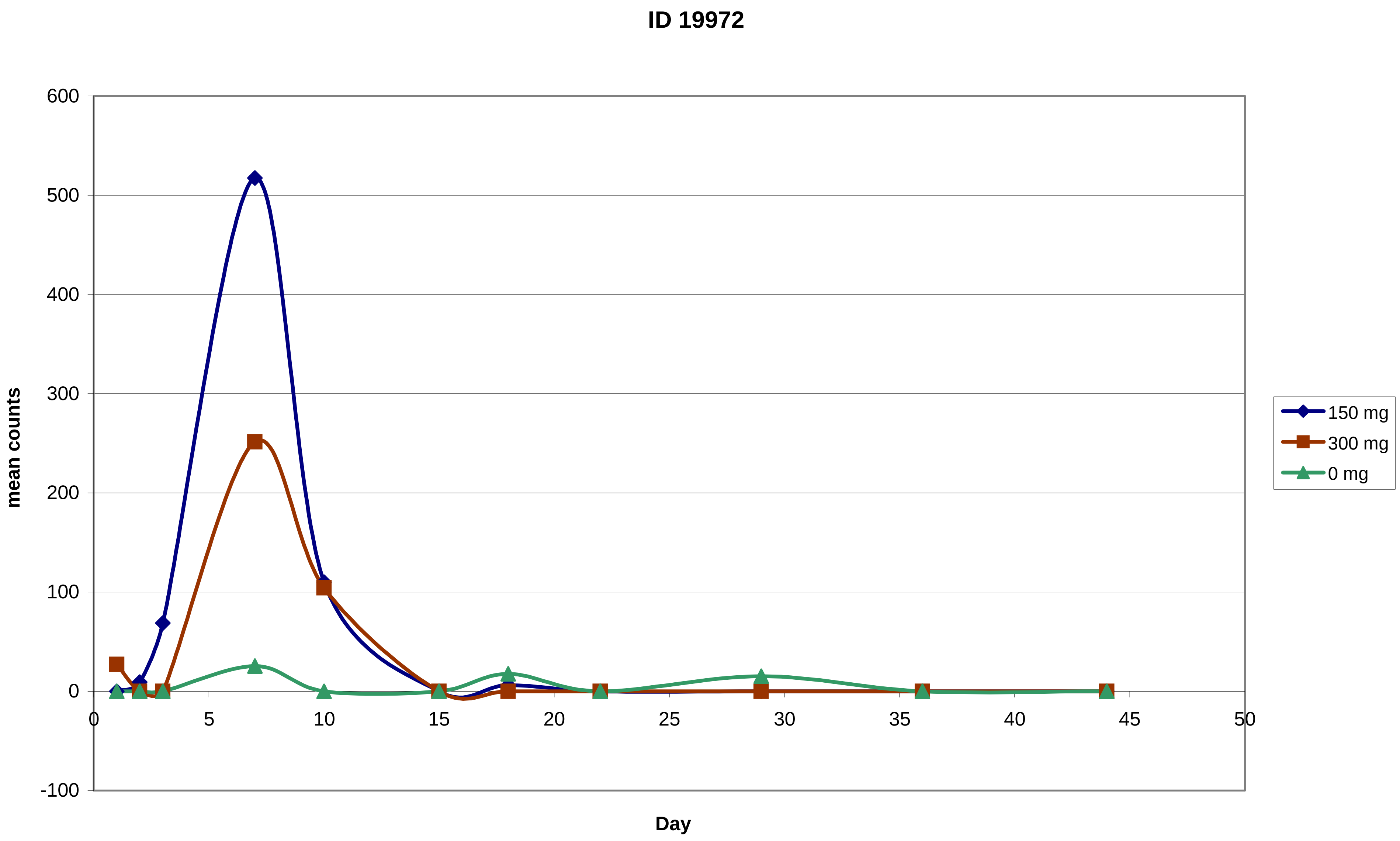
ID 19972
mean counts
600
500
400
300
200
100
0
-100
0
5
10
15
20
25
30
35
40
45
50
Day
150 mg
300 mg
0 mg

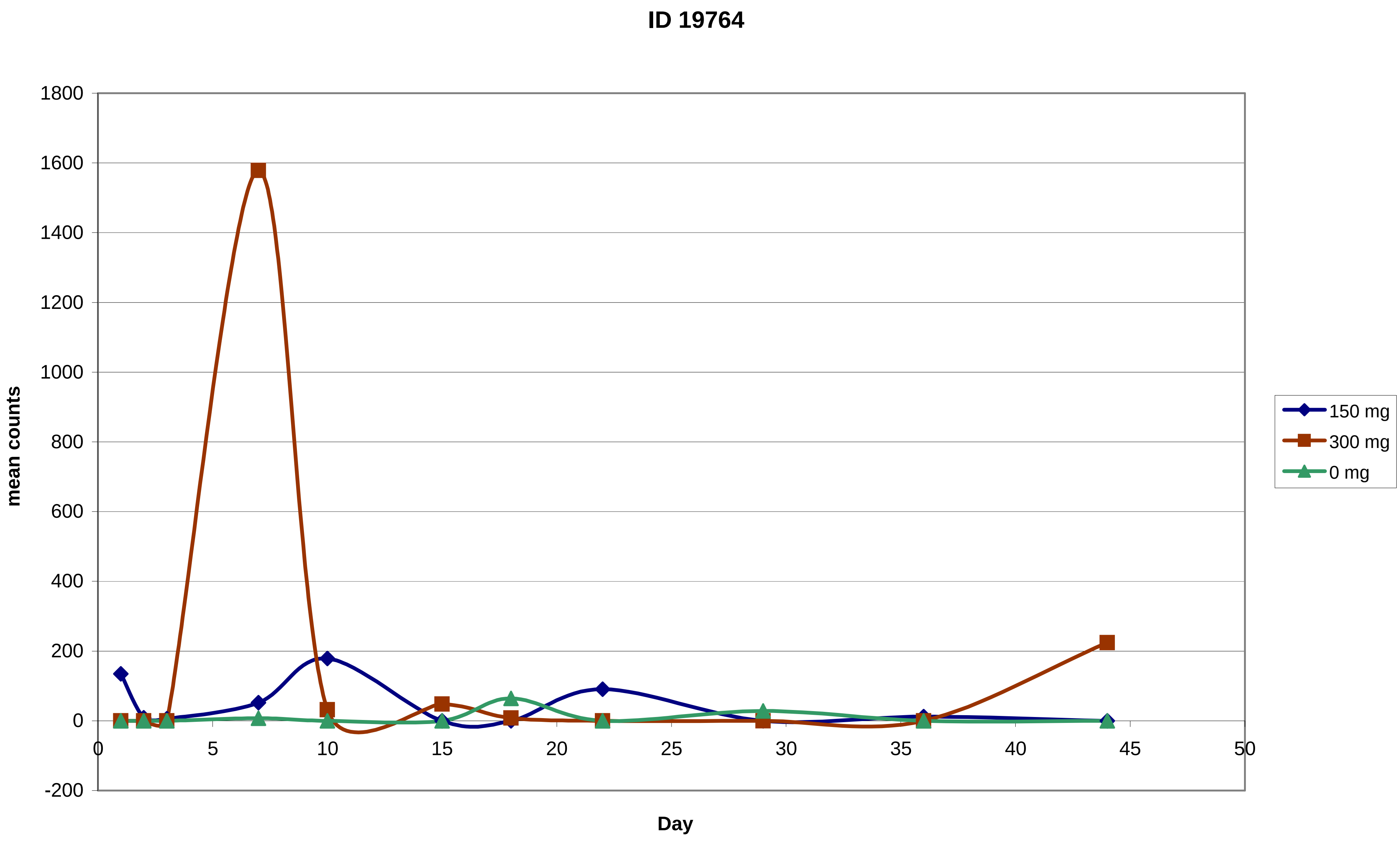
ID 19764
mean counts
1800
1600
1400
1200
1000
800
600
400
200
0
-200
0
5
10
15
20
25
30
35
40
45
50
Day
150 mg
300 mg
0 mg

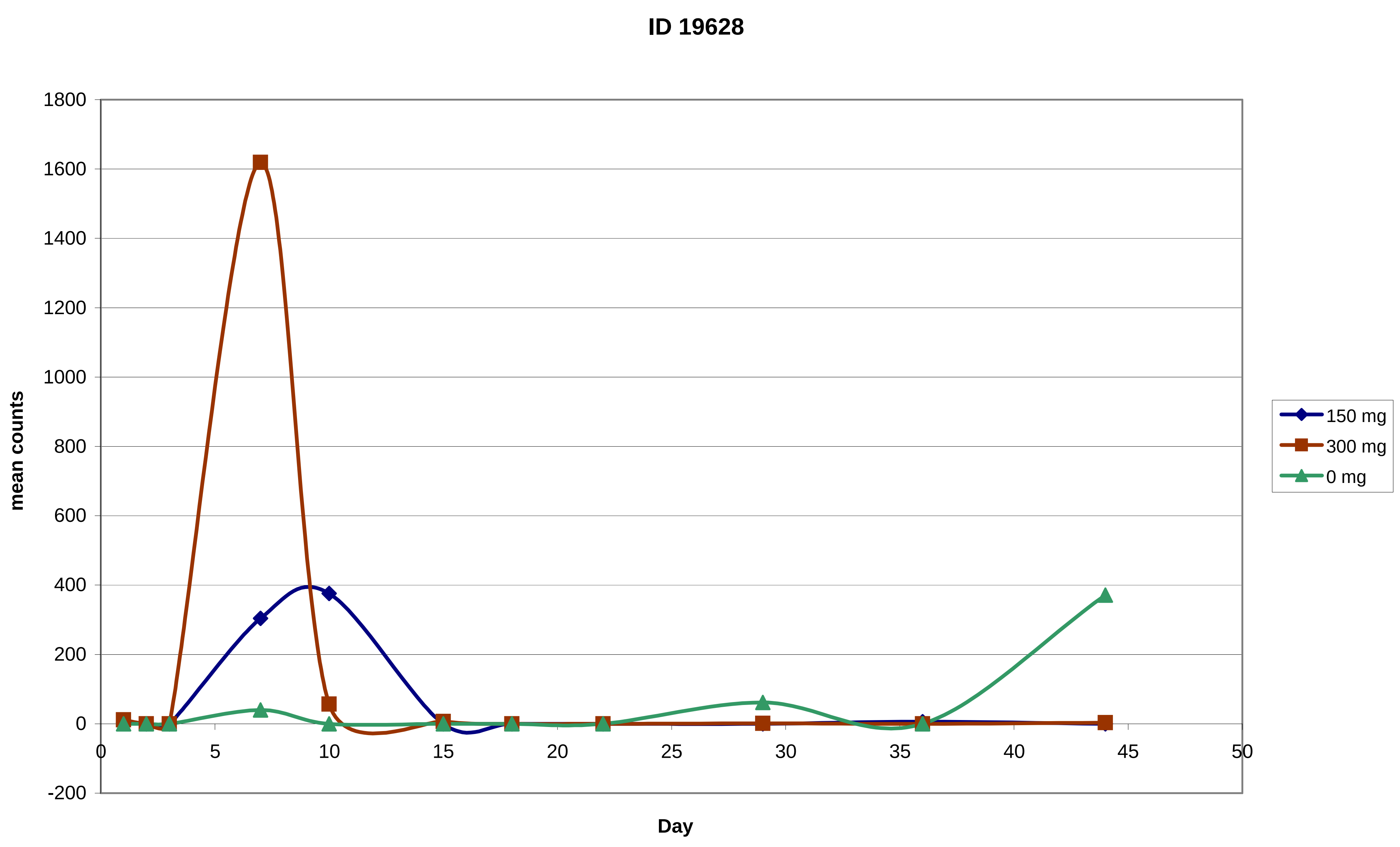

ID 19628
mean counts
Day
1800
1600
1400
1200
1000
800
600
400
200
0
-200
0
5
10
15
20
25
30
35
40
45
50
150 mg
300 mg
0 mg

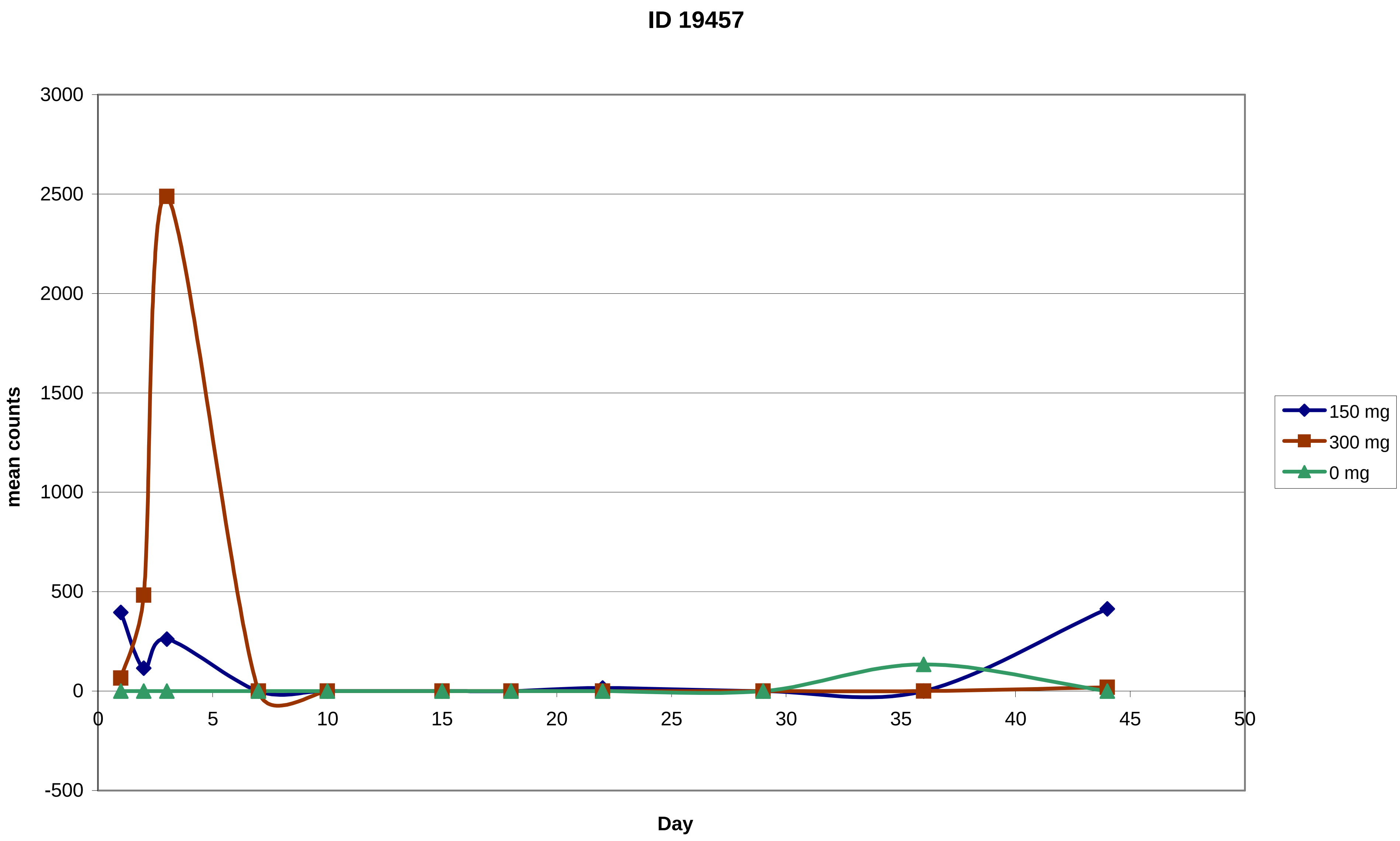
ID 19457
mean counts
3000
2500
2000
1500
1000
500
0
-500
0
5
10
15
20
25
30
35
40
45
50
Day
150 mg
300 mg
0 mg

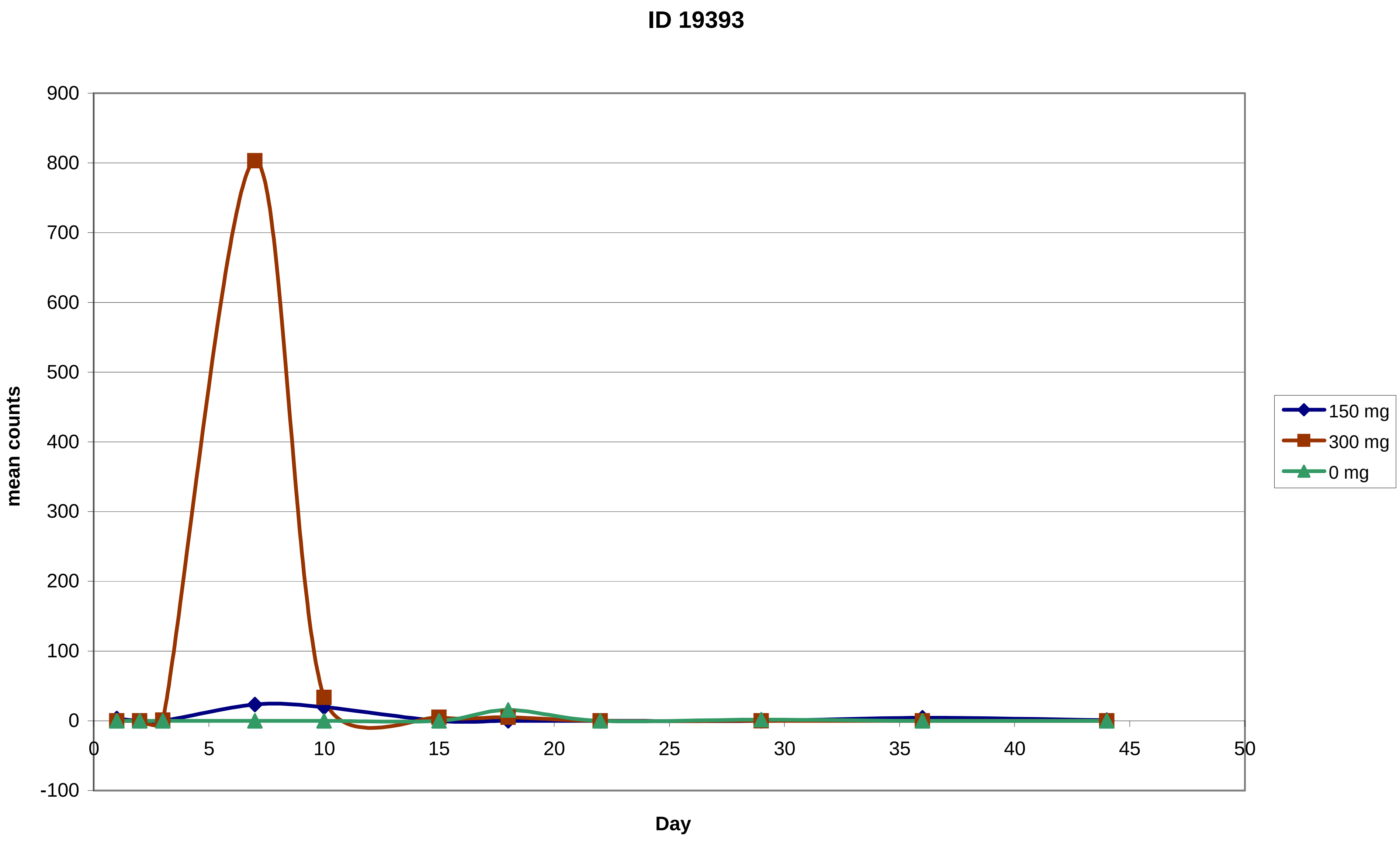
ID 19393
900
800
700
600
500
400
300
200
100
0
-100
mean counts
0
5
10
15
20
25
30
35
40
45
50
Day
150 mg
300 mg
0 mg

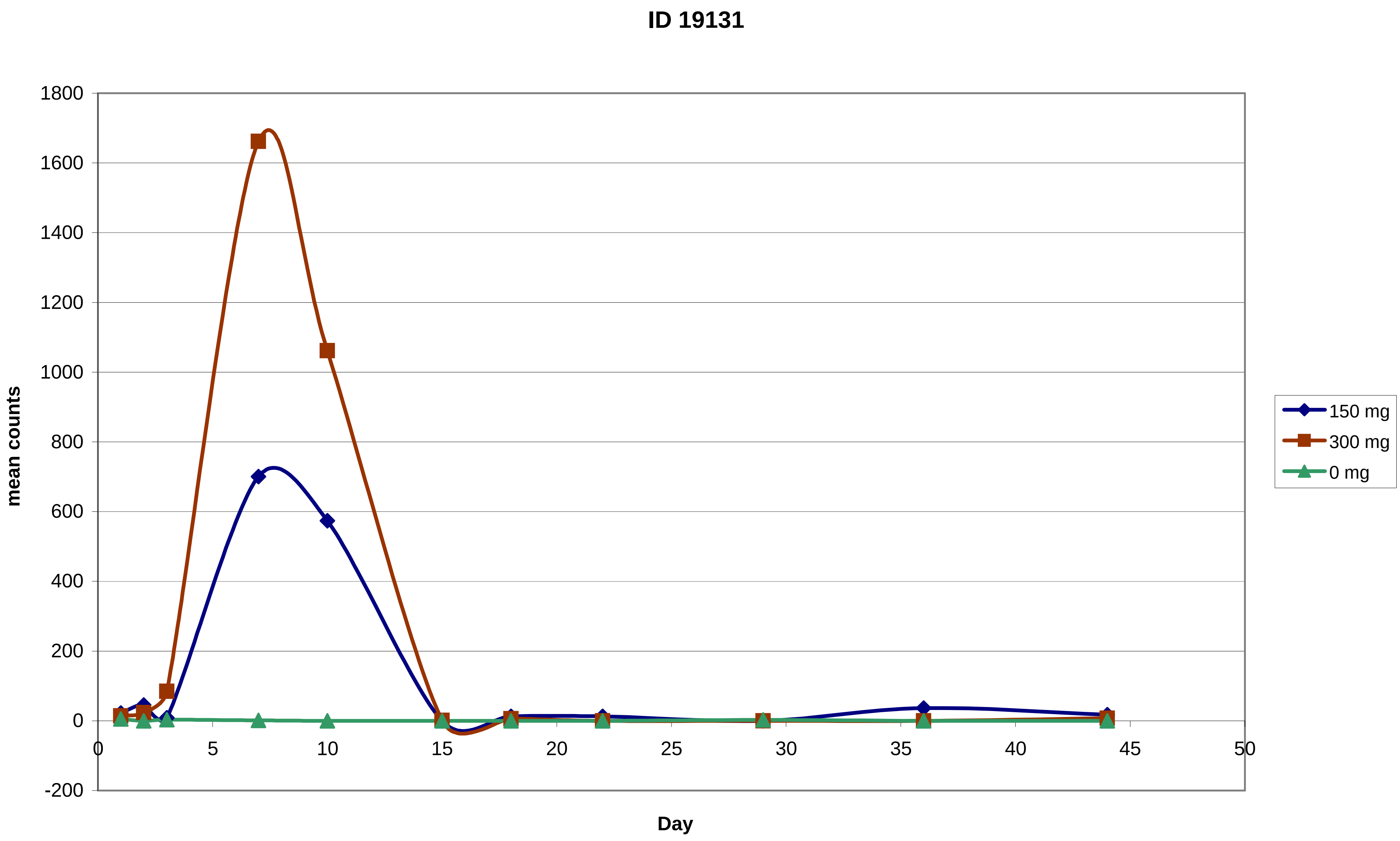
ID 19131
mean counts
Day
1800
1600
1400
1200
1000
800
600
400
200
0
-200
0
5
10
15
20
25
30
35
40
45
50
150 mg
300 mg
0 mg
